# Immunosuppressive effects of mesenchymal stem cells on lung B cell gene expression in LPS-induced acute lung injury

**DOI:** 10.1186/s13287-020-01934-x

**Published:** 2020-09-25

**Authors:** Bing Feng, Jiaqi Zhu, Yanping Xu, Wenyi Chen, Xinyu Sheng, Xudong Feng, Xiaowei Shi, Jingqi Liu, Qiaoling Pan, Jinfeng Yang, Jiong Yu, Lanjuan Li, Hongcui Cao

**Affiliations:** 1 State Key Laboratory for the Diagnosis and Treatment of Infectious Diseases, The First Affiliated Hospital, College of Medicine, Zhejiang University, 79 Qingchun Rd, Hangzhou City, 310003 China; 2 National Clinical Research Center for Infectious Diseases, 79 Qingchun Rd, Hangzhou City, 310003 China; 3 Zhejiang Provincial Key Laboratory for Diagnosis and Treatment of Aging and Physic-chemical Injury Diseases, 79 Qingchun Rd, Hangzhou City, 310003 China

**Keywords:** Mesenchymal stem cells, Lung B cells, Single-cell RNA sequencing, Acute lung injury

## Abstract

**Background:**

Immune system disorders play important roles in acute lung injury (ALI), and mesenchymal stem cell (MSC) treatment can reduce inflammation during ALI. In this study, we compared the changes in lung B cells during MSC treatment.

**Methods:**

We investigated the effects of MSCs on lung B cells in a mouse model of lipopolysaccharide (LPS)-induced ALI. MSCs were administered intratracheally 4 h after LPS. As vehicle-treated controls, mice were treated with phosphate-buffered saline (PBS) containing 2% C57BL/6 (PBS group). Histopathological changes, survival rate, inflammatory factor levels, and the number of neutrophils in bronchoalveolar lavage fluid (BALF) were determined. Single-cell RNA sequencing (scRNA-Seq) analysis was performed to evaluate the transcriptional changes in lung B cells between the PBS, LPS, and LPS/MSC groups on days 3 and 7.

**Results:**

MSC treatment ameliorated LPS-induced ALI, as indicated by the reductions in mortality, the levels of chemokines and cytokines in BALF, and the severity of lung tissue histopathology in ALI mice. Lung B cells in the PBS group remained undifferentiated and had an inhibitory phenotype. Based on our scRNA-Seq results, the differentially expressed genes (DEGs) in lung B cells in both the PBS group and LPS group were involved in chemotaxis processes and some proinflammatory pathways. MSC treatment inhibited the expression of chemokine genes that were upregulated by LPS and were related to the recruitment of neutrophils into lung tissues. Immunoglobulin-related gene expression was decreased in lung B cells of mice treated with LPS/MSC for 7 days. The DEGs regulated by MSCs were enriched in biological processes, including humoral immune response and apoptotic signaling.

**Conclusions:**

Lung B cells played an important role in the effects of treatment of ALI with MSCs. These observations provide new insights into the mechanisms underlying the effects of MSC treatment for ALI.

## Background

Acute lung injury (ALI) is a clinical manifestation of acute respiratory distress syndrome (ARDS). Its clinical features include bilateral pulmonary infiltrates, severe hypoxemia, and noncardiogenic pulmonary edema [1]. The mortality rate is high in ARDS patients; clinical therapy consists of protective mechanical ventilation, and no effective pharmacological treatments are available [1]. Lipopolysaccharide (LPS)-induced ALI is an animal model that has several of the classic pathological characteristics of ARDS [2]. Neutrophils play an important role in the severity and outcome of ARDS [3]. Neutrophil depletion in mice reduces the severity of lung injury [4]. Lee et al. reported that the chemokines, chemokine CC ligands (CCL)3 and CCL4, promote the local influx of neutrophils in vivo [5]. There have been some reports that B cells express and secrete CCL3/4 [6, 7]. The adaptive immune system, including B cells, also plays important roles in the pathogenesis of lung diseases. The presence of B cells in airway inflammatory infiltrates is correlated with disease severity in many airway diseases. Randall et al. reported that lung biopsies from patients with severe asthma often have B cell clusters [8]. Bosken et al. reported that the numbers of B cells and lymphoid follicles in the adventitia of the small airways were higher in cases of lung inflammation [9]. IgM, IgG, IgE, IgA, and IgD produced by B cells are also correlated with the progression of lung diseases. Cheng et al. reported that IgG, IgA, and IgM are correlated with the Global Initiative for Chronic Obstructive Lung Disease (GOLD) stage of chronic obstructive pulmonary disease (COPD) [10]. IgG and IgA levels are increased in patients with pigeon hypersensitivity pneumonitis (HP) as well as in asymptomatic pigeon breeders [11].

Mesenchymal stem cells (MSCs) can undergo self-renewal and differentiation into various cell types and tissues including chondrocytes, osteoblasts, and adipocytes [12]. Many studies have demonstrated that MSCs can suppress the activation and function of innate and adaptive immune cells, including macrophages [13], neutrophils [14], natural killer cells [15], dendritic cells [16], and T cells [17]. Many preclinical studies have demonstrated that treatment with MSCs can improve survival, reduce inflammation, and enhance bacterial clearance [18–21]. However, the mechanisms underlying these effects are not completely understood. Extensive research has shown that MSCs suppress stimulated B cell immunoglobulin production, proliferation, and differentiation into plasma cells. Asari et al. reported that MSCs exert a suppressive effect on the terminal differentiation of B cells in vitro and in vivo by releasing humoral factors [22]. Feng et al. reported that MSCs downregulate the expression of olfactory 1/early B cell factor-associated zinc-finger protein, which can reverse the inhibition of IgG and IgM production in B cells [23]. Che et al. also reported that umbilical cord MSCs can suppress IgM and IgG production in B cells [24]. The effects of MSCs on lung B cells during MSC treatment in ALI are still unclear. The present study was performed to investigate the changes in lung B cells associated with MSC treatment in a mouse model of LPS-induced ALI.

## Methods

### Analysis of chemokine and cytokine levels and cell identification in bronchoalveolar lavage fluid

Mice were killed and bronchoalveolar lavage fluid (BALF) was collected by gentle lavage of the lungs twice with 0.6 mL of phosphate-buffered saline (PBS). BALF was centrifuged for 5 min at 400×*g* and the supernatant was stored at − 80 °C until the experiments. The concentrations of chemokines and cytokines in BALF were determined using a LEGENDplex mouse chemokine panel and cytokine panel (BioLegend, London, UK). Cells in BALF were stained with Wright-Giemsa (BaSO, Zhuhai, China). The numbers of neutrophils per 200 cells were determined based on morphology.

### Lung morphology

Lungs were fixed in 4% paraformaldehyde, embedded in paraffin, cut into sections 5 μm thick, and stained with hematoxylin and eosin (H&E). Lung slices were scanned using a desktop single slide scanner (NanoZoomer-SQ; Hamamatsu Corp., Hamamatsu, Japan), and images of lung sections were captured at a magnification of × 20 using NDP.view.2 software (Hamamatsu Corp.).

### Induction of acute lung injury and MSC treatment

Male C57BL/6 mice, 6–8 weeks old, were purchased from Nanjing Biomedical Research Institute of Nanjing University and maintained in the Experimental Animal Center of Zhejiang University. Mice were treated intratracheally with 20 μg/g of lipopolysaccharide (*Escherichia coli* serotype 0111:B4; Sigma-Aldrich, St. Louis, MO). After 4 h, mice were treated intratracheally with 0.1 mL of PBS containing 2% C57BL/6 serum with or without 5 × 10^5^ MSCs. As a vehicle control group, an equal volume of PBS containing 2% C57BL/6 serum was administered (PBS group). The PBS group consisted of 5 mice, and the LPS and LPS/MSC groups each consisted of 10 mice. The mice were euthanized on days 3 or 7 after MSC or PBS administration, and lung tissues were collected for histological analysis and prepared for lung immune cell separation.

### Lung immune cell separation

After mice were euthanized, the lungs were cut into pieces and digested using a Mouse Lung Dissociation Kit (Miltenyi Biotec, Bergisch Gladbach, Germany). They were then homogenized using a gentleMACS C tube and a GentleMACS™ Dissociator (Miltenyi Biotec). The homogenates were filtered through a 100-μm cell strainer (Falcon®; Corning Inc., Corning, NY) and centrifuged for 10 min at 300×*g*. The pellets were resuspended with 36% Percoll and centrifuged for 5 min at 450×*g*. The pellets were resuspended in 3 mL of ACK (ammonium-chloride-potassium) lysing buffer and incubated for 5 min at room temperature to lyse red blood cells. The process was ended by the addition of 5 mL of PBS.

### Single-cell RNA sequencing

Separated single lung immune cells were positively selected with a magnetic-activated cell sorting (MACS) cell separation system (Miltenyi Biotec) using anti-mouse CD45 microbeads. The purified CD45^+^ cell fraction contained > 95% total separated cells as determined by flow cytometry (data not shown). Cell viability was determined by trypan blue staining. Single CD45^+^ lung immune cells (10^6^/mL) were suspended in calcium- and magnesium-free PBS containing 0.04% weight/volume bovine serum albumin (BSA). Single-cell RNA sequencing (scRNA-Seq) was performed using a Chromium Next GEM Single Cell 3′ GEM, Library & Gel Bead Kit v3.11, 4 rxns (10x Genomics, Pleasanton, CA). The libraries were quantified by Qubit and sequenced on an Illumina HiSeq 4000 next-generation sequencing platform (Illumina, San Diego, CA). CellRanger version 3.0.0 (10x Genomics) was used for data quality analysis and mapping to the Ensembl gene symbols.

### scRNA-Seq data processing

Raw scRNA-Seq data per sample from CellRanger were combined in R (version 3.6.1; R Foundation for Statistical Computing, Vienna, Austria) and transformed to a Seurat object using the Seurat R package (version 3.1.2). We selected B cell clusters from the first analysis. Based on the expression of nGene, nUMI, and percent.mito, we removed doublets and damaged cells. The percentage of mitochondrial genes for each sample was < 0.1% (data not shown), suggesting that our separated lung immune cells were of high quality. Due to the treatment and time differences of our samples, filtering was carried out individually for each sample. We normalized gene expression data with the LogNormalize function in the Seurat package. In total, 2000 variable genes were taken to run a graph-based method (resolution = 0.3) and t-distributed stochastic neighbor embedding (tSNE) method to cluster and reduce the dimensions. Differentially expressed genes (DEGs) of samples were used for the Findmarker function and were selected according to *P* < 0.05 and fold change > 1.2, as shown in Tables S1–3. The marker genes of each cluster were obtained with the FindAllMarker function in the Seurat package. Table S4 lists all marker genes of each cluster. Gene ontology (GO) and Kyoto Encyclopedia of Genes and Genomes (KEGG) enrichment and gene set enrichment analysis (GSEA) were performed with the clusterProfiler package [25]. *P* < 0.05 was taken to indicate statistical significance. The enrichplot package was used to visualize the enrichment data.

### Statistical analysis

GraphPad Prism (version 6.0; GraphPad Software, San Diego, CA), Seurat R package, and the clusterProfiler package were used for data analysis. The unpaired Student’s *t* test, Kaplan-Meier test, or Wilcoxon test was used to compare differences between the two groups, as appropriate. tSNE plot and violin plot were generated in the Seurat and ggplot2 R package. GSEA, GO, and KEGG enrichment were performed using the clusterProfiler package. The enrichplot package was used to visualize the enrichment data. Data are presented as the mean ± standard error of the mean. In all analyses, *P* < 0.05 was taken to indicate statistical significance.

## Results

### MSCs ameliorate ALI

**Fig. 1:**
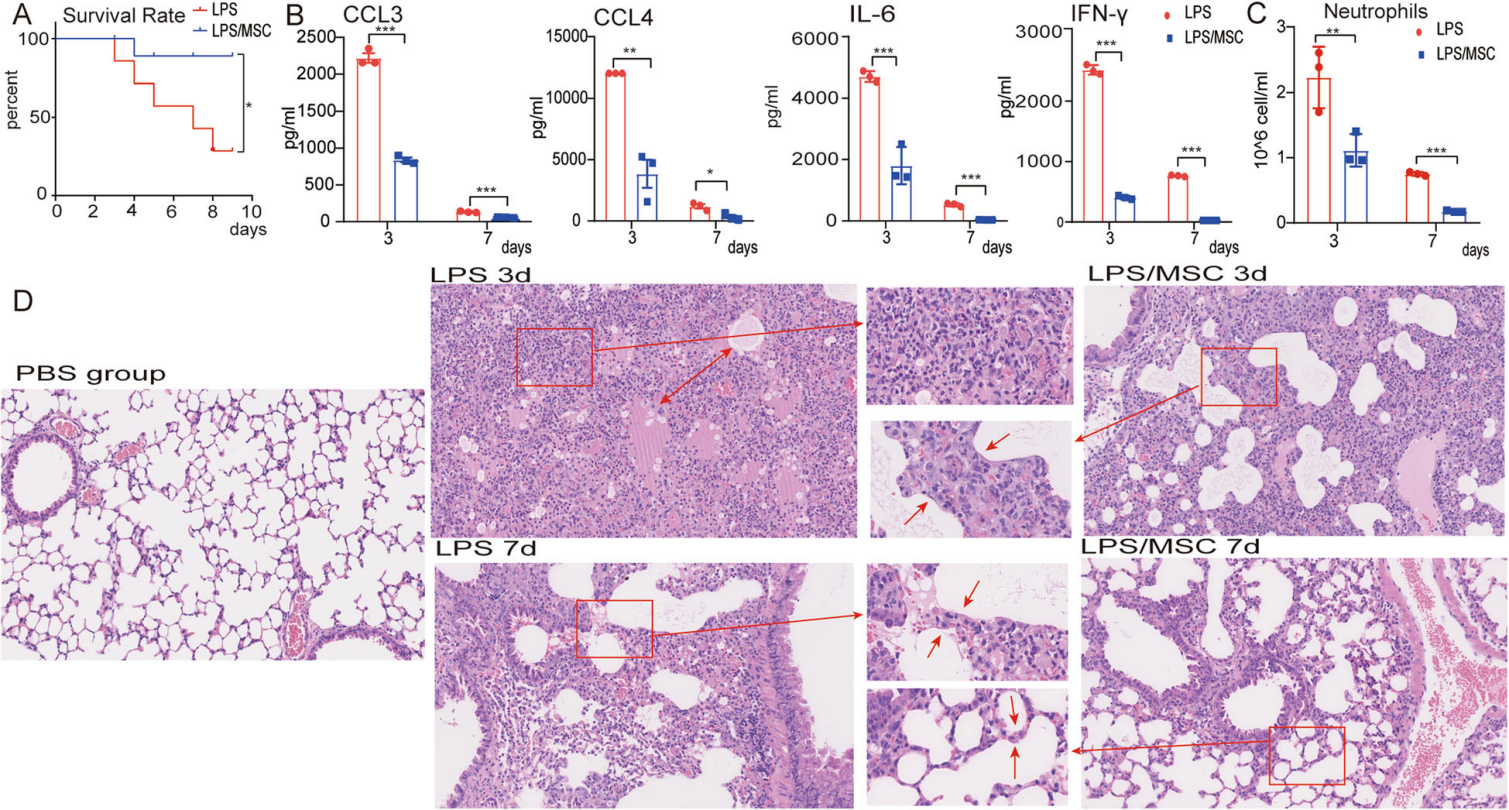
MSCs ameliorate LPS-induced ALI. **a** Survival curve of mice treated with LPS and MSCs. **b** CCL4, CCL3, IL-6, and IFN-γ concentrations in BALF at 3 and 7 days after LPS or LPS/MSC treatment; data are means ± SEM (*n* = 3 per group). **c** Numbers of neutrophils in BALF in different groups; data are means ± SEM (*n* = 3 per group). **d** H&E staining of lung sections at PBS group, 3 and 7 days after LPS and LPS/MSC treatment. Red arrows indicating cell infiltration in the alveolar interstitium and thickening of the alveolar interstitium. On day 3 after LPS treatment, many immune cells, especially neutrophils, infiltrated into the alveolar interstitium, while thinner alveolar interstitium and reduced immune cell infiltration were observed on day 3 after MSC treatment (damage phase). On day 7 after LPS and MSC treatment, the alveolar interstitium became thinner than day 3, and ALI had recovered almost completely at day 7 (recovery phase). *P < 0.05, **P < 0.01, ***P < 0.001 unpaired Student’s *t* test

To evaluate the efficacy of MSCs in ALI, we recorded survival rate, histopathology, and the cytokine and chemokine levels in BALF after MSC treatment. At 7 days after MSC treatment, LPS-treated mice had a significantly higher survival rate (*P* < 0.05) than mice that received LPS alone (Fig. 1a). The levels of chemokines CCL3 and CCL4 were significantly decreased in the MSC-treated LPS-induced ALI model mice, but were upregulated in the LPS-only group at 3 and 7 days (Fig. 1b). The levels of the cytokines, interleukin (IL)-6 and interferon (IFN)-γ, were also decreased in LPS-induced ALI model mice after MSC treatment at 3 and 7 days (Fig. 1b). The neutrophil numbers were decreased at 3 and 7 days after MSC treatment (Fig. 1c). After LPS administration, typical pathological changes, including infiltration of large numbers of cells into the alveolar interstitium and thickening of the alveolar walls and interstitium, were observed under the microscope (Fig. 1d). In contrast, the LPS/MSC group had thinner alveolar walls and interstitium and reduced immune cell infiltration. ALI was severe at 3 days (damage phase) and had recovered almost completely at 7 days (recovery phase) after treatment. Therefore, MSC administration significantly alleviated LPS-induced ALI.

### Chemokine gene expression in B cells is induced during ALI

**Fig. 2:**
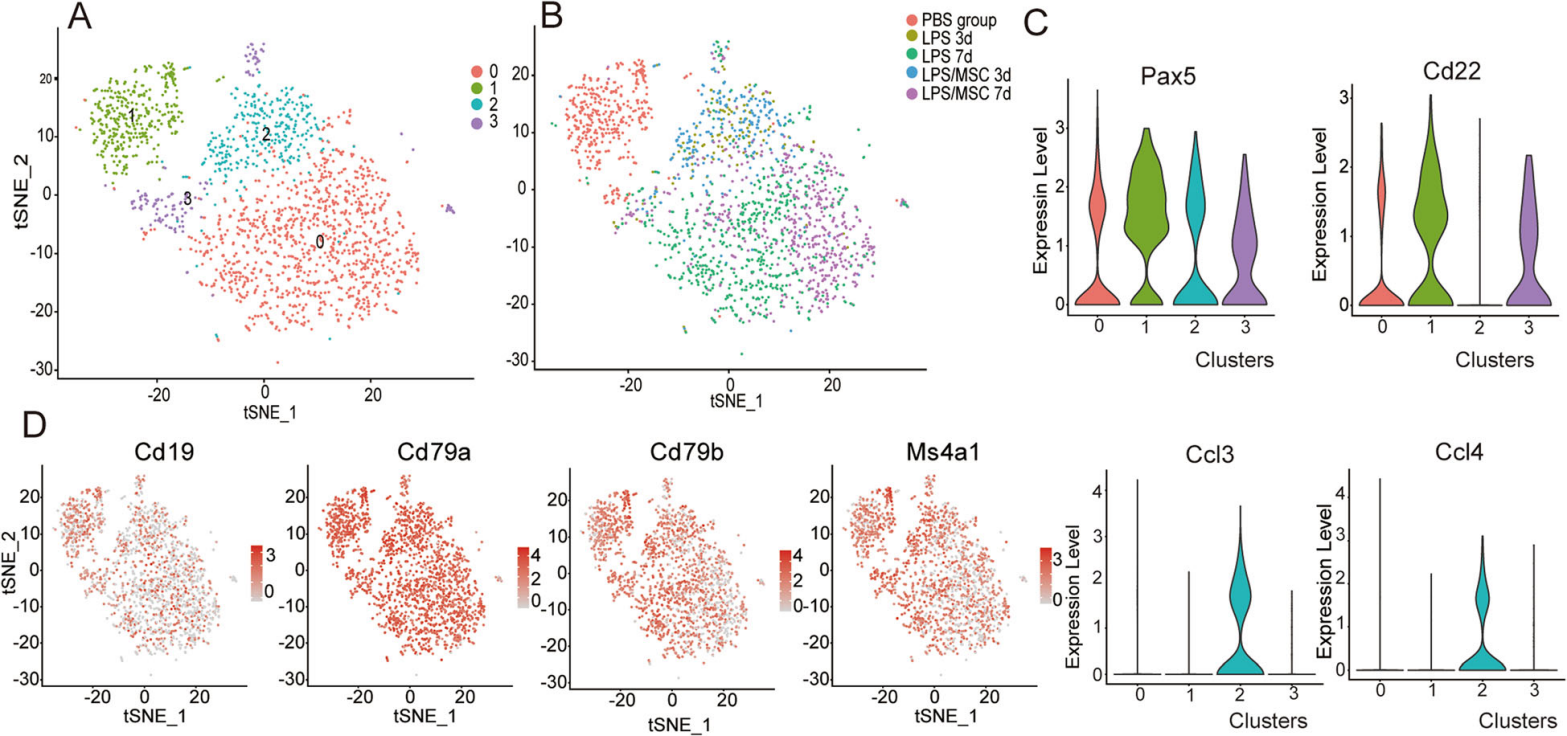
High-dimensional transcriptomic scRNA-Seq clustering of lung B cell compartment. **a** tSNE map of different scRNA-Seq clusters identified by graph-based method. **b** tSNE map colored by different groups. **c** Violin plots showing the log-transformed expression of selected genes: *Pax5*, *Cd22*, *Ccl3*, and *Ccl4*. **d** tSNE map of expression of B cell marker genes: *Cd19*, *Cd79a*, *Cd79b*, and *Ms4a1*

We separated lung immune cells and performed scRNA-Seq analysis to analyze transcriptional changes in B cells after LPS treatment. Using a graph-based method and visualization by tSNE, we found four B cell clusters (Fig. 2a). tSNE maps of the B cell marker genes, including *Cd79a*, *Cd79b*, *Ms4a1*, and *Cd19*, are shown in Fig. 2d. The top 30 marker genes of each cluster based on fold change are shown in Fig. S2. *Pax5* and *Cd22* were highly expressed in cluster 1 (Fig. 2c), which corresponded mainly to the PBS group (Fig. S3). *Ccl3* and *Ccl4* were highly expressed in cluster 2 (Fig. 2c) and mainly corresponded to 3 days after LPS or LPS/MSC treatment (Fig. S3).

**Fig. 3:**
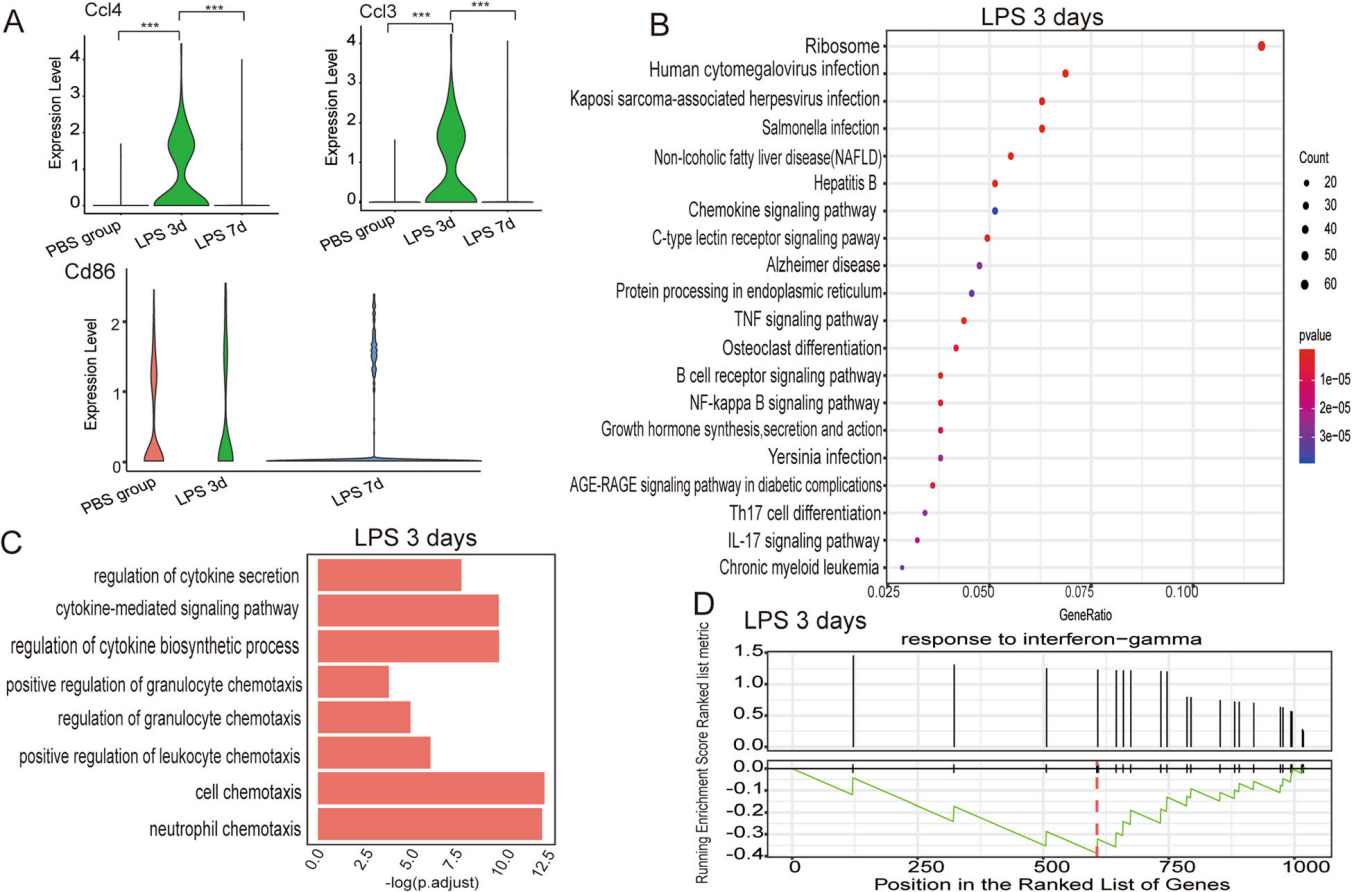
scRNA-Seq identification of lung B cell compartment in the vehicle control and LPS groups. **a** Violin plots showing the log-transformed expression of selected genes, *Ccl3*, *Ccl4*, and *Cd86*, in the PBS group, and in mice at 3 and 7 days after LPS treatment. *P < 0.05, **P < 0.01, ***P < 0.001, Wilcoxon test. **b** Top 20 functional enrichment analysis with KEGG analysis using DEGs between the PBS group and mice at 3 days after LPS treatment. The *x*-axis represents the gene ratio. **c** Bar plot of the DEGs between the PBS group and mice at 3 days after LPS treatment involved in biological process terms of GO functional enrichment analysis. **d** Gene set enrichment analysis **(**GSEA) of the response to IFN-γ (GO: 0071346) in the DEGs between the vehicle control group and 3 days after LPS treatment. Black bars indicate positions of the response to IFN-γ in the ordered list of genes. The green line indicates line of running enrichment score. The red broken line represents the gene with the highest enrichment score. *P* < 0.01

We compared the DEGs between the PBS group and 3 days after LPS treatment. The results revealed that 261 genes were upregulated and 763 were downregulated after LPS treatment (Table S1). Among the DEGs (fold change > 1.2, *P* < 0.05), chemokine genes *Ccl3* and *Ccl4* were highly expressed 3 days after LPS treatment and decreased 7 days after LPS treatment (Fig. 3a). The trends in changes in expression of the costimulatory molecule *Cd86* were also consistent with the changes in chemokine gene expression, but the two groups did not differ significantly (Fig. 3a). The chemokine expression profiles were consistent with the results of the histopathological analysis. Three days after LPS treatment, ALI was severe, many cells infiltrated the alveolar space, the alveolar walls and interstitium were thickened, and the gene expression levels of chemokines CCL3 and CCL4 were markedly increased. On day 7, the injury had almost recovered and the expression levels of chemokines had decreased.

### DEGs regulated by LPS in lung B cells were involved in proinflammatory pathways

We next examined which pathways were involved in the differential gene expression between the PBS group and the LPS group 3 days after treatment. Enrichment analysis on KEGG using the clusterProfiler package identified genes involved in proinflammatory pathways, including tumor necrosis factor (TNF) signaling pathway, nuclear factor (NF)-κB signaling pathway, and T helper (Th)17 differentiation (Fig. 3b). The biological processes were regulated by LPS, including regulation of cytokine secretion (GO:0050707), cytokine-mediated signaling pathway (GO:0019221), regulation of cytokine biosynthetic process (GO:0042035), positive regulation of granulocyte chemotaxis (GO:0071624), regulation of granulocyte chemotaxis (GO:0071622), positive regulation of leukocyte chemotaxis (GO:0002690), cell chemotaxis (GO:0060326), and neutrophil chemotaxis (GO:0030593) (Fig. 3c). Lung B cells 3 days after LPS treatment exhibited upregulation of multiple genes induced by IFN, including *Ifi47*, *Irf1*, *Ifitm1*, and *Ifitm2* (fold change > 1.2, *P* < 0.05, Table S1). The GSEA of GO biological processes also revealed that the response to IFN-γ was activated in lung B cells at 3 days after LPS treatment (Fig. 3d). These results indicate that chemokine gene expression in lung B cells was stimulated and proinflammatory pathways were activated at 3 days after LPS treatment during ALI.

### Chemokine gene and immunoglobulin expression in lung B cells are decreased by MSC treatment

**Fig. 4:**
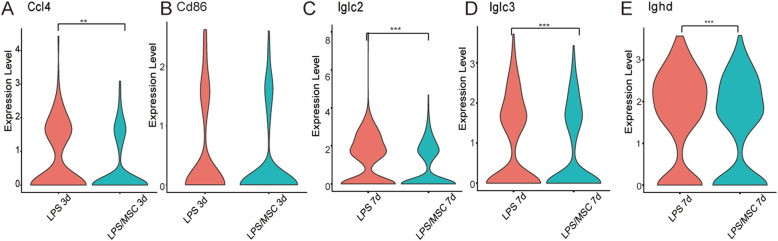
Expression levels of chemokine- and immunoglobulin-related genes in lung B cells were decreased by MSC treatment. Violin plots showing the log-transformed expression of selected genes: *Ccl4* (**a**) and *Cd86* (**b**) at 3 days after LPS and LPS/MSC treatment; *Iglc2* (**c**), *Iglc3* (**d**), and *Ighd* (**e**) at 7 days after LPS and LPS/MSC treatment. *P < 0.05, **P < 0.01, ***P < 0.001, Wilcoxon test

In total, 189 DEGs were downregulated and 73 were upregulated in lung B cells at 3 days after MSC treatment, and 207 DEGs were downregulated and 166 were upregulated in lung B cells at 7 days after MSC treatment (Fig. S4). *Ccl4* expression was reduced 3 days after MSC treatment (Fig. 4a). However, *Ccl3* was not markedly reduced at 3 days after MSC treatment (Fig. S5). *Cd86* expression was reduced (fold change > 1.2, *P* > 0.05) 3 days after MSC treatment (Fig. 4b). *Iglc2* (fold change > 3, *P* < 0.05) and *Iglc3* (fold change > 1.2, *P* < 0.05) expression were both markedly reduced 7 days after MSC treatment (Fig. 4c, d). *Ighd* expression was also reduced by MSC treatment (Fig. 4e). However, *Iglc2*, *Iglc3*, and *Ighd* were not included in the DEGs at 3 days after LPS treatment and MSC treatment (Table S2).

### Predicted function and pathway enrichment of DEGs regulated by MSCs

**Fig. 5:**
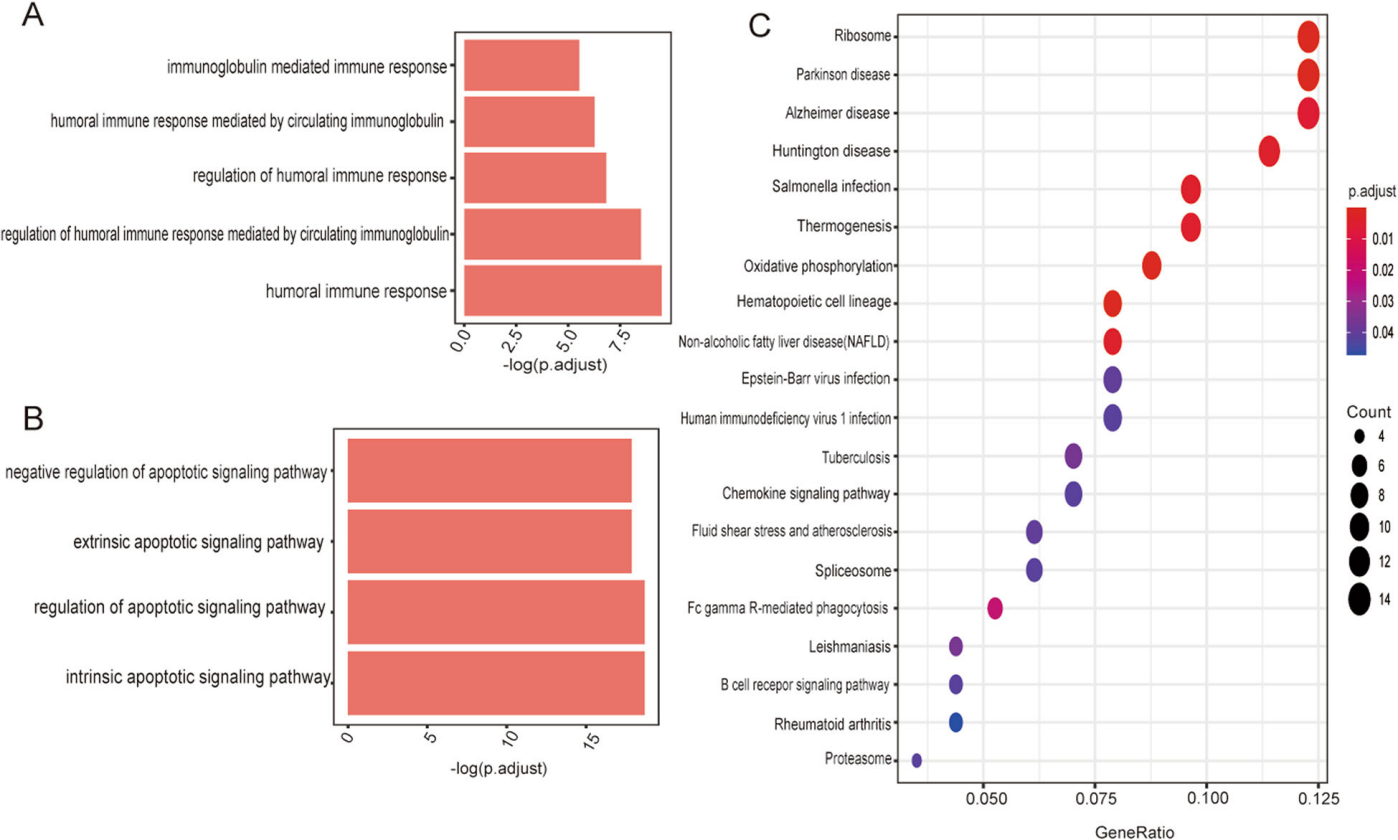
Predicted functions and pathways with enrichment of DEGs regulated by MSCs. The downregulated genes (**a**) and upregulated genes (**b**) by MSCs after 7 days involved in selected biological process terms on GO functional enrichment analysis. **c** Dot plot of the downregulated DEGs at 7 days after LPS and LPS/MSC treatment involved in KEGG enriched terms

Analysis of the alterations in gene expression in lung B cells between LPS/MSC and LPS groups identified the functions and pathways involved in DEGs regulated by MSCs. The genes downregulated by MSCs were involved in biological processes, such as humoral immune response (GO:0051016) and immunoglobulin-mediated immune response (GO:0030220) (Fig. 5a). The genes upregulated by MSCs were involved in some biological processes related to apoptotic signaling pathways (Fig. 5b). In KEGG analysis, inflammatory pathways, such as the chemokine signaling pathway and B cell receptor signaling pathway, were associated with the DEGs downregulated by MSCs (Fig. 5c).

## Discussion

ALI is a serious lung disease with a high mortality rate, and no efficacious therapies are currently available. There is a great deal of preclinical evidence that MSC therapy can reduce lung injury, but the involvement of immune cells during MSC treatment of ALI is a dynamic and complex process, and the mechanisms of action on immune cells, especially B cells, are still unclear. Our results were consistent with previous reports that MSCs have potential for treating ALI. Histological appearance and survival curves indicated the efficacy of MSC treatment of ALI. MSCs inhibited ALI proinflammatory cytokines (CCL3 and CCL4) and chemokine production (IL-6 and IFN-γ), which were mainly expressed by alveolar epithelial cells, effector T cells, and macrophages.

Using scRNA-Seq, we found four lung B cell clusters. Lung B cells were mostly in cluster 2 on day 3 after treatment with LPS/MSC and LPS and mostly in cluster 0 on day 7. The numbers of clusters differed minimally between the LPS and LPS/MSC groups, indicating that B cells also differed minimally between these two groups. Cluster 1 mainly corresponded to the PBS group and had higher levels of *Pax5* and *Cd22* expression. Pax5 is a key transcription factor controlling the differentiation of B cells and has been reported to repress the expression of genes associated with plasma cell development and function, including immunoglobulin genes (IgL, J chain) [26]. CD22 is an inhibitory co-receptor of the B cell receptor that is exclusively expressed on B cells and is a regulatory molecule that prevents overactivation of the immune system and development of autoimmune diseases [27]. B cells remained undifferentiated and had an inhibitory phenotype in the PBS group.

Although B cells differed minimally between the LPS and LPS/MSC groups, we compared proinflammatory factors in lung B cells between LPS and LPS/MSC groups. After LPS treatment, chemokine genes including *Ccl3*, *Ccl4*, and *Cxcl10* were upregulated (fold change > 1.5, *P* < 0.05, Table S1) in lung B cells. Furthermore, the DEGs were involved in proinflammatory signaling pathways, such as the TNF signaling pathway, Th17 differentiation, and NF-κB signaling pathway, and in biological processes, such as cell chemotaxis. Multiple genes induced by IFN, which involved increased expression in BALF, such as *Ifi47*, *Irf1*, *Ifitm1*, and *Ifitm2* (fold change > 1.2, *P* < 0.05, Table S1), were also upregulated in lung B cells 3 days after LPS treatment. The results of DEG analysis indicated that MSC treatment inhibited lung B cell chemokine *Ccl4* expression. Neutrophils play an important role in the severity and outcome of ALI [3, 28]. Neutrophils infiltrate the lungs and migrate to the airways where they express proinflammatory cytokines such as IL-1β and TNF-α in ALI [29] and release reactive oxygen species, cytotoxic molecules, and proteases. These molecules trigger a variety of chemotactic signals that result in positive feedback and enhanced inflammation [30]. In animal experiments, neutrophil depletion was shown to reduce the severity of lung injury [4]. Lee et al. also reported that the chemokines CCL3 and CCL4 promote the local influx of neutrophils in vivo [5]. The neutrophil number was significantly decreased by MSC treatment. MSCs inhibited lung B cell expression of chemokine genes that recruit neutrophils into the lung tissue, which may contribute to the reduction of neutrophils and the efficacy of MSC treatment in the acute phase of ALI.

B cells are classically associated with antibody production, and we found that MSCs decreased expression of *Iglc2*, *Iglc3*, and *Ighd* after 7 days, while these genes were not included in the DEGs at 3 days after LPS and MSC treatment (Table S2). IgA, IgM, IgD, IgG, and IgE play roles in the lungs under physiological as well as pathological conditions. IgE antibodies are associated with asthma. The number of specific IgG- and IgE-producing pulmonary plasma cells is increased after pulmonary ovalbumin exposure [31]. Cheng et al. reported that IgG, IgA, and IgM are correlated with the GOLD stage of COPD [10]. Previous studies have demonstrated that secreted IgD antibodies are frequently polyreactive and recognize respiratory bacteria, such as *Moraxella catarrhalis* and *Haemophilus influenzae*, but have a high rate of autoreactivity [32]. Therefore, immunoglobulin production by lung B cells plays an important role in lung inflammatory diseases. Due to the immunosuppressive function of MSCs, extensive studies have explored the effects of MSCs on B cells. MSCs mainly suppress B cell proliferation, plasma cell differentiation, and immunoglobulin production. In MSC transplantation, soluble factors, including membrane vesicles (containing IL-6 and IL-8) [33] and galectin-9 [34], can suppress immunoglobulin production in B cells. The number of lung B cells expressing *Cd86* was lower after MSC treatment. CD86 was reported to boost the activity of B cells and increase expression of IgG1 and IgG2a isotypes [35, 36]. MSC treatment can suppress immunoglobulin expression in lung B cells in the recovery phase of ALI, which may contribute to the reduction of lung inflammation. The functions and pathways with enrichment of DEGs regulated by MSCs also revealed that MSCs have an immunomodulatory function in treatment of ALI. However, biological processes such as CD4^+^ T cell activation and differentiation and positive regulation of cellular catabolic processes were included in the GO analysis (Fig. S6), and further experiments are required to confirm the functions of lung B cells during MSC treatment.

This study had some limitations. The number of B cells at 3 days after LPS treatment was small, which may limit ex vivo study. The number of lung B cells expressing IgG- and IgM-related genes was also small. The effects of MSCs on antibody production in lung B cells may require longer observation periods.

## Conclusions

The results of the present study demonstrated that MSCs have potential in treatment of ALI. The effects of MSCs on ALI are associated with their immunosuppressive function in lung B cells, including decreased expression of chemokines that are related to recruiting neutrophils and immunoglobulin production in lung B cells. Our results provide new insights into the mechanisms underlying the effects of MSC treatment in ALI.

## Supplementary information

**Additional file 1: Fig. S1.** Characteristics of MSCs. (A) MSCs were spindle-shaped in passage 3. MSCs differentiated into adipocytes (B) and osteocytes (C). (D) FACS analysis of MSCs using monoclonal antibodies including CD29, CD44, SCA-1, CD45, CD86, CD31, CD11b and MHC-Ia. **Fig. S2.** Heatmap of top 30 marker genes according to foldchange of each cluster identified using cluster specific DEGs. **Fig. S3.** Proportion of clusters in different groups. The red bars represent cluster 0, the green bars represent cluster 1, the cyan bars represent cluster 2, and the purple bars represent cluster 3. **Fig. S4.** Volcano plot shows the DEGs of LPS and LPS/MSC groups. *P* values were calculated using Wilcoxon test. **Fig. S5.** Violin plots show the log-transformed expression of *Ccl3* at 3 days after LPS and LPS/MSC treatment. **Fig. S6.** Selected biological processes predicted by GO functional enrichment analysis using the upregulated genes by MSCs after 7 days. **Table S1.** Results of differential gene expression analysis between the PBS group and mice at 3 days after LPS treatment. **Table S2.** Results of differential gene expression analysis at 3 days after LPS and LPS/MSC treatment. **Table S3.** Results of differential gene expression analysis at 7 days after LPS and LPS/MSC treatment. **Table S4.** All markers of each cluster.

## Data Availability

All data generated or analyzed during this study are included in this article.

## Abbreviations

ALI: Acute lung injury
ARDS: Acute respiratory distress syndrome
BALF: Bronchoalveolar lavage fluid
DEGs: Differential expression genes
GO: Gene ontology
GSEA: Gene set enrichment analysis
GOLD: Global Initiative for Chronic Obstructive Lung Disease
HP: Hypersensitivity pneumonitis
IFN: Interferon
IL: Interleukin
KEGG: Kyoto Encyclopedia of Genes and Genomes
LPS: Lipopolysaccharide
MSCs: Mesenchymal stem cells
scRNA-seq: Single-cell RNA sequencing
tSNE: t-Distributed stochastic neighbor embedding
