# Supplementary material for: Immunosuppressive effects of mesenchymal stem cells on lung B cell gene expression in LPS-induced acute lung injury
Source: Stem Cell Res Ther. 2020 Sep 25;11:418. doi: 10.1186/s13287-020-01934-x (PMC7517809; doi:10.1186/s13287-020-01934-x)
Supplement: Supplementary file 1 — Additional file 1: Fig. S1. Characteristics of MSCs. (A) MSCs were spindle-shaped in passage 3. MSCs differentiated into adipocytes (B) and osteocytes (C). (D) FACS analysis of MSCs using monoclonal antibodies including CD29, CD44, SCA-1, CD45, CD86, CD31, CD11b and MHC-Ia. Fig. S2. Heatmap of top 30 marker genes according to foldchange of each cluster identified using cluster specific DEGs. Fig. S3. Proportion of clusters in different groups. The red bars represent cluster 0, the green bars represent cluster 1, the cyan bars represent cluster 2, and the purple bars represent cluster 3. Fig. S4. Volcano plot shows the DEGs of LPS and LPS/MSC groups. P values were calculated using Wilcoxon test. Fig. S5. Violin plots show the log-transformed expression of Ccl3 at 3 days after LPS and LPS/MSC treatment. Fig. S6. Selected biological processes predicted by GO functional enrichment analysis using the upregulated genes by MSCs after 7 days. Table S1. Results of differential gene expression analysis between the PBS group and mice at 3 days after LPS treatment. Table S2. Results of differential gene expression analysis at 3 days after LPS and LPS/MSC treatment. Table S3. Results of differential gene expression analysis at 7 days after LPS and LPS/MSC treatment. Table S4. All markers of each cluster. [file 13287_2020_1934_MOESM1_ESM.docx]

**Supplementary methods**

**Immunosuppressive effects of mesenchymal stem cells on lung B cell gene expression in LPS-induced acute lung injury**

Bing Feng^1,2^, Jiaqi Zhu^1,2^, Yanping Xu^1,2^, Wenyi Chen^1,2^, Xinyu Sheng^1,2^, Xudong Feng^1,2^, Xiaowei Shi^1^, Jingqi Liu^1^, Qiaoling Pan^1,2^, Jinfeng Yang^1,2^, Jiong Yu^1,2^, Lanjuan Li^1,2^, Hongcui Cao†^1,2,3^

1 State Key Laboratory for the Diagnosis and Treatment of Infectious Diseases, the First Affiliated Hospital, College of Medicine, Zhejiang University, 79 Qingchun Rd., Hangzhou City 310003, China

2 National Clinical Research Center for Infectious Diseases, 79 Qingchun Rd., Hangzhou City 310003, China

3 Zhejiang Provincial Key Laboratory for Diagnosis and Treatment of Aging and Physic-chemical Injury Diseases, 79 Qingchun Rd, Hangzhou City 310003, China.

**†Corresponding author:**

Hongcui Cao, M.D.

State Key Laboratory for the Diagnosis and Treatment of Infectious Diseases, the First Affiliated Hospital, College of Medicine, Zhejiang University, 79 Qingchun Rd., Hangzhou City 310003, China. Tel: 86-571-87236451; Fax: 86-571-87236459

E-mail: hccao@zju.edu.cn

***Isolation, purification and differentiation of MSCs***

MSCs were isolated and cultured according to Zhu *et al*[1]. Mice aged 2–3 weeks were killed, and humeri, tibiae and femurs were obtained, cut into pieces, and manipulated by 2 mg/mL collagenase II digestion solution (Gibco Life Technologies, Grand Island, NY, USA) for 1.5 h with continuous rotation at 37°C. Tissue pieces were removed for culture in 7.5 mL C57BL/6-MSC special complete MEM (OriCell™ C57BL/6 MSC Complete Medium; Cyagen Biosciences, Guangzhou, China). The nonadherent cells were discarded after 3 days of culture. After 5 days, the cells were washed and harvested with trypsin (0.25%)–EDTA and replated in flasks with complete MEM for further culture. The MSCs in passage 3 were used for experiments. We used the osteoinductive medium (OriCell™ C57BL6 MSC Osteogenic Differentiation Medium; Cyagen Biosciences) and the adipogenic culture medium (OriCell™ C57BL6 MSC Adipogenic Differentiation Medium; Cyagen Biosciences) to induce osteogenic and adipogenic differentiation, respectively. Alizarin Red S (Cyagen Biosciences) staining and Oil red O (Cyagen Biosciences) staining were used to identify results of differentiation.

***Flow cytometry***

We used monoclonal antibodies including anti-APC-CD29 (HMβ1-1, Biolegend®, Enabling Legendary Discovery®; San Diego, CA, USA), anti-APCCy7-CD11b (M1/70; Biolegend), anti-APCCy7-MCHII-1A (M5/114.15.2; Biolegend), anti-PE-CD44 (IM7; Biolegend), anti-PE-SCA-1 (D7; Biolegend), anti-PECy7-CD31 (390; Biolegend), anti-PerCP-CD86 (GL-1; Biolegend), and anti-PECy7-CD45 (30-F11; Biolegend). MSCs were incubated with the above antibodies for 30 min at 4°C in darkness and washed twice with PBS containing 2% fetal bovine serum and 0.1% sodium azide. Flow cytometry was performed and analyzed with FlowJo software (Tree Star, Ashland, OR, USA).

**Supplementary refereneces**

1.Zhu H, Guo ZK, Jiang XX, Li H, Wang XY, Yao HY, et al. A protocol for isolation and culture of mesenchymal stem cells from mouse compact bone. Nat. Protoc. 2010; 5(3): 550-560.

**Supplementary figure and figure legends**


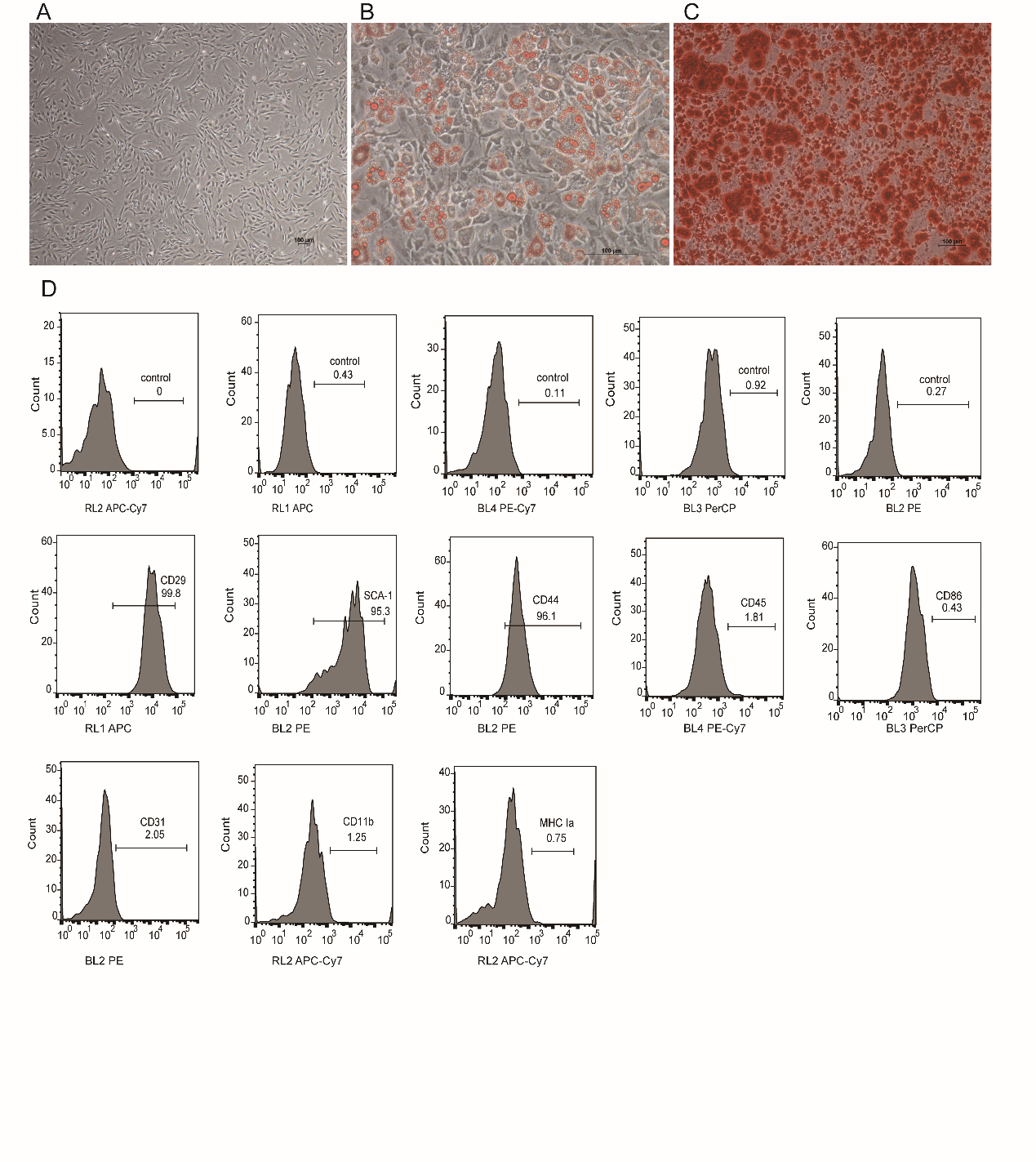
**Figure S1** Characteristics of MSCs. (**A)** MSCs were spindle-shaped in passage 3. MSCs differentiated into adipocytes (**B**) and osteocytes (**C**). (**D)** FACS analysis of MSCs using monoclonal antibodies including CD29, CD44, SCA-1, CD45, CD86, CD31, CD11b and MHC-Ia.


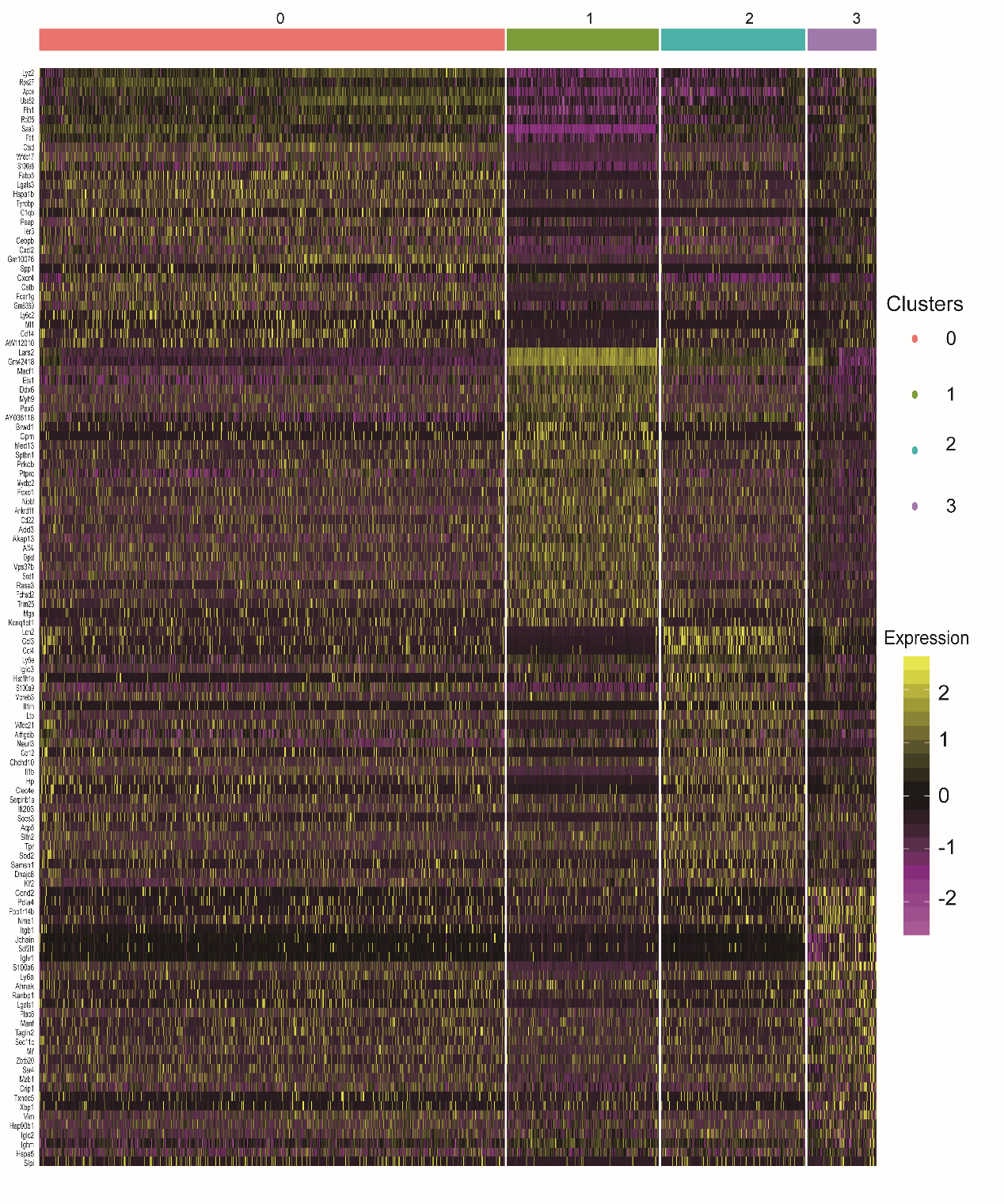


**Figure S2** Heatmap of top 30 marker genes according to foldchange of each cluster identified using cluster specific DEGs.


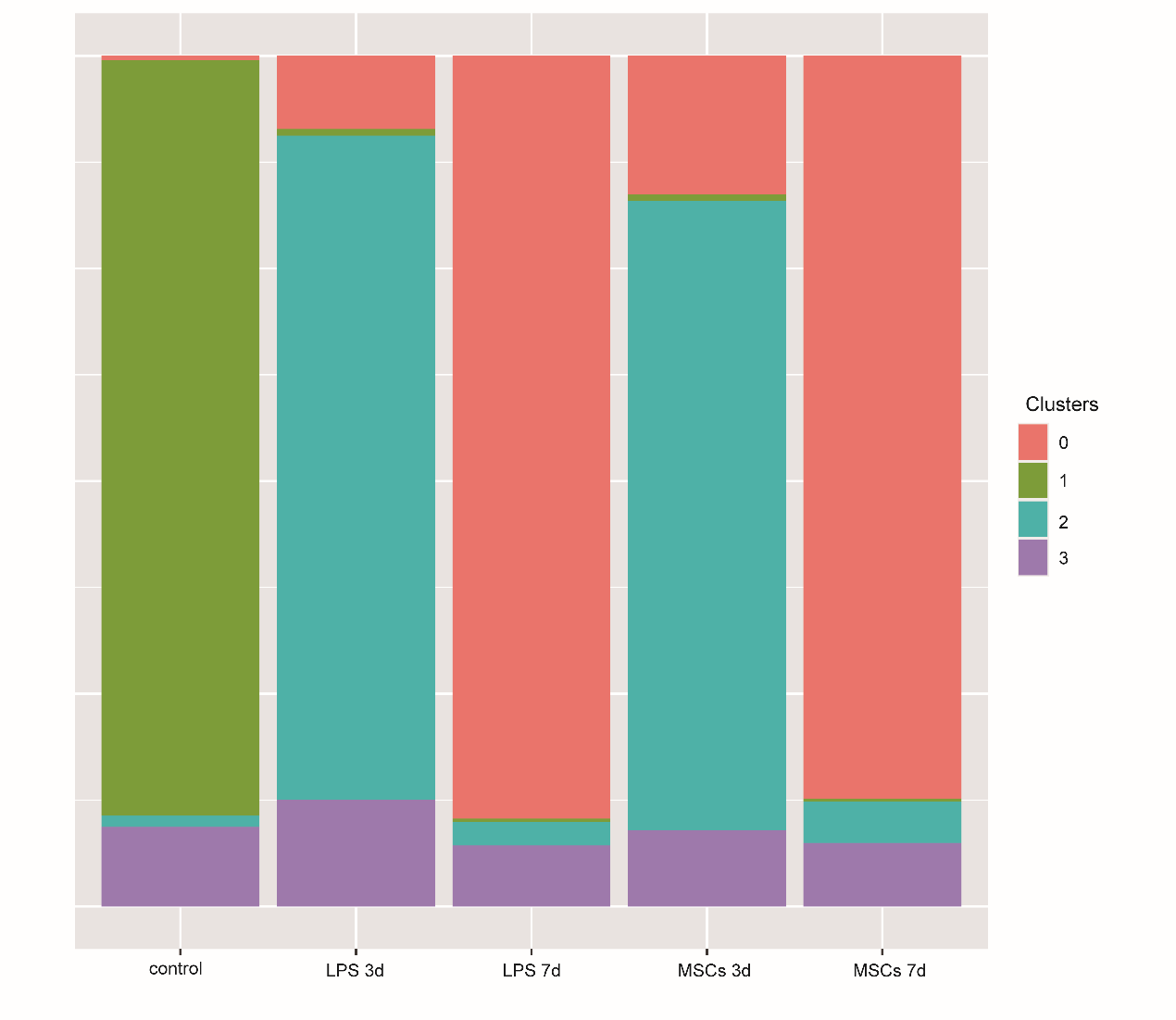


**Figure S3** Proportion of clusters in different groups. The red bars represent cluster 0, the green bars represent cluster 1, the cyan bars represent cluster 2, and the purple bars represent cluster 3.


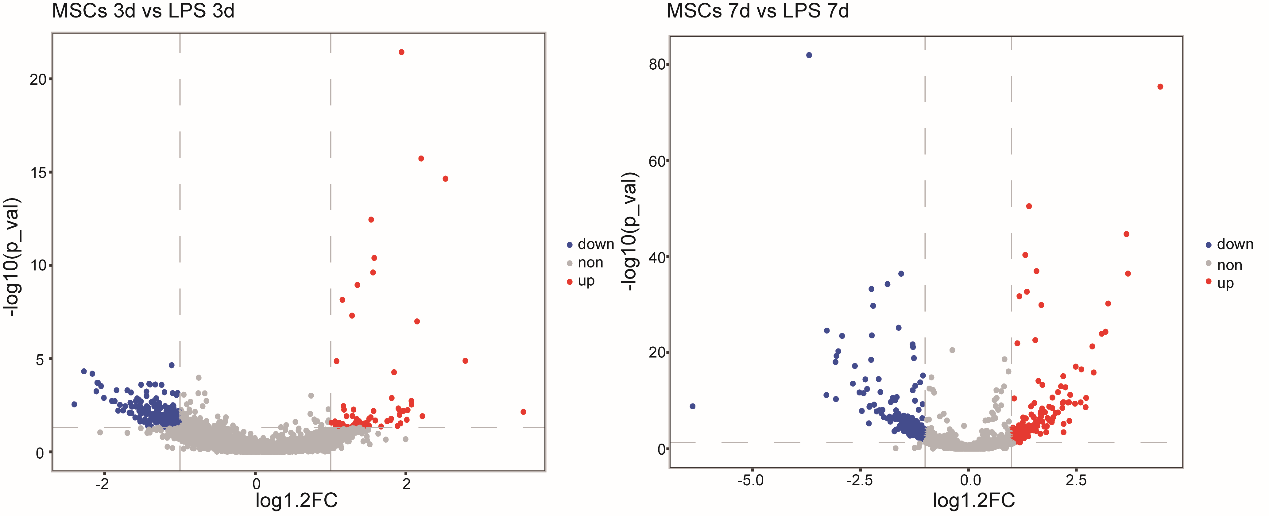


**Figure S4** Volcano plot shows the DEGs of LPS and LPS/MSC groups. *P* values were calculated using Wilcoxon test.


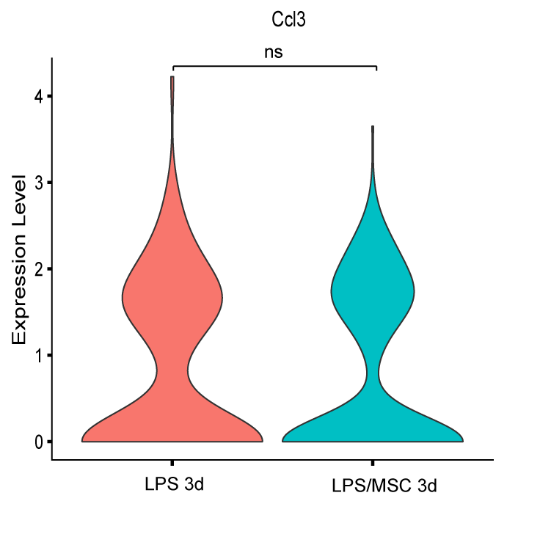


**Figure S5** Violin plots show the log-transformed expression of *Ccl3* at 3 days after LPS and LPS/MSC treatment.


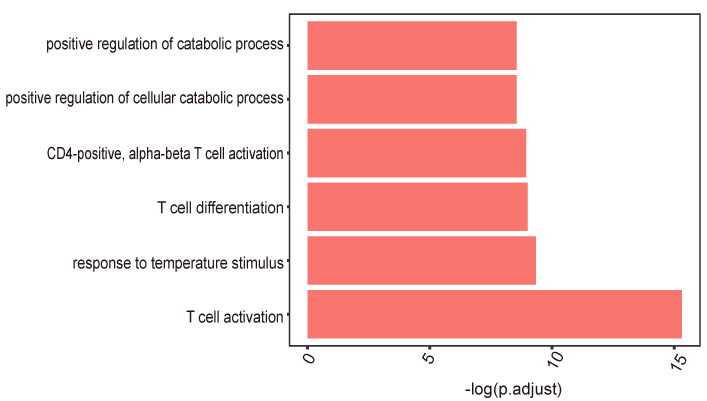


**Figure S6** Selected biological processes predicted by GO functional enrichment analysis using the upregulated genes by MSCs after 7 days.

**Supplementary tables**

**Table S1 Results of differential gene expression analysis between the PBS group and mice at 3 days after LPS treatment**

| gene | p_val | avg_logFC | pct.1 | pct.2 | p_val_adj | Foldchange | log1.2FC |
| --- | --- | --- | --- | --- | --- | --- | --- |
| Saa3 | 1.89E-98 | -2.59187 | 0.029 | 0.961 | 5.87E-94 | 0.07488 | -14.2159 |
| Gm42418 | 7.21E-59 | 1.148338 | 1 | 1 | 2.24E-54 | 3.152949 | 6.298423 |
| Lcn2 | 1.26E-58 | -1.58283 | 0.01 | 0.617 | 3.92E-54 | 0.205393 | -8.68152 |
| Cxcl2 | 8.93E-53 | -2.02081 | 0.171 | 0.805 | 2.77E-48 | 0.132548 | -11.0838 |
| Lars2 | 1.05E-45 | 1.051562 | 0.99 | 0.883 | 3.25E-41 | 2.862119 | 5.767624 |
| S100a8 | 6.99E-45 | -1.58074 | 0.229 | 0.812 | 2.17E-40 | 0.205822 | -8.67009 |
| S100a9 | 2.81E-44 | -1.5164 | 0.283 | 0.844 | 8.74E-40 | 0.219501 | -8.31716 |
| Ccl3 | 4.77E-44 | -1.36843 | 0.008 | 0.477 | 1.48E-39 | 0.254507 | -7.50556 |
| Fth1 | 3.75E-42 | -0.82225 | 0.995 | 1 | 1.16E-37 | 0.439441 | -4.5099 |
| Il1b | 6.15E-39 | -1.42816 | 0.138 | 0.672 | 1.91E-34 | 0.239751 | -7.83317 |
| Wfdc17 | 2.00E-35 | -1.3652 | 0.07 | 0.539 | 6.20E-31 | 0.255329 | -7.48788 |
| Hp | 3.69E-34 | -0.99169 | 0.013 | 0.398 | 1.15E-29 | 0.370948 | -5.43926 |
| Rps19 | 7.37E-34 | -0.44736 | 1 | 1 | 2.29E-29 | 0.639316 | -2.45367 |
| Rpl8 | 3.84E-33 | -0.46879 | 0.997 | 1 | 1.19E-28 | 0.625759 | -2.57123 |
| Ccl4 | 2.24E-31 | -1.26066 | 0.031 | 0.422 | 6.95E-27 | 0.283465 | -6.91451 |
| Tpt1 | 4.70E-31 | -0.37972 | 1 | 1 | 1.46E-26 | 0.684053 | -2.08269 |
| Rps18 | 4.19E-30 | -0.46243 | 0.995 | 1 | 1.30E-25 | 0.629751 | -2.53634 |
| Rpl13 | 2.03E-29 | -0.41305 | 0.995 | 1 | 6.31E-25 | 0.661627 | -2.26552 |
| Rpl21 | 7.71E-29 | -0.43495 | 0.992 | 0.992 | 2.39E-24 | 0.647295 | -2.38563 |
| Rps10 | 2.73E-27 | -0.38411 | 0.997 | 1 | 8.47E-23 | 0.681058 | -2.10676 |
| Rps15a | 7.00E-27 | -0.40383 | 1 | 1 | 2.17E-22 | 0.667756 | -2.21495 |
| Rpl17 | 2.99E-26 | -0.39593 | 1 | 1 | 9.28E-22 | 0.673057 | -2.17158 |
| Rpl9 | 1.24E-25 | -0.3809 | 1 | 1 | 3.85E-21 | 0.683249 | -2.08915 |
| Ftl1 | 3.28E-25 | -0.67597 | 0.961 | 0.969 | 1.02E-20 | 0.508662 | -3.70758 |
| Rps14 | 3.93E-24 | -0.34642 | 0.997 | 1 | 1.22E-19 | 0.707217 | -1.90004 |
| Rps24 | 1.08E-23 | -0.335 | 1 | 1 | 3.36E-19 | 0.715339 | -1.8374 |
| Wfdc21 | 1.61E-23 | -0.95955 | 0.057 | 0.406 | 4.99E-19 | 0.383064 | -5.26297 |
| Rpl27a | 2.80E-23 | -0.34427 | 1 | 1 | 8.69E-19 | 0.70874 | -1.88824 |
| Rpl19 | 2.28E-22 | -0.32838 | 1 | 0.992 | 7.09E-18 | 0.720093 | -1.80108 |
| Rpl12 | 3.34E-21 | -0.39121 | 0.995 | 1 | 1.04E-16 | 0.676235 | -2.14574 |
| Rpl23 | 7.71E-21 | -0.32762 | 0.995 | 1 | 2.39E-16 | 0.720635 | -1.79695 |
| mt-Co1 | 1.52E-20 | 0.319505 | 1 | 1 | 4.72E-16 | 1.376446 | 1.752426 |
| Rpl29 | 1.66E-20 | -0.5472 | 0.958 | 0.977 | 5.15E-16 | 0.578568 | -3.00129 |
| Fcer1g | 2.34E-20 | -0.83198 | 0.073 | 0.398 | 7.26E-16 | 0.435188 | -4.56324 |
| Ccrl2 | 2.90E-20 | -0.83854 | 0.016 | 0.266 | 9.02E-16 | 0.432343 | -4.59921 |
| Rpl18a | 3.29E-20 | -0.33669 | 0.995 | 1 | 1.02E-15 | 0.71413 | -1.84668 |
| Malat1 | 6.56E-20 | 0.354236 | 1 | 1 | 2.04E-15 | 1.425091 | 1.942917 |
| Rpsa | 8.59E-20 | -0.3758 | 1 | 1 | 2.67E-15 | 0.686737 | -2.06121 |
| Rps16 | 2.33E-19 | -0.31017 | 1 | 1 | 7.22E-15 | 0.73332 | -1.70124 |
| Rpl32 | 2.56E-19 | -0.35741 | 0.997 | 1 | 7.96E-15 | 0.699484 | -1.96034 |
| Fau | 3.50E-19 | -0.30411 | 1 | 1 | 1.09E-14 | 0.737777 | -1.66801 |
| Rpl24 | 1.70E-18 | -0.45155 | 0.987 | 0.992 | 5.27E-14 | 0.636638 | -2.47669 |
| Ier3 | 2.04E-18 | -0.68976 | 0.026 | 0.273 | 6.35E-14 | 0.501697 | -3.7832 |
| Rps27a | 7.74E-18 | -0.35616 | 0.997 | 0.992 | 2.40E-13 | 0.70036 | -1.95347 |
| Rpl18 | 8.52E-18 | -0.37945 | 1 | 0.992 | 2.65E-13 | 0.684236 | -2.08122 |
| Rpl26 | 1.29E-17 | -0.35462 | 0.997 | 1 | 4.01E-13 | 0.701437 | -1.94505 |
| Rps20 | 2.18E-17 | -0.33035 | 1 | 1 | 6.78E-13 | 0.71867 | -1.81192 |
| Rps3 | 4.85E-17 | -0.31742 | 0.997 | 1 | 1.51E-12 | 0.728028 | -1.74096 |
| Rps5 | 1.45E-16 | -0.33067 | 1 | 0.992 | 4.49E-12 | 0.718439 | -1.81369 |
| Rpl15 | 1.51E-16 | -0.40434 | 0.995 | 0.984 | 4.67E-12 | 0.667415 | -2.21775 |
| Lyz2 | 1.59E-16 | -0.68458 | 0.486 | 0.742 | 4.93E-12 | 0.504302 | -3.7548 |
| Ddx6 | 1.77E-16 | 0.695428 | 0.873 | 0.492 | 5.50E-12 | 2.004566 | 3.814292 |
| Rps7 | 2.21E-16 | -0.32428 | 1 | 1 | 6.87E-12 | 0.723047 | -1.77862 |
| Rps13 | 2.61E-16 | -0.28211 | 0.995 | 0.992 | 8.11E-12 | 0.754194 | -1.5473 |
| Rps4x | 3.07E-16 | -0.28428 | 1 | 1 | 9.52E-12 | 0.752558 | -1.55921 |
| Il1a | 3.24E-16 | -0.49627 | 0.005 | 0.188 | 1.00E-11 | 0.608797 | -2.72195 |
| AA467197 | 5.50E-15 | -0.52425 | 0.003 | 0.164 | 1.71E-10 | 0.591997 | -2.87544 |
| Fus | 1.57E-14 | 0.635553 | 0.883 | 0.492 | 4.88E-10 | 1.888066 | 3.48589 |
| Myh9 | 2.14E-14 | 0.641928 | 0.795 | 0.359 | 6.64E-10 | 1.90014 | 3.520854 |
| Tmsb4x | 2.43E-14 | -0.32304 | 1 | 1 | 7.54E-10 | 0.723948 | -1.77179 |
| Rps8 | 3.66E-14 | -0.26405 | 1 | 1 | 1.14E-09 | 0.767932 | -1.44829 |
| Ets1 | 3.66E-14 | 0.524481 | 0.956 | 0.703 | 1.14E-09 | 1.689581 | 2.87668 |
| Foxp1 | 2.78E-13 | 0.552682 | 0.979 | 0.695 | 8.64E-09 | 1.737909 | 3.031361 |
| Iqgap1 | 4.83E-13 | 0.547493 | 0.904 | 0.531 | 1.50E-08 | 1.728913 | 3.002899 |
| Rpl38 | 6.04E-13 | 0.274286 | 1 | 0.992 | 1.88E-08 | 1.315591 | 1.504409 |
| Ighm | 7.52E-13 | 0.454013 | 0.995 | 0.969 | 2.34E-08 | 1.574618 | 2.490175 |
| Prdx5 | 7.99E-13 | -0.75349 | 0.309 | 0.555 | 2.48E-08 | 0.470722 | -4.13275 |
| Cd14 | 1.65E-12 | -0.8181 | 0.052 | 0.266 | 5.12E-08 | 0.441269 | -4.48714 |
| Rps3a1 | 2.34E-12 | -0.2273 | 0.997 | 1 | 7.28E-08 | 0.79668 | -1.24671 |
| Rps6 | 3.67E-12 | -0.2831 | 0.99 | 0.984 | 1.14E-07 | 0.753446 | -1.55274 |
| Syk | 4.06E-12 | 0.563186 | 0.842 | 0.484 | 1.26E-07 | 1.756258 | 3.088969 |
| Rps11 | 5.51E-12 | -0.31598 | 1 | 0.992 | 1.71E-07 | 0.729073 | -1.7331 |
| Gm5483 | 6.21E-12 | -0.45847 | 0.005 | 0.141 | 1.93E-07 | 0.632253 | -2.5146 |
| Rplp0 | 7.34E-12 | -0.34735 | 0.987 | 1 | 2.28E-07 | 0.70656 | -1.90513 |
| Dgkd | 9.40E-12 | 0.591985 | 0.621 | 0.219 | 2.92E-07 | 1.807574 | 3.24693 |
| Kdm6b | 9.67E-12 | 0.686481 | 0.655 | 0.273 | 3.00E-07 | 1.986712 | 3.765221 |
| BC005537 | 1.20E-11 | 0.572459 | 0.655 | 0.258 | 3.72E-07 | 1.77262 | 3.139831 |
| Macf1 | 1.64E-11 | 0.554216 | 0.899 | 0.641 | 5.09E-07 | 1.740575 | 3.039771 |
| Rpl11 | 2.32E-11 | -0.29929 | 0.995 | 1 | 7.22E-07 | 0.741346 | -1.64154 |
| Rpl10a | 3.23E-11 | -0.31806 | 0.992 | 0.984 | 1.00E-06 | 0.72756 | -1.74449 |
| Rpl7 | 4.38E-11 | -0.26377 | 0.997 | 1 | 1.36E-06 | 0.768147 | -1.44675 |
| Prkcb | 4.84E-11 | 0.552857 | 0.66 | 0.273 | 1.50E-06 | 1.738212 | 3.032318 |
| Pax5 | 4.96E-11 | 0.531006 | 0.813 | 0.406 | 1.54E-06 | 1.700643 | 2.912472 |
| Socs3 | 6.39E-11 | -0.63896 | 0.078 | 0.297 | 1.99E-06 | 0.527842 | -3.50457 |
| Ddx17 | 7.06E-11 | 0.484801 | 0.522 | 0.148 | 2.19E-06 | 1.623852 | 2.659043 |
| Cxcl10 | 1.01E-10 | -0.45042 | 0.003 | 0.117 | 3.14E-06 | 0.637364 | -2.47044 |
| Scd1 | 1.79E-10 | 0.563311 | 0.821 | 0.516 | 5.57E-06 | 1.756479 | 3.089659 |
| Il1rn | 1.94E-10 | -0.46677 | 0.008 | 0.133 | 6.04E-06 | 0.627026 | -2.56014 |
| Cerk | 2.27E-10 | 0.575988 | 0.509 | 0.156 | 7.06E-06 | 1.778887 | 3.159187 |
| Fli1 | 2.48E-10 | 0.532957 | 0.496 | 0.148 | 7.71E-06 | 1.703963 | 2.92317 |
| Rbm3 | 2.65E-10 | -0.55909 | 0.605 | 0.727 | 8.22E-06 | 0.571731 | -3.06649 |
| Itpr3 | 3.93E-10 | 0.580577 | 0.364 | 0.07 | 1.22E-05 | 1.78707 | 3.184359 |
| Aff4 | 5.55E-10 | 0.52573 | 0.629 | 0.266 | 1.72E-05 | 1.691693 | 2.883529 |
| Ifitm2 | 6.49E-10 | -0.55605 | 0.034 | 0.195 | 2.02E-05 | 0.573469 | -3.04983 |
| Chchd2 | 6.56E-10 | -0.47321 | 0.8 | 0.82 | 2.04E-05 | 0.622999 | -2.59547 |
| mt-Nd3 | 6.73E-10 | 0.390854 | 0.969 | 0.758 | 2.09E-05 | 1.478243 | 2.143764 |
| Itga4 | 9.05E-10 | 0.468661 | 0.727 | 0.367 | 2.81E-05 | 1.597853 | 2.570516 |
| Pgap1 | 9.72E-10 | 0.552697 | 0.587 | 0.242 | 3.02E-05 | 1.737934 | 3.031442 |
| Cd55 | 1.00E-09 | 0.474255 | 0.782 | 0.43 | 3.11E-05 | 1.606817 | 2.601203 |
| Serinc3 | 1.09E-09 | 0.350023 | 0.966 | 0.766 | 3.39E-05 | 1.4191 | 1.919812 |
| Rpl3 | 1.23E-09 | -0.29129 | 0.995 | 0.992 | 3.81E-05 | 0.747299 | -1.59767 |
| March7 | 1.31E-09 | 0.462718 | 0.553 | 0.195 | 4.06E-05 | 1.588385 | 2.537921 |
| Sptbn1 | 1.49E-09 | 0.515276 | 0.603 | 0.234 | 4.61E-05 | 1.6741 | 2.826193 |
| Rpl37a | 1.80E-09 | 0.208355 | 1 | 0.992 | 5.57E-05 | 1.23165 | 1.142787 |
| Cnot1 | 2.00E-09 | 0.488332 | 0.421 | 0.109 | 6.20E-05 | 1.629596 | 2.678413 |
| Smg1 | 2.55E-09 | 0.516614 | 0.636 | 0.297 | 7.93E-05 | 1.676342 | 2.833533 |
| Ahnak | 2.63E-09 | 0.863746 | 0.371 | 0.094 | 8.16E-05 | 2.372029 | 4.737485 |
| Ripor2 | 2.77E-09 | 0.456601 | 0.73 | 0.367 | 8.62E-05 | 1.578698 | 2.50437 |
| Rps9 | 3.02E-09 | -0.21866 | 1 | 1 | 9.37E-05 | 0.803597 | -1.1993 |
| Kmt2e | 3.21E-09 | 0.448114 | 0.836 | 0.523 | 9.98E-05 | 1.565358 | 2.457825 |
| Rps23 | 5.62E-09 | -0.22787 | 0.997 | 0.992 | 0.000174 | 0.796226 | -1.24984 |
| Ash1l | 6.17E-09 | 0.41074 | 0.647 | 0.289 | 0.000192 | 1.507933 | 2.252833 |
| Mau2 | 6.36E-09 | 0.508771 | 0.382 | 0.102 | 0.000197 | 1.663246 | 2.790514 |
| Brd2 | 6.51E-09 | 0.460318 | 0.668 | 0.328 | 0.000202 | 1.584578 | 2.524759 |
| Rpl6 | 7.11E-09 | -0.25135 | 0.995 | 0.992 | 0.000221 | 0.777752 | -1.3786 |
| Rpl28 | 1.04E-08 | -0.26167 | 0.997 | 0.992 | 0.000322 | 0.769769 | -1.43518 |
| Add3 | 1.06E-08 | 0.488916 | 0.631 | 0.297 | 0.000328 | 1.630549 | 2.681616 |
| Notch2 | 1.07E-08 | 0.427306 | 0.294 | 0.039 | 0.000333 | 1.533122 | 2.343695 |
| Dnaja1 | 1.12E-08 | -0.59992 | 0.722 | 0.773 | 0.000348 | 0.548857 | -3.29043 |
| mt-Nd4 | 1.31E-08 | 0.237158 | 1 | 0.992 | 0.000408 | 1.267642 | 1.30077 |
| Rsrp1 | 1.45E-08 | -0.58262 | 0.725 | 0.781 | 0.00045 | 0.558435 | -3.19554 |
| Cpm | 1.48E-08 | 0.596618 | 0.291 | 0.047 | 0.000458 | 1.815966 | 3.272337 |
| Akap13 | 1.53E-08 | 0.429965 | 0.86 | 0.562 | 0.000475 | 1.537204 | 2.358278 |
| Sfpq | 2.14E-08 | 0.431152 | 0.813 | 0.453 | 0.000664 | 1.53903 | 2.364789 |
| Stt3b | 2.18E-08 | 0.434866 | 0.408 | 0.117 | 0.000676 | 1.544756 | 2.385159 |
| Hcar2 | 2.18E-08 | -0.38285 | 0.008 | 0.109 | 0.000678 | 0.681917 | -2.09984 |
| Cstb | 2.25E-08 | -0.65154 | 0.169 | 0.367 | 0.000699 | 0.521244 | -3.57356 |
| Pfn1 | 2.44E-08 | -0.35731 | 0.888 | 0.883 | 0.000756 | 0.699556 | -1.95977 |
| Foxo1 | 2.72E-08 | 0.482826 | 0.579 | 0.258 | 0.000845 | 1.620648 | 2.648213 |
| Pde3b | 2.86E-08 | 0.46366 | 0.403 | 0.117 | 0.000888 | 1.589882 | 2.543087 |
| Ptprc | 2.88E-08 | 0.378324 | 0.943 | 0.68 | 0.000894 | 1.459836 | 2.075037 |
| Rasa3 | 3.14E-08 | 0.552955 | 0.481 | 0.188 | 0.000974 | 1.738383 | 3.032858 |
| Ago2 | 3.36E-08 | 0.410651 | 0.465 | 0.148 | 0.001043 | 1.507799 | 2.252345 |
| Med13 | 3.62E-08 | 0.407006 | 0.623 | 0.281 | 0.001123 | 1.502314 | 2.232354 |
| Clec4e | 3.77E-08 | -0.48687 | 0.023 | 0.148 | 0.001169 | 0.614549 | -2.67038 |
| Rpl27 | 3.85E-08 | -0.29952 | 0.987 | 0.984 | 0.001194 | 0.741171 | -1.64283 |
| Cfap43 | 4.19E-08 | 0.476079 | 0.301 | 0.055 | 0.001302 | 1.60975 | 2.611204 |
| Msl2 | 4.52E-08 | 0.45665 | 0.299 | 0.055 | 0.001404 | 1.578776 | 2.50464 |
| Mtss1 | 5.20E-08 | 0.406404 | 0.574 | 0.258 | 0.001614 | 1.501408 | 2.229049 |
| Rps2 | 5.71E-08 | -0.26832 | 0.997 | 1 | 0.001774 | 0.764662 | -1.47169 |
| Lrrk2 | 6.41E-08 | 0.521102 | 0.455 | 0.18 | 0.00199 | 1.683882 | 2.858146 |
| Crebbp | 6.82E-08 | 0.476639 | 0.543 | 0.227 | 0.002117 | 1.610651 | 2.614275 |
| Ifitm1 | 7.04E-08 | -0.44925 | 0.018 | 0.133 | 0.002186 | 0.638106 | -2.46406 |
| Txn1 | 7.12E-08 | -0.59776 | 0.34 | 0.508 | 0.002212 | 0.550041 | -3.27862 |
| Rplp2 | 7.14E-08 | -0.19429 | 1 | 1 | 0.002217 | 0.823422 | -1.06562 |
| Usp24 | 7.40E-08 | 0.453171 | 0.33 | 0.078 | 0.002297 | 1.573293 | 2.485559 |
| Pde7a | 1.03E-07 | 0.447865 | 0.449 | 0.164 | 0.003184 | 1.564967 | 2.456457 |
| Nlrp3 | 1.03E-07 | -0.34485 | 0.013 | 0.117 | 0.003204 | 0.708329 | -1.89142 |
| Mob1a | 1.08E-07 | 0.410444 | 0.439 | 0.148 | 0.003354 | 1.507487 | 2.251209 |
| Fcer2a | 1.16E-07 | 0.450313 | 0.553 | 0.234 | 0.0036 | 1.568804 | 2.469886 |
| Rpl35 | 1.23E-07 | 0.263665 | 0.99 | 0.969 | 0.003808 | 1.301692 | 1.446155 |
| Tmem245 | 1.35E-07 | 0.447969 | 0.294 | 0.055 | 0.004191 | 1.56513 | 2.457027 |
| Ankrd11 | 1.49E-07 | 0.428575 | 0.816 | 0.516 | 0.004623 | 1.535068 | 2.350652 |
| Baz2b | 1.50E-07 | 0.418867 | 0.6 | 0.281 | 0.004672 | 1.520238 | 2.297407 |
| Gm8369 | 1.95E-07 | -0.64901 | 0.532 | 0.68 | 0.006061 | 0.522561 | -3.55972 |
| Dock8 | 1.96E-07 | 0.414218 | 0.418 | 0.148 | 0.006078 | 1.513187 | 2.271911 |
| Prkcd | 2.07E-07 | 0.501501 | 0.442 | 0.172 | 0.006418 | 1.651197 | 2.750637 |
| Cyth1 | 2.30E-07 | 0.437162 | 0.545 | 0.242 | 0.007153 | 1.548307 | 2.397754 |
| Gbp2 | 2.37E-07 | -0.57007 | 0.044 | 0.18 | 0.007367 | 0.565486 | -3.12672 |
| Bach2 | 2.40E-07 | 0.503798 | 0.548 | 0.242 | 0.007463 | 1.654996 | 2.763241 |
| Srgn | 2.66E-07 | -0.36257 | 0.958 | 0.922 | 0.008262 | 0.695887 | -1.98862 |
| Rassf3 | 2.80E-07 | 0.484252 | 0.361 | 0.117 | 0.008681 | 1.62296 | 2.656031 |
| Cacybp | 3.18E-07 | -0.5422 | 0.187 | 0.367 | 0.009866 | 0.581468 | -2.97386 |
| Tgfbi | 3.34E-07 | -0.31535 | 0.018 | 0.125 | 0.010364 | 0.729531 | -1.72965 |
| Gm10076 | 3.40E-07 | 0.426047 | 0.369 | 0.117 | 0.010549 | 1.531193 | 2.336791 |
| Blk | 3.40E-07 | 0.471561 | 0.413 | 0.148 | 0.010568 | 1.602494 | 2.586426 |
| Fbxo33 | 3.57E-07 | 0.385877 | 0.226 | 0.023 | 0.011082 | 1.470904 | 2.116465 |
| Ubb | 3.90E-07 | -0.2622 | 0.987 | 0.992 | 0.012112 | 0.769357 | -1.43812 |
| Plekha2 | 3.92E-07 | 0.381778 | 0.517 | 0.203 | 0.012166 | 1.464887 | 2.093983 |
| Arhgef18 | 4.34E-07 | 0.391518 | 0.384 | 0.117 | 0.013482 | 1.479225 | 2.147404 |
| Sik1 | 4.41E-07 | 0.405975 | 0.561 | 0.258 | 0.013704 | 1.500765 | 2.226698 |
| Rps27rt | 4.46E-07 | -0.38415 | 0.857 | 0.906 | 0.013834 | 0.681028 | -2.107 |
| Ccdc88c | 4.54E-07 | 0.408842 | 0.416 | 0.148 | 0.014101 | 1.505075 | 2.242425 |
| B3gnt5 | 4.72E-07 | 0.459725 | 0.34 | 0.102 | 0.014659 | 1.583638 | 2.521506 |
| Etnk1 | 5.45E-07 | 0.420334 | 0.6 | 0.297 | 0.01691 | 1.52247 | 2.305453 |
| Uhrf1bp1l | 5.50E-07 | 0.394903 | 0.234 | 0.031 | 0.017091 | 1.484241 | 2.165972 |
| Chd4 | 5.50E-07 | 0.461677 | 0.657 | 0.367 | 0.017093 | 1.586732 | 2.532211 |
| Sbk1 | 5.69E-07 | 0.393962 | 0.312 | 0.078 | 0.017655 | 1.482845 | 2.16081 |
| Dennd4a | 5.83E-07 | 0.388369 | 0.821 | 0.539 | 0.018106 | 1.474573 | 2.13013 |
| Sod2 | 5.88E-07 | -0.7123 | 0.197 | 0.367 | 0.018245 | 0.490513 | -3.90685 |
| Flii | 6.04E-07 | 0.40304 | 0.268 | 0.055 | 0.018771 | 1.496367 | 2.210599 |
| Brwd1 | 6.76E-07 | 0.43514 | 0.392 | 0.133 | 0.020999 | 1.545179 | 2.38666 |
| Fam208b | 6.82E-07 | 0.383015 | 0.322 | 0.086 | 0.021166 | 1.4667 | 2.100767 |
| Dennd1b | 7.56E-07 | 0.404099 | 0.408 | 0.141 | 0.023463 | 1.497952 | 2.216407 |
| Herc1 | 7.60E-07 | 0.406469 | 0.387 | 0.133 | 0.023603 | 1.501506 | 2.229406 |
| P2ry10 | 7.73E-07 | 0.382888 | 0.797 | 0.469 | 0.023998 | 1.466514 | 2.10007 |
| Nfkbia | 8.39E-07 | -0.40345 | 0.712 | 0.781 | 0.026043 | 0.668014 | -2.21283 |
| Srebf2 | 9.07E-07 | 0.37662 | 0.304 | 0.078 | 0.028165 | 1.457351 | 2.065692 |
| AW112010 | 9.25E-07 | -0.49863 | 0.101 | 0.258 | 0.028718 | 0.607361 | -2.7349 |
| Rfx7 | 9.36E-07 | 0.396984 | 0.4 | 0.148 | 0.029069 | 1.487331 | 2.177381 |
| Canx | 9.47E-07 | 0.415486 | 0.483 | 0.203 | 0.029415 | 1.515107 | 2.278864 |
| Wipi2 | 9.77E-07 | 0.394114 | 0.278 | 0.062 | 0.030338 | 1.48307 | 2.161642 |
| Cbl | 1.01E-06 | 0.338067 | 0.439 | 0.172 | 0.031223 | 1.402235 | 1.854236 |
| Tob2 | 1.04E-06 | 0.405824 | 0.584 | 0.305 | 0.032316 | 1.500538 | 2.225869 |
| Arhgef1 | 1.07E-06 | 0.437199 | 0.623 | 0.336 | 0.033364 | 1.548365 | 2.397958 |
| Rpl30 | 1.08E-06 | -0.1833 | 1 | 1 | 0.033464 | 0.832516 | -1.00539 |
| Adgre5 | 1.10E-06 | 0.503249 | 0.47 | 0.211 | 0.034007 | 1.654086 | 2.760227 |
| Kcnq1ot1 | 1.12E-06 | 0.625283 | 0.423 | 0.172 | 0.034849 | 1.868774 | 3.429561 |
| Zc3hav1 | 1.13E-06 | 0.299936 | 0.449 | 0.18 | 0.034954 | 1.349773 | 1.645096 |
| Lbh | 1.19E-06 | 0.356144 | 0.496 | 0.211 | 0.037053 | 1.427813 | 1.953382 |
| Map4k2 | 1.21E-06 | 0.305511 | 0.34 | 0.102 | 0.037481 | 1.357318 | 1.67567 |
| Mknk2 | 1.26E-06 | 0.380189 | 0.452 | 0.18 | 0.039063 | 1.462561 | 2.085268 |
| Cd22 | 1.30E-06 | 0.359776 | 0.608 | 0.312 | 0.040375 | 1.433008 | 1.973304 |
| Trp53inp1 | 1.30E-06 | 0.449008 | 0.317 | 0.094 | 0.040435 | 1.566758 | 2.462728 |
| Bmp2k | 1.43E-06 | 0.24408 | 0.462 | 0.172 | 0.044385 | 1.276446 | 1.338734 |
| Wdr26 | 1.51E-06 | 0.372904 | 0.442 | 0.18 | 0.046751 | 1.451945 | 2.045309 |
| Hmgb2 | 1.76E-06 | 0.338483 | 0.686 | 0.383 | 0.054687 | 1.402818 | 1.856517 |
| Naca | 1.76E-06 | -0.31022 | 0.943 | 0.906 | 0.054752 | 0.733285 | -1.70151 |
| Cd2 | 1.77E-06 | 0.440067 | 0.525 | 0.242 | 0.054962 | 1.552811 | 2.413685 |
| Vps37b | 1.80E-06 | 0.459445 | 0.725 | 0.445 | 0.055996 | 1.583195 | 2.519971 |
| Clec4d | 1.98E-06 | -0.4116 | 0.013 | 0.102 | 0.06141 | 0.662588 | -2.25756 |
| Cd19 | 2.01E-06 | 0.402247 | 0.673 | 0.367 | 0.06252 | 1.495181 | 2.20625 |
| Ptgs2 | 2.04E-06 | -0.37744 | 0.013 | 0.102 | 0.06341 | 0.685613 | -2.0702 |
| Prpf39 | 2.09E-06 | 0.379545 | 0.343 | 0.109 | 0.065047 | 1.46162 | 2.081735 |
| Fpr2 | 2.13E-06 | -0.31859 | 0.013 | 0.102 | 0.066174 | 0.727175 | -1.7474 |
| Hip1r | 2.29E-06 | 0.313524 | 0.384 | 0.133 | 0.070987 | 1.368238 | 1.71962 |
| Rabgap1l | 2.45E-06 | 0.423024 | 0.626 | 0.344 | 0.076041 | 1.526572 | 2.32021 |
| Rb1cc1 | 2.45E-06 | 0.435726 | 0.509 | 0.227 | 0.076116 | 1.546085 | 2.389876 |
| Marf1 | 2.54E-06 | 0.30593 | 0.218 | 0.031 | 0.078931 | 1.357887 | 1.67797 |
| Chst3 | 2.68E-06 | 0.402647 | 0.281 | 0.07 | 0.083236 | 1.495779 | 2.208444 |
| Ivns1abp | 2.72E-06 | 0.262432 | 0.429 | 0.164 | 0.08453 | 1.300089 | 1.439393 |
| Ddx3x | 2.74E-06 | 0.389004 | 0.696 | 0.414 | 0.085113 | 1.475511 | 2.133616 |
| Yme1l1 | 2.75E-06 | 0.237417 | 0.374 | 0.117 | 0.0853 | 1.267969 | 1.302186 |
| Ccl6 | 2.76E-06 | 0.376557 | 0.223 | 0.039 | 0.08571 | 1.457259 | 2.065347 |
| Larp4b | 2.81E-06 | 0.359907 | 0.335 | 0.109 | 0.087247 | 1.433197 | 1.974026 |
| Nckap1l | 2.93E-06 | 0.33195 | 0.345 | 0.117 | 0.090878 | 1.393683 | 1.820684 |
| Larp1 | 3.05E-06 | 0.341135 | 0.338 | 0.109 | 0.094573 | 1.406544 | 1.871064 |
| Zfp36l1 | 3.15E-06 | 0.319384 | 0.912 | 0.703 | 0.097701 | 1.376279 | 1.75176 |
| Ankrd44 | 3.21E-06 | 0.322301 | 0.595 | 0.297 | 0.099662 | 1.3803 | 1.767761 |
| Fam107b | 3.25E-06 | 0.352752 | 0.888 | 0.609 | 0.100919 | 1.422979 | 1.934782 |
| Gpr132 | 3.25E-06 | 0.422507 | 0.444 | 0.195 | 0.101036 | 1.525781 | 2.317371 |
| Icam1 | 3.53E-06 | -0.49868 | 0.122 | 0.281 | 0.109749 | 0.607332 | -2.73517 |
| Baz2a | 3.64E-06 | 0.398291 | 0.338 | 0.117 | 0.113012 | 1.489277 | 2.18455 |
| Myadm | 3.64E-06 | 0.387856 | 0.19 | 0.023 | 0.113102 | 1.473817 | 2.127316 |
| Fam117b | 3.72E-06 | 0.423411 | 0.325 | 0.109 | 0.115534 | 1.527163 | 2.322334 |
| Atp2a3 | 3.84E-06 | 0.393421 | 0.335 | 0.117 | 0.119192 | 1.482043 | 2.157843 |
| Eif1 | 3.88E-06 | -0.23351 | 0.997 | 0.984 | 0.120454 | 0.791753 | -1.28074 |
| Nfatc3 | 4.14E-06 | 0.317168 | 0.621 | 0.336 | 0.128406 | 1.373233 | 1.739607 |
| Ncoa3 | 4.21E-06 | 0.356771 | 0.348 | 0.117 | 0.130713 | 1.428709 | 1.956824 |
| Usp7 | 4.37E-06 | 0.405095 | 0.418 | 0.172 | 0.135574 | 1.499445 | 2.221872 |
| Myo1g | 4.53E-06 | 0.387012 | 0.314 | 0.102 | 0.140644 | 1.472574 | 2.12269 |
| Sf1 | 4.54E-06 | 0.331182 | 0.483 | 0.211 | 0.141068 | 1.392614 | 1.816474 |
| Pecam1 | 4.85E-06 | 0.343259 | 0.439 | 0.188 | 0.150559 | 1.409534 | 1.882714 |
| Tcf12 | 5.14E-06 | 0.347561 | 0.431 | 0.18 | 0.15951 | 1.415611 | 1.906309 |
| Gpcpd1 | 5.16E-06 | 0.338165 | 0.47 | 0.211 | 0.160267 | 1.402372 | 1.854772 |
| Nipbl | 5.34E-06 | 0.366622 | 0.66 | 0.391 | 0.165818 | 1.442852 | 2.010852 |
| Med15 | 5.35E-06 | 0.312911 | 0.19 | 0.023 | 0.166095 | 1.3674 | 1.716262 |
| Arid1a | 5.60E-06 | 0.387055 | 0.478 | 0.219 | 0.173918 | 1.472637 | 2.122925 |
| Hnrnph1 | 5.89E-06 | 0.42841 | 0.665 | 0.391 | 0.182931 | 1.534815 | 2.34975 |
| Mbnl1 | 6.09E-06 | 0.323696 | 0.834 | 0.586 | 0.189161 | 1.382227 | 1.775412 |
| N4bp2 | 6.19E-06 | 0.399835 | 0.371 | 0.141 | 0.192097 | 1.491579 | 2.193024 |
| Birc6 | 6.27E-06 | 0.342187 | 0.587 | 0.281 | 0.194639 | 1.408024 | 1.876834 |
| Ifitm3 | 6.54E-06 | -0.32185 | 0.034 | 0.141 | 0.202939 | 0.724804 | -1.76531 |
| Ddb1 | 7.00E-06 | 0.292586 | 0.221 | 0.039 | 0.217503 | 1.339888 | 1.604781 |
| Stim1 | 7.08E-06 | 0.364835 | 0.257 | 0.07 | 0.219907 | 1.440276 | 2.00105 |
| Kif21b | 7.26E-06 | 0.361313 | 0.34 | 0.117 | 0.225352 | 1.435213 | 1.981737 |
| Srsf2 | 7.46E-06 | 0.310443 | 0.655 | 0.359 | 0.231558 | 1.36403 | 1.702724 |
| Plcg2 | 8.01E-06 | 0.269326 | 0.395 | 0.141 | 0.248846 | 1.309082 | 1.477203 |
| Rps25 | 8.19E-06 | -0.20175 | 1 | 1 | 0.254339 | 0.817301 | -1.10655 |
| Bptf | 8.25E-06 | 0.303709 | 0.722 | 0.398 | 0.256217 | 1.354875 | 1.66579 |
| Arap1 | 8.33E-06 | 0.325643 | 0.273 | 0.07 | 0.258591 | 1.384921 | 1.786092 |
| Atp5g2 | 8.39E-06 | -0.3535 | 0.719 | 0.742 | 0.260498 | 0.702224 | -1.9389 |
| Ankhd1 | 8.45E-06 | 0.368427 | 0.327 | 0.109 | 0.262541 | 1.445459 | 2.020755 |
| Arfgef1 | 8.67E-06 | 0.379436 | 0.475 | 0.219 | 0.269225 | 1.46146 | 2.081136 |
| Susd6 | 8.95E-06 | 0.310689 | 0.387 | 0.156 | 0.277958 | 1.364365 | 1.704071 |
| Nfat5 | 9.07E-06 | 0.321178 | 0.481 | 0.227 | 0.281786 | 1.378751 | 1.761601 |
| Dnajc5 | 9.08E-06 | 0.326866 | 0.343 | 0.125 | 0.28198 | 1.386616 | 1.792802 |
| Mcl1 | 9.11E-06 | -0.3816 | 0.831 | 0.828 | 0.28294 | 0.682766 | -2.09302 |
| Appbp2 | 9.15E-06 | 0.244343 | 0.239 | 0.055 | 0.28405 | 1.276783 | 1.340178 |
| Arhgef3 | 9.24E-06 | 0.375552 | 0.257 | 0.07 | 0.286923 | 1.455795 | 2.059835 |
| Fam168b | 9.78E-06 | 0.297717 | 0.384 | 0.148 | 0.303636 | 1.34678 | 1.632921 |
| Gigyf1 | 9.85E-06 | 0.322421 | 0.278 | 0.078 | 0.305923 | 1.380465 | 1.768418 |
| Fmnl1 | 1.03E-05 | 0.311928 | 0.322 | 0.109 | 0.318591 | 1.366056 | 1.710865 |
| Cdc73 | 1.04E-05 | 0.332139 | 0.223 | 0.047 | 0.323461 | 1.393946 | 1.82172 |
| Rubcn | 1.05E-05 | 0.33104 | 0.239 | 0.055 | 0.325582 | 1.392415 | 1.815691 |
| Azin1 | 1.08E-05 | 0.350448 | 0.299 | 0.094 | 0.334372 | 1.419703 | 1.922141 |
| Dhx15 | 1.11E-05 | 0.338432 | 0.387 | 0.148 | 0.345253 | 1.402746 | 1.856235 |
| Cyfip2 | 1.14E-05 | 0.285852 | 0.439 | 0.188 | 0.353725 | 1.330895 | 1.567843 |
| Tle3 | 1.18E-05 | 0.350366 | 0.192 | 0.031 | 0.364975 | 1.419587 | 1.921693 |
| Wdr89 | 1.22E-05 | -0.43959 | 0.616 | 0.648 | 0.378003 | 0.644303 | -2.41105 |
| Slpi | 1.31E-05 | -0.43926 | 0.049 | 0.164 | 0.408162 | 0.644516 | -2.40924 |
| Pan3 | 1.33E-05 | 0.371718 | 0.403 | 0.172 | 0.413544 | 1.450224 | 2.038806 |
| Neat1 | 1.41E-05 | 0.207034 | 0.522 | 0.258 | 0.438789 | 1.230024 | 1.135541 |
| Zfp831 | 1.45E-05 | 0.273705 | 0.197 | 0.031 | 0.449737 | 1.314826 | 1.501219 |
| Rc3h1 | 1.45E-05 | 0.283003 | 0.291 | 0.086 | 0.451188 | 1.327109 | 1.552219 |
| Nedd9 | 1.48E-05 | 0.373814 | 0.405 | 0.172 | 0.459951 | 1.453266 | 2.050299 |
| Ptp4a2 | 1.53E-05 | 0.292226 | 0.782 | 0.484 | 0.474585 | 1.339406 | 1.602806 |
| Pdzd8 | 1.54E-05 | 0.324487 | 0.236 | 0.055 | 0.478129 | 1.383321 | 1.77975 |
| Txndc16 | 1.63E-05 | 0.322845 | 0.465 | 0.219 | 0.505711 | 1.381052 | 1.770747 |
| Taok1 | 1.67E-05 | 0.294906 | 0.348 | 0.125 | 0.519399 | 1.343 | 1.617505 |
| Helz2 | 1.69E-05 | 0.322007 | 0.39 | 0.148 | 0.523919 | 1.379894 | 1.766147 |
| Nsf | 1.70E-05 | 0.419039 | 0.374 | 0.148 | 0.528092 | 1.5205 | 2.298352 |
| Cbx4 | 1.71E-05 | 0.344707 | 0.231 | 0.055 | 0.530338 | 1.411576 | 1.890652 |
| Tcf3 | 1.72E-05 | 0.318238 | 0.288 | 0.086 | 0.534821 | 1.374703 | 1.745476 |
| Srsf6 | 1.80E-05 | 0.346249 | 0.449 | 0.211 | 0.558642 | 1.413754 | 1.89911 |
| Dock2 | 1.85E-05 | 0.301109 | 0.701 | 0.406 | 0.574283 | 1.351357 | 1.65153 |
| Rack1 | 1.92E-05 | -0.19311 | 0.997 | 0.992 | 0.596316 | 0.824394 | -1.05916 |
| Rbms1 | 1.93E-05 | 0.251539 | 0.397 | 0.172 | 0.599297 | 1.286003 | 1.379644 |
| Ppp6r1 | 1.93E-05 | 0.365291 | 0.265 | 0.086 | 0.60082 | 1.440934 | 2.003556 |
| Rpl14 | 1.94E-05 | -0.22945 | 0.992 | 0.969 | 0.602781 | 0.794972 | -1.25849 |
| Luc7l2 | 1.99E-05 | 0.267717 | 0.784 | 0.531 | 0.618847 | 1.306977 | 1.468378 |
| Pcmtd1 | 2.14E-05 | 0.343375 | 0.408 | 0.18 | 0.664894 | 1.409697 | 1.883347 |
| 4932438A13Rik | 2.15E-05 | 0.294672 | 0.447 | 0.203 | 0.667181 | 1.342686 | 1.616223 |
| Hbp1 | 2.19E-05 | 0.338933 | 0.356 | 0.141 | 0.680047 | 1.403449 | 1.858985 |
| Snx9 | 2.24E-05 | 0.316311 | 0.335 | 0.125 | 0.696289 | 1.372057 | 1.734908 |
| Lbr | 2.27E-05 | 0.293585 | 0.46 | 0.211 | 0.705292 | 1.341228 | 1.610261 |
| Fam43a | 2.38E-05 | 0.315581 | 0.377 | 0.148 | 0.740368 | 1.371055 | 1.730902 |
| H3f3a | 2.39E-05 | -0.2072 | 0.992 | 1 | 0.742817 | 0.812857 | -1.13645 |
| Csk | 2.47E-05 | 0.20774 | 0.535 | 0.266 | 0.765571 | 1.230894 | 1.139418 |
| Cltc | 2.47E-05 | 0.282236 | 0.439 | 0.195 | 0.76773 | 1.326091 | 1.548011 |
| Ptprj | 2.61E-05 | 0.313523 | 0.361 | 0.141 | 0.809284 | 1.368237 | 1.719617 |
| Cebpb | 2.66E-05 | -0.57287 | 0.558 | 0.648 | 0.825344 | 0.563905 | -3.14208 |
| Ep400 | 2.82E-05 | 0.31213 | 0.429 | 0.188 | 0.874343 | 1.366333 | 1.711978 |
| Rnf4 | 2.86E-05 | 0.274774 | 0.236 | 0.062 | 0.887155 | 1.316234 | 1.507086 |
| Per1 | 2.89E-05 | 0.329517 | 0.377 | 0.156 | 0.897606 | 1.390296 | 1.807338 |
| Runx1 | 2.94E-05 | 0.260196 | 0.358 | 0.141 | 0.913197 | 1.297185 | 1.427129 |
| Hvcn1 | 2.97E-05 | 0.355061 | 0.681 | 0.422 | 0.920952 | 1.426267 | 1.947442 |
| Ppp1r16b | 2.97E-05 | 0.355738 | 0.47 | 0.234 | 0.921307 | 1.427234 | 1.951158 |
| Grk2 | 3.02E-05 | 0.325484 | 0.595 | 0.32 | 0.938244 | 1.3847 | 1.785219 |
| Ptpn18 | 3.25E-05 | -0.36832 | 0.678 | 0.711 | 1 | 0.691897 | -2.02016 |
| Pik3cd | 3.29E-05 | 0.303493 | 0.317 | 0.117 | 1 | 1.354582 | 1.664602 |
| Blnk | 3.33E-05 | 0.281393 | 0.522 | 0.258 | 1 | 1.324975 | 1.543391 |
| Eef1b2 | 3.46E-05 | -0.22567 | 0.974 | 0.984 | 1 | 0.79798 | -1.23777 |
| mt-Nd5 | 3.50E-05 | 0.259313 | 0.912 | 0.703 | 1 | 1.296039 | 1.422282 |
| Dck | 3.54E-05 | 0.309863 | 0.182 | 0.031 | 1 | 1.363239 | 1.699542 |
| Kmt2d | 3.60E-05 | 0.369843 | 0.239 | 0.07 | 1 | 1.447508 | 2.028522 |
| Prdm2 | 3.60E-05 | 0.363014 | 0.371 | 0.164 | 1 | 1.437655 | 1.991063 |
| Limd1 | 3.66E-05 | 0.300866 | 0.429 | 0.195 | 1 | 1.351029 | 1.650196 |
| Pbxip1 | 3.66E-05 | 0.360842 | 0.379 | 0.172 | 1 | 1.434537 | 1.979152 |
| Cox5a | 3.70E-05 | -0.47127 | 0.455 | 0.531 | 1 | 0.624207 | -2.58485 |
| Ccm2 | 3.76E-05 | 0.29114 | 0.39 | 0.164 | 1 | 1.337952 | 1.596849 |
| Ly6c2 | 3.78E-05 | -0.29853 | 0.023 | 0.109 | 1 | 0.741911 | -1.63736 |
| Fcrla | 3.79E-05 | 0.233874 | 0.597 | 0.32 | 1 | 1.263486 | 1.282757 |
| Lrrc8c | 3.90E-05 | 0.311334 | 0.187 | 0.039 | 1 | 1.365246 | 1.707612 |
| Klf4 | 3.91E-05 | 0.39824 | 0.301 | 0.109 | 1 | 1.489202 | 2.184275 |
| Trim7 | 3.99E-05 | 0.336746 | 0.304 | 0.102 | 1 | 1.400384 | 1.846991 |
| Wsb1 | 4.01E-05 | 0.28054 | 0.397 | 0.164 | 1 | 1.323845 | 1.538713 |
| Hnrnpul1 | 4.03E-05 | 0.25714 | 0.348 | 0.133 | 1 | 1.293227 | 1.410368 |
| Lrrc8d | 4.05E-05 | 0.289843 | 0.221 | 0.055 | 1 | 1.336218 | 1.589737 |
| Nop53 | 4.07E-05 | 0.297974 | 0.74 | 0.438 | 1 | 1.347127 | 1.634332 |
| Kdm7a | 4.11E-05 | 0.30439 | 0.571 | 0.312 | 1 | 1.355797 | 1.66952 |
| Fry | 4.13E-05 | 0.316483 | 0.203 | 0.047 | 1 | 1.372292 | 1.735848 |
| Ireb2 | 4.25E-05 | 0.274017 | 0.223 | 0.055 | 1 | 1.315237 | 1.502932 |
| Hipk1 | 4.29E-05 | 0.322364 | 0.462 | 0.227 | 1 | 1.380388 | 1.768109 |
| Atp2b1 | 4.32E-05 | 0.305189 | 0.652 | 0.383 | 1 | 1.356881 | 1.673905 |
| Wdfy4 | 4.39E-05 | 0.357369 | 0.413 | 0.203 | 1 | 1.429564 | 1.960105 |
| Arcn1 | 4.43E-05 | 0.276677 | 0.351 | 0.141 | 1 | 1.318741 | 1.517524 |
| Ubr3 | 4.53E-05 | 0.300435 | 0.177 | 0.031 | 1 | 1.350445 | 1.647828 |
| Qk | 4.55E-05 | 0.303574 | 0.416 | 0.188 | 1 | 1.354692 | 1.665046 |
| Peli1 | 4.76E-05 | 0.332619 | 0.475 | 0.227 | 1 | 1.394615 | 1.824352 |
| Itpr1 | 4.80E-05 | 0.305533 | 0.39 | 0.164 | 1 | 1.357348 | 1.67579 |
| Arhgdib | 4.86E-05 | -0.26696 | 0.904 | 0.914 | 1 | 0.765704 | -1.46423 |
| Slc1a5 | 5.00E-05 | 0.288426 | 0.286 | 0.094 | 1 | 1.334326 | 1.581964 |
| Atf6 | 5.12E-05 | 0.278345 | 0.257 | 0.078 | 1 | 1.320942 | 1.526671 |
| Dlg1 | 5.21E-05 | 0.284292 | 0.19 | 0.039 | 1 | 1.328821 | 1.559291 |
| Cdc42se2 | 5.30E-05 | 0.337759 | 0.379 | 0.172 | 1 | 1.401803 | 1.852546 |
| Txndc11 | 5.41E-05 | 0.300323 | 0.247 | 0.07 | 1 | 1.350295 | 1.647216 |
| Ndufb8 | 5.41E-05 | -0.45596 | 0.275 | 0.398 | 1 | 0.633839 | -2.50086 |
| Lpgat1 | 5.46E-05 | 0.240576 | 0.39 | 0.164 | 1 | 1.271982 | 1.319517 |
| Zmiz1 | 5.51E-05 | 0.311413 | 0.286 | 0.102 | 1 | 1.365353 | 1.708041 |
| Rapgef1 | 5.51E-05 | 0.279 | 0.275 | 0.094 | 1 | 1.321807 | 1.530263 |
| Son | 5.54E-05 | 0.236644 | 0.909 | 0.727 | 1 | 1.26699 | 1.297949 |
| Cxcr5 | 5.59E-05 | 0.256991 | 0.473 | 0.234 | 1 | 1.293034 | 1.40955 |
| Zfp740 | 5.76E-05 | 0.281015 | 0.213 | 0.055 | 1 | 1.324473 | 1.541313 |
| Hnrnpdl | 5.82E-05 | 0.305818 | 0.597 | 0.344 | 1 | 1.357735 | 1.677356 |
| Ccnt1 | 5.89E-05 | 0.259898 | 0.382 | 0.164 | 1 | 1.296798 | 1.425495 |
| Cxcr4 | 5.92E-05 | 0.31499 | 0.836 | 0.625 | 1 | 1.370246 | 1.727663 |
| Nr2c2 | 5.98E-05 | 0.336056 | 0.26 | 0.086 | 1 | 1.399418 | 1.843206 |
| Purb | 6.02E-05 | 0.341294 | 0.657 | 0.383 | 1 | 1.406766 | 1.871932 |
| Mllt10 | 6.10E-05 | 0.286133 | 0.369 | 0.164 | 1 | 1.331269 | 1.569386 |
| Pink1 | 6.10E-05 | 0.259976 | 0.132 | 0.008 | 1 | 1.296899 | 1.42592 |
| Csnk1a1 | 6.17E-05 | 0.27834 | 0.597 | 0.312 | 1 | 1.320935 | 1.526643 |
| Vps13d | 6.18E-05 | 0.311568 | 0.2 | 0.047 | 1 | 1.365564 | 1.708891 |
| Gapvd1 | 6.29E-05 | 0.283565 | 0.306 | 0.117 | 1 | 1.327856 | 1.555303 |
| Tgfb1 | 6.40E-05 | 0.274414 | 0.623 | 0.359 | 1 | 1.315759 | 1.505108 |
| Glud1 | 6.41E-05 | 0.29118 | 0.322 | 0.125 | 1 | 1.338005 | 1.597069 |
| Odc1 | 6.41E-05 | 0.307465 | 0.283 | 0.102 | 1 | 1.359973 | 1.686389 |
| Uba6 | 6.43E-05 | 0.255538 | 0.148 | 0.016 | 1 | 1.291156 | 1.401577 |
| Dync1h1 | 6.52E-05 | 0.264055 | 0.366 | 0.148 | 1 | 1.3022 | 1.448292 |
| Laptm5 | 6.82E-05 | 0.243112 | 0.857 | 0.617 | 1 | 1.275211 | 1.333423 |
| Pum2 | 6.89E-05 | 0.295591 | 0.522 | 0.266 | 1 | 1.343921 | 1.621264 |
| Kmt2c | 7.03E-05 | 0.35197 | 0.442 | 0.219 | 1 | 1.421866 | 1.930492 |
| Fchsd2 | 7.08E-05 | 0.386415 | 0.647 | 0.406 | 1 | 1.471695 | 2.119415 |
| Flna | 7.16E-05 | 0.365191 | 0.434 | 0.211 | 1 | 1.440789 | 2.003005 |
| Plin2 | 7.24E-05 | -0.39013 | 0.052 | 0.156 | 1 | 0.676966 | -2.13981 |
| Cux1 | 7.24E-05 | 0.311801 | 0.369 | 0.156 | 1 | 1.365883 | 1.71017 |
| Mapk8 | 7.28E-05 | 0.284665 | 0.158 | 0.023 | 1 | 1.329317 | 1.561338 |
| Tcf4 | 7.32E-05 | 0.350668 | 0.538 | 0.305 | 1 | 1.420015 | 1.923347 |
| Ptbp1 | 7.45E-05 | 0.331847 | 0.382 | 0.18 | 1 | 1.393539 | 1.820119 |
| Ppib | 7.58E-05 | -0.38095 | 0.571 | 0.617 | 1 | 0.683214 | -2.08943 |
| Gm20186 | 7.73E-05 | 0.290055 | 0.184 | 0.039 | 1 | 1.336502 | 1.590901 |
| Gramd4 | 7.77E-05 | 0.250533 | 0.184 | 0.039 | 1 | 1.28471 | 1.374126 |
| AI504432 | 7.81E-05 | 0.296585 | 0.187 | 0.039 | 1 | 1.345257 | 1.626713 |
| Ppp1r15b | 7.90E-05 | 0.25883 | 0.226 | 0.062 | 1 | 1.295413 | 1.419634 |
| Gpd1l | 8.04E-05 | 0.292387 | 0.247 | 0.078 | 1 | 1.339622 | 1.603691 |
| Cnot6 | 8.14E-05 | 0.2235 | 0.299 | 0.102 | 1 | 1.250446 | 1.225856 |
| Btf3 | 8.17E-05 | -0.26987 | 0.862 | 0.805 | 1 | 0.763477 | -1.4802 |
| Rassf5 | 8.20E-05 | 0.264917 | 0.249 | 0.078 | 1 | 1.303323 | 1.453021 |
| Mzt1 | 8.24E-05 | 0.236887 | 0.161 | 0.023 | 1 | 1.267298 | 1.299281 |
| Nfkb1 | 8.40E-05 | 0.271227 | 0.569 | 0.328 | 1 | 1.311572 | 1.487628 |
| St6gal1 | 8.43E-05 | 0.29911 | 0.306 | 0.117 | 1 | 1.348658 | 1.640564 |
| Inpp5d | 8.54E-05 | 0.267558 | 0.657 | 0.367 | 1 | 1.30677 | 1.467507 |
| Tnrc6a | 8.57E-05 | 0.23142 | 0.426 | 0.203 | 1 | 1.260389 | 1.269298 |
| Rasgrp1 | 9.00E-05 | 0.285008 | 0.236 | 0.07 | 1 | 1.329772 | 1.563214 |
| Gcn1l1 | 9.05E-05 | 0.254695 | 0.187 | 0.039 | 1 | 1.290068 | 1.396955 |
| 2310001H17Rik | 9.12E-05 | -0.38188 | 0.068 | 0.18 | 1 | 0.682577 | -2.09454 |
| Mef2d | 9.23E-05 | 0.286289 | 0.473 | 0.242 | 1 | 1.331477 | 1.570243 |
| Wipf1 | 9.52E-05 | 0.278859 | 0.319 | 0.125 | 1 | 1.321621 | 1.529489 |
| Dynll1 | 9.54E-05 | -0.39543 | 0.566 | 0.625 | 1 | 0.673393 | -2.16884 |
| Prrc2a | 9.70E-05 | 0.280559 | 0.429 | 0.203 | 1 | 1.32387 | 1.538816 |
| Lsp1 | 9.71E-05 | 0.319069 | 0.79 | 0.539 | 1 | 1.375846 | 1.750033 |
| Gnb1 | 9.71E-05 | 0.218061 | 0.387 | 0.164 | 1 | 1.243663 | 1.196022 |
| Gm26917 | 9.80E-05 | 0.265907 | 0.558 | 0.312 | 1 | 1.304614 | 1.45845 |
| Snx30 | 0.000104 | 0.30351 | 0.345 | 0.141 | 1 | 1.354606 | 1.664698 |
| Nsd3 | 0.000106 | 0.260261 | 0.556 | 0.305 | 1 | 1.297268 | 1.427482 |
| Mapre1 | 0.000115 | 0.218093 | 0.39 | 0.172 | 1 | 1.243702 | 1.196197 |
| Atad2b | 0.000116 | 0.334937 | 0.294 | 0.117 | 1 | 1.397853 | 1.83707 |
| Ing1 | 0.000117 | 0.27361 | 0.332 | 0.133 | 1 | 1.314703 | 1.500703 |
| Lnpep | 0.000117 | 0.341472 | 0.455 | 0.234 | 1 | 1.407017 | 1.87291 |
| Nisch | 0.000119 | 0.305521 | 0.351 | 0.148 | 1 | 1.357332 | 1.675728 |
| Tcf25 | 0.000121 | 0.282111 | 0.605 | 0.359 | 1 | 1.325925 | 1.547324 |
| Celf1 | 0.000123 | 0.278598 | 0.387 | 0.18 | 1 | 1.321276 | 1.528057 |
| Ubald2 | 0.000125 | 0.312799 | 0.387 | 0.18 | 1 | 1.367247 | 1.715645 |
| Gmip | 0.000125 | 0.288317 | 0.288 | 0.109 | 1 | 1.33418 | 1.581365 |
| Cop1 | 0.000127 | 0.249809 | 0.351 | 0.156 | 1 | 1.283781 | 1.370158 |
| Cast | 0.000133 | 0.235711 | 0.364 | 0.164 | 1 | 1.265809 | 1.292833 |
| Senp1 | 0.000134 | 0.29352 | 0.179 | 0.039 | 1 | 1.34114 | 1.609901 |
| Ppp2r5a | 0.000135 | 0.275162 | 0.447 | 0.219 | 1 | 1.316744 | 1.509212 |
| Lst1 | 0.000136 | -0.34376 | 0.036 | 0.125 | 1 | 0.709102 | -1.88544 |
| Dennd5a | 0.000137 | 0.301377 | 0.187 | 0.047 | 1 | 1.351719 | 1.652999 |
| Bin2 | 0.000137 | 0.256819 | 0.262 | 0.094 | 1 | 1.29281 | 1.408602 |
| Rictor | 0.000139 | 0.301502 | 0.397 | 0.195 | 1 | 1.351888 | 1.653683 |
| Zbtb44 | 0.00014 | 0.280242 | 0.242 | 0.078 | 1 | 1.323449 | 1.537073 |
| Mycbp2 | 0.000146 | 0.214383 | 0.732 | 0.492 | 1 | 1.239097 | 1.175852 |
| Zbtb20 | 0.000147 | 0.41388 | 0.439 | 0.234 | 1 | 1.512675 | 2.270054 |
| Slc7a1 | 0.000147 | 0.279888 | 0.226 | 0.07 | 1 | 1.322982 | 1.535134 |
| Tial1 | 0.000148 | 0.209543 | 0.231 | 0.07 | 1 | 1.233114 | 1.149302 |
| Adipor2 | 0.000148 | 0.266892 | 0.262 | 0.094 | 1 | 1.305899 | 1.463851 |
| Gdi2 | 0.00015 | 0.294973 | 0.839 | 0.602 | 1 | 1.343091 | 1.617874 |
| Tmem123 | 0.00015 | 0.255832 | 0.6 | 0.359 | 1 | 1.291536 | 1.403192 |
| Man1a | 0.00015 | 0.314117 | 0.655 | 0.422 | 1 | 1.369049 | 1.722871 |
| Kctd12 | 0.000152 | 0.283436 | 0.53 | 0.289 | 1 | 1.327684 | 1.554595 |
| Mxd4 | 0.000156 | 0.271445 | 0.306 | 0.125 | 1 | 1.311859 | 1.488828 |
| Erbin | 0.000162 | 0.322519 | 0.374 | 0.172 | 1 | 1.380601 | 1.768958 |
| Snrnp70 | 0.000163 | 0.260404 | 0.675 | 0.398 | 1 | 1.297454 | 1.428265 |
| Oaz1 | 0.000167 | -0.26247 | 0.919 | 0.914 | 1 | 0.769149 | -1.4396 |
| Fam208a | 0.000176 | 0.272903 | 0.416 | 0.203 | 1 | 1.313773 | 1.496824 |
| B4galnt1 | 0.00018 | 0.268832 | 0.384 | 0.18 | 1 | 1.308435 | 1.474492 |
| Ssr1 | 0.000186 | 0.270483 | 0.255 | 0.094 | 1 | 1.310597 | 1.483548 |
| Acss1 | 0.000195 | 0.285047 | 0.145 | 0.023 | 1 | 1.329824 | 1.563427 |
| Ube3a | 0.000198 | 0.24341 | 0.39 | 0.18 | 1 | 1.275592 | 1.33506 |
| Slc16a6 | 0.000198 | 0.330142 | 0.216 | 0.07 | 1 | 1.391166 | 1.810769 |
| Sh3bgrl3 | 0.000198 | -0.27062 | 0.919 | 0.867 | 1 | 0.762904 | -1.48431 |
| Dusp3 | 0.0002 | 0.274674 | 0.148 | 0.023 | 1 | 1.316101 | 1.506535 |
| Otulin | 0.000203 | 0.23789 | 0.332 | 0.141 | 1 | 1.26857 | 1.304782 |
| Tmem127 | 0.000204 | 0.273426 | 0.145 | 0.023 | 1 | 1.314461 | 1.499693 |
| Sec24c | 0.000205 | 0.271007 | 0.187 | 0.047 | 1 | 1.311285 | 1.486425 |
| Arid1b | 0.000208 | 0.217972 | 0.353 | 0.156 | 1 | 1.243552 | 1.195535 |
| Brd4 | 0.000208 | 0.27717 | 0.475 | 0.25 | 1 | 1.319391 | 1.520226 |
| Puf60 | 0.000209 | 0.288519 | 0.382 | 0.195 | 1 | 1.33445 | 1.582473 |
| Bst2 | 0.000209 | -0.45949 | 0.169 | 0.289 | 1 | 0.631603 | -2.52024 |
| Foxn3 | 0.000212 | 0.264422 | 0.577 | 0.328 | 1 | 1.302678 | 1.450305 |
| Slc12a6 | 0.000213 | 0.209374 | 0.475 | 0.234 | 1 | 1.232906 | 1.14838 |
| Ifnar1 | 0.000216 | 0.257061 | 0.278 | 0.109 | 1 | 1.293124 | 1.409932 |
| Cables2 | 0.000217 | 0.265536 | 0.13 | 0.016 | 1 | 1.304129 | 1.456414 |
| Chka | 0.000222 | 0.290648 | 0.283 | 0.109 | 1 | 1.337294 | 1.594153 |
| Itsn2 | 0.000222 | 0.265642 | 0.561 | 0.336 | 1 | 1.304268 | 1.456997 |
| Dock10 | 0.00023 | 0.2781 | 0.548 | 0.32 | 1 | 1.320618 | 1.525326 |
| Ifi27l2a | 0.00023 | 0.266349 | 0.54 | 0.305 | 1 | 1.30519 | 1.460873 |
| Stag2 | 0.000233 | 0.256621 | 0.397 | 0.188 | 1 | 1.292555 | 1.407516 |
| Cand1 | 0.000234 | 0.225541 | 0.169 | 0.039 | 1 | 1.253001 | 1.237051 |
| Dcaf11 | 0.000235 | 0.232435 | 0.174 | 0.039 | 1 | 1.261668 | 1.274861 |
| Mast4 | 0.000242 | 0.289197 | 0.262 | 0.102 | 1 | 1.335355 | 1.586191 |
| Osbpl8 | 0.000242 | 0.248117 | 0.314 | 0.125 | 1 | 1.28161 | 1.360875 |
| Rprd2 | 0.000243 | 0.227489 | 0.283 | 0.109 | 1 | 1.255444 | 1.247738 |
| Bmt2 | 0.000245 | 0.265207 | 0.143 | 0.023 | 1 | 1.303701 | 1.454612 |
| mt-Nd4l | 0.00025 | 0.242369 | 0.639 | 0.391 | 1 | 1.274265 | 1.32935 |
| Ep300 | 0.000251 | 0.326839 | 0.452 | 0.242 | 1 | 1.386578 | 1.792652 |
| Ptbp2 | 0.000252 | 0.271878 | 0.182 | 0.047 | 1 | 1.312427 | 1.491201 |
| Ppia | 0.000252 | -0.24357 | 0.974 | 0.984 | 1 | 0.783825 | -1.33593 |
| Itch | 0.000252 | 0.230411 | 0.444 | 0.219 | 1 | 1.259118 | 1.263764 |
| Vcpip1 | 0.000252 | 0.25033 | 0.223 | 0.07 | 1 | 1.284449 | 1.373012 |
| Neurl3 | 0.000254 | -0.3987 | 0.657 | 0.727 | 1 | 0.671191 | -2.18681 |
| Sec24b | 0.000255 | 0.231161 | 0.187 | 0.047 | 1 | 1.260062 | 1.267875 |
| Mrpl19 | 0.000258 | 0.220982 | 0.114 | 0.008 | 1 | 1.247301 | 1.212047 |
| Btg1 | 0.000258 | -0.2561 | 0.984 | 0.953 | 1 | 0.774065 | -1.40466 |
| Zcchc6 | 0.000259 | 0.289866 | 0.558 | 0.32 | 1 | 1.336248 | 1.589859 |
| Klf16 | 0.000264 | 0.204131 | 0.114 | 0.008 | 1 | 1.226459 | 1.11962 |
| Tnpo1 | 0.000264 | 0.205186 | 0.288 | 0.109 | 1 | 1.227754 | 1.125408 |
| Pi4ka | 0.000268 | 0.254589 | 0.205 | 0.062 | 1 | 1.289931 | 1.396372 |
| Ino80d | 0.000269 | 0.231908 | 0.353 | 0.164 | 1 | 1.261003 | 1.271971 |
| Eif4g3 | 0.000269 | 0.230617 | 0.356 | 0.164 | 1 | 1.259377 | 1.26489 |
| Rap1gds1 | 0.000271 | 0.257574 | 0.184 | 0.047 | 1 | 1.293788 | 1.412747 |
| Sesn3 | 0.000274 | 0.306827 | 0.369 | 0.18 | 1 | 1.359106 | 1.682891 |
| S1pr4 | 0.000275 | 0.213363 | 0.301 | 0.117 | 1 | 1.237834 | 1.170255 |
| Clint1 | 0.000281 | 0.255509 | 0.392 | 0.18 | 1 | 1.291119 | 1.40142 |
| Rock1 | 0.000283 | 0.210924 | 0.634 | 0.391 | 1 | 1.234818 | 1.156878 |
| Pnpla8 | 0.000283 | 0.291436 | 0.335 | 0.156 | 1 | 1.338348 | 1.598471 |
| Gtf3c2 | 0.000283 | 0.262078 | 0.242 | 0.086 | 1 | 1.299628 | 1.437451 |
| Ankrd13a | 0.000299 | 0.202427 | 0.283 | 0.109 | 1 | 1.224371 | 1.110276 |
| Smchd1 | 0.000303 | 0.264074 | 0.616 | 0.398 | 1 | 1.302225 | 1.448397 |
| Zyg11b | 0.000306 | 0.235071 | 0.306 | 0.125 | 1 | 1.264999 | 1.289324 |
| Kdm5b | 0.000308 | 0.212157 | 0.213 | 0.062 | 1 | 1.236341 | 1.163639 |
| Tnfrsf13c | 0.00031 | 0.274819 | 0.369 | 0.172 | 1 | 1.316293 | 1.507332 |
| Pank3 | 0.000311 | 0.235421 | 0.143 | 0.023 | 1 | 1.265441 | 1.29124 |
| Il4ra | 0.000312 | 0.263072 | 0.356 | 0.164 | 1 | 1.300921 | 1.442903 |
| Arid4b | 0.000313 | 0.262035 | 0.564 | 0.344 | 1 | 1.299572 | 1.437212 |
| Mgea5 | 0.00032 | 0.252703 | 0.353 | 0.164 | 1 | 1.287501 | 1.386032 |
| Fryl | 0.000323 | 0.292094 | 0.283 | 0.117 | 1 | 1.339229 | 1.602082 |
| Syncrip | 0.000327 | 0.185412 | 0.465 | 0.234 | 1 | 1.203715 | 1.016953 |
| Xpo6 | 0.000328 | 0.244953 | 0.229 | 0.078 | 1 | 1.277561 | 1.34352 |
| Ell2 | 0.000329 | 0.191674 | 0.247 | 0.086 | 1 | 1.211276 | 1.051297 |
| Trip12 | 0.000331 | 0.229106 | 0.413 | 0.203 | 1 | 1.257475 | 1.256603 |
| Rcc2 | 0.000332 | 0.258982 | 0.319 | 0.141 | 1 | 1.295611 | 1.420471 |
| Dnajc13 | 0.000333 | 0.252959 | 0.182 | 0.047 | 1 | 1.28783 | 1.387431 |
| Ankrd17 | 0.000333 | 0.272386 | 0.468 | 0.25 | 1 | 1.313094 | 1.493986 |
| Zbtb10 | 0.000333 | 0.234814 | 0.195 | 0.055 | 1 | 1.264673 | 1.287909 |
| Trappc8 | 0.000341 | 0.239698 | 0.153 | 0.031 | 1 | 1.270866 | 1.314702 |
| Arhgap45 | 0.000345 | 0.251912 | 0.618 | 0.383 | 1 | 1.286483 | 1.381691 |
| AU020206 | 0.000352 | 0.241127 | 0.229 | 0.078 | 1 | 1.272683 | 1.322538 |
| S100a6 | 0.000359 | -0.33827 | 0.177 | 0.32 | 1 | 0.713002 | -1.85535 |
| Bag6 | 0.000362 | 0.219158 | 0.169 | 0.039 | 1 | 1.245028 | 1.20204 |
| Birc3 | 0.000362 | 0.220553 | 0.592 | 0.344 | 1 | 1.246766 | 1.209695 |
| Cpsf6 | 0.000364 | 0.274223 | 0.353 | 0.164 | 1 | 1.315508 | 1.504063 |
| Usp38 | 0.000368 | 0.23345 | 0.234 | 0.078 | 1 | 1.262949 | 1.280429 |
| Ppp1r12a | 0.00037 | 0.205056 | 0.46 | 0.234 | 1 | 1.227594 | 1.124696 |
| Mon2 | 0.000372 | 0.22134 | 0.26 | 0.094 | 1 | 1.247748 | 1.214011 |
| Gnai3 | 0.000378 | 0.204782 | 0.294 | 0.117 | 1 | 1.227257 | 1.12319 |
| Map3k1 | 0.000381 | 0.228331 | 0.605 | 0.359 | 1 | 1.256501 | 1.252353 |
| Dnm2 | 0.000383 | 0.245932 | 0.319 | 0.141 | 1 | 1.278813 | 1.348892 |
| Kpna1 | 0.000389 | 0.253314 | 0.27 | 0.109 | 1 | 1.288287 | 1.389378 |
| Eif4g2 | 0.000391 | 0.239934 | 0.79 | 0.586 | 1 | 1.271165 | 1.315994 |
| Gadd45b | 0.000395 | -0.48218 | 0.247 | 0.375 | 1 | 0.617438 | -2.64465 |
| Zfp871 | 0.000399 | 0.215022 | 0.304 | 0.125 | 1 | 1.239889 | 1.179355 |
| Ccni | 0.000401 | 0.266738 | 0.281 | 0.117 | 1 | 1.305699 | 1.463011 |
| Rsf1 | 0.000406 | 0.266541 | 0.379 | 0.188 | 1 | 1.305442 | 1.461931 |
| Insig1 | 0.000408 | 0.275341 | 0.135 | 0.023 | 1 | 1.31698 | 1.510194 |
| Sod1 | 0.000411 | 0.221059 | 0.268 | 0.102 | 1 | 1.247397 | 1.212468 |
| Bdp1 | 0.000415 | 0.266036 | 0.306 | 0.133 | 1 | 1.304782 | 1.459158 |
| Csde1 | 0.000421 | 0.20507 | 0.681 | 0.406 | 1 | 1.227611 | 1.124773 |
| Atg16l2 | 0.000423 | 0.195566 | 0.197 | 0.055 | 1 | 1.215999 | 1.072645 |
| Mark2 | 0.000424 | 0.213349 | 0.377 | 0.188 | 1 | 1.237817 | 1.170182 |
| Junb | 0.000427 | -0.22279 | 0.961 | 1 | 1 | 0.800281 | -1.22197 |
| Ylpm1 | 0.000431 | 0.244997 | 0.226 | 0.078 | 1 | 1.277617 | 1.343761 |
| Mbtps1 | 0.000436 | 0.248375 | 0.226 | 0.078 | 1 | 1.28194 | 1.36229 |
| Nfatc1 | 0.000442 | 0.30557 | 0.273 | 0.117 | 1 | 1.357399 | 1.675997 |
| Trrap | 0.000443 | 0.200157 | 0.223 | 0.078 | 1 | 1.221595 | 1.097826 |
| Trim25 | 0.000444 | 0.324966 | 0.509 | 0.297 | 1 | 1.383984 | 1.782381 |
| Tbl1x | 0.000445 | 0.264446 | 0.197 | 0.062 | 1 | 1.302709 | 1.450439 |
| Ctbp1 | 0.000446 | 0.224126 | 0.327 | 0.141 | 1 | 1.251228 | 1.229287 |
| Sppl3 | 0.000455 | 0.221966 | 0.145 | 0.031 | 1 | 1.248528 | 1.21744 |
| Papd5 | 0.000457 | 0.269986 | 0.148 | 0.031 | 1 | 1.309947 | 1.480826 |
| Trim11 | 0.000457 | 0.285029 | 0.252 | 0.094 | 1 | 1.329801 | 1.563334 |
| Stat5b | 0.000463 | 0.214695 | 0.164 | 0.039 | 1 | 1.239484 | 1.177564 |
| Slc4a7 | 0.000469 | 0.275651 | 0.392 | 0.195 | 1 | 1.317388 | 1.511893 |
| Kif5b | 0.000472 | 0.187813 | 0.548 | 0.289 | 1 | 1.206607 | 1.030117 |
| Zbtb7a | 0.000477 | 0.254209 | 0.426 | 0.219 | 1 | 1.289441 | 1.394288 |
| Uhrf2 | 0.000485 | 0.270026 | 0.309 | 0.141 | 1 | 1.309998 | 1.481042 |
| Midn | 0.000487 | 0.189677 | 0.255 | 0.094 | 1 | 1.20886 | 1.040346 |
| Gorasp2 | 0.000488 | 0.194822 | 0.221 | 0.078 | 1 | 1.215094 | 1.06856 |
| Atf7 | 0.000494 | 0.242412 | 0.247 | 0.094 | 1 | 1.274319 | 1.329584 |
| Hsp90aa1 | 0.000494 | -0.31483 | 0.836 | 0.82 | 1 | 0.729916 | -1.72676 |
| Ptbp3 | 0.000498 | 0.271338 | 0.745 | 0.516 | 1 | 1.311718 | 1.488239 |
| Bicd2 | 0.000501 | 0.184676 | 0.119 | 0.016 | 1 | 1.202829 | 1.012915 |
| Abca3 | 0.000502 | 0.196122 | 0.135 | 0.023 | 1 | 1.216676 | 1.075695 |
| L3mbtl3 | 0.000514 | 0.254753 | 0.171 | 0.047 | 1 | 1.290142 | 1.397271 |
| Rpl7a | 0.000516 | -0.18369 | 0.971 | 0.961 | 1 | 0.832193 | -1.00751 |
| Mbnl2 | 0.000519 | 0.251124 | 0.343 | 0.164 | 1 | 1.28547 | 1.377369 |
| Dhx30 | 0.000519 | 0.22477 | 0.171 | 0.047 | 1 | 1.252035 | 1.232822 |
| Mtmr3 | 0.000523 | 0.243075 | 0.249 | 0.094 | 1 | 1.275164 | 1.333221 |
| Zfp106 | 0.000532 | 0.251306 | 0.449 | 0.25 | 1 | 1.285703 | 1.378365 |
| Akna | 0.000535 | 0.235634 | 0.327 | 0.148 | 1 | 1.265711 | 1.292411 |
| Zcchc2 | 0.000538 | 0.219506 | 0.164 | 0.039 | 1 | 1.245461 | 1.203949 |
| Aff3 | 0.000538 | 0.221541 | 0.319 | 0.141 | 1 | 1.247998 | 1.215109 |
| Batf | 0.000543 | -0.33653 | 0.052 | 0.141 | 1 | 0.714246 | -1.84579 |
| Zcchc11 | 0.000544 | 0.256215 | 0.81 | 0.562 | 1 | 1.29203 | 1.405291 |
| Mga | 0.000551 | 0.215998 | 0.387 | 0.188 | 1 | 1.241099 | 1.184707 |
| Rbm17 | 0.000554 | 0.222272 | 0.234 | 0.086 | 1 | 1.248912 | 1.219123 |
| Hcfc1 | 0.000556 | 0.243258 | 0.184 | 0.055 | 1 | 1.275397 | 1.334223 |
| Pou2af1 | 0.000558 | 0.281298 | 0.665 | 0.469 | 1 | 1.324848 | 1.542867 |
| Fnbp1 | 0.000561 | 0.309278 | 0.327 | 0.164 | 1 | 1.362441 | 1.696333 |
| Arid3b | 0.000567 | 0.200568 | 0.184 | 0.055 | 1 | 1.222097 | 1.100077 |
| Fam193a | 0.000569 | 0.262382 | 0.26 | 0.102 | 1 | 1.300024 | 1.439119 |
| Tnrc6b | 0.000569 | 0.255536 | 0.605 | 0.383 | 1 | 1.291153 | 1.401567 |
| 1110008F13Rik | 0.000571 | 0.242809 | 0.223 | 0.078 | 1 | 1.274825 | 1.331761 |
| Eif2s3x | 0.000572 | 0.216655 | 0.171 | 0.047 | 1 | 1.241915 | 1.188312 |
| Smarca4 | 0.000573 | 0.2167 | 0.294 | 0.125 | 1 | 1.241972 | 1.188562 |
| Man2a1 | 0.000576 | 0.236644 | 0.288 | 0.125 | 1 | 1.266991 | 1.297951 |
| Wbp11 | 0.000579 | 0.19778 | 0.377 | 0.18 | 1 | 1.218694 | 1.084786 |
| Ndufa3 | 0.00058 | 0.244122 | 0.434 | 0.234 | 1 | 1.276499 | 1.338961 |
| Phip | 0.000581 | 0.25777 | 0.623 | 0.383 | 1 | 1.294041 | 1.413821 |
| Ttc3 | 0.000581 | 0.298603 | 0.223 | 0.086 | 1 | 1.347975 | 1.637783 |
| Tcp11l2 | 0.000581 | 0.256954 | 0.543 | 0.328 | 1 | 1.292985 | 1.409342 |
| Elmsan1 | 0.000586 | 0.265591 | 0.223 | 0.078 | 1 | 1.304201 | 1.456716 |
| Tnk2 | 0.000588 | 0.190772 | 0.104 | 0.008 | 1 | 1.210183 | 1.046348 |
| Dido1 | 0.000589 | 0.233339 | 0.242 | 0.094 | 1 | 1.262809 | 1.279819 |
| Pik3c3 | 0.000592 | 0.220633 | 0.101 | 0.008 | 1 | 1.246866 | 1.210134 |
| Nsd1 | 0.000617 | 0.242919 | 0.6 | 0.352 | 1 | 1.274965 | 1.332366 |
| Lemd3 | 0.000618 | 0.193961 | 0.174 | 0.047 | 1 | 1.214049 | 1.063838 |
| Ccnl1 | 0.000619 | 0.240964 | 0.701 | 0.469 | 1 | 1.272476 | 1.321645 |
| Acbd5 | 0.000619 | 0.205465 | 0.2 | 0.062 | 1 | 1.228095 | 1.126935 |
| Ythdc1 | 0.000619 | 0.291709 | 0.566 | 0.344 | 1 | 1.338714 | 1.599972 |
| Pds5a | 0.000631 | 0.265281 | 0.369 | 0.188 | 1 | 1.303797 | 1.455018 |
| Gsk3a | 0.000631 | 0.20364 | 0.226 | 0.078 | 1 | 1.225857 | 1.11693 |
| Esyt2 | 0.00064 | 0.241074 | 0.208 | 0.07 | 1 | 1.272616 | 1.322248 |
| Phc3 | 0.000641 | 0.216163 | 0.2 | 0.062 | 1 | 1.241305 | 1.185616 |
| Golga4 | 0.000644 | 0.281877 | 0.252 | 0.102 | 1 | 1.325615 | 1.54604 |
| Hmgcs1 | 0.000649 | 0.221058 | 0.117 | 0.016 | 1 | 1.247396 | 1.212465 |
| Rasal3 | 0.000652 | 0.232494 | 0.221 | 0.078 | 1 | 1.261743 | 1.275189 |
| Ccr7 | 0.000661 | 0.287737 | 0.805 | 0.594 | 1 | 1.333406 | 1.578183 |
| Treml2 | 0.000661 | 0.26394 | 0.275 | 0.117 | 1 | 1.30205 | 1.447661 |
| Kctd3 | 0.000662 | 0.219585 | 0.117 | 0.016 | 1 | 1.24556 | 1.204383 |
| Sp100 | 0.000667 | 0.220054 | 0.665 | 0.414 | 1 | 1.246144 | 1.206956 |
| Spopl | 0.000676 | 0.204352 | 0.21 | 0.07 | 1 | 1.22673 | 1.120835 |
| Cdc37l1 | 0.000677 | 0.266765 | 0.327 | 0.148 | 1 | 1.305734 | 1.463159 |
| Polr2l | 0.000678 | 0.192664 | 0.132 | 0.023 | 1 | 1.212475 | 1.056724 |
| Mef2c | 0.000694 | 0.266159 | 0.935 | 0.805 | 1 | 1.304942 | 1.459833 |
| Copg1 | 0.000694 | 0.209748 | 0.156 | 0.039 | 1 | 1.233367 | 1.150427 |
| Kmt5b | 0.000697 | 0.211599 | 0.314 | 0.141 | 1 | 1.235653 | 1.160583 |
| Slc26a2 | 0.000699 | 0.252192 | 0.127 | 0.023 | 1 | 1.286843 | 1.383225 |
| Zfp729a | 0.0007 | 0.244921 | 0.117 | 0.016 | 1 | 1.27752 | 1.343345 |
| Adss | 0.000703 | 0.239445 | 0.27 | 0.117 | 1 | 1.270544 | 1.313314 |
| Xiap | 0.000711 | 0.267064 | 0.335 | 0.164 | 1 | 1.306124 | 1.464798 |
| Rps6ka3 | 0.000725 | 0.230146 | 0.171 | 0.047 | 1 | 1.258784 | 1.262308 |
| Psmb8 | 0.00073 | -0.37095 | 0.564 | 0.578 | 1 | 0.690081 | -2.03457 |
| Myo1c | 0.000734 | 0.196211 | 0.322 | 0.148 | 1 | 1.216784 | 1.076181 |
| Gatad2b | 0.00074 | 0.235227 | 0.47 | 0.258 | 1 | 1.265196 | 1.290176 |
| Pik3ca | 0.000741 | 0.267131 | 0.239 | 0.094 | 1 | 1.306211 | 1.465163 |
| Gxylt1 | 0.000757 | 0.261034 | 0.239 | 0.094 | 1 | 1.298272 | 1.431725 |
| Mcmbp | 0.000763 | 0.23734 | 0.239 | 0.094 | 1 | 1.267872 | 1.301764 |
| Tet2 | 0.000767 | 0.195424 | 0.265 | 0.109 | 1 | 1.215826 | 1.071864 |
| Nrbf2 | 0.000778 | 0.224567 | 0.13 | 0.023 | 1 | 1.251781 | 1.231709 |
| Cnot6l | 0.000796 | 0.248801 | 0.577 | 0.328 | 1 | 1.282487 | 1.36463 |
| Ezr | 0.000805 | 0.226681 | 0.722 | 0.445 | 1 | 1.254429 | 1.243301 |
| Zfp148 | 0.000813 | 0.204925 | 0.345 | 0.172 | 1 | 1.227433 | 1.123974 |
| Srrt | 0.000817 | 0.225194 | 0.252 | 0.102 | 1 | 1.252566 | 1.235148 |
| Pacs1 | 0.000823 | 0.219316 | 0.143 | 0.031 | 1 | 1.245224 | 1.202906 |
| Lpp | 0.000824 | 0.302134 | 0.275 | 0.125 | 1 | 1.352743 | 1.657152 |
| Ndufa13 | 0.000825 | -0.29253 | 0.512 | 0.555 | 1 | 0.746373 | -1.60447 |
| Numa1 | 0.000826 | 0.21192 | 0.374 | 0.18 | 1 | 1.236049 | 1.162343 |
| Syngr2 | 0.000835 | 0.228902 | 0.332 | 0.156 | 1 | 1.257219 | 1.255488 |
| Uso1 | 0.000837 | 0.215155 | 0.205 | 0.07 | 1 | 1.240055 | 1.180088 |
| Dennd5b | 0.000853 | 0.250111 | 0.283 | 0.125 | 1 | 1.284167 | 1.37181 |
| Inpp4b | 0.000855 | 0.22671 | 0.14 | 0.031 | 1 | 1.254466 | 1.243461 |
| Tcf20 | 0.000857 | 0.222625 | 0.278 | 0.117 | 1 | 1.249352 | 1.221056 |
| Mapk14 | 0.000864 | 0.204259 | 0.153 | 0.039 | 1 | 1.226616 | 1.120324 |
| Fbxw2 | 0.000865 | 0.212552 | 0.268 | 0.109 | 1 | 1.23683 | 1.165807 |
| Cep68 | 0.000878 | 0.187681 | 0.114 | 0.016 | 1 | 1.206448 | 1.029394 |
| Smc4 | 0.000886 | 0.285364 | 0.314 | 0.148 | 1 | 1.330246 | 1.565168 |
| Tardbp | 0.000899 | 0.235726 | 0.325 | 0.156 | 1 | 1.265827 | 1.292913 |
| Hist1h1e | 0.000905 | -0.58319 | 0.197 | 0.305 | 1 | 0.558114 | -3.1987 |
| Tsc1 | 0.000918 | 0.211811 | 0.14 | 0.031 | 1 | 1.235915 | 1.161745 |
| Gna13 | 0.00093 | 0.1842 | 0.46 | 0.242 | 1 | 1.202257 | 1.010305 |
| Rpl22l1 | 0.000933 | -0.28418 | 0.823 | 0.82 | 1 | 0.752631 | -1.55867 |
| Washc2 | 0.000937 | 0.245643 | 0.252 | 0.102 | 1 | 1.278443 | 1.347307 |
| Bcl2a1b | 0.00094 | -0.26368 | 0.247 | 0.367 | 1 | 0.768221 | -1.44622 |
| Irf8 | 0.000944 | 0.198625 | 0.571 | 0.344 | 1 | 1.219724 | 1.089421 |
| Tnrc18 | 0.000945 | 0.21494 | 0.153 | 0.039 | 1 | 1.239787 | 1.178904 |
| Usp6nl | 0.000946 | 0.247296 | 0.164 | 0.047 | 1 | 1.280559 | 1.356375 |
| Ciita | 0.000948 | 0.271876 | 0.465 | 0.25 | 1 | 1.312425 | 1.491192 |
| Sh2b3 | 0.000963 | 0.207213 | 0.361 | 0.18 | 1 | 1.230244 | 1.136524 |
| Cap1 | 0.000989 | 0.24981 | 0.319 | 0.156 | 1 | 1.283781 | 1.370161 |
| Cdt1 | 0.000993 | 0.234377 | 0.294 | 0.133 | 1 | 1.264121 | 1.285516 |
| Setd1b | 0.000994 | 0.229472 | 0.164 | 0.047 | 1 | 1.257936 | 1.258612 |
| 9530068E07Rik | 0.000998 | 0.210019 | 0.164 | 0.047 | 1 | 1.233702 | 1.151916 |
| Ubr1 | 0.000999 | 0.209369 | 0.153 | 0.039 | 1 | 1.2329 | 1.148352 |
| Pcm1 | 0.001004 | 0.23469 | 0.301 | 0.133 | 1 | 1.264516 | 1.287229 |
| Ibtk | 0.001007 | 0.246612 | 0.195 | 0.07 | 1 | 1.279683 | 1.352622 |
| Dcaf8 | 0.00103 | 0.240487 | 0.306 | 0.148 | 1 | 1.271868 | 1.319026 |
| Il10ra | 0.001032 | 0.209828 | 0.351 | 0.172 | 1 | 1.233466 | 1.15087 |
| Ly6a | 0.001037 | -0.47265 | 0.361 | 0.469 | 1 | 0.62335 | -2.59238 |
| Iws1 | 0.001038 | 0.200244 | 0.291 | 0.133 | 1 | 1.221701 | 1.098303 |
| Fcho2 | 0.001046 | 0.220613 | 0.244 | 0.102 | 1 | 1.24684 | 1.21002 |
| Heatr6 | 0.001048 | 0.249488 | 0.187 | 0.062 | 1 | 1.283368 | 1.368397 |
| Got1 | 0.001052 | 0.238659 | 0.345 | 0.172 | 1 | 1.269546 | 1.309001 |
| Gabarap | 0.001058 | -0.28665 | 0.6 | 0.641 | 1 | 0.750773 | -1.57224 |
| Tram1 | 0.001058 | 0.195199 | 0.374 | 0.188 | 1 | 1.215553 | 1.070629 |
| Kbtbd2 | 0.001059 | 0.18716 | 0.166 | 0.047 | 1 | 1.20582 | 1.026536 |
| Mepce | 0.001063 | 0.195923 | 0.301 | 0.133 | 1 | 1.216433 | 1.074602 |
| Sgms1 | 0.001063 | 0.281029 | 0.371 | 0.188 | 1 | 1.324492 | 1.54139 |
| Map3k2 | 0.001068 | 0.264481 | 0.197 | 0.07 | 1 | 1.302755 | 1.450632 |
| Ubl3 | 0.001069 | 0.263742 | 0.532 | 0.328 | 1 | 1.301793 | 1.446578 |
| Foxk1 | 0.001074 | 0.196169 | 0.125 | 0.023 | 1 | 1.216733 | 1.075952 |
| Pafah1b2 | 0.001082 | 0.183483 | 0.169 | 0.047 | 1 | 1.201395 | 1.006371 |
| Casp3 | 0.001084 | 0.186729 | 0.125 | 0.023 | 1 | 1.205301 | 1.024175 |
| Ier2 | 0.001087 | -0.39443 | 0.673 | 0.727 | 1 | 0.674064 | -2.16338 |
| Secisbp2 | 0.001092 | 0.219355 | 0.153 | 0.039 | 1 | 1.245273 | 1.203121 |
| Tagln2 | 0.001096 | 0.458941 | 0.413 | 0.234 | 1 | 1.582397 | 2.517207 |
| Cul3 | 0.0011 | 0.199073 | 0.408 | 0.219 | 1 | 1.220271 | 1.091879 |
| Gng5 | 0.001108 | -0.30378 | 0.634 | 0.625 | 1 | 0.738025 | -1.66616 |
| Ddx25 | 0.001111 | -0.34274 | 0.057 | 0.141 | 1 | 0.70982 | -1.87989 |
| Vpreb3 | 0.001112 | -0.3625 | 0.481 | 0.562 | 1 | 0.695932 | -1.98826 |
| Arhgap4 | 0.001118 | 0.198802 | 0.252 | 0.102 | 1 | 1.21994 | 1.090392 |
| Mecp2 | 0.001127 | 0.264784 | 0.208 | 0.078 | 1 | 1.30315 | 1.452292 |
| Rif1 | 0.001131 | 0.223548 | 0.213 | 0.078 | 1 | 1.250506 | 1.226121 |
| Ccnt2 | 0.001138 | 0.216276 | 0.27 | 0.117 | 1 | 1.241445 | 1.186233 |
| Selenow | 0.001166 | -0.42561 | 0.564 | 0.578 | 1 | 0.653368 | -2.33442 |
| Huwe1 | 0.001167 | 0.217506 | 0.488 | 0.273 | 1 | 1.242973 | 1.19298 |
| Slc15a2 | 0.001167 | 0.379254 | 0.143 | 0.039 | 1 | 1.461194 | 2.080136 |
| Xpr1 | 0.00118 | 0.259918 | 0.338 | 0.164 | 1 | 1.296823 | 1.4256 |
| Cd83 | 0.001217 | 0.297876 | 0.675 | 0.461 | 1 | 1.346995 | 1.633797 |
| Clic4 | 0.001223 | 0.266239 | 0.231 | 0.094 | 1 | 1.305047 | 1.46027 |
| Lrrc58 | 0.001245 | 0.21365 | 0.148 | 0.039 | 1 | 1.238189 | 1.17183 |
| Unc50 | 0.001253 | -0.3447 | 0.109 | 0.211 | 1 | 0.708434 | -1.8906 |
| Gpr174 | 0.001253 | 0.222875 | 0.268 | 0.117 | 1 | 1.249664 | 1.222428 |
| Zfp703 | 0.00126 | 0.194554 | 0.109 | 0.016 | 1 | 1.214769 | 1.067092 |
| Prpf38a | 0.001267 | 0.224441 | 0.171 | 0.055 | 1 | 1.251622 | 1.231016 |
| Zmym5 | 0.00127 | 0.20517 | 0.301 | 0.141 | 1 | 1.227733 | 1.125318 |
| Cbfb | 0.001272 | 0.188975 | 0.213 | 0.078 | 1 | 1.208011 | 1.036492 |
| Edem3 | 0.001275 | 0.209411 | 0.106 | 0.016 | 1 | 1.232952 | 1.148583 |
| Sp1 | 0.001275 | 0.251616 | 0.286 | 0.133 | 1 | 1.286102 | 1.380066 |
| Atf7ip | 0.001293 | 0.218276 | 0.418 | 0.227 | 1 | 1.24393 | 1.197201 |
| Pou2f2 | 0.001312 | 0.268978 | 0.678 | 0.484 | 1 | 1.308626 | 1.475295 |
| Naa15 | 0.001317 | 0.22664 | 0.304 | 0.148 | 1 | 1.254379 | 1.243081 |
| Kat6a | 0.001326 | 0.208678 | 0.387 | 0.195 | 1 | 1.232048 | 1.14456 |
| Acox3 | 0.001333 | 0.221617 | 0.171 | 0.055 | 1 | 1.248093 | 1.215529 |
| Gls | 0.001338 | 0.277803 | 0.553 | 0.359 | 1 | 1.320226 | 1.523698 |
| Smad5 | 0.00134 | 0.18655 | 0.106 | 0.016 | 1 | 1.205085 | 1.023191 |
| Tspo | 0.001343 | -0.37708 | 0.379 | 0.453 | 1 | 0.685861 | -2.06821 |
| Pde4b | 0.001346 | 0.245596 | 0.647 | 0.43 | 1 | 1.278383 | 1.347048 |
| Rab43 | 0.001353 | 0.200975 | 0.106 | 0.016 | 1 | 1.222594 | 1.102309 |
| Ints7 | 0.001363 | 0.244734 | 0.132 | 0.031 | 1 | 1.277281 | 1.342319 |
| Ptk2b | 0.001367 | 0.24217 | 0.506 | 0.305 | 1 | 1.274011 | 1.328259 |
| Hexim1 | 0.001368 | 0.261328 | 0.395 | 0.211 | 1 | 1.298654 | 1.433336 |
| Cobll1 | 0.001372 | 0.225486 | 0.148 | 0.039 | 1 | 1.252932 | 1.236751 |
| Cr2 | 0.001375 | 0.282573 | 0.343 | 0.172 | 1 | 1.326539 | 1.549863 |
| Ogt | 0.001395 | 0.225792 | 0.579 | 0.344 | 1 | 1.253315 | 1.238429 |
| Ube2h | 0.001402 | 0.215057 | 0.33 | 0.156 | 1 | 1.239933 | 1.17955 |
| 2410002F23Rik | 0.001415 | 0.227922 | 0.156 | 0.047 | 1 | 1.255987 | 1.250108 |
| Ints8 | 0.001419 | 0.212842 | 0.119 | 0.023 | 1 | 1.237189 | 1.167399 |
| Phrf1 | 0.001431 | 0.246794 | 0.177 | 0.062 | 1 | 1.279915 | 1.353618 |
| Smarca2 | 0.001436 | 0.230682 | 0.252 | 0.109 | 1 | 1.259459 | 1.265248 |
| Jmy | 0.001436 | 0.222162 | 0.145 | 0.039 | 1 | 1.248774 | 1.21852 |
| Bsdc1 | 0.001439 | 0.185237 | 0.177 | 0.055 | 1 | 1.203504 | 1.015992 |
| Mkln1 | 0.001444 | 0.246221 | 0.371 | 0.195 | 1 | 1.279182 | 1.350475 |
| Rpa1 | 0.00146 | 0.219077 | 0.158 | 0.047 | 1 | 1.244927 | 1.201598 |
| Nktr | 0.001465 | 0.211929 | 0.587 | 0.375 | 1 | 1.23606 | 1.162391 |
| Zc3h10 | 0.001473 | 0.187231 | 0.145 | 0.039 | 1 | 1.205906 | 1.026929 |
| Hspa8 | 0.00151 | -0.18442 | 0.969 | 0.977 | 1 | 0.831587 | -1.01151 |
| Bicra | 0.001523 | 0.190725 | 0.171 | 0.055 | 1 | 1.210126 | 1.04609 |
| Msrb1 | 0.001528 | -0.3889 | 0.158 | 0.258 | 1 | 0.677802 | -2.13305 |
| Ube2d2a | 0.001549 | 0.189559 | 0.405 | 0.211 | 1 | 1.208717 | 1.039697 |
| Chtf8 | 0.00155 | 0.191486 | 0.148 | 0.039 | 1 | 1.211048 | 1.050265 |
| Polr1d | 0.001552 | -0.2942 | 0.668 | 0.672 | 1 | 0.745129 | -1.61362 |
| Pten | 0.00157 | 0.208174 | 0.6 | 0.391 | 1 | 1.231428 | 1.141798 |
| Pdcd4 | 0.001571 | 0.291511 | 0.556 | 0.375 | 1 | 1.338449 | 1.598886 |
| Uimc1 | 0.001572 | 0.21465 | 0.223 | 0.094 | 1 | 1.239428 | 1.177317 |
| Btla | 0.001584 | 0.217612 | 0.517 | 0.305 | 1 | 1.243105 | 1.193562 |
| Dcaf17 | 0.001612 | 0.231359 | 0.104 | 0.016 | 1 | 1.260312 | 1.268961 |
| Arrdc3 | 0.001615 | 0.242765 | 0.117 | 0.023 | 1 | 1.274769 | 1.33152 |
| Tyrobp | 0.001622 | -0.38626 | 0.239 | 0.336 | 1 | 0.679593 | -2.11857 |
| Golgb1 | 0.00164 | 0.2781 | 0.213 | 0.086 | 1 | 1.320619 | 1.525329 |
| Sirt1 | 0.001647 | 0.241816 | 0.26 | 0.117 | 1 | 1.27356 | 1.326315 |
| Gga2 | 0.001657 | 0.221484 | 0.397 | 0.219 | 1 | 1.247927 | 1.214799 |
| Zmym2 | 0.001699 | 0.191618 | 0.213 | 0.078 | 1 | 1.211208 | 1.05099 |
| Pim1 | 0.001711 | 0.215763 | 0.639 | 0.414 | 1 | 1.240809 | 1.183422 |
| Pcnt | 0.001725 | 0.227084 | 0.171 | 0.055 | 1 | 1.254935 | 1.245513 |
| Crkl | 0.001728 | 0.20041 | 0.143 | 0.039 | 1 | 1.221904 | 1.099213 |
| Gm26699 | 0.001729 | -0.38156 | 0.112 | 0.203 | 1 | 0.682799 | -2.09276 |
| Klf7 | 0.001738 | 0.187656 | 0.216 | 0.086 | 1 | 1.206419 | 1.02926 |
| Snx29 | 0.001764 | 0.30227 | 0.296 | 0.148 | 1 | 1.352927 | 1.657897 |
| Tomm20 | 0.001785 | -0.31579 | 0.514 | 0.539 | 1 | 0.729212 | -1.73205 |
| Atxn2l | 0.001798 | 0.200115 | 0.351 | 0.18 | 1 | 1.221543 | 1.097592 |
| Fuca1 | 0.001814 | 0.220273 | 0.312 | 0.156 | 1 | 1.246418 | 1.208159 |
| Insr | 0.001825 | 0.226566 | 0.143 | 0.039 | 1 | 1.254285 | 1.242672 |
| Fam102a | 0.001825 | 0.196641 | 0.143 | 0.039 | 1 | 1.217307 | 1.078542 |
| Tfrc | 0.001828 | 0.189049 | 0.182 | 0.062 | 1 | 1.2081 | 1.0369 |
| Mid1 | 0.001848 | 0.191818 | 0.117 | 0.023 | 1 | 1.21145 | 1.052087 |
| Cd164 | 0.001864 | 0.197612 | 0.395 | 0.219 | 1 | 1.21849 | 1.083868 |
| Med1 | 0.001877 | 0.208002 | 0.273 | 0.125 | 1 | 1.231215 | 1.14085 |
| Washc4 | 0.001891 | 0.229503 | 0.234 | 0.102 | 1 | 1.257975 | 1.258781 |
| Prkdc | 0.001917 | 0.22586 | 0.114 | 0.023 | 1 | 1.253401 | 1.238803 |
| Abl1 | 0.00192 | 0.202841 | 0.101 | 0.016 | 1 | 1.224878 | 1.112546 |
| Emsy | 0.001938 | 0.225265 | 0.27 | 0.125 | 1 | 1.252655 | 1.235539 |
| Scaf8 | 0.001939 | 0.183068 | 0.195 | 0.07 | 1 | 1.200896 | 1.004093 |
| Dennd6a | 0.001969 | 0.221724 | 0.192 | 0.07 | 1 | 1.248226 | 1.216113 |
| Ap1s3 | 0.001979 | 0.184774 | 0.132 | 0.031 | 1 | 1.202947 | 1.013452 |
| Gigyf2 | 0.00201 | 0.215697 | 0.203 | 0.078 | 1 | 1.240726 | 1.183058 |
| Gtf2a1 | 0.002015 | 0.199155 | 0.192 | 0.07 | 1 | 1.220371 | 1.092328 |
| Gse1 | 0.002017 | 0.193596 | 0.179 | 0.062 | 1 | 1.213606 | 1.061838 |
| Fgd2 | 0.002019 | 0.239493 | 0.197 | 0.078 | 1 | 1.270604 | 1.313573 |
| Abce1 | 0.002027 | 0.220937 | 0.203 | 0.078 | 1 | 1.247245 | 1.2118 |
| Dcp2 | 0.002054 | 0.202249 | 0.127 | 0.031 | 1 | 1.224153 | 1.1093 |
| Mindy2 | 0.002064 | 0.183245 | 0.231 | 0.094 | 1 | 1.201108 | 1.005062 |
| Med13l | 0.002065 | 0.202837 | 0.304 | 0.148 | 1 | 1.224873 | 1.112525 |
| Zzef1 | 0.002067 | 0.231865 | 0.208 | 0.086 | 1 | 1.260949 | 1.271736 |
| Aqr | 0.002079 | 0.195373 | 0.275 | 0.125 | 1 | 1.215765 | 1.071586 |
| Ubn2 | 0.002095 | 0.191777 | 0.397 | 0.227 | 1 | 1.211401 | 1.051864 |
| Ube2j1 | 0.002098 | 0.206709 | 0.19 | 0.07 | 1 | 1.229625 | 1.133761 |
| Dyrk1a | 0.00213 | 0.197301 | 0.216 | 0.086 | 1 | 1.21811 | 1.082158 |
| Setx | 0.002139 | 0.204866 | 0.34 | 0.172 | 1 | 1.22736 | 1.123651 |
| Metap2 | 0.00214 | 0.211155 | 0.314 | 0.156 | 1 | 1.235103 | 1.158145 |
| Slfn2 | 0.002151 | -0.39792 | 0.525 | 0.594 | 1 | 0.671714 | -2.18253 |
| Cdc25b | 0.002159 | 0.205033 | 0.153 | 0.047 | 1 | 1.227566 | 1.12457 |
| Ccl5 | 0.002168 | -0.32919 | 0.078 | 0.164 | 1 | 0.719503 | -1.80557 |
| Zc3h13 | 0.002188 | 0.188805 | 0.325 | 0.164 | 1 | 1.207806 | 1.035561 |
| Smarcd2 | 0.002205 | 0.228913 | 0.221 | 0.094 | 1 | 1.257233 | 1.255547 |
| Samd1 | 0.002232 | 0.184167 | 0.114 | 0.023 | 1 | 1.202216 | 1.010121 |
| Trak2 | 0.002254 | 0.200569 | 0.182 | 0.062 | 1 | 1.222099 | 1.100087 |
| Arhgap15 | 0.002288 | 0.22222 | 0.709 | 0.5 | 1 | 1.248846 | 1.218834 |
| Cpt1a | 0.002306 | 0.207223 | 0.138 | 0.039 | 1 | 1.230257 | 1.13658 |
| Ndufb11 | 0.002316 | -0.27492 | 0.494 | 0.555 | 1 | 0.759635 | -1.50787 |
| Rnf144a | 0.002345 | 0.22278 | 0.223 | 0.094 | 1 | 1.249545 | 1.221906 |
| Itm2b | 0.002346 | -0.25311 | 0.758 | 0.742 | 1 | 0.776382 | -1.38826 |
| Jade1 | 0.002379 | 0.194065 | 0.143 | 0.039 | 1 | 1.214175 | 1.064411 |
| Madd | 0.002383 | 0.228307 | 0.252 | 0.117 | 1 | 1.256471 | 1.252223 |
| Sept7 | 0.002392 | -0.34445 | 0.408 | 0.469 | 1 | 0.708609 | -1.88925 |
| 2810403A07Rik | 0.002477 | 0.185073 | 0.234 | 0.102 | 1 | 1.203306 | 1.01509 |
| Btg2 | 0.002486 | -0.26389 | 0.896 | 0.844 | 1 | 0.76806 | -1.44737 |
| Slc30a5 | 0.002535 | 0.222112 | 0.2 | 0.078 | 1 | 1.248712 | 1.218246 |
| Kansl3 | 0.002579 | 0.207335 | 0.205 | 0.086 | 1 | 1.230395 | 1.137195 |
| Cers4 | 0.002606 | 0.261921 | 0.205 | 0.086 | 1 | 1.299424 | 1.436589 |
| Sat1 | 0.002612 | -0.36436 | 0.473 | 0.531 | 1 | 0.694639 | -1.99846 |
| Cyba | 0.002628 | -0.2539 | 0.816 | 0.828 | 1 | 0.775773 | -1.39257 |
| C1galt1 | 0.002633 | 0.202305 | 0.299 | 0.141 | 1 | 1.224221 | 1.109603 |
| Iglc3 | 0.002652 | -0.41688 | 0.629 | 0.656 | 1 | 0.6591 | -2.28651 |
| Sec16a | 0.002666 | 0.203345 | 0.161 | 0.055 | 1 | 1.225496 | 1.115311 |
| Rabep1 | 0.002684 | 0.194709 | 0.221 | 0.094 | 1 | 1.214957 | 1.067942 |
| Atxn7 | 0.002692 | 0.203233 | 0.208 | 0.086 | 1 | 1.225358 | 1.114694 |
| Apoe | 0.002725 | -0.6888 | 0.382 | 0.484 | 1 | 0.502177 | -3.77795 |
| Prpf38b | 0.002738 | 0.220612 | 0.634 | 0.406 | 1 | 1.246839 | 1.210014 |
| Ppp3ca | 0.002761 | 0.276649 | 0.701 | 0.516 | 1 | 1.318704 | 1.517369 |
| Setd2 | 0.002775 | 0.194868 | 0.491 | 0.297 | 1 | 1.215151 | 1.068817 |
| Sh3pxd2a | 0.002812 | 0.238386 | 0.195 | 0.078 | 1 | 1.269199 | 1.307501 |
| Arhgap17 | 0.002858 | 0.204643 | 0.444 | 0.25 | 1 | 1.227087 | 1.122428 |
| Gpx4 | 0.002908 | -0.42231 | 0.403 | 0.453 | 1 | 0.655532 | -2.31628 |
| Xylt1 | 0.002927 | 0.209564 | 0.273 | 0.125 | 1 | 1.23314 | 1.149421 |
| 1700020I14Rik | 0.002947 | 0.192835 | 0.366 | 0.203 | 1 | 1.212683 | 1.057664 |
| Cic | 0.002949 | 0.191121 | 0.208 | 0.086 | 1 | 1.210606 | 1.048263 |
| Rpia | 0.00297 | 0.222629 | 0.182 | 0.07 | 1 | 1.249356 | 1.221076 |
| Mat2a | 0.002972 | 0.223328 | 0.501 | 0.312 | 1 | 1.25023 | 1.224912 |
| Tecpr1 | 0.002977 | 0.18317 | 0.177 | 0.062 | 1 | 1.201019 | 1.004655 |
| Tnks | 0.002984 | 0.183486 | 0.125 | 0.031 | 1 | 1.201399 | 1.006389 |
| 5031439G07Rik | 0.002989 | 0.219933 | 0.184 | 0.07 | 1 | 1.245993 | 1.206293 |
| Ensa | 0.002995 | 0.198321 | 0.306 | 0.156 | 1 | 1.219354 | 1.087757 |
| Ewsr1 | 0.002997 | 0.187983 | 0.392 | 0.227 | 1 | 1.206813 | 1.031052 |
| Gramd3 | 0.003059 | 0.193562 | 0.408 | 0.234 | 1 | 1.213565 | 1.061653 |
| Gm20696 | 0.003094 | 0.196245 | 0.265 | 0.125 | 1 | 1.216826 | 1.07637 |
| Agfg1 | 0.003103 | 0.189293 | 0.229 | 0.102 | 1 | 1.208395 | 1.038237 |
| Depdc5 | 0.003106 | 0.215107 | 0.135 | 0.039 | 1 | 1.239995 | 1.179823 |
| Eloa | 0.003113 | 0.18903 | 0.236 | 0.102 | 1 | 1.208077 | 1.036795 |
| Rpl10-ps3 | 0.003146 | -0.41135 | 0.397 | 0.438 | 1 | 0.662753 | -2.2562 |
| Zfp280d | 0.003216 | 0.224439 | 0.236 | 0.109 | 1 | 1.25162 | 1.231005 |
| Sec61a1 | 0.003226 | 0.184404 | 0.247 | 0.117 | 1 | 1.202501 | 1.011419 |
| Chd8 | 0.003242 | 0.275839 | 0.343 | 0.188 | 1 | 1.317636 | 1.512929 |
| Klf3 | 0.003258 | 0.33058 | 0.405 | 0.25 | 1 | 1.391776 | 1.813172 |
| Jarid2 | 0.003262 | 0.19083 | 0.296 | 0.148 | 1 | 1.210254 | 1.046668 |
| Abl2 | 0.003263 | 0.190697 | 0.125 | 0.031 | 1 | 1.210093 | 1.04594 |
| Akap11 | 0.003333 | 0.226607 | 0.132 | 0.039 | 1 | 1.254337 | 1.242897 |
| Nap1l1 | 0.00334 | 0.2038 | 0.709 | 0.484 | 1 | 1.226052 | 1.117803 |
| Mast3 | 0.003342 | 0.197114 | 0.135 | 0.039 | 1 | 1.217883 | 1.081134 |
| Usp9x | 0.003369 | 0.251396 | 0.41 | 0.234 | 1 | 1.285819 | 1.378858 |
| Ptp4a3 | 0.003371 | 0.201196 | 0.535 | 0.328 | 1 | 1.222865 | 1.103526 |
| Ifi47 | 0.003383 | -0.29832 | 0.042 | 0.109 | 1 | 0.742067 | -1.63621 |
| Cnr2 | 0.00342 | 0.192874 | 0.106 | 0.023 | 1 | 1.212731 | 1.057881 |
| Fyb | 0.003452 | -0.21945 | 0.036 | 0.102 | 1 | 0.802957 | -1.20367 |
| Rabgap1 | 0.003464 | 0.230326 | 0.169 | 0.062 | 1 | 1.25901 | 1.263293 |
| Derl1 | 0.003469 | 0.192791 | 0.208 | 0.086 | 1 | 1.212629 | 1.057423 |
| Satb1 | 0.003473 | 0.295907 | 0.761 | 0.625 | 1 | 1.344345 | 1.622996 |
| Ldha | 0.003475 | -0.40839 | 0.343 | 0.398 | 1 | 0.66472 | -2.23994 |
| Reps1 | 0.003563 | 0.18723 | 0.122 | 0.031 | 1 | 1.205905 | 1.026923 |
| Eif4g1 | 0.003588 | 0.185676 | 0.351 | 0.188 | 1 | 1.204032 | 1.018399 |
| Dad1 | 0.003596 | -0.31612 | 0.688 | 0.656 | 1 | 0.728974 | -1.73384 |
| Tma7 | 0.003602 | -0.23817 | 0.681 | 0.688 | 1 | 0.788069 | -1.30632 |
| Sike1 | 0.003754 | 0.194761 | 0.179 | 0.07 | 1 | 1.21502 | 1.068227 |
| Ikbkb | 0.003773 | 0.194451 | 0.504 | 0.305 | 1 | 1.214644 | 1.06653 |
| Sell | 0.003815 | 0.217083 | 0.668 | 0.461 | 1 | 1.242448 | 1.190662 |
| Cxxc5 | 0.003829 | 0.221971 | 0.229 | 0.102 | 1 | 1.248536 | 1.217471 |
| Celf2 | 0.003849 | 0.196229 | 0.657 | 0.453 | 1 | 1.216806 | 1.07628 |
| Retreg1 | 0.003854 | 0.188359 | 0.379 | 0.211 | 1 | 1.207266 | 1.033112 |
| Sub1 | 0.004039 | -0.26973 | 0.844 | 0.812 | 1 | 0.763584 | -1.47943 |
| Kmo | 0.004058 | 0.20227 | 0.119 | 0.031 | 1 | 1.224179 | 1.109414 |
| Usp53 | 0.004145 | 0.193868 | 0.119 | 0.031 | 1 | 1.213936 | 1.063328 |
| Pgls | 0.004168 | -0.30689 | 0.481 | 0.516 | 1 | 0.735731 | -1.68324 |
| R3hdm1 | 0.004182 | 0.229348 | 0.421 | 0.25 | 1 | 1.257779 | 1.257929 |
| Kmt2a | 0.004207 | 0.194286 | 0.657 | 0.438 | 1 | 1.214444 | 1.065623 |
| Txnip | 0.004226 | 0.285182 | 0.509 | 0.32 | 1 | 1.330004 | 1.564172 |
| Plekhm3 | 0.004249 | 0.19221 | 0.226 | 0.102 | 1 | 1.211926 | 1.054239 |
| Trim56 | 0.004335 | 0.223638 | 0.184 | 0.078 | 1 | 1.250618 | 1.226613 |
| Ddx3y | 0.004375 | 0.212314 | 0.382 | 0.219 | 1 | 1.236536 | 1.164503 |
| Fos | 0.00447 | -0.38069 | 0.556 | 0.602 | 1 | 0.683388 | -2.08803 |
| Krit1 | 0.004472 | 0.231965 | 0.429 | 0.25 | 1 | 1.261076 | 1.272287 |
| Mier3 | 0.004472 | 0.191009 | 0.13 | 0.039 | 1 | 1.210471 | 1.047651 |
| Malt1 | 0.004488 | 0.261519 | 0.655 | 0.469 | 1 | 1.298901 | 1.434381 |
| Gtf3c1 | 0.004544 | 0.211465 | 0.13 | 0.039 | 1 | 1.235487 | 1.159846 |
| Rell1 | 0.004606 | 0.187245 | 0.195 | 0.078 | 1 | 1.205922 | 1.027002 |
| Ablim1 | 0.004623 | 0.202738 | 0.634 | 0.406 | 1 | 1.224751 | 1.111978 |
| Neil1 | 0.004642 | 0.198228 | 0.143 | 0.047 | 1 | 1.21924 | 1.087244 |
| Dcaf12 | 0.004739 | 0.188263 | 0.179 | 0.07 | 1 | 1.207151 | 1.032587 |
| Plk2 | 0.004832 | 0.277716 | 0.223 | 0.102 | 1 | 1.320111 | 1.52322 |
| Ucp2 | 0.004835 | 0.240911 | 0.634 | 0.43 | 1 | 1.272408 | 1.321353 |
| Kdm6a | 0.004838 | 0.199269 | 0.164 | 0.062 | 1 | 1.22051 | 1.092953 |
| Csrp2 | 0.004902 | 0.189225 | 0.119 | 0.031 | 1 | 1.208313 | 1.037866 |
| Irf2bp2 | 0.004937 | 0.241827 | 0.286 | 0.148 | 1 | 1.273574 | 1.326378 |
| Ubtf | 0.004952 | 0.229145 | 0.319 | 0.18 | 1 | 1.257524 | 1.256816 |
| Cpsf7 | 0.004969 | 0.182826 | 0.223 | 0.102 | 1 | 1.200605 | 1.002767 |
| Snrpe | 0.0051 | -0.32938 | 0.595 | 0.586 | 1 | 0.719367 | -1.80661 |
| Ppfia1 | 0.0051 | 0.201576 | 0.2 | 0.086 | 1 | 1.223329 | 1.105607 |
| Setd5 | 0.00516 | 0.191977 | 0.358 | 0.203 | 1 | 1.211643 | 1.052959 |
| Slc31a1 | 0.005261 | -0.27276 | 0.073 | 0.148 | 1 | 0.761277 | -1.49603 |
| Epb41 | 0.005311 | 0.222063 | 0.338 | 0.195 | 1 | 1.24865 | 1.217974 |
| Zfp292 | 0.00532 | 0.201142 | 0.475 | 0.289 | 1 | 1.222798 | 1.103226 |
| Dhx40 | 0.005347 | 0.197869 | 0.273 | 0.141 | 1 | 1.218802 | 1.085272 |
| Copb1 | 0.005415 | 0.189015 | 0.319 | 0.172 | 1 | 1.20806 | 1.036715 |
| Cacna1i | 0.005498 | 0.190915 | 0.127 | 0.039 | 1 | 1.210357 | 1.047135 |
| Ssr4 | 0.005505 | -0.33302 | 0.361 | 0.406 | 1 | 0.716752 | -1.82658 |
| Jak2 | 0.005567 | 0.225818 | 0.369 | 0.219 | 1 | 1.253348 | 1.23857 |
| Gimap9 | 0.005605 | -0.4769 | 0.177 | 0.258 | 1 | 0.620703 | -2.61572 |
| Nme1 | 0.005644 | -0.36555 | 0.317 | 0.391 | 1 | 0.693815 | -2.00498 |
| Arih2 | 0.005674 | 0.188782 | 0.21 | 0.094 | 1 | 1.207777 | 1.035433 |
| Cenpx | 0.005751 | -0.34557 | 0.148 | 0.234 | 1 | 0.70782 | -1.89536 |
| Timm8a1 | 0.00577 | -0.23418 | 0.049 | 0.117 | 1 | 0.791219 | -1.28444 |
| Trp53inp2 | 0.005904 | 0.196051 | 0.127 | 0.039 | 1 | 1.216589 | 1.075305 |
| Pgm2l1 | 0.006134 | 0.186152 | 0.14 | 0.047 | 1 | 1.204606 | 1.021012 |
| Nxf1 | 0.006166 | 0.182938 | 0.338 | 0.195 | 1 | 1.20074 | 1.003383 |
| Gbp7 | 0.006237 | -0.22416 | 0.049 | 0.117 | 1 | 0.799188 | -1.22947 |
| Zrsr1 | 0.006267 | -0.20198 | 0.049 | 0.117 | 1 | 0.817113 | -1.10781 |
| Rassf4 | 0.006344 | 0.191315 | 0.112 | 0.031 | 1 | 1.210841 | 1.049326 |
| Atp6v1f | 0.006361 | -0.34579 | 0.532 | 0.539 | 1 | 0.707662 | -1.89658 |
| Xrn2 | 0.006531 | 0.211715 | 0.483 | 0.305 | 1 | 1.235795 | 1.161216 |
| Stat4 | 0.006592 | 0.195944 | 0.512 | 0.328 | 1 | 1.216458 | 1.074714 |
| Mpeg1 | 0.006666 | -0.28881 | 0.075 | 0.148 | 1 | 0.749154 | -1.58408 |
| Mrpl23 | 0.006668 | -0.29959 | 0.306 | 0.391 | 1 | 0.741124 | -1.64318 |
| Psmb1 | 0.006748 | -0.28675 | 0.613 | 0.602 | 1 | 0.750702 | -1.57275 |
| Pfdn5 | 0.00685 | -0.25669 | 0.862 | 0.828 | 1 | 0.77361 | -1.40788 |
| Phf2 | 0.007293 | 0.191975 | 0.138 | 0.047 | 1 | 1.211641 | 1.052949 |
| Zeb2 | 0.007356 | 0.209458 | 0.286 | 0.156 | 1 | 1.23301 | 1.148838 |
| Hsp90ab1 | 0.007394 | -0.22402 | 0.974 | 0.953 | 1 | 0.799299 | -1.22871 |
| Taok3 | 0.00752 | 0.190078 | 0.327 | 0.188 | 1 | 1.209344 | 1.042544 |
| Ctsb | 0.007578 | -0.3051 | 0.208 | 0.289 | 1 | 0.737052 | -1.6734 |
| Ywhaz | 0.007703 | 0.206065 | 0.732 | 0.508 | 1 | 1.228833 | 1.130226 |
| Ankle2 | 0.007757 | 0.18795 | 0.169 | 0.07 | 1 | 1.206774 | 1.030873 |
| Atp6ap1 | 0.00798 | 0.182551 | 0.265 | 0.141 | 1 | 1.200275 | 1.001257 |
| Cox5b | 0.008303 | -0.32459 | 0.506 | 0.508 | 1 | 0.72282 | -1.78034 |
| Sec24a | 0.00832 | 0.184242 | 0.169 | 0.07 | 1 | 1.202307 | 1.010533 |
| Cd24a | 0.008386 | 0.227425 | 0.514 | 0.336 | 1 | 1.255363 | 1.247384 |
| Lyst | 0.008459 | 0.199222 | 0.382 | 0.234 | 1 | 1.220453 | 1.092698 |
| Rnf157 | 0.008635 | 0.196772 | 0.117 | 0.039 | 1 | 1.217466 | 1.079257 |
| Arhgap31 | 0.008644 | 0.199203 | 0.187 | 0.086 | 1 | 1.22043 | 1.092591 |
| Ppp3cb | 0.008697 | 0.211396 | 0.229 | 0.117 | 1 | 1.235402 | 1.15947 |
| Tmf1 | 0.008763 | 0.196934 | 0.281 | 0.156 | 1 | 1.217663 | 1.080145 |
| Aldoa | 0.008898 | -0.30802 | 0.499 | 0.523 | 1 | 0.734901 | -1.68943 |
| Pnrc1 | 0.009072 | -0.27522 | 0.891 | 0.836 | 1 | 0.759405 | -1.50953 |
| Cd37 | 0.009136 | -0.19371 | 0.899 | 0.836 | 1 | 0.823894 | -1.06248 |
| Sumo2 | 0.009278 | -0.31049 | 0.59 | 0.578 | 1 | 0.733088 | -1.70298 |
| Irs2 | 0.009426 | 0.21709 | 0.387 | 0.234 | 1 | 1.242456 | 1.190699 |
| Mllt6 | 0.009582 | 0.185326 | 0.213 | 0.102 | 1 | 1.203611 | 1.016479 |
| Ptms | 0.009856 | 0.191412 | 0.161 | 0.07 | 1 | 1.210958 | 1.049857 |
| Polr3k | 0.010016 | -0.27252 | 0.078 | 0.148 | 1 | 0.761455 | -1.49475 |
| Cst3 | 0.010036 | -0.28813 | 0.54 | 0.562 | 1 | 0.749663 | -1.58035 |
| Cd274 | 0.010049 | -0.3651 | 0.086 | 0.156 | 1 | 0.694127 | -2.00251 |
| Fam193b | 0.010569 | 0.185736 | 0.117 | 0.039 | 1 | 1.204104 | 1.018728 |
| Yae1d1 | 0.010921 | -0.22103 | 0.049 | 0.109 | 1 | 0.801691 | -1.21232 |
| Ly86 | 0.010942 | -0.30406 | 0.66 | 0.625 | 1 | 0.73782 | -1.66769 |
| Cox4i1 | 0.01127 | -0.23216 | 0.839 | 0.82 | 1 | 0.792817 | -1.27337 |
| H2afz | 0.011491 | -0.31092 | 0.636 | 0.609 | 1 | 0.732771 | -1.70535 |
| Phf20l1 | 0.011838 | 0.19149 | 0.444 | 0.266 | 1 | 1.211053 | 1.050286 |
| Gbf1 | 0.012108 | 0.210699 | 0.145 | 0.062 | 1 | 1.234541 | 1.155645 |
| Cox8a | 0.012473 | -0.21289 | 0.795 | 0.75 | 1 | 0.808244 | -1.16767 |
| Cd69 | 0.013045 | -0.3325 | 0.626 | 0.641 | 1 | 0.717125 | -1.82373 |
| Crip1 | 0.013303 | 0.473786 | 0.636 | 0.492 | 1 | 1.606063 | 2.598627 |
| Irf1 | 0.013555 | -0.35271 | 0.301 | 0.375 | 1 | 0.702783 | -1.93453 |
| Ctsd | 0.013658 | -0.33281 | 0.208 | 0.289 | 1 | 0.716905 | -1.82541 |
| Ptpn22 | 0.014526 | -0.32718 | 0.288 | 0.375 | 1 | 0.720952 | -1.79453 |
| Itpr2 | 0.014799 | 0.196293 | 0.356 | 0.211 | 1 | 1.216884 | 1.076633 |
| Gnpnat1 | 0.014827 | -0.23047 | 0.052 | 0.109 | 1 | 0.794158 | -1.2641 |
| Uqcrfs1 | 0.014976 | -0.34101 | 0.283 | 0.344 | 1 | 0.711049 | -1.8704 |
| Pop5 | 0.015012 | -0.30692 | 0.166 | 0.242 | 1 | 0.735706 | -1.68343 |
| Hspe1 | 0.016164 | -0.27831 | 0.704 | 0.656 | 1 | 0.757064 | -1.52647 |
| Ubl5 | 0.016473 | -0.21829 | 0.53 | 0.547 | 1 | 0.803893 | -1.19728 |
| Mcts1 | 0.016792 | -0.2548 | 0.088 | 0.156 | 1 | 0.77507 | -1.39754 |
| Rpl36al | 0.01681 | -0.20054 | 0.808 | 0.773 | 1 | 0.818285 | -1.09995 |
| Tmbim4 | 0.016828 | -0.35192 | 0.166 | 0.242 | 1 | 0.703339 | -1.9302 |
| Anp32b | 0.01695 | -0.40241 | 0.496 | 0.5 | 1 | 0.668703 | -2.20717 |
| Chd6 | 0.017409 | 0.200571 | 0.371 | 0.227 | 1 | 1.2221 | 1.100094 |
| Spib | 0.017481 | 0.244775 | 0.434 | 0.281 | 1 | 1.277334 | 1.342546 |
| Pcbp2 | 0.017793 | 0.202731 | 0.652 | 0.477 | 1 | 1.224743 | 1.111942 |
| Rasgef1b | 0.018057 | 0.203371 | 0.273 | 0.156 | 1 | 1.225527 | 1.115453 |
| Ctsz | 0.018273 | -0.27853 | 0.564 | 0.562 | 1 | 0.756898 | -1.52767 |
| Gapdh | 0.018663 | -0.23349 | 0.779 | 0.727 | 1 | 0.791763 | -1.28067 |
| Scand1 | 0.019335 | -0.32293 | 0.478 | 0.492 | 1 | 0.724028 | -1.77118 |
| Pla2g16 | 0.021053 | -0.27433 | 0.086 | 0.148 | 1 | 0.760081 | -1.50465 |
| Uqcrh | 0.021398 | -0.19158 | 0.86 | 0.812 | 1 | 0.825653 | -1.05078 |
| Tifa | 0.02165 | 0.210266 | 0.106 | 0.039 | 1 | 1.234006 | 1.153271 |
| Gpx1 | 0.021937 | -0.24128 | 0.384 | 0.445 | 1 | 0.785621 | -1.32338 |
| Ndufa4 | 0.022647 | -0.1873 | 0.499 | 0.508 | 1 | 0.829194 | -1.02731 |
| Eif3i | 0.02285 | -0.29928 | 0.481 | 0.484 | 1 | 0.741353 | -1.64149 |
| Rps6ka5 | 0.022854 | 0.18973 | 0.158 | 0.078 | 1 | 1.208923 | 1.040634 |
| Mrpl35 | 0.023772 | -0.22794 | 0.078 | 0.141 | 1 | 0.796174 | -1.2502 |
| Cdkn1a | 0.025074 | -0.24017 | 0.068 | 0.125 | 1 | 0.786491 | -1.31731 |
| Brd8 | 0.025176 | 0.197088 | 0.325 | 0.203 | 1 | 1.217851 | 1.080989 |
| Id2 | 0.025263 | -0.3223 | 0.068 | 0.125 | 1 | 0.724478 | -1.76778 |
| Timm10b | 0.025647 | -0.2831 | 0.21 | 0.281 | 1 | 0.753442 | -1.55277 |
| Txn2 | 0.026357 | -0.33526 | 0.177 | 0.234 | 1 | 0.715154 | -1.83882 |
| H2-Q6 | 0.026394 | 0.240374 | 0.486 | 0.359 | 1 | 1.271725 | 1.318408 |
| Ndufa2 | 0.026494 | -0.25561 | 0.561 | 0.539 | 1 | 0.774443 | -1.40198 |
| Fam174a | 0.027237 | -0.2847 | 0.158 | 0.227 | 1 | 0.752243 | -1.5615 |
| Ino80b | 0.029373 | -0.26541 | 0.081 | 0.141 | 1 | 0.766894 | -1.4557 |
| Myl12b | 0.02968 | -0.28798 | 0.569 | 0.555 | 1 | 0.749776 | -1.57952 |
| Plek | 0.029684 | -0.34695 | 0.312 | 0.359 | 1 | 0.706839 | -1.90297 |
| Cd48 | 0.030424 | -0.29428 | 0.223 | 0.281 | 1 | 0.745069 | -1.61406 |
| Atg101 | 0.030625 | -0.31026 | 0.221 | 0.281 | 1 | 0.73326 | -1.70169 |
| P2rx4 | 0.03131 | 0.211641 | 0.283 | 0.18 | 1 | 1.235705 | 1.160814 |
| Selenok | 0.031803 | -0.24296 | 0.499 | 0.492 | 1 | 0.784299 | -1.33262 |
| Nedd8 | 0.031949 | -0.24706 | 0.436 | 0.453 | 1 | 0.781091 | -1.3551 |
| Nmi | 0.032345 | -0.29679 | 0.135 | 0.195 | 1 | 0.743203 | -1.62782 |
| Gtf2h5 | 0.033461 | -0.335 | 0.203 | 0.266 | 1 | 0.715335 | -1.83744 |
| Anapc16 | 0.033472 | -0.35727 | 0.265 | 0.312 | 1 | 0.699581 | -1.95958 |
| Bax | 0.033517 | -0.3744 | 0.286 | 0.328 | 1 | 0.687698 | -2.05354 |
| Ap4s1 | 0.034004 | -0.21448 | 0.088 | 0.148 | 1 | 0.806961 | -1.17638 |
| Bfsp2 | 0.034399 | -0.33487 | 0.145 | 0.211 | 1 | 0.715431 | -1.8367 |
| Ctsl | 0.037201 | -0.24029 | 0.057 | 0.109 | 1 | 0.786403 | -1.31792 |
| Coq10b | 0.038258 | -0.30892 | 0.41 | 0.438 | 1 | 0.734242 | -1.69435 |
| Pold4 | 0.038453 | -0.25628 | 0.584 | 0.562 | 1 | 0.773922 | -1.40567 |
| Ndufb10 | 0.039715 | -0.27787 | 0.418 | 0.414 | 1 | 0.757393 | -1.52408 |
| Cd52 | 0.040106 | -0.20885 | 0.945 | 0.945 | 1 | 0.811519 | -1.14549 |
| Npm1 | 0.041501 | -0.18679 | 0.891 | 0.812 | 1 | 0.829619 | -1.0245 |
| Eif3h | 0.041724 | -0.25175 | 0.683 | 0.641 | 1 | 0.777438 | -1.38081 |
| Eif5a | 0.042914 | -0.24847 | 0.61 | 0.57 | 1 | 0.779992 | -1.36282 |
| Clic1 | 0.043198 | -0.27487 | 0.675 | 0.609 | 1 | 0.759673 | -1.5076 |
| Mxd1 | 0.043229 | -0.33679 | 0.13 | 0.188 | 1 | 0.714058 | -1.84724 |
| 2810474O19Rik | 0.043462 | -0.23898 | 0.281 | 0.344 | 1 | 0.787428 | -1.31078 |
| BC031181 | 0.044607 | -0.31281 | 0.156 | 0.211 | 1 | 0.731389 | -1.71571 |
| Sdhaf1 | 0.047374 | -0.34535 | 0.14 | 0.195 | 1 | 0.707975 | -1.89416 |

**Table S2 Results of differential gene expression analysis at 3 days after LPS and LPS/MSC treatment**

| gene | p_val | avg_logFC | pct.1 | pct.2 | p_val_adj | Foldchange | log1.2FC |
| --- | --- | --- | --- | --- | --- | --- | --- |
| Rps29 | 3.69E-22 | -0.354 | 0.992 | 0.996 | 1.15E-17 | 0.701876 | -1.94161 |
| Rpl38 | 1.86E-16 | -0.40151 | 0.992 | 1 | 5.78E-12 | 0.669311 | -2.20219 |
| Rpl35 | 2.26E-15 | -0.46032 | 0.969 | 0.976 | 7.01E-11 | 0.631081 | -2.52478 |
| Rps27 | 3.53E-13 | -0.28055 | 0.992 | 1 | 1.10E-08 | 0.755368 | -1.53876 |
| Rpl37 | 4.00E-11 | -0.28785 | 1 | 0.996 | 1.24E-06 | 0.749871 | -1.57883 |
| Rps21 | 2.40E-10 | -0.28505 | 0.992 | 1 | 7.45E-06 | 0.751977 | -1.56345 |
| Rpl37a | 1.13E-09 | -0.24709 | 0.992 | 1 | 3.50E-05 | 0.781072 | -1.35523 |
| mt-Co1 | 7.04E-09 | -0.21087 | 1 | 1 | 0.000219 | 0.809882 | -1.15657 |
| Rpl36 | 4.93E-08 | -0.23418 | 0.984 | 0.988 | 0.001531 | 0.791221 | -1.28442 |
| Uba52 | 1.01E-07 | -0.39165 | 0.758 | 0.85 | 0.003132 | 0.675943 | -2.14811 |
| Gm10076 | 1.31E-05 | -0.50838 | 0.117 | 0.313 | 0.406538 | 0.601471 | -2.78835 |
| Rps28 | 1.37E-05 | -0.19679 | 0.977 | 0.992 | 0.426756 | 0.821361 | -1.07937 |
| Gm42418 | 2.27E-05 | 0.202287 | 1 | 1 | 0.704908 | 1.224199 | 1.109505 |
| Rsrp1 | 4.79E-05 | 0.414606 | 0.781 | 0.65 | 1 | 1.513775 | 2.274039 |
| mt-Nd3 | 5.40E-05 | -0.33591 | 0.758 | 0.866 | 1 | 0.714688 | -1.8424 |
| Cxcr4 | 6.45E-05 | 0.394256 | 0.625 | 0.382 | 1 | 1.48328 | 2.162421 |
| Icam1 | 0.000198 | 0.382512 | 0.281 | 0.126 | 1 | 1.465962 | 2.098005 |
| Sgta | 0.0002 | 0.380576 | 0.141 | 0.037 | 1 | 1.463127 | 2.087387 |
| Ppa2 | 0.000226 | 0.256895 | 0.133 | 0.033 | 1 | 1.29291 | 1.409023 |
| Man2a2 | 0.000242 | 0.254539 | 0.117 | 0.024 | 1 | 1.289867 | 1.396098 |
| Yae1d1 | 0.000245 | 0.241822 | 0.109 | 0.02 | 1 | 1.273567 | 1.326347 |
| Slc31a1 | 0.000253 | 0.276392 | 0.148 | 0.041 | 1 | 1.318364 | 1.515957 |
| Zrsr1 | 0.000255 | 0.226569 | 0.117 | 0.024 | 1 | 1.25429 | 1.242691 |
| Dnaja1 | 0.000297 | 0.373212 | 0.773 | 0.622 | 1 | 1.452392 | 2.046996 |
| Setd2 | 0.000498 | 0.335761 | 0.297 | 0.142 | 1 | 1.399005 | 1.841587 |
| Lars2 | 0.000501 | 0.309772 | 0.883 | 0.744 | 1 | 1.363115 | 1.699044 |
| Rpl10-ps3 | 0.000568 | 0.384715 | 0.438 | 0.276 | 1 | 1.469195 | 2.11009 |
| Stxbp3 | 0.000586 | 0.263301 | 0.156 | 0.049 | 1 | 1.301218 | 1.444158 |
| Nup210 | 0.000634 | 0.219764 | 0.141 | 0.041 | 1 | 1.245783 | 1.205365 |
| Ssh2 | 0.000658 | 0.30071 | 0.672 | 0.516 | 1 | 1.350818 | 1.649341 |
| Rpl27 | 0.000702 | 0.187754 | 0.984 | 0.963 | 1 | 1.206537 | 1.029797 |
| Scaper | 0.000716 | 0.199949 | 0.141 | 0.041 | 1 | 1.22134 | 1.096682 |
| Bambi | 0.000901 | 0.190705 | 0.148 | 0.049 | 1 | 1.210103 | 1.045983 |
| Papd4 | 0.001029 | 0.263153 | 0.133 | 0.041 | 1 | 1.301026 | 1.443346 |
| Bst2 | 0.001277 | 0.36631 | 0.289 | 0.154 | 1 | 1.442403 | 2.009144 |
| Gm31243 | 0.0013 | -0.33034 | 0.445 | 0.589 | 1 | 0.718681 | -1.81184 |
| Tmx4 | 0.001338 | 0.239798 | 0.109 | 0.028 | 1 | 1.270993 | 1.31525 |
| Wdr89 | 0.001357 | 0.285891 | 0.648 | 0.484 | 1 | 1.330948 | 1.568061 |
| Mpc1 | 0.001545 | 0.282749 | 0.359 | 0.203 | 1 | 1.326772 | 1.550824 |
| Crip1 | 0.00186 | -0.37785 | 0.492 | 0.614 | 1 | 0.685331 | -2.07246 |
| Tab2 | 0.001881 | 0.346998 | 0.227 | 0.11 | 1 | 1.414814 | 1.90322 |
| Nr1h2 | 0.001885 | 0.289224 | 0.188 | 0.077 | 1 | 1.335391 | 1.586343 |
| Mxd1 | 0.001912 | 0.339996 | 0.188 | 0.081 | 1 | 1.404942 | 1.864817 |
| Rps27rt | 0.001967 | 0.247269 | 0.906 | 0.772 | 1 | 1.280523 | 1.356222 |
| Ndufb8 | 0.001976 | 0.276725 | 0.398 | 0.24 | 1 | 1.318804 | 1.517788 |
| Unc50 | 0.002144 | 0.251266 | 0.211 | 0.093 | 1 | 1.285652 | 1.378145 |
| Nr3c1 | 0.002329 | 0.302438 | 0.312 | 0.171 | 1 | 1.353154 | 1.658816 |
| Chd7 | 0.002448 | 0.232054 | 0.219 | 0.102 | 1 | 1.261188 | 1.272775 |
| Trabd | 0.002821 | 0.240334 | 0.18 | 0.077 | 1 | 1.271673 | 1.318185 |
| Ccl4 | 0.002839 | 0.437921 | 0.422 | 0.268 | 1 | 1.549483 | 2.401917 |
| Abce1 | 0.002899 | -0.37821 | 0.078 | 0.191 | 1 | 0.685083 | -2.07444 |
| Rabep2 | 0.003034 | 0.327485 | 0.188 | 0.085 | 1 | 1.387475 | 1.796197 |
| Ddx3x | 0.003126 | 0.279297 | 0.414 | 0.256 | 1 | 1.3222 | 1.531893 |
| Brpf1 | 0.003171 | 0.199161 | 0.125 | 0.041 | 1 | 1.220379 | 1.092364 |
| Rapgef6 | 0.003403 | 0.19779 | 0.445 | 0.272 | 1 | 1.218706 | 1.084842 |
| Retreg3 | 0.003495 | 0.299917 | 0.156 | 0.065 | 1 | 1.349746 | 1.644987 |
| Arih1 | 0.003537 | 0.21828 | 0.258 | 0.13 | 1 | 1.243935 | 1.197224 |
| Igkc | 0.00354 | -0.21341 | 0.945 | 0.976 | 1 | 0.807827 | -1.1705 |
| Ddx25 | 0.003583 | 0.262695 | 0.141 | 0.053 | 1 | 1.30043 | 1.440831 |
| Ubn1 | 0.003585 | 0.244734 | 0.25 | 0.126 | 1 | 1.277281 | 1.342318 |
| Rbbp6 | 0.003586 | 0.314437 | 0.453 | 0.305 | 1 | 1.369488 | 1.724629 |
| Sla | 0.003718 | 0.283291 | 0.289 | 0.154 | 1 | 1.327491 | 1.553799 |
| Mrps15 | 0.003746 | 0.288294 | 0.156 | 0.065 | 1 | 1.334149 | 1.581237 |
| Tap1 | 0.003751 | 0.299055 | 0.266 | 0.142 | 1 | 1.348584 | 1.640264 |
| Gm26699 | 0.003862 | 0.2611 | 0.203 | 0.093 | 1 | 1.298357 | 1.432084 |
| S100a9 | 0.00391 | 0.239802 | 0.844 | 0.744 | 1 | 1.270997 | 1.315268 |
| Zfp36 | 0.003922 | 0.277431 | 0.672 | 0.476 | 1 | 1.319735 | 1.521658 |
| Ndfip2 | 0.004282 | 0.210626 | 0.203 | 0.093 | 1 | 1.234451 | 1.155246 |
| Lax1 | 0.004371 | 0.259052 | 0.125 | 0.045 | 1 | 1.295701 | 1.420851 |
| Eps15 | 0.004379 | 0.228782 | 0.164 | 0.069 | 1 | 1.257068 | 1.254827 |
| Brms1 | 0.004658 | 0.197251 | 0.125 | 0.045 | 1 | 1.218049 | 1.081884 |
| Prpf38a | 0.004726 | -0.34631 | 0.055 | 0.15 | 1 | 0.707292 | -1.89946 |
| Slc41a1 | 0.004787 | 0.202597 | 0.117 | 0.041 | 1 | 1.224579 | 1.111209 |
| Sptan1 | 0.004823 | 0.278143 | 0.211 | 0.106 | 1 | 1.320675 | 1.525561 |
| Elk4 | 0.004929 | 0.267075 | 0.258 | 0.138 | 1 | 1.306138 | 1.464855 |
| Mdm4 | 0.005078 | 0.232137 | 0.281 | 0.154 | 1 | 1.261292 | 1.273227 |
| Son | 0.005404 | -0.23788 | 0.727 | 0.817 | 1 | 0.788299 | -1.30471 |
| Smn1 | 0.005484 | -0.21481 | 0.023 | 0.106 | 1 | 0.806698 | -1.17817 |
| AW549877 | 0.005696 | 0.201029 | 0.141 | 0.057 | 1 | 1.222661 | 1.102609 |
| Cyp4f18 | 0.005753 | -0.36906 | 0.234 | 0.354 | 1 | 0.691383 | -2.02423 |
| Mga | 0.005984 | 0.319649 | 0.188 | 0.089 | 1 | 1.376644 | 1.753213 |
| Ost4 | 0.006152 | -0.34957 | 0.258 | 0.366 | 1 | 0.704992 | -1.91732 |
| Wnk1 | 0.006173 | 0.331954 | 0.57 | 0.415 | 1 | 1.393689 | 1.820706 |
| Tkt | 0.006192 | 0.275904 | 0.328 | 0.203 | 1 | 1.317722 | 1.513285 |
| N4bp2l2 | 0.006351 | 0.209448 | 0.406 | 0.252 | 1 | 1.232997 | 1.148782 |
| Rab18 | 0.006412 | 0.280876 | 0.195 | 0.098 | 1 | 1.324289 | 1.540552 |
| Cbx1 | 0.006426 | -0.35816 | 0.18 | 0.301 | 1 | 0.698962 | -1.96444 |
| Ssh1 | 0.006427 | 0.184829 | 0.102 | 0.033 | 1 | 1.203013 | 1.013754 |
| Hmgb2 | 0.006456 | -0.28635 | 0.383 | 0.512 | 1 | 0.751001 | -1.57057 |
| Rbmxl1 | 0.006533 | 0.21539 | 0.25 | 0.134 | 1 | 1.240345 | 1.181374 |
| Ilf3 | 0.00667 | 0.19832 | 0.148 | 0.061 | 1 | 1.219353 | 1.087749 |
| Cox5a | 0.006678 | 0.278096 | 0.531 | 0.402 | 1 | 1.320612 | 1.525303 |
| Eef2 | 0.006817 | 0.239208 | 0.852 | 0.776 | 1 | 1.270243 | 1.312014 |
| Cdip1 | 0.006898 | 0.193648 | 0.172 | 0.077 | 1 | 1.213669 | 1.062125 |
| Qrich1 | 0.007013 | 0.184176 | 0.141 | 0.057 | 1 | 1.202227 | 1.01017 |
| Hspa1a | 0.007166 | -0.64895 | 0.125 | 0.236 | 1 | 0.522595 | -3.55937 |
| Zfp622 | 0.007206 | 0.25812 | 0.297 | 0.171 | 1 | 1.294495 | 1.415742 |
| Mbd2 | 0.007809 | 0.270335 | 0.336 | 0.203 | 1 | 1.310403 | 1.482736 |
| Fndc3a | 0.007862 | 0.204654 | 0.164 | 0.073 | 1 | 1.227101 | 1.122492 |
| Tpp2 | 0.007939 | 0.267522 | 0.258 | 0.142 | 1 | 1.306723 | 1.46731 |
| Lamtor3 | 0.007991 | 0.190991 | 0.172 | 0.077 | 1 | 1.210449 | 1.047551 |
| Phc2 | 0.008007 | 0.304535 | 0.273 | 0.163 | 1 | 1.355995 | 1.670321 |
| Ddt | 0.008035 | 0.194024 | 0.195 | 0.093 | 1 | 1.214126 | 1.064187 |
| Sp1 | 0.008109 | 0.197189 | 0.133 | 0.053 | 1 | 1.217974 | 1.081544 |
| Arglu1 | 0.008157 | 0.303678 | 0.445 | 0.317 | 1 | 1.354833 | 1.665617 |
| Cd82 | 0.008411 | 0.193086 | 0.164 | 0.073 | 1 | 1.212987 | 1.059043 |
| Ppp1r14b | 0.008492 | 0.299406 | 0.227 | 0.126 | 1 | 1.349057 | 1.642185 |
| Bnip3l | 0.008808 | 0.235028 | 0.406 | 0.256 | 1 | 1.264945 | 1.289087 |
| Prrc2c | 0.009043 | 0.276848 | 0.641 | 0.541 | 1 | 1.318966 | 1.518461 |
| Rab24 | 0.009051 | 0.203938 | 0.203 | 0.102 | 1 | 1.226222 | 1.118562 |
| Mrpl20 | 0.009086 | 0.188675 | 0.273 | 0.154 | 1 | 1.207649 | 1.03485 |
| Nlrp3 | 0.009331 | 0.211545 | 0.117 | 0.045 | 1 | 1.235586 | 1.160287 |
| Bfsp2 | 0.009344 | 0.261702 | 0.211 | 0.11 | 1 | 1.299139 | 1.435385 |
| Psmd6 | 0.009404 | 0.24656 | 0.188 | 0.093 | 1 | 1.279616 | 1.352337 |
| Chd1 | 0.009537 | 0.251308 | 0.297 | 0.179 | 1 | 1.285706 | 1.37838 |
| Cnot6l | 0.009761 | 0.243926 | 0.328 | 0.191 | 1 | 1.276249 | 1.337887 |
| Cmip | 0.009889 | 0.186866 | 0.227 | 0.118 | 1 | 1.205466 | 1.024927 |
| Atg3 | 0.010075 | 0.228714 | 0.227 | 0.122 | 1 | 1.256983 | 1.254455 |
| Vav1 | 0.010338 | 0.22008 | 0.195 | 0.098 | 1 | 1.246176 | 1.207098 |
| Metap2 | 0.010372 | -0.3493 | 0.156 | 0.264 | 1 | 0.705185 | -1.91582 |
| Scml4 | 0.010477 | 0.221393 | 0.117 | 0.045 | 1 | 1.247814 | 1.214299 |
| Rras2 | 0.010842 | 0.231437 | 0.109 | 0.041 | 1 | 1.26041 | 1.269387 |
| Rbm38 | 0.011529 | 0.184445 | 0.219 | 0.114 | 1 | 1.202551 | 1.011647 |
| Cox7c | 0.011883 | -0.23493 | 0.578 | 0.654 | 1 | 0.790623 | -1.28857 |
| Pdcd4 | 0.012223 | -0.40443 | 0.375 | 0.451 | 1 | 0.667355 | -2.21824 |
| Napsa | 0.012352 | -0.22068 | 0.688 | 0.74 | 1 | 0.801971 | -1.21041 |
| Pabpc1 | 0.012619 | 0.204845 | 0.828 | 0.646 | 1 | 1.227335 | 1.123537 |
| Arhgap30 | 0.012804 | 0.207951 | 0.461 | 0.317 | 1 | 1.231153 | 1.140572 |
| Pou2f1 | 0.013092 | 0.195922 | 0.203 | 0.106 | 1 | 1.216432 | 1.074596 |
| Chchd2 | 0.013159 | 0.188516 | 0.82 | 0.764 | 1 | 1.207456 | 1.033975 |
| Tmod3 | 0.013451 | 0.209421 | 0.453 | 0.325 | 1 | 1.232964 | 1.148636 |
| Nadk | 0.013592 | 0.246608 | 0.156 | 0.077 | 1 | 1.279677 | 1.352598 |
| Rnaset2a | 0.013721 | 0.242392 | 0.281 | 0.171 | 1 | 1.274293 | 1.329474 |
| Fam174a | 0.014 | 0.19458 | 0.227 | 0.122 | 1 | 1.214801 | 1.067236 |
| Ak2 | 0.0141 | -0.27949 | 0.102 | 0.199 | 1 | 0.756167 | -1.53296 |
| Mgea5 | 0.01419 | 0.184392 | 0.164 | 0.077 | 1 | 1.202487 | 1.011357 |
| Cd274 | 0.014331 | 0.290638 | 0.156 | 0.077 | 1 | 1.337281 | 1.594096 |
| Zfp292 | 0.014545 | 0.226407 | 0.289 | 0.179 | 1 | 1.254086 | 1.241801 |
| Ube2s | 0.014944 | 0.250903 | 0.453 | 0.325 | 1 | 1.285186 | 1.376158 |
| Zfc3h1 | 0.01501 | 0.227609 | 0.117 | 0.049 | 1 | 1.255594 | 1.248392 |
| Epc1 | 0.01532 | 0.281416 | 0.211 | 0.118 | 1 | 1.325004 | 1.543513 |
| Med13l | 0.015473 | 0.212661 | 0.148 | 0.069 | 1 | 1.236965 | 1.166407 |
| Cep135 | 0.016415 | 0.198303 | 0.109 | 0.045 | 1 | 1.219332 | 1.087656 |
| Ubxn6 | 0.016648 | 0.242608 | 0.148 | 0.073 | 1 | 1.274569 | 1.33066 |
| Otulin | 0.016724 | -0.32909 | 0.141 | 0.24 | 1 | 0.719579 | -1.80499 |
| Srp72 | 0.016775 | 0.211439 | 0.25 | 0.146 | 1 | 1.235454 | 1.159703 |
| Prrc2b | 0.016851 | 0.215241 | 0.156 | 0.077 | 1 | 1.24016 | 1.180554 |
| Esyt1 | 0.017046 | 0.189197 | 0.164 | 0.081 | 1 | 1.208279 | 1.037711 |
| Hspd1 | 0.017079 | 0.252103 | 0.359 | 0.236 | 1 | 1.286728 | 1.382738 |
| Lsm4 | 0.01709 | -0.27543 | 0.266 | 0.362 | 1 | 0.759242 | -1.51071 |
| Uqcrfs1 | 0.017133 | 0.235566 | 0.344 | 0.228 | 1 | 1.265625 | 1.292037 |
| Acin1 | 0.017202 | 0.237399 | 0.438 | 0.313 | 1 | 1.267947 | 1.302092 |
| Ndufb10 | 0.017336 | 0.252763 | 0.414 | 0.305 | 1 | 1.287578 | 1.386359 |
| Il2rg | 0.017489 | -0.24561 | 0.359 | 0.459 | 1 | 0.782226 | -1.34714 |
| Ubr5 | 0.017584 | 0.234502 | 0.141 | 0.065 | 1 | 1.264279 | 1.286199 |
| Med21 | 0.018039 | 0.266953 | 0.172 | 0.089 | 1 | 1.30598 | 1.46419 |
| Ppm1m | 0.018269 | 0.194121 | 0.141 | 0.065 | 1 | 1.214243 | 1.064716 |
| Tagap | 0.01845 | 0.221183 | 0.133 | 0.061 | 1 | 1.247551 | 1.213146 |
| Psma1 | 0.018497 | 0.248636 | 0.375 | 0.248 | 1 | 1.282276 | 1.363725 |
| Ndufa3 | 0.019055 | -0.36683 | 0.234 | 0.325 | 1 | 0.692927 | -2.01199 |
| Cd84 | 0.019108 | 0.223928 | 0.234 | 0.13 | 1 | 1.250981 | 1.228202 |
| Csnk2a2 | 0.019178 | 0.196521 | 0.219 | 0.122 | 1 | 1.217161 | 1.077883 |
| Kdm5a | 0.019342 | 0.235256 | 0.312 | 0.203 | 1 | 1.265232 | 1.290334 |
| Ccl5 | 0.019705 | 0.221492 | 0.164 | 0.085 | 1 | 1.247937 | 1.214842 |
| Prpf31 | 0.020108 | 0.200052 | 0.148 | 0.073 | 1 | 1.221466 | 1.097248 |
| Stim1 | 0.020232 | -0.32725 | 0.07 | 0.146 | 1 | 0.720903 | -1.79491 |
| Gm10073 | 0.020825 | -0.29059 | 0.086 | 0.167 | 1 | 0.747822 | -1.59383 |
| Srrm2 | 0.020907 | 0.215713 | 0.719 | 0.606 | 1 | 1.240746 | 1.183146 |
| Mis12 | 0.020973 | 0.185633 | 0.102 | 0.041 | 1 | 1.20398 | 1.018162 |
| Ptpn6 | 0.020996 | 0.225363 | 0.523 | 0.394 | 1 | 1.252778 | 1.236075 |
| Nfkb1 | 0.021271 | 0.236097 | 0.328 | 0.211 | 1 | 1.266297 | 1.294946 |
| Trim12c | 0.02145 | 0.221324 | 0.219 | 0.126 | 1 | 1.247727 | 1.21392 |
| Phip | 0.022099 | 0.209555 | 0.383 | 0.264 | 1 | 1.233129 | 1.149368 |
| Mef2d | 0.022117 | 0.214606 | 0.242 | 0.146 | 1 | 1.239373 | 1.177073 |
| Cdc42se2 | 0.022522 | -0.31925 | 0.172 | 0.264 | 1 | 0.726694 | -1.75103 |
| Chd3 | 0.022608 | 0.265852 | 0.219 | 0.13 | 1 | 1.304542 | 1.45815 |
| Tle4 | 0.022648 | -0.25103 | 0.188 | 0.285 | 1 | 0.777997 | -1.37687 |
| Ctnnb1 | 0.022938 | 0.233854 | 0.219 | 0.13 | 1 | 1.26346 | 1.282646 |
| Acbd5 | 0.023286 | -0.19303 | 0.062 | 0.142 | 1 | 0.824455 | -1.05875 |
| Prpf8 | 0.023334 | 0.267414 | 0.227 | 0.134 | 1 | 1.306581 | 1.466715 |
| Gbp2 | 0.023482 | 0.278568 | 0.18 | 0.102 | 1 | 1.321236 | 1.527894 |
| Slc38a2 | 0.023672 | 0.224914 | 0.781 | 0.667 | 1 | 1.252215 | 1.233609 |
| Sp110 | 0.023985 | 0.184814 | 0.359 | 0.236 | 1 | 1.202994 | 1.013668 |
| Anp32b | 0.024163 | 0.269576 | 0.5 | 0.382 | 1 | 1.309409 | 1.478572 |
| 2610507B11Rik | 0.024225 | 0.19193 | 0.141 | 0.069 | 1 | 1.211586 | 1.052701 |
| H2-Q7 | 0.025307 | 0.188249 | 0.469 | 0.354 | 1 | 1.207134 | 1.03251 |
| Hist1h1e | 0.025497 | 0.240648 | 0.305 | 0.195 | 1 | 1.272073 | 1.31991 |
| Srpk3 | 0.025774 | -0.25292 | 0.047 | 0.118 | 1 | 0.776531 | -1.38721 |
| Polr2m | 0.025814 | 0.198067 | 0.227 | 0.134 | 1 | 1.219045 | 1.086363 |
| Gnpnat1 | 0.026024 | 0.184275 | 0.109 | 0.049 | 1 | 1.202346 | 1.010712 |
| Plin2 | 0.026269 | 0.222189 | 0.156 | 0.081 | 1 | 1.248808 | 1.218667 |
| Raly | 0.026414 | 0.217763 | 0.344 | 0.236 | 1 | 1.243292 | 1.194388 |
| Arid1b | 0.026781 | 0.209923 | 0.156 | 0.081 | 1 | 1.233583 | 1.151386 |
| Rubcn | 0.026892 | -0.24271 | 0.055 | 0.126 | 1 | 0.784501 | -1.3312 |
| Bag5 | 0.027015 | -0.18445 | 0.055 | 0.13 | 1 | 0.831563 | -1.01167 |
| Cntrl | 0.027058 | 0.2125 | 0.25 | 0.15 | 1 | 1.236766 | 1.165521 |
| Brd3 | 0.027539 | 0.198942 | 0.133 | 0.065 | 1 | 1.220112 | 1.091162 |
| Commd3 | 0.027698 | 0.238245 | 0.156 | 0.081 | 1 | 1.26902 | 1.306728 |
| Hadhb | 0.027758 | 0.184237 | 0.172 | 0.093 | 1 | 1.202301 | 1.010506 |
| Caap1 | 0.027794 | -0.26998 | 0.039 | 0.102 | 1 | 0.763395 | -1.48079 |
| Rab8b | 0.02792 | 0.188162 | 0.109 | 0.049 | 1 | 1.207029 | 1.032032 |
| Zfp1 | 0.027952 | -0.20292 | 0.039 | 0.106 | 1 | 0.816345 | -1.11297 |
| Snrnp200 | 0.027969 | 0.221775 | 0.164 | 0.089 | 1 | 1.24829 | 1.216394 |
| G3bp2 | 0.028607 | 0.198346 | 0.297 | 0.199 | 1 | 1.219385 | 1.087893 |
| Brox | 0.028726 | 0.188199 | 0.117 | 0.053 | 1 | 1.207073 | 1.032235 |
| Slfn2 | 0.029051 | 0.256699 | 0.594 | 0.48 | 1 | 1.292656 | 1.407947 |
| Surf4 | 0.029164 | 0.189303 | 0.133 | 0.065 | 1 | 1.208407 | 1.038291 |
| Cnot3 | 0.02919 | 0.212135 | 0.25 | 0.154 | 1 | 1.236315 | 1.163521 |
| Bod1l | 0.029353 | 0.223311 | 0.336 | 0.228 | 1 | 1.250209 | 1.224819 |
| Stag2 | 0.029604 | -0.35161 | 0.188 | 0.272 | 1 | 0.703552 | -1.92854 |
| Uqcr11 | 0.030082 | -0.26365 | 0.203 | 0.301 | 1 | 0.768241 | -1.44608 |
| Usp1 | 0.030524 | -0.24514 | 0.07 | 0.146 | 1 | 0.782596 | -1.34454 |
| Cul5 | 0.031351 | 0.203651 | 0.102 | 0.045 | 1 | 1.22587 | 1.116989 |
| Atp5e | 0.031494 | -0.19928 | 0.75 | 0.748 | 1 | 0.819317 | -1.09304 |
| Klf2 | 0.031609 | -0.265 | 0.586 | 0.659 | 1 | 0.767207 | -1.45347 |
| Cd55 | 0.031768 | -0.19459 | 0.43 | 0.537 | 1 | 0.823172 | -1.06729 |
| Eno1 | 0.031825 | -0.26391 | 0.188 | 0.276 | 1 | 0.768042 | -1.4475 |
| Ilf2 | 0.03217 | 0.209351 | 0.234 | 0.146 | 1 | 1.232878 | 1.148252 |
| Ly6d | 0.03228 | -0.21176 | 0.859 | 0.894 | 1 | 0.809156 | -1.16148 |
| Tob1 | 0.032875 | 0.196701 | 0.102 | 0.045 | 1 | 1.21738 | 1.078868 |
| Znrf2 | 0.032879 | 0.261091 | 0.188 | 0.11 | 1 | 1.298346 | 1.432036 |
| Sept11 | 0.033237 | 0.223566 | 0.156 | 0.085 | 1 | 1.250528 | 1.226217 |
| Rab2a | 0.033719 | 0.192139 | 0.336 | 0.224 | 1 | 1.211839 | 1.053847 |
| Bsdc1 | 0.034087 | -0.23991 | 0.055 | 0.122 | 1 | 0.786702 | -1.31584 |
| Atp5j2 | 0.034586 | -0.25544 | 0.375 | 0.447 | 1 | 0.774572 | -1.40106 |
| Jund | 0.034595 | -0.20324 | 0.875 | 0.939 | 1 | 0.816084 | -1.11472 |
| Ly86 | 0.035705 | 0.191895 | 0.625 | 0.516 | 1 | 1.211544 | 1.052511 |
| Ninj1 | 0.036939 | -0.25091 | 0.07 | 0.142 | 1 | 0.778093 | -1.37619 |
| Hs6st1 | 0.03759 | 0.192336 | 0.117 | 0.057 | 1 | 1.212078 | 1.054929 |
| 0610012G03Rik | 0.038198 | -0.19491 | 0.086 | 0.163 | 1 | 0.822905 | -1.06907 |
| Erbin | 0.038608 | 0.188473 | 0.172 | 0.098 | 1 | 1.207405 | 1.033741 |
| Gimap3 | 0.038667 | 0.221258 | 0.375 | 0.264 | 1 | 1.247645 | 1.213559 |
| Nfyb | 0.039364 | -0.25304 | 0.047 | 0.11 | 1 | 0.776438 | -1.38787 |
| Mirt1 | 0.039633 | -0.25416 | 0.109 | 0.191 | 1 | 0.775568 | -1.39402 |
| Bin2 | 0.040283 | -0.20762 | 0.094 | 0.171 | 1 | 0.812514 | -1.13877 |
| 1110038F14Rik | 0.040353 | -0.21018 | 0.047 | 0.11 | 1 | 0.81044 | -1.15279 |
| Srpk2 | 0.040444 | 0.25988 | 0.258 | 0.167 | 1 | 1.296774 | 1.425393 |
| Filip1l | 0.040477 | -0.23602 | 0.398 | 0.496 | 1 | 0.789764 | -1.29453 |
| mt-Nd4l | 0.040538 | -0.27555 | 0.391 | 0.467 | 1 | 0.759153 | -1.51135 |
| Flii | 0.040677 | -0.23998 | 0.055 | 0.118 | 1 | 0.786644 | -1.31625 |
| Hdac1 | 0.040757 | 0.209951 | 0.211 | 0.126 | 1 | 1.233618 | 1.151543 |
| Sars | 0.040822 | -0.34421 | 0.117 | 0.191 | 1 | 0.708782 | -1.88791 |
| Naa50 | 0.041472 | 0.195788 | 0.227 | 0.142 | 1 | 1.216269 | 1.073861 |
| Romo1 | 0.042185 | -0.26869 | 0.164 | 0.248 | 1 | 0.764384 | -1.47369 |
| Tmem245 | 0.04225 | -0.243 | 0.055 | 0.118 | 1 | 0.784274 | -1.33279 |
| Glul | 0.042668 | 0.226166 | 0.148 | 0.081 | 1 | 1.253784 | 1.240478 |
| Rfxap | 0.0427 | -0.25177 | 0.07 | 0.138 | 1 | 0.777424 | -1.38091 |
| Gpx4 | 0.042888 | 0.220588 | 0.453 | 0.341 | 1 | 1.24681 | 1.209884 |
| Pttg1ip | 0.043058 | 0.184935 | 0.148 | 0.081 | 1 | 1.20314 | 1.014334 |
| Ddx10 | 0.043815 | -0.30462 | 0.086 | 0.154 | 1 | 0.737407 | -1.67076 |
| Dync1h1 | 0.04465 | 0.186811 | 0.148 | 0.081 | 1 | 1.2054 | 1.024626 |
| Rps19bp1 | 0.044706 | -0.21562 | 0.055 | 0.118 | 1 | 0.806042 | -1.18263 |
| Stk24 | 0.045097 | 0.191776 | 0.438 | 0.325 | 1 | 1.211399 | 1.051856 |
| Ndufaf8 | 0.045366 | -0.22319 | 0.109 | 0.183 | 1 | 0.799964 | -1.22415 |
| Farsb | 0.045546 | 0.250448 | 0.234 | 0.15 | 1 | 1.2846 | 1.373659 |
| Cnn3 | 0.046241 | 0.218148 | 0.219 | 0.138 | 1 | 1.243771 | 1.196502 |
| Pgd | 0.046844 | -0.20916 | 0.055 | 0.118 | 1 | 0.811265 | -1.14721 |
| Hsd17b11 | 0.046877 | 0.184822 | 0.102 | 0.049 | 1 | 1.203004 | 1.013714 |
| Zyg11b | 0.048252 | 0.190076 | 0.125 | 0.065 | 1 | 1.209341 | 1.04253 |
| Pdia3 | 0.048541 | 0.192621 | 0.359 | 0.26 | 1 | 1.212423 | 1.05649 |
| Psmb4 | 0.049695 | -0.19999 | 0.234 | 0.325 | 1 | 0.818738 | -1.09691 |
| 2310001H17Rik | 0.049792 | 0.214116 | 0.18 | 0.11 | 1 | 1.238766 | 1.174387 |

**Table S3 Results of differential gene expression analysis at 7 days after LPS and LPS/MSC treatment**

| gene | p_val | avg_logFC | pct.1 | pct.2 | p_val_adj | Foldchange | log1.2FC |
| --- | --- | --- | --- | --- | --- | --- | --- |
| Uba52 | 1.23E-82 | 0.672639 | 0.99 | 0.924 | 3.83E-78 | 1.959401 | 3.689298 |
| Saa3 | 4.48E-76 | -0.8111 | 0.878 | 0.986 | 1.39E-71 | 0.444371 | -4.44871 |
| Rps27 | 3.37E-51 | -0.25637 | 1 | 1 | 1.05E-46 | 0.773858 | -1.40613 |
| Rps27rt | 2.12E-45 | -0.66794 | 0.659 | 0.892 | 6.58E-41 | 0.512763 | -3.66354 |
| Fau | 4.68E-41 | -0.24049 | 1 | 0.998 | 1.45E-36 | 0.786246 | -1.31902 |
| Rpl8 | 1.09E-37 | -0.2878 | 0.998 | 1 | 3.39E-33 | 0.749908 | -1.57856 |
| Dnaja1 | 3.49E-37 | -0.67438 | 0.598 | 0.844 | 1.08E-32 | 0.509472 | -3.69885 |
| Tmsb4x | 3.72E-37 | 0.283679 | 1 | 1 | 1.16E-32 | 1.328007 | 1.555929 |
| Rpl10 | 5.47E-35 | 0.341403 | 0.998 | 0.986 | 1.70E-30 | 1.406919 | 1.87253 |
| Tmsb10 | 5.58E-34 | 0.409089 | 0.993 | 0.936 | 1.73E-29 | 1.505446 | 2.243777 |
| Rpl21 | 2.11E-33 | -0.2472 | 1 | 1 | 6.55E-29 | 0.780985 | -1.35584 |
| mt-Atp6 | 1.86E-32 | -0.21552 | 1 | 1 | 5.79E-28 | 0.806125 | -1.18207 |
| Rbm3 | 5.99E-31 | -0.59078 | 0.622 | 0.826 | 1.86E-26 | 0.553894 | -3.24033 |
| Rps28 | 1.21E-30 | -0.3082 | 0.99 | 0.993 | 3.75E-26 | 0.734771 | -1.6904 |
| Lyz2 | 1.76E-30 | 0.402283 | 0.993 | 0.976 | 5.48E-26 | 1.495234 | 2.206445 |
| Rpl22 | 6.48E-26 | 0.294429 | 0.99 | 0.983 | 2.01E-21 | 1.342359 | 1.614886 |
| S100a9 | 2.55E-25 | 0.597731 | 0.808 | 0.576 | 7.91E-21 | 1.81799 | 3.278446 |
| Hsp90aa1 | 4.46E-25 | -0.57961 | 0.726 | 0.891 | 1.39E-20 | 0.560118 | -3.17905 |
| Cxcr4 | 1.16E-24 | -0.56373 | 0.652 | 0.83 | 3.61E-20 | 0.569082 | -3.09196 |
| Sh3bgrl3 | 2.58E-24 | 0.407738 | 0.925 | 0.783 | 8.00E-20 | 1.503413 | 2.236368 |
| Ly6d | 3.27E-24 | 0.533343 | 0.853 | 0.674 | 1.01E-19 | 1.704621 | 2.925288 |
| Rpl29 | 2.48E-23 | -0.28262 | 0.967 | 0.983 | 7.70E-19 | 0.753803 | -1.55014 |
| Rpl12 | 1.12E-22 | -0.207 | 0.998 | 0.998 | 3.49E-18 | 0.813017 | -1.13537 |
| Rps26 | 1.88E-22 | 0.235296 | 0.998 | 0.988 | 5.84E-18 | 1.265283 | 1.290556 |
| Btg2 | 4.91E-22 | -0.52383 | 0.687 | 0.832 | 1.52E-17 | 0.592248 | -2.87311 |
| Rpl37 | 6.62E-22 | 0.233737 | 0.998 | 0.995 | 2.05E-17 | 1.263313 | 1.282006 |
| Gm10076 | 5.21E-21 | 0.549431 | 0.645 | 0.413 | 1.62E-16 | 1.732267 | 3.013527 |
| S100a11 | 4.54E-20 | 0.556904 | 0.61 | 0.368 | 1.41E-15 | 1.74526 | 3.054513 |
| Rps12 | 1.37E-19 | 0.229818 | 1 | 0.997 | 4.26E-15 | 1.25837 | 1.260507 |
| Serf2 | 3.06E-19 | 0.410749 | 0.837 | 0.661 | 9.49E-15 | 1.507946 | 2.25288 |
| Klf2 | 8.62E-19 | 0.561042 | 0.591 | 0.345 | 2.68E-14 | 1.752497 | 3.077209 |
| H2-DMb2 | 5.82E-18 | 0.479328 | 0.657 | 0.438 | 1.81E-13 | 1.614989 | 2.629025 |
| AY036118 | 8.14E-18 | -0.45364 | 0.614 | 0.807 | 2.53E-13 | 0.635312 | -2.48812 |
| Dnajb1 | 3.00E-17 | -0.47757 | 0.253 | 0.486 | 9.31E-13 | 0.620292 | -2.61936 |
| Mat2a | 1.30E-16 | -0.52927 | 0.397 | 0.608 | 4.04E-12 | 0.589035 | -2.90295 |
| Rpl35 | 5.80E-16 | 0.191594 | 0.993 | 0.993 | 1.80E-11 | 1.211179 | 1.05086 |
| Rsrp1 | 7.96E-16 | -0.40075 | 0.615 | 0.766 | 2.47E-11 | 0.669818 | -2.19804 |
| Arhgdib | 3.16E-15 | 0.378753 | 0.816 | 0.71 | 9.81E-11 | 1.460463 | 2.077393 |
| mt-Nd4l | 3.61E-15 | 0.435142 | 0.698 | 0.519 | 1.12E-10 | 1.545183 | 2.386675 |
| Hspa8 | 7.50E-15 | -0.29577 | 0.948 | 0.969 | 2.33E-10 | 0.743962 | -1.62222 |
| Rpl15 | 1.52E-14 | 0.203452 | 0.995 | 0.981 | 4.71E-10 | 1.225626 | 1.115897 |
| Fcer2a | 2.74E-14 | 0.487992 | 0.395 | 0.193 | 8.52E-10 | 1.629042 | 2.676545 |
| Junb | 4.99E-14 | -0.31289 | 0.942 | 0.974 | 1.55E-09 | 0.73133 | -1.71614 |
| H2-Ab1 | 7.84E-14 | 0.224469 | 0.991 | 0.965 | 2.43E-09 | 1.251658 | 1.231172 |
| Dynll1 | 9.86E-14 | -0.39139 | 0.544 | 0.701 | 3.06E-09 | 0.676114 | -2.14672 |
| Slc38a2 | 1.62E-13 | -0.41029 | 0.642 | 0.752 | 5.03E-09 | 0.663459 | -2.25035 |
| Lsp1 | 3.57E-13 | 0.427688 | 0.605 | 0.403 | 1.11E-08 | 1.533708 | 2.345792 |
| H3f3b | 6.62E-13 | 0.235028 | 0.976 | 0.95 | 2.06E-08 | 1.264944 | 1.289083 |
| Myl6 | 1.61E-12 | 0.371514 | 0.724 | 0.575 | 4.99E-08 | 1.449927 | 2.037683 |
| Wfdc17 | 1.61E-12 | -0.37872 | 0.556 | 0.715 | 5.01E-08 | 0.68474 | -2.07718 |
| Ccl6 | 1.91E-12 | 0.459913 | 0.294 | 0.128 | 5.92E-08 | 1.583935 | 2.522535 |
| Grap | 2.77E-12 | 0.442558 | 0.334 | 0.158 | 8.59E-08 | 1.556684 | 2.427349 |
| Kdm6b | 6.00E-12 | -0.43008 | 0.336 | 0.53 | 1.86E-07 | 0.65046 | -2.35889 |
| Ifi27l2a | 6.67E-12 | 0.599658 | 0.362 | 0.198 | 2.07E-07 | 1.821495 | 3.289012 |
| Foxp1 | 1.76E-11 | 0.30238 | 0.813 | 0.675 | 5.47E-07 | 1.353075 | 1.658498 |
| Gm8369 | 2.20E-11 | -0.35528 | 0.57 | 0.719 | 6.83E-07 | 0.700978 | -1.94864 |
| Npm1 | 2.56E-11 | 0.318912 | 0.827 | 0.729 | 7.95E-07 | 1.37563 | 1.749174 |
| Stat3 | 2.56E-11 | -0.49743 | 0.24 | 0.405 | 7.95E-07 | 0.608091 | -2.72831 |
| Gm42418 | 3.35E-11 | -0.19394 | 1 | 0.997 | 1.04E-06 | 0.823705 | -1.06374 |
| Crip1 | 4.50E-11 | 0.559389 | 0.724 | 0.571 | 1.40E-06 | 1.749603 | 3.068146 |
| Ptprc | 6.97E-11 | 0.307762 | 0.74 | 0.597 | 2.16E-06 | 1.360377 | 1.688015 |
| Ppia | 9.93E-11 | 0.225404 | 0.972 | 0.951 | 3.08E-06 | 1.252828 | 1.236298 |
| Ctsd | 1.84E-10 | 0.327415 | 0.666 | 0.486 | 5.72E-06 | 1.387378 | 1.795812 |
| Farsb | 2.01E-10 | -0.42519 | 0.105 | 0.243 | 6.23E-06 | 0.653646 | -2.33208 |
| Neurl3 | 2.06E-10 | -0.40141 | 0.444 | 0.606 | 6.38E-06 | 0.669376 | -2.20165 |
| Hspa1b | 2.23E-10 | -0.47449 | 0.292 | 0.495 | 6.91E-06 | 0.622202 | -2.6025 |
| Nr4a1 | 2.33E-10 | -0.42226 | 0.406 | 0.571 | 7.23E-06 | 0.655566 | -2.316 |
| Serinc3 | 3.10E-10 | -0.27884 | 0.806 | 0.891 | 9.63E-06 | 0.756661 | -1.52939 |
| Zfp36 | 3.14E-10 | -0.39789 | 0.57 | 0.714 | 9.74E-06 | 0.671738 | -2.18234 |
| Irf1 | 4.04E-10 | -0.45045 | 0.194 | 0.347 | 1.25E-05 | 0.63734 | -2.47064 |
| mt-Nd2 | 5.28E-10 | 0.193291 | 0.97 | 0.967 | 1.64E-05 | 1.213235 | 1.060164 |
| Ezr | 6.74E-10 | 0.397972 | 0.535 | 0.366 | 2.09E-05 | 1.488803 | 2.182805 |
| Cxcl2 | 1.15E-09 | 0.415856 | 0.72 | 0.597 | 3.56E-05 | 1.515668 | 2.280895 |
| Iglc2 | 1.46E-09 | 1.164785 | 0.607 | 0.438 | 4.54E-05 | 3.205232 | 6.388628 |
| Btg1 | 1.48E-09 | -0.26458 | 0.923 | 0.962 | 4.61E-05 | 0.767532 | -1.45115 |
| Dedd2 | 1.75E-09 | -0.34144 | 0.096 | 0.226 | 5.43E-05 | 0.710748 | -1.87272 |
| Hmgb2 | 1.83E-09 | 0.418237 | 0.519 | 0.373 | 5.69E-05 | 1.51928 | 2.293951 |
| Sbno2 | 2.26E-09 | -0.49541 | 0.135 | 0.273 | 7.02E-05 | 0.609322 | -2.71722 |
| Arid5a | 3.14E-09 | -0.39712 | 0.238 | 0.392 | 9.76E-05 | 0.672253 | -2.17813 |
| Satb1 | 3.38E-09 | -0.35466 | 0.607 | 0.71 | 0.000105 | 0.701409 | -1.94527 |
| Mcl1 | 4.14E-09 | -0.31166 | 0.687 | 0.79 | 0.000129 | 0.732232 | -1.70939 |
| Ddx5 | 7.37E-09 | 0.209906 | 0.958 | 0.936 | 0.000229 | 1.233562 | 1.151297 |
| Ralgps2 | 8.05E-09 | 0.36248 | 0.521 | 0.368 | 0.00025 | 1.436888 | 1.988135 |
| S100a10 | 9.55E-09 | 0.375065 | 0.545 | 0.406 | 0.000296 | 1.455086 | 2.057164 |
| S100a8 | 9.98E-09 | 0.3053 | 0.818 | 0.698 | 0.00031 | 1.357032 | 1.674514 |
| Vim | 1.35E-08 | 0.449867 | 0.64 | 0.53 | 0.00042 | 1.568103 | 2.467437 |
| Stap1 | 1.65E-08 | 0.387714 | 0.42 | 0.273 | 0.000511 | 1.473608 | 2.126537 |
| Mir142hg | 2.18E-08 | -0.30603 | 0.045 | 0.142 | 0.000677 | 0.736364 | -1.67852 |
| Lgmn | 2.42E-08 | -0.32376 | 0.212 | 0.354 | 0.000752 | 0.723425 | -1.77575 |
| Cox7c | 2.63E-08 | 0.302098 | 0.731 | 0.595 | 0.000817 | 1.352694 | 1.656952 |
| BC017643 | 2.76E-08 | -0.31556 | 0.145 | 0.278 | 0.000858 | 0.729377 | -1.73081 |
| Sdc4 | 2.79E-08 | -0.37853 | 0.236 | 0.377 | 0.000867 | 0.684869 | -2.07616 |
| Socs3 | 3.42E-08 | -0.36799 | 0.145 | 0.273 | 0.001062 | 0.692123 | -2.01836 |
| Cebpb | 3.46E-08 | -0.31394 | 0.64 | 0.759 | 0.001075 | 0.730565 | -1.72189 |
| Cr2 | 4.31E-08 | 0.367109 | 0.22 | 0.102 | 0.001337 | 1.443556 | 2.013527 |
| Plaur | 5.01E-08 | -0.30509 | 0.476 | 0.609 | 0.001555 | 0.737059 | -1.67335 |
| Rac2 | 5.43E-08 | 0.294226 | 0.64 | 0.505 | 0.001685 | 1.342087 | 1.613775 |
| Clk1 | 7.30E-08 | -0.26633 | 0.661 | 0.769 | 0.002266 | 0.766189 | -1.46075 |
| Nap1l1 | 1.19E-07 | 0.308794 | 0.544 | 0.408 | 0.00368 | 1.361781 | 1.693677 |
| Ndufa13 | 1.32E-07 | 0.272448 | 0.451 | 0.3 | 0.004101 | 1.313175 | 1.494325 |
| Rhoa | 1.46E-07 | 0.30016 | 0.74 | 0.625 | 0.004531 | 1.350075 | 1.646323 |
| Ets1 | 1.82E-07 | 0.255635 | 0.753 | 0.625 | 0.005637 | 1.291282 | 1.402112 |
| Hspa1a | 1.94E-07 | -0.30751 | 0.243 | 0.403 | 0.006019 | 0.735275 | -1.68664 |
| Slc25a3 | 1.98E-07 | -0.27433 | 0.537 | 0.66 | 0.006138 | 0.76008 | -1.50466 |
| Hnrnpc | 2.12E-07 | 0.332633 | 0.456 | 0.328 | 0.006585 | 1.394636 | 1.824431 |
| 4930523C07Rik | 2.50E-07 | 0.313453 | 0.537 | 0.399 | 0.007769 | 1.368141 | 1.719232 |
| Swap70 | 2.64E-07 | 0.357472 | 0.266 | 0.148 | 0.008185 | 1.42971 | 1.960667 |
| Ptpn1 | 2.82E-07 | -0.35838 | 0.313 | 0.441 | 0.008767 | 0.698808 | -1.96564 |
| Ubc | 3.14E-07 | -0.23137 | 0.834 | 0.896 | 0.009764 | 0.793445 | -1.26903 |
| Ier2 | 3.24E-07 | -0.27666 | 0.565 | 0.681 | 0.010063 | 0.758313 | -1.51742 |
| Atp5e | 3.90E-07 | 0.237527 | 0.806 | 0.701 | 0.012121 | 1.26811 | 1.302793 |
| 5031425E22Rik | 4.12E-07 | -0.33449 | 0.227 | 0.358 | 0.012808 | 0.715706 | -1.83459 |
| Ppdpf | 4.31E-07 | 0.304948 | 0.332 | 0.205 | 0.013384 | 1.356555 | 1.672584 |
| Ly6e | 4.38E-07 | 0.194708 | 0.937 | 0.891 | 0.013606 | 1.214956 | 1.067938 |
| Eef1d | 4.45E-07 | 0.278938 | 0.622 | 0.483 | 0.013831 | 1.321726 | 1.529925 |
| Chchd2 | 4.72E-07 | -0.24291 | 0.729 | 0.786 | 0.014658 | 0.784343 | -1.33231 |
| H2-Q7 | 4.79E-07 | 0.341601 | 0.491 | 0.354 | 0.014879 | 1.407199 | 1.87362 |
| Calm1 | 5.08E-07 | 0.254935 | 0.811 | 0.745 | 0.01576 | 1.290377 | 1.398269 |
| Coq10b | 5.74E-07 | -0.33653 | 0.288 | 0.415 | 0.017816 | 0.714242 | -1.84582 |
| Cd79b | 5.77E-07 | 0.239567 | 0.783 | 0.684 | 0.01793 | 1.270699 | 1.313982 |
| Sept6 | 6.40E-07 | 0.294478 | 0.243 | 0.13 | 0.019879 | 1.342425 | 1.615157 |
| Mdh1 | 6.51E-07 | 0.308653 | 0.309 | 0.189 | 0.020205 | 1.36159 | 1.692906 |
| Hmox1 | 6.79E-07 | -0.30111 | 0.073 | 0.168 | 0.02108 | 0.739995 | -1.65154 |
| Tob2 | 8.38E-07 | -0.35415 | 0.323 | 0.453 | 0.026008 | 0.701771 | -1.94244 |
| Man1a | 9.01E-07 | 0.282557 | 0.486 | 0.347 | 0.027968 | 1.326517 | 1.549773 |
| Tmem123 | 9.64E-07 | 0.275685 | 0.409 | 0.28 | 0.029926 | 1.317433 | 1.51208 |
| Gem | 1.20E-06 | -0.34258 | 0.18 | 0.297 | 0.037404 | 0.709934 | -1.87901 |
| Sem1 | 1.45E-06 | 0.266042 | 0.6 | 0.486 | 0.045091 | 1.30479 | 1.45919 |
| Emb | 1.49E-06 | -0.42674 | 0.154 | 0.266 | 0.046133 | 0.652633 | -2.34059 |
| Cd52 | 1.64E-06 | 0.220403 | 0.923 | 0.872 | 0.051047 | 1.246579 | 1.20887 |
| Fkbp5 | 2.13E-06 | -0.28653 | 0.068 | 0.156 | 0.066124 | 0.750866 | -1.57155 |
| Add3 | 2.16E-06 | 0.329465 | 0.369 | 0.247 | 0.067127 | 1.390224 | 1.807052 |
| Csrnp1 | 2.24E-06 | -0.31318 | 0.196 | 0.311 | 0.069597 | 0.731115 | -1.71776 |
| Pfn1 | 2.31E-06 | 0.236585 | 0.867 | 0.826 | 0.07186 | 1.266915 | 1.297624 |
| Ahsa1 | 2.37E-06 | -0.29785 | 0.24 | 0.359 | 0.073672 | 0.74241 | -1.63367 |
| Cd2 | 2.40E-06 | 0.245191 | 0.334 | 0.208 | 0.074533 | 1.277866 | 1.344829 |
| Bbip1 | 2.46E-06 | 0.231287 | 0.135 | 0.054 | 0.076513 | 1.260221 | 1.268569 |
| Arpc3 | 2.80E-06 | 0.228467 | 0.741 | 0.62 | 0.086794 | 1.256672 | 1.253098 |
| Cacybp | 2.82E-06 | -0.35253 | 0.241 | 0.349 | 0.087663 | 0.70291 | -1.93355 |
| Ldb1 | 3.34E-06 | -0.23 | 0.089 | 0.184 | 0.103839 | 0.794535 | -1.2615 |
| Eef1g | 3.41E-06 | 0.214171 | 0.759 | 0.677 | 0.10588 | 1.238834 | 1.174688 |
| Cd55 | 3.69E-06 | 0.280076 | 0.554 | 0.403 | 0.114649 | 1.323231 | 1.536166 |
| Hmgn2 | 4.24E-06 | 0.258095 | 0.238 | 0.132 | 0.131542 | 1.294462 | 1.415604 |
| Rpl22l1 | 4.33E-06 | 0.224542 | 0.788 | 0.759 | 0.134351 | 1.25175 | 1.231574 |
| Prkcb | 4.40E-06 | 0.241772 | 0.339 | 0.224 | 0.136482 | 1.273504 | 1.326076 |
| Tagln2 | 5.11E-06 | 0.420344 | 0.376 | 0.267 | 0.158532 | 1.522485 | 2.305508 |
| Cdc37 | 5.87E-06 | 0.257177 | 0.262 | 0.155 | 0.182343 | 1.293274 | 1.410567 |
| Pim1 | 6.53E-06 | -0.40256 | 0.374 | 0.479 | 0.202712 | 0.668603 | -2.20799 |
| Elob | 7.99E-06 | 0.252428 | 0.563 | 0.441 | 0.248011 | 1.287146 | 1.384519 |
| Siah2 | 8.57E-06 | -0.22957 | 0.065 | 0.144 | 0.266106 | 0.794874 | -1.25916 |
| Lmo4 | 9.25E-06 | -0.28414 | 0.112 | 0.207 | 0.287378 | 0.752665 | -1.55843 |
| Lcp1 | 9.29E-06 | 0.237449 | 0.565 | 0.438 | 0.288391 | 1.26801 | 1.302364 |
| Calr | 1.17E-05 | 0.275763 | 0.572 | 0.474 | 0.363795 | 1.317536 | 1.512512 |
| Mef2c | 1.20E-05 | 0.230792 | 0.867 | 0.79 | 0.371189 | 1.259597 | 1.265849 |
| Gm26740 | 1.26E-05 | -0.27416 | 0.372 | 0.484 | 0.392617 | 0.760209 | -1.50373 |
| Lman2l | 1.30E-05 | -0.2135 | 0.054 | 0.128 | 0.403734 | 0.807752 | -1.17101 |
| Dok3 | 1.35E-05 | -0.2551 | 0.201 | 0.311 | 0.41781 | 0.774837 | -1.39919 |
| Cdc42 | 1.37E-05 | 0.197401 | 0.753 | 0.668 | 0.424388 | 1.218232 | 1.082708 |
| Birc2 | 1.60E-05 | -0.29214 | 0.187 | 0.292 | 0.497147 | 0.746666 | -1.60232 |
| Ltb | 1.66E-05 | 0.289277 | 0.425 | 0.314 | 0.516908 | 1.335462 | 1.586633 |
| Litaf | 1.91E-05 | -0.33198 | 0.222 | 0.318 | 0.591932 | 0.7175 | -1.82086 |
| Plekho2 | 1.92E-05 | -0.25238 | 0.11 | 0.2 | 0.596133 | 0.776951 | -1.38424 |
| Rnase6 | 2.15E-05 | 0.234292 | 0.194 | 0.104 | 0.667355 | 1.264014 | 1.285049 |
| Skil | 2.33E-05 | -0.32096 | 0.243 | 0.342 | 0.722919 | 0.725456 | -1.76038 |
| Lax1 | 2.57E-05 | -0.23504 | 0.063 | 0.135 | 0.797342 | 0.790537 | -1.28917 |
| Zc3h15 | 2.72E-05 | 0.228329 | 0.309 | 0.203 | 0.843142 | 1.256499 | 1.252345 |
| Gm6377 | 3.12E-05 | -0.25589 | 0.105 | 0.191 | 0.969434 | 0.774228 | -1.40351 |
| Snrpd1 | 3.31E-05 | 0.283537 | 0.238 | 0.148 | 1 | 1.327818 | 1.555147 |
| Magt1 | 3.35E-05 | 0.250398 | 0.159 | 0.082 | 1 | 1.284536 | 1.373385 |
| Hmgb1 | 3.53E-05 | 0.205145 | 0.764 | 0.693 | 1 | 1.227702 | 1.12518 |
| Bcl3 | 3.69E-05 | -0.22216 | 0.107 | 0.194 | 1 | 0.800783 | -1.21853 |
| Rpa3 | 4.02E-05 | -0.18856 | 0.044 | 0.108 | 1 | 0.828154 | -1.0342 |
| Grcc10 | 4.09E-05 | 0.23553 | 0.24 | 0.144 | 1 | 1.265579 | 1.291836 |
| Sri | 4.11E-05 | 0.210268 | 0.341 | 0.227 | 1 | 1.234009 | 1.153281 |
| Gm2000 | 4.34E-05 | -0.21331 | 0.096 | 0.179 | 1 | 0.807904 | -1.16998 |
| Rbx1 | 4.50E-05 | 0.229503 | 0.5 | 0.389 | 1 | 1.257975 | 1.258784 |
| Rps27l | 4.51E-05 | 0.259429 | 0.35 | 0.243 | 1 | 1.29619 | 1.422922 |
| Anp32a | 4.98E-05 | 0.26611 | 0.385 | 0.285 | 1 | 1.304878 | 1.459562 |
| Pnrc1 | 4.98E-05 | -0.1863 | 0.706 | 0.795 | 1 | 0.830027 | -1.02181 |
| Zmat2 | 5.16E-05 | 0.278263 | 0.21 | 0.125 | 1 | 1.320833 | 1.526219 |
| Pglyrp1 | 6.63E-05 | 0.226626 | 0.142 | 0.069 | 1 | 1.254361 | 1.243004 |
| Abca1 | 6.98E-05 | -0.20076 | 0.175 | 0.273 | 1 | 0.818112 | -1.10111 |
| Mrps21 | 7.06E-05 | 0.221162 | 0.231 | 0.142 | 1 | 1.247525 | 1.213031 |
| Pcmtd1 | 7.12E-05 | 0.296075 | 0.278 | 0.184 | 1 | 1.344571 | 1.623918 |
| Ptp4a3 | 7.52E-05 | 0.260607 | 0.336 | 0.233 | 1 | 1.297717 | 1.42938 |
| Ccdc12 | 7.71E-05 | 0.185302 | 0.29 | 0.186 | 1 | 1.203581 | 1.016345 |
| Gpx4 | 8.43E-05 | -0.2233 | 0.367 | 0.465 | 1 | 0.799877 | -1.22475 |
| Lst1 | 8.67E-05 | 0.252926 | 0.163 | 0.087 | 1 | 1.287788 | 1.387253 |
| Tpm3 | 8.82E-05 | 0.208638 | 0.603 | 0.491 | 1 | 1.231999 | 1.144342 |
| Banf1 | 8.87E-05 | 0.234771 | 0.248 | 0.156 | 1 | 1.26462 | 1.287678 |
| Ahsa2 | 8.94E-05 | -0.18398 | 0.066 | 0.137 | 1 | 0.831956 | -1.00908 |
| AW112010 | 9.03E-05 | -0.2835 | 0.226 | 0.323 | 1 | 0.753144 | -1.55494 |
| Apobec3 | 9.10E-05 | -0.2064 | 0.507 | 0.615 | 1 | 0.813504 | -1.13209 |
| Adgre5 | 0.000102 | 0.305649 | 0.358 | 0.266 | 1 | 1.357506 | 1.67643 |
| Dnajc7 | 0.000103 | 0.21701 | 0.299 | 0.2 | 1 | 1.242356 | 1.190257 |
| Arl5c | 0.000104 | -0.18587 | 0.107 | 0.191 | 1 | 0.830383 | -1.01946 |
| Eif2s2 | 0.000106 | 0.217196 | 0.49 | 0.384 | 1 | 1.242588 | 1.191281 |
| Ifi203 | 0.000107 | 0.271737 | 0.465 | 0.354 | 1 | 1.312241 | 1.490426 |
| Il1b | 0.000115 | 0.191439 | 0.584 | 0.49 | 1 | 1.210991 | 1.050006 |
| Sf3b2 | 0.000115 | 0.231148 | 0.404 | 0.293 | 1 | 1.260046 | 1.267807 |
| Cox16 | 0.000116 | 0.236346 | 0.114 | 0.052 | 1 | 1.266612 | 1.296312 |
| U2af1 | 0.000122 | 0.211795 | 0.262 | 0.168 | 1 | 1.235895 | 1.161658 |
| March1 | 0.000125 | 0.25361 | 0.28 | 0.186 | 1 | 1.288669 | 1.391003 |
| Cox6c | 0.000129 | 0.214014 | 0.661 | 0.582 | 1 | 1.23864 | 1.173827 |
| Ctsl | 0.000142 | -0.25926 | 0.133 | 0.215 | 1 | 0.771623 | -1.42199 |
| Fam174a | 0.000147 | -0.27598 | 0.161 | 0.243 | 1 | 0.758825 | -1.51372 |
| Ly6c2 | 0.000149 | -0.23902 | 0.161 | 0.252 | 1 | 0.787396 | -1.311 |
| Snrnp25 | 0.000157 | 0.227913 | 0.133 | 0.068 | 1 | 1.255976 | 1.250061 |
| Zufsp | 0.000159 | 0.187402 | 0.18 | 0.101 | 1 | 1.206112 | 1.027865 |
| Clic1 | 0.000163 | 0.201229 | 0.633 | 0.531 | 1 | 1.222904 | 1.103701 |
| Wdr89 | 0.000164 | -0.26025 | 0.427 | 0.507 | 1 | 0.770858 | -1.42743 |
| Tgif1 | 0.000168 | -0.21696 | 0.381 | 0.472 | 1 | 0.804965 | -1.18997 |
| Ddx54 | 0.000169 | 0.237605 | 0.231 | 0.146 | 1 | 1.268208 | 1.303217 |
| Emg1 | 0.00017 | 0.264145 | 0.264 | 0.177 | 1 | 1.302317 | 1.448786 |
| Gt(ROSA)26Sor | 0.000185 | -0.22836 | 0.122 | 0.203 | 1 | 0.795839 | -1.2525 |
| Fosl2 | 0.000188 | -0.28635 | 0.121 | 0.2 | 1 | 0.751003 | -1.57055 |
| Atp5k | 0.000189 | 0.265972 | 0.329 | 0.238 | 1 | 1.304698 | 1.458805 |
| Stt3b | 0.000194 | 0.272773 | 0.274 | 0.191 | 1 | 1.313602 | 1.496108 |
| Dbnl | 0.000208 | 0.210992 | 0.213 | 0.134 | 1 | 1.234903 | 1.157253 |
| C1d | 0.000209 | 0.189681 | 0.133 | 0.068 | 1 | 1.208864 | 1.040366 |
| Ranbp1 | 0.000224 | 0.25742 | 0.292 | 0.201 | 1 | 1.293588 | 1.411902 |
| Ms4a4c | 0.000224 | 0.312459 | 0.299 | 0.215 | 1 | 1.366782 | 1.713779 |
| Cd9 | 0.000227 | 0.234611 | 0.255 | 0.168 | 1 | 1.264417 | 1.286797 |
| Hsph1 | 0.000233 | -0.22505 | 0.131 | 0.212 | 1 | 0.798475 | -1.23436 |
| Ighd | 0.000233 | 0.19007 | 0.753 | 0.674 | 1 | 1.209334 | 1.042499 |
| Dock2 | 0.000237 | 0.251596 | 0.392 | 0.3 | 1 | 1.286076 | 1.379957 |
| Cldnd1 | 0.000242 | -0.18533 | 0.072 | 0.137 | 1 | 0.830833 | -1.01648 |
| Nrm | 0.000246 | -0.23655 | 0.129 | 0.207 | 1 | 0.789344 | -1.29745 |
| Mrps14 | 0.000249 | 0.200817 | 0.257 | 0.167 | 1 | 1.222402 | 1.101446 |
| Jmjd1c | 0.000251 | -0.24717 | 0.351 | 0.446 | 1 | 0.781006 | -1.3557 |
| Mrpl54 | 0.00026 | 0.203465 | 0.198 | 0.12 | 1 | 1.225643 | 1.115969 |
| Mrps16 | 0.000264 | 0.190499 | 0.226 | 0.142 | 1 | 1.209853 | 1.044852 |
| Pou2af1 | 0.000266 | 0.196632 | 0.554 | 0.446 | 1 | 1.217296 | 1.078492 |
| Rad23b | 0.00027 | 0.212565 | 0.199 | 0.122 | 1 | 1.236846 | 1.165877 |
| Ints6 | 0.000276 | -0.22304 | 0.152 | 0.234 | 1 | 0.80008 | -1.22335 |
| Srsf2 | 0.000281 | -0.2041 | 0.503 | 0.604 | 1 | 0.815384 | -1.11943 |
| Anxa2 | 0.000284 | 0.203652 | 0.152 | 0.082 | 1 | 1.225871 | 1.116992 |
| Atpif1 | 0.000293 | 0.192731 | 0.351 | 0.257 | 1 | 1.212556 | 1.057092 |
| Ddx24 | 0.000304 | 0.192708 | 0.315 | 0.217 | 1 | 1.212528 | 1.056966 |
| Cap1 | 0.000319 | 0.214825 | 0.184 | 0.111 | 1 | 1.239645 | 1.178275 |
| Ms4a1 | 0.000332 | 0.207556 | 0.703 | 0.623 | 1 | 1.230667 | 1.138407 |
| Dusp2 | 0.000347 | -0.32868 | 0.439 | 0.503 | 1 | 0.719872 | -1.80276 |
| Chchd1 | 0.000351 | 0.202068 | 0.226 | 0.142 | 1 | 1.223931 | 1.108305 |
| Hexim1 | 0.000356 | -0.21774 | 0.171 | 0.252 | 1 | 0.804333 | -1.19427 |
| Txnip | 0.000365 | 0.199604 | 0.414 | 0.316 | 1 | 1.220919 | 1.094789 |
| Gadd45b | 0.000365 | -0.40218 | 0.252 | 0.333 | 1 | 0.668862 | -2.20587 |
| Capg | 0.000365 | 0.185137 | 0.434 | 0.335 | 1 | 1.203383 | 1.015442 |
| Gm11808 | 0.000392 | -0.24998 | 0.406 | 0.49 | 1 | 0.77882 | -1.37107 |
| Cox7b | 0.000397 | 0.192691 | 0.444 | 0.354 | 1 | 1.212509 | 1.056877 |
| Glud1 | 0.000408 | 0.223187 | 0.206 | 0.13 | 1 | 1.250055 | 1.224142 |
| Trim7 | 0.000439 | 0.208892 | 0.21 | 0.13 | 1 | 1.232312 | 1.145735 |
| Serbp1 | 0.000464 | 0.189158 | 0.654 | 0.564 | 1 | 1.208232 | 1.037499 |
| Dock8 | 0.000492 | 0.189274 | 0.173 | 0.102 | 1 | 1.208372 | 1.038135 |
| Zranb2 | 0.000509 | 0.185202 | 0.15 | 0.083 | 1 | 1.203462 | 1.0158 |
| Wsb1 | 0.000512 | -0.22656 | 0.206 | 0.288 | 1 | 0.797275 | -1.24261 |
| Pkm | 0.000519 | -0.2442 | 0.29 | 0.37 | 1 | 0.783334 | -1.33937 |
| Crem | 0.000525 | -0.21591 | 0.337 | 0.439 | 1 | 0.805807 | -1.18423 |
| Pdia6 | 0.000575 | 0.20132 | 0.248 | 0.165 | 1 | 1.223017 | 1.104205 |
| Thoc7 | 0.000578 | 0.207346 | 0.187 | 0.116 | 1 | 1.230408 | 1.137254 |
| Mrps15 | 0.000593 | 0.196149 | 0.124 | 0.066 | 1 | 1.216708 | 1.075841 |
| Cib1 | 0.00064 | 0.204568 | 0.156 | 0.092 | 1 | 1.226995 | 1.122019 |
| Ucp2 | 0.000641 | 0.211403 | 0.435 | 0.356 | 1 | 1.23541 | 1.159506 |
| Ppp1r12a | 0.000642 | 0.240429 | 0.266 | 0.186 | 1 | 1.271794 | 1.318707 |
| Atp5c1 | 0.000662 | 0.228532 | 0.399 | 0.314 | 1 | 1.256753 | 1.253453 |
| Traf4 | 0.000664 | -0.19955 | 0.079 | 0.141 | 1 | 0.819101 | -1.09449 |
| Anxa5 | 0.000666 | 0.20793 | 0.192 | 0.12 | 1 | 1.231127 | 1.140456 |
| Zc3h13 | 0.000689 | 0.194675 | 0.161 | 0.094 | 1 | 1.214916 | 1.067755 |
| Alkbh1 | 0.000697 | 0.205905 | 0.212 | 0.135 | 1 | 1.228636 | 1.129349 |
| Fam46a | 0.000719 | -0.22788 | 0.101 | 0.167 | 1 | 0.796217 | -1.2499 |
| Nfkbid | 0.000731 | -0.30422 | 0.29 | 0.366 | 1 | 0.737698 | -1.6686 |
| Safb2 | 0.000734 | -0.23835 | 0.21 | 0.288 | 1 | 0.787929 | -1.30729 |
| Plekhm1 | 0.000734 | -0.1851 | 0.098 | 0.165 | 1 | 0.831022 | -1.01524 |
| Jmjd6 | 0.000736 | -0.19444 | 0.117 | 0.189 | 1 | 0.823293 | -1.06649 |
| Atp5f1 | 0.000749 | 0.191139 | 0.425 | 0.325 | 1 | 1.210628 | 1.048364 |
| Cox7a2 | 0.00078 | 0.190151 | 0.531 | 0.443 | 1 | 1.209432 | 1.04294 |
| Psma6 | 0.000786 | 0.211411 | 0.224 | 0.149 | 1 | 1.23542 | 1.159548 |
| Arhgap30 | 0.000817 | -0.22941 | 0.313 | 0.391 | 1 | 0.795 | -1.25829 |
| Lncpint | 0.000835 | -0.18891 | 0.22 | 0.309 | 1 | 0.827857 | -1.03616 |
| Ptpn6 | 0.000921 | 0.250723 | 0.353 | 0.269 | 1 | 1.284954 | 1.375168 |
| Mrps33 | 0.000928 | 0.195511 | 0.21 | 0.137 | 1 | 1.215932 | 1.07234 |
| Gngt2 | 0.000961 | 0.222588 | 0.233 | 0.158 | 1 | 1.249305 | 1.220852 |
| Iglc3 | 0.000992 | 0.237607 | 0.502 | 0.406 | 1 | 1.268211 | 1.30323 |
| S100a13 | 0.001011 | 0.211276 | 0.208 | 0.137 | 1 | 1.235253 | 1.158809 |
| Chd3 | 0.00102 | 0.195072 | 0.168 | 0.104 | 1 | 1.215398 | 1.069933 |
| Fchsd2 | 0.001026 | 0.237336 | 0.381 | 0.293 | 1 | 1.267867 | 1.301742 |
| Ndufa7 | 0.001042 | 0.197498 | 0.409 | 0.323 | 1 | 1.218351 | 1.08324 |
| Rtraf | 0.001145 | 0.183081 | 0.332 | 0.243 | 1 | 1.200912 | 1.004165 |
| Ctnnb1 | 0.001148 | -0.19577 | 0.238 | 0.319 | 1 | 0.822199 | -1.07378 |
| Ptpn18 | 0.001149 | -0.18575 | 0.596 | 0.641 | 1 | 0.830479 | -1.01882 |
| Cd200 | 0.001161 | -0.2063 | 0.089 | 0.151 | 1 | 0.813587 | -1.13153 |
| Tmbim4 | 0.001173 | -0.18943 | 0.138 | 0.208 | 1 | 0.827433 | -1.03897 |
| Ybx1 | 0.001185 | 0.213349 | 0.664 | 0.628 | 1 | 1.237816 | 1.170179 |
| Ubl5 | 0.001232 | 0.189491 | 0.633 | 0.559 | 1 | 1.208634 | 1.039321 |
| Tob1 | 0.001236 | -0.22097 | 0.084 | 0.142 | 1 | 0.80174 | -1.21199 |
| Prkar1a | 0.00126 | 0.240158 | 0.367 | 0.288 | 1 | 1.271451 | 1.317224 |
| Chmp2a | 0.001311 | 0.214928 | 0.262 | 0.188 | 1 | 1.239772 | 1.178838 |
| Atp5o.1 | 0.001339 | 0.208706 | 0.362 | 0.285 | 1 | 1.232082 | 1.144711 |
| Ikzf3 | 0.001348 | 0.182783 | 0.217 | 0.142 | 1 | 1.200554 | 1.002529 |
| Sumo3 | 0.00135 | -0.21531 | 0.112 | 0.177 | 1 | 0.806294 | -1.18092 |
| Gclc | 0.001397 | -0.22021 | 0.061 | 0.113 | 1 | 0.802348 | -1.20783 |
| Fcrla | 0.00141 | 0.210647 | 0.421 | 0.33 | 1 | 1.234477 | 1.155361 |
| Tax1bp1 | 0.00142 | 0.193112 | 0.488 | 0.387 | 1 | 1.213019 | 1.059185 |
| Tbca | 0.001506 | 0.233843 | 0.372 | 0.293 | 1 | 1.263446 | 1.282587 |
| Luc7l3 | 0.001563 | -0.18936 | 0.248 | 0.328 | 1 | 0.827486 | -1.03862 |
| Lcn2 | 0.001588 | -0.22104 | 0.184 | 0.257 | 1 | 0.801683 | -1.21238 |
| Ahnak | 0.001625 | 0.219125 | 0.208 | 0.139 | 1 | 1.244987 | 1.20186 |
| Tnrc6a | 0.001737 | -0.18327 | 0.212 | 0.292 | 1 | 0.832539 | -1.00523 |
| Myadm | 0.001752 | 0.199276 | 0.112 | 0.061 | 1 | 1.220518 | 1.09299 |
| Impdh2 | 0.001776 | 0.246169 | 0.213 | 0.148 | 1 | 1.279116 | 1.350192 |
| Mien1 | 0.001831 | 0.202956 | 0.185 | 0.122 | 1 | 1.225018 | 1.113175 |
| Tomm7 | 0.001874 | 0.183942 | 0.441 | 0.359 | 1 | 1.201946 | 1.008889 |
| Gm26532 | 0.001921 | -0.24181 | 0.15 | 0.219 | 1 | 0.785202 | -1.32631 |
| Ndufb3 | 0.002224 | 0.194747 | 0.28 | 0.208 | 1 | 1.215004 | 1.068154 |
| H2afx | 0.002227 | -0.22785 | 0.1 | 0.156 | 1 | 0.796243 | -1.24972 |
| Eno1 | 0.00228 | 0.217101 | 0.323 | 0.248 | 1 | 1.24247 | 1.190759 |
| Tap1 | 0.002437 | -0.20415 | 0.17 | 0.24 | 1 | 0.815339 | -1.11973 |
| Icam1 | 0.002552 | -0.25318 | 0.098 | 0.153 | 1 | 0.776324 | -1.38867 |
| Cirbp | 0.002607 | -0.19766 | 0.269 | 0.342 | 1 | 0.82065 | -1.08412 |
| Tmem189 | 0.002633 | -0.19398 | 0.108 | 0.167 | 1 | 0.823675 | -1.06394 |
| Dusp11 | 0.002638 | 0.193859 | 0.318 | 0.245 | 1 | 1.213926 | 1.063283 |
| Neu1 | 0.002673 | -0.19451 | 0.149 | 0.214 | 1 | 0.82324 | -1.06683 |
| Mylip | 0.002685 | -0.19232 | 0.154 | 0.22 | 1 | 0.825046 | -1.05482 |
| Dazap1 | 0.002878 | 0.190612 | 0.166 | 0.108 | 1 | 1.20999 | 1.045474 |
| Cyp4f18 | 0.002944 | -0.20497 | 0.213 | 0.283 | 1 | 0.814672 | -1.12422 |
| Pxk | 0.003036 | 0.196269 | 0.315 | 0.241 | 1 | 1.216855 | 1.076501 |
| Cct5 | 0.003114 | 0.207922 | 0.323 | 0.252 | 1 | 1.231117 | 1.140413 |
| Gm26669 | 0.003294 | -0.22934 | 0.093 | 0.146 | 1 | 0.795057 | -1.25789 |
| H2-Q4 | 0.003317 | -0.19919 | 0.4 | 0.467 | 1 | 0.819395 | -1.09252 |
| Slc6a6 | 0.003454 | -0.19401 | 0.126 | 0.186 | 1 | 0.823647 | -1.06413 |
| Neat1 | 0.003557 | -0.19317 | 0.358 | 0.431 | 1 | 0.824343 | -1.0595 |
| Bcl2a1d | 0.003998 | -0.23332 | 0.147 | 0.21 | 1 | 0.791899 | -1.27973 |
| Psme1 | 0.004126 | 0.21053 | 0.49 | 0.422 | 1 | 1.234332 | 1.154719 |
| Psmb2 | 0.004185 | 0.183352 | 0.26 | 0.194 | 1 | 1.201237 | 1.005652 |
| mt-Nd6 | 0.004306 | 0.185502 | 0.171 | 0.115 | 1 | 1.203823 | 1.017445 |
| Cdc37l1 | 0.004455 | -0.19514 | 0.117 | 0.174 | 1 | 0.822722 | -1.07029 |
| Brd8 | 0.004539 | 0.211364 | 0.208 | 0.148 | 1 | 1.235363 | 1.159295 |
| Bcl2 | 0.004811 | 0.201033 | 0.22 | 0.16 | 1 | 1.222665 | 1.102628 |
| 1110004F10Rik | 0.00489 | 0.182444 | 0.219 | 0.155 | 1 | 1.200147 | 1.000673 |
| Bcl2l11 | 0.004939 | -0.19455 | 0.142 | 0.201 | 1 | 0.823206 | -1.06707 |
| Add1 | 0.005285 | 0.182409 | 0.164 | 0.109 | 1 | 1.200104 | 1.000478 |
| Rrbp1 | 0.005321 | -0.23215 | 0.327 | 0.385 | 1 | 0.792824 | -1.27332 |
| Pknox1 | 0.005333 | -0.19452 | 0.066 | 0.113 | 1 | 0.823231 | -1.0669 |
| Hipk1 | 0.005427 | -0.18915 | 0.231 | 0.299 | 1 | 0.82766 | -1.03747 |
| Lrif1 | 0.005591 | -0.2068 | 0.159 | 0.217 | 1 | 0.813183 | -1.13426 |
| Per1 | 0.006386 | -0.23528 | 0.192 | 0.25 | 1 | 0.790347 | -1.29048 |
| Vps29 | 0.006463 | 0.195339 | 0.17 | 0.116 | 1 | 1.215723 | 1.071398 |
| Nr4a3 | 0.006688 | -0.18368 | 0.063 | 0.106 | 1 | 0.832201 | -1.00746 |
| Cers4 | 0.006844 | 0.194886 | 0.122 | 0.076 | 1 | 1.215173 | 1.068914 |
| Arhgap15 | 0.007465 | 0.185757 | 0.497 | 0.444 | 1 | 1.204129 | 1.01884 |
| Pcm1 | 0.00816 | 0.186484 | 0.164 | 0.113 | 1 | 1.205005 | 1.022831 |
| Ssh2 | 0.008378 | -0.18845 | 0.526 | 0.585 | 1 | 0.828243 | -1.03361 |
| Gm47283 | 0.009236 | 0.189871 | 0.229 | 0.172 | 1 | 1.209094 | 1.041408 |
| Nfkbiz | 0.009263 | -0.20243 | 0.154 | 0.212 | 1 | 0.816746 | -1.11028 |
| Susd6 | 0.009917 | 0.18423 | 0.17 | 0.118 | 1 | 1.202293 | 1.010469 |
| Pax5 | 0.010339 | 0.19379 | 0.448 | 0.368 | 1 | 1.213841 | 1.062903 |
| Eif3c | 0.010902 | 0.196093 | 0.329 | 0.267 | 1 | 1.21664 | 1.075533 |
| Picalm | 0.011442 | -0.19095 | 0.292 | 0.352 | 1 | 0.826171 | -1.04735 |
| Gpx1 | 0.012204 | 0.183101 | 0.423 | 0.368 | 1 | 1.200936 | 1.004276 |
| Hs3st1 | 0.012827 | -0.1836 | 0.22 | 0.276 | 1 | 0.832271 | -1.007 |
| Gch1 | 0.013503 | -0.18573 | 0.166 | 0.219 | 1 | 0.8305 | -1.01868 |
| Arglu1 | 0.013559 | -0.19263 | 0.339 | 0.396 | 1 | 0.82479 | -1.05652 |
| Treml2 | 0.015046 | -0.19719 | 0.131 | 0.179 | 1 | 0.821033 | -1.08156 |
| Mob4 | 0.018281 | -0.19035 | 0.222 | 0.271 | 1 | 0.826669 | -1.04404 |
| Lats2 | 0.02431 | -0.18749 | 0.114 | 0.155 | 1 | 0.829039 | -1.02834 |
| Ddx50 | 0.02525 | -0.1909 | 0.299 | 0.354 | 1 | 0.826213 | -1.04707 |
| Ywhah | 0.027202 | -0.18595 | 0.227 | 0.276 | 1 | 0.830319 | -1.01988 |
| Irf4 | 0.028445 | -0.21971 | 0.122 | 0.163 | 1 | 0.80275 | -1.20508 |
| Irs2 | 0.028529 | -0.1936 | 0.196 | 0.245 | 1 | 0.823992 | -1.06183 |
| Lrp10 | 0.029766 | -0.19648 | 0.191 | 0.238 | 1 | 0.821615 | -1.07767 |
| Pou2f2 | 0.037507 | -0.19006 | 0.402 | 0.444 | 1 | 0.82691 | -1.04244 |
| Rrad | 0.043707 | -0.21848 | 0.14 | 0.179 | 1 | 0.803742 | -1.19831 |
| Ube2i | 0.043843 | -0.21615 | 0.371 | 0.408 | 1 | 0.805618 | -1.18552 |
| Clec2i | 0.049261 | -0.19464 | 0.171 | 0.214 | 1 | 0.823132 | -1.06756 |

**Table S4 All markers of each cluster**

| gene | p_val | avg_logFC | pct.1 | pct.2 | p_val_adj | cluster |
| --- | --- | --- | --- | --- | --- | --- |
| Lyz2 | 1.28E-198 | 1.341845 | 0.978 | 0.647 | 3.98E-194 | 0 |
| Gm42418 | 4.88E-191 | -2.35061 | 0.998 | 1 | 1.51E-186 | 0 |
| Lars2 | 7.47E-168 | -1.87042 | 0.277 | 0.82 | 2.32E-163 | 0 |
| Rps27 | 7.79E-141 | 0.438733 | 1 | 0.998 | 2.42E-136 | 0 |
| Apoe | 1.04E-140 | 1.053484 | 0.95 | 0.513 | 3.24E-136 | 0 |
| Uba52 | 6.78E-115 | 0.829937 | 0.958 | 0.858 | 2.11E-110 | 0 |
| Fth1 | 2.94E-94 | 0.46259 | 1 | 0.998 | 9.13E-90 | 0 |
| Rps29 | 2.46E-90 | 0.310772 | 1 | 0.998 | 7.65E-86 | 0 |
| Rpl39 | 6.32E-89 | 0.380522 | 1 | 0.999 | 1.96E-84 | 0 |
| Rpl35 | 2.24E-86 | 0.449875 | 0.993 | 0.982 | 6.96E-82 | 0 |
| Saa3 | 2.10E-81 | 0.752143 | 0.932 | 0.526 | 6.51E-77 | 0 |
| Rps24 | 2.31E-76 | 0.261243 | 1 | 1 | 7.19E-72 | 0 |
| Rps15a | 9.96E-73 | 0.296964 | 1 | 0.999 | 3.09E-68 | 0 |
| Ftl1 | 1.17E-71 | 0.465365 | 0.992 | 0.971 | 3.63E-67 | 0 |
| Rps21 | 7.08E-64 | 0.30024 | 1 | 0.995 | 2.20E-59 | 0 |
| Fau | 1.90E-60 | 0.259907 | 0.999 | 1 | 5.89E-56 | 0 |
| AY036118 | 2.63E-59 | -0.6199 | 0.707 | 0.919 | 8.17E-55 | 0 |
| Ctsd | 1.87E-52 | 0.833617 | 0.569 | 0.289 | 5.79E-48 | 0 |
| Rpl38 | 1.92E-51 | 0.272462 | 1 | 0.999 | 5.97E-47 | 0 |
| Rpl37a | 1.06E-49 | 0.240061 | 1 | 0.999 | 3.30E-45 | 0 |
| Rps25 | 3.65E-49 | 0.268502 | 0.997 | 0.995 | 1.13E-44 | 0 |
| Wfdc17 | 1.14E-48 | 0.741029 | 0.631 | 0.321 | 3.53E-44 | 0 |
| Rpl36 | 7.55E-48 | 0.276377 | 0.998 | 0.99 | 2.34E-43 | 0 |
| Ubb | 5.36E-47 | 0.316448 | 0.993 | 0.986 | 1.66E-42 | 0 |
| Rpl21 | 1.40E-41 | 0.232988 | 1 | 0.995 | 4.33E-37 | 0 |
| Macf1 | 3.54E-41 | -0.60917 | 0.449 | 0.733 | 1.10E-36 | 0 |
| Rpl34 | 1.50E-39 | 0.213286 | 1 | 0.996 | 4.67E-35 | 0 |
| Ighm | 4.61E-39 | -1.02085 | 0.951 | 0.984 | 1.43E-34 | 0 |
| Rps4x | 6.92E-39 | 0.231675 | 1 | 0.999 | 2.15E-34 | 0 |
| Rpl12 | 1.49E-38 | 0.232145 | 0.999 | 0.996 | 4.63E-34 | 0 |
| mt-Co1 | 3.16E-38 | 0.200397 | 0.999 | 1 | 9.81E-34 | 0 |
| Rpl26 | 1.89E-36 | 0.21397 | 0.999 | 0.999 | 5.88E-32 | 0 |
| S100a8 | 7.34E-36 | 0.556014 | 0.767 | 0.535 | 2.28E-31 | 0 |
| Fabp5 | 8.17E-33 | 0.734701 | 0.293 | 0.086 | 2.54E-28 | 0 |
| Lgals3 | 1.25E-32 | 0.646943 | 0.424 | 0.198 | 3.88E-28 | 0 |
| Rps3a1 | 3.05E-32 | 0.180388 | 1 | 0.999 | 9.47E-28 | 0 |
| Rpl23 | 8.57E-31 | 0.1779 | 0.999 | 0.998 | 2.66E-26 | 0 |
| Rps26 | 5.24E-30 | 0.22493 | 0.993 | 0.996 | 1.63E-25 | 0 |
| Rps10 | 6.16E-29 | 0.170872 | 0.999 | 0.998 | 1.91E-24 | 0 |
| mt-Nd2 | 6.82E-29 | -0.23739 | 0.971 | 0.993 | 2.12E-24 | 0 |
| Rpl36a | 1.16E-28 | 0.269082 | 0.985 | 0.981 | 3.59E-24 | 0 |
| Rpl37 | 1.28E-28 | 0.223032 | 0.997 | 0.996 | 3.96E-24 | 0 |
| Rpl30 | 1.54E-28 | 0.182917 | 0.999 | 0.999 | 4.78E-24 | 0 |
| Rplp2 | 1.72E-28 | 0.172389 | 1 | 1 | 5.35E-24 | 0 |
| Rps27a | 3.70E-28 | 0.184627 | 1 | 0.998 | 1.15E-23 | 0 |
| Hist1h1e | 8.83E-27 | -0.49228 | 0.047 | 0.208 | 2.74E-22 | 0 |
| Rpl27a | 4.68E-26 | 0.165746 | 0.999 | 1 | 1.45E-21 | 0 |
| B2m | 1.24E-25 | 0.261091 | 0.969 | 0.969 | 3.84E-21 | 0 |
| Tpr | 1.44E-25 | -0.37989 | 0.346 | 0.629 | 4.47E-21 | 0 |
| mt-Atp6 | 2.06E-25 | 0.146277 | 1 | 1 | 6.40E-21 | 0 |
| Hspa1b | 6.17E-25 | 0.703049 | 0.393 | 0.184 | 1.92E-20 | 0 |
| Swap70 | 8.08E-25 | -0.40806 | 0.194 | 0.434 | 2.51E-20 | 0 |
| Tyrobp | 1.52E-24 | 0.606661 | 0.485 | 0.313 | 4.73E-20 | 0 |
| Rpl35a | 2.75E-24 | 0.149798 | 1 | 0.999 | 8.53E-20 | 0 |
| C1qb | 2.88E-24 | 0.50333 | 0.181 | 0.033 | 8.93E-20 | 0 |
| Rps16 | 3.05E-24 | 0.147063 | 0.999 | 0.999 | 9.46E-20 | 0 |
| Psap | 3.79E-22 | 0.481061 | 0.635 | 0.542 | 1.18E-17 | 0 |
| mt-Co3 | 7.35E-22 | 0.12619 | 1 | 1 | 2.28E-17 | 0 |
| Ier3 | 1.57E-21 | 0.560961 | 0.33 | 0.156 | 4.87E-17 | 0 |
| Cebpb | 2.66E-21 | 0.42858 | 0.707 | 0.584 | 8.26E-17 | 0 |
| Malat1 | 2.78E-21 | -0.20302 | 0.997 | 0.999 | 8.62E-17 | 0 |
| Cxcl2 | 3.27E-21 | 0.451663 | 0.662 | 0.473 | 1.01E-16 | 0 |
| Prpf4b | 3.62E-21 | -0.31076 | 0.293 | 0.557 | 1.12E-16 | 0 |
| 9930111J21Rik2 | 4.07E-21 | -0.36856 | 0.116 | 0.297 | 1.26E-16 | 0 |
| Rps20 | 1.04E-20 | 0.158187 | 1 | 0.999 | 3.24E-16 | 0 |
| Rps23 | 1.52E-20 | 0.158973 | 1 | 0.996 | 4.73E-16 | 0 |
| Iglc3 | 2.42E-19 | -0.3927 | 0.429 | 0.662 | 7.53E-15 | 0 |
| Ncl | 2.49E-19 | -0.33539 | 0.587 | 0.819 | 7.74E-15 | 0 |
| Gm10076 | 8.63E-19 | 0.461832 | 0.499 | 0.368 | 2.68E-14 | 0 |
| Spp1 | 9.83E-19 | 0.434627 | 0.154 | 0.033 | 3.05E-14 | 0 |
| Tmsb10 | 1.82E-18 | 0.256106 | 0.964 | 0.97 | 5.66E-14 | 0 |
| Ltb | 1.88E-18 | -0.39108 | 0.377 | 0.607 | 5.85E-14 | 0 |
| S100a9 | 2.16E-18 | 0.403608 | 0.694 | 0.539 | 6.71E-14 | 0 |
| Sptbn1 | 2.60E-18 | -0.31003 | 0.22 | 0.432 | 8.09E-14 | 0 |
| Cxcr4 | 2.98E-18 | 0.442431 | 0.733 | 0.672 | 9.26E-14 | 0 |
| Ddx21 | 4.12E-18 | -0.28543 | 0.198 | 0.406 | 1.28E-13 | 0 |
| Eif3c | 1.09E-17 | -0.30448 | 0.277 | 0.498 | 3.37E-13 | 0 |
| Ms4a1 | 1.15E-17 | -0.35293 | 0.652 | 0.813 | 3.57E-13 | 0 |
| Rpl8 | 1.73E-17 | 0.1468 | 0.999 | 0.999 | 5.36E-13 | 0 |
| Rpl9 | 3.83E-17 | 0.13626 | 0.998 | 0.998 | 1.19E-12 | 0 |
| C1qa | 6.08E-17 | 0.369021 | 0.123 | 0.02 | 1.89E-12 | 0 |
| Rpl18a | 7.45E-17 | 0.12364 | 1 | 0.998 | 2.31E-12 | 0 |
| Dock8 | 8.90E-17 | -0.24279 | 0.123 | 0.289 | 2.76E-12 | 0 |
| Kat6a | 9.80E-17 | -0.2923 | 0.123 | 0.286 | 3.04E-12 | 0 |
| Rps19 | 1.40E-16 | 0.128633 | 1 | 1 | 4.33E-12 | 0 |
| Mycbp2 | 1.48E-16 | -0.30134 | 0.355 | 0.585 | 4.60E-12 | 0 |
| Prrc2c | 2.04E-16 | -0.26192 | 0.452 | 0.692 | 6.32E-12 | 0 |
| Rpl28 | 2.24E-16 | 0.152701 | 0.999 | 0.998 | 6.95E-12 | 0 |
| Ptpn6 | 3.89E-16 | -0.30007 | 0.299 | 0.514 | 1.21E-11 | 0 |
| Pdia3 | 4.26E-16 | -0.27183 | 0.183 | 0.374 | 1.32E-11 | 0 |
| Rpl32 | 9.74E-16 | 0.139017 | 1 | 0.998 | 3.02E-11 | 0 |
| Ly6e | 1.01E-15 | -0.21318 | 0.917 | 0.973 | 3.14E-11 | 0 |
| Rpl11 | 1.07E-15 | 0.149125 | 0.998 | 0.998 | 3.31E-11 | 0 |
| Map4k1 | 1.30E-15 | -0.25 | 0.133 | 0.297 | 4.05E-11 | 0 |
| Cstb | 1.32E-15 | 0.47891 | 0.436 | 0.303 | 4.10E-11 | 0 |
| Myo1e | 1.41E-15 | -0.27425 | 0.15 | 0.323 | 4.39E-11 | 0 |
| Fn1 | 1.90E-15 | 0.392592 | 0.156 | 0.045 | 5.90E-11 | 0 |
| mt-Atp8 | 2.10E-15 | -0.24694 | 0.214 | 0.411 | 6.52E-11 | 0 |
| Ikzf3 | 4.79E-15 | -0.23147 | 0.166 | 0.344 | 1.49E-10 | 0 |
| Il1b | 5.06E-15 | 0.367188 | 0.536 | 0.397 | 1.57E-10 | 0 |
| Fcer1g | 8.21E-15 | 0.494663 | 0.341 | 0.205 | 2.55E-10 | 0 |
| Rpl17 | 9.32E-15 | 0.127435 | 1 | 0.999 | 2.90E-10 | 0 |
| Dnajc7 | 1.54E-14 | -0.25081 | 0.227 | 0.422 | 4.77E-10 | 0 |
| Gm8369 | 1.54E-14 | 0.450464 | 0.652 | 0.585 | 4.79E-10 | 0 |
| S100a6 | 1.67E-14 | -0.40174 | 0.489 | 0.304 | 5.17E-10 | 0 |
| H2-K1 | 1.73E-14 | 0.22494 | 0.948 | 0.958 | 5.38E-10 | 0 |
| Ddx6 | 1.82E-14 | -0.29828 | 0.46 | 0.667 | 5.66E-10 | 0 |
| Rfc1 | 1.93E-14 | -0.24404 | 0.095 | 0.232 | 6.00E-10 | 0 |
| Rpl10 | 2.15E-14 | 0.187095 | 0.994 | 0.984 | 6.69E-10 | 0 |
| Rps7 | 2.41E-14 | 0.12653 | 1 | 0.999 | 7.49E-10 | 0 |
| Ly6c2 | 2.66E-14 | 0.4081 | 0.198 | 0.078 | 8.25E-10 | 0 |
| Trip11 | 2.84E-14 | -0.22827 | 0.121 | 0.27 | 8.81E-10 | 0 |
| Vars | 3.36E-14 | -0.2655 | 0.233 | 0.42 | 1.04E-09 | 0 |
| Hspa1a | 3.55E-14 | 0.309893 | 0.328 | 0.173 | 1.10E-09 | 0 |
| Rpl29 | 4.28E-14 | 0.174475 | 0.975 | 0.968 | 1.33E-09 | 0 |
| Ptprj | 4.49E-14 | -0.20963 | 0.109 | 0.252 | 1.39E-09 | 0 |
| Trem2 | 5.06E-14 | 0.331811 | 0.113 | 0.024 | 1.57E-09 | 0 |
| Pdia6 | 5.10E-14 | -0.2704 | 0.181 | 0.354 | 1.58E-09 | 0 |
| Rps8 | 5.34E-14 | 0.109367 | 1 | 1 | 1.66E-09 | 0 |
| Tnfrsf13b | 6.05E-14 | -0.22411 | 0.124 | 0.273 | 1.88E-09 | 0 |
| Pou2f2 | 6.28E-14 | -0.28294 | 0.396 | 0.605 | 1.95E-09 | 0 |
| Dnaja1 | 6.39E-14 | 0.388942 | 0.716 | 0.708 | 1.99E-09 | 0 |
| Prdx1 | 7.55E-14 | 0.3427 | 0.613 | 0.558 | 2.34E-09 | 0 |
| Arhgap25 | 7.57E-14 | -0.20929 | 0.14 | 0.295 | 2.35E-09 | 0 |
| Hltf | 8.22E-14 | -0.17443 | 0.051 | 0.159 | 2.55E-09 | 0 |
| Pax5 | 8.50E-14 | -0.26851 | 0.396 | 0.603 | 2.64E-09 | 0 |
| Rps13 | 9.22E-14 | 0.122867 | 0.999 | 0.994 | 2.86E-09 | 0 |
| Ralgps2 | 1.20E-13 | -0.28348 | 0.448 | 0.674 | 3.74E-09 | 0 |
| Hist1h4d | 1.41E-13 | -0.20122 | 0.022 | 0.103 | 4.39E-09 | 0 |
| Tln1 | 1.76E-13 | -0.25062 | 0.268 | 0.465 | 5.47E-09 | 0 |
| Eif3a | 2.09E-13 | -0.27116 | 0.322 | 0.521 | 6.49E-09 | 0 |
| Ptprc | 3.71E-13 | -0.25162 | 0.665 | 0.829 | 1.15E-08 | 0 |
| Mgea5 | 4.95E-13 | -0.21405 | 0.107 | 0.24 | 1.54E-08 | 0 |
| Hdlbp | 5.18E-13 | -0.18001 | 0.105 | 0.241 | 1.61E-08 | 0 |
| Glg1 | 5.30E-13 | -0.2184 | 0.108 | 0.242 | 1.64E-08 | 0 |
| Kmt2a | 5.31E-13 | -0.22915 | 0.316 | 0.521 | 1.65E-08 | 0 |
| Mrpl12 | 5.44E-13 | -0.18814 | 0.074 | 0.191 | 1.69E-08 | 0 |
| Klf13 | 6.27E-13 | -0.19873 | 0.249 | 0.442 | 1.95E-08 | 0 |
| Rps6 | 8.12E-13 | 0.148459 | 0.991 | 0.992 | 2.52E-08 | 0 |
| S1pr1 | 9.50E-13 | -0.23233 | 0.177 | 0.337 | 2.95E-08 | 0 |
| Nedd9 | 1.05E-12 | -0.21672 | 0.138 | 0.284 | 3.26E-08 | 0 |
| Usp24 | 1.19E-12 | -0.19788 | 0.089 | 0.211 | 3.70E-08 | 0 |
| Plaur | 1.22E-12 | 0.401113 | 0.548 | 0.451 | 3.78E-08 | 0 |
| Ly6d | 1.29E-12 | -0.28161 | 0.759 | 0.888 | 4.00E-08 | 0 |
| Trim44 | 1.29E-12 | -0.2006 | 0.105 | 0.236 | 4.01E-08 | 0 |
| mt-Nd3 | 1.34E-12 | -0.18903 | 0.796 | 0.901 | 4.15E-08 | 0 |
| Samd9l | 1.36E-12 | -0.25043 | 0.187 | 0.348 | 4.22E-08 | 0 |
| Cct5 | 1.36E-12 | -0.21302 | 0.262 | 0.462 | 4.22E-08 | 0 |
| Dock2 | 1.45E-12 | -0.22674 | 0.341 | 0.541 | 4.50E-08 | 0 |
| Ppp4r3a | 1.58E-12 | -0.20753 | 0.137 | 0.28 | 4.92E-08 | 0 |
| Eprs | 1.76E-12 | -0.2207 | 0.159 | 0.311 | 5.47E-08 | 0 |
| Tmod3 | 1.78E-12 | -0.2324 | 0.346 | 0.536 | 5.54E-08 | 0 |
| Nsd1 | 1.82E-12 | -0.19852 | 0.251 | 0.447 | 5.66E-08 | 0 |
| Tpt1 | 2.00E-12 | 0.089164 | 1 | 1 | 6.22E-08 | 0 |
| Nipbl | 2.44E-12 | -0.25101 | 0.31 | 0.501 | 7.58E-08 | 0 |
| Tmsb4x | 2.47E-12 | 0.131879 | 1 | 1 | 7.68E-08 | 0 |
| Aff3 | 2.55E-12 | -0.2058 | 0.094 | 0.217 | 7.91E-08 | 0 |
| Cd22 | 2.64E-12 | -0.24859 | 0.252 | 0.431 | 8.19E-08 | 0 |
| Rpl31 | 2.93E-12 | 0.214358 | 0.914 | 0.908 | 9.09E-08 | 0 |
| Mt1 | 3.45E-12 | 0.416269 | 0.181 | 0.08 | 1.07E-07 | 0 |
| Cd14 | 3.58E-12 | 0.441541 | 0.291 | 0.171 | 1.11E-07 | 0 |
| Usp34 | 3.89E-12 | -0.21072 | 0.22 | 0.396 | 1.21E-07 | 0 |
| Mia2 | 4.33E-12 | -0.20452 | 0.127 | 0.264 | 1.34E-07 | 0 |
| Arid4a | 4.81E-12 | -0.18635 | 0.174 | 0.332 | 1.49E-07 | 0 |
| Myh9 | 4.84E-12 | -0.23599 | 0.354 | 0.549 | 1.50E-07 | 0 |
| Sptan1 | 4.93E-12 | -0.17909 | 0.123 | 0.261 | 1.53E-07 | 0 |
| Mndal | 5.13E-12 | -0.24672 | 0.387 | 0.593 | 1.59E-07 | 0 |
| Trp53inp1 | 6.06E-12 | -0.2156 | 0.097 | 0.22 | 1.88E-07 | 0 |
| Mga | 6.40E-12 | -0.24607 | 0.133 | 0.266 | 1.99E-07 | 0 |
| Lcorl | 7.09E-12 | -0.19528 | 0.056 | 0.154 | 2.20E-07 | 0 |
| Csnk2a2 | 7.12E-12 | -0.1512 | 0.1 | 0.226 | 2.21E-07 | 0 |
| Ankrd11 | 7.27E-12 | -0.25832 | 0.481 | 0.668 | 2.26E-07 | 0 |
| Rpl24 | 7.58E-12 | 0.150699 | 0.988 | 0.987 | 2.35E-07 | 0 |
| Mtdh | 7.69E-12 | -0.21376 | 0.349 | 0.551 | 2.39E-07 | 0 |
| Spag9 | 8.37E-12 | -0.1941 | 0.233 | 0.409 | 2.60E-07 | 0 |
| Cecr2 | 8.38E-12 | -0.19065 | 0.039 | 0.125 | 2.60E-07 | 0 |
| Xrn1 | 8.42E-12 | -0.20939 | 0.099 | 0.22 | 2.62E-07 | 0 |
| Diaph1 | 8.72E-12 | -0.19096 | 0.183 | 0.344 | 2.71E-07 | 0 |
| Itgb7 | 9.26E-12 | -0.20711 | 0.099 | 0.22 | 2.88E-07 | 0 |
| Cep350 | 9.42E-12 | -0.17426 | 0.096 | 0.22 | 2.92E-07 | 0 |
| Ap2b1 | 1.06E-11 | -0.16203 | 0.112 | 0.242 | 3.29E-07 | 0 |
| Sik1 | 1.14E-11 | -0.19548 | 0.24 | 0.418 | 3.54E-07 | 0 |
| Pdia4 | 1.27E-11 | -0.23433 | 0.119 | 0.247 | 3.93E-07 | 0 |
| Calr | 1.48E-11 | -0.2665 | 0.498 | 0.702 | 4.59E-07 | 0 |
| Rock1 | 1.50E-11 | -0.21125 | 0.292 | 0.483 | 4.67E-07 | 0 |
| Rasa3 | 1.70E-11 | -0.21234 | 0.169 | 0.322 | 5.28E-07 | 0 |
| Rps11 | 1.78E-11 | 0.134475 | 0.998 | 0.996 | 5.52E-07 | 0 |
| Rbm3 | 1.83E-11 | 0.283338 | 0.712 | 0.673 | 5.68E-07 | 0 |
| Smap2 | 1.90E-11 | -0.19132 | 0.365 | 0.591 | 5.91E-07 | 0 |
| Numa1 | 2.06E-11 | -0.17787 | 0.131 | 0.267 | 6.39E-07 | 0 |
| Atf6 | 2.27E-11 | -0.17324 | 0.087 | 0.2 | 7.04E-07 | 0 |
| Dnajc3 | 2.49E-11 | -0.17554 | 0.114 | 0.242 | 7.72E-07 | 0 |
| U2surp | 2.66E-11 | -0.20397 | 0.185 | 0.34 | 8.25E-07 | 0 |
| Wdr43 | 3.34E-11 | -0.17028 | 0.105 | 0.229 | 1.04E-06 | 0 |
| Cdv3 | 3.47E-11 | -0.1857 | 0.21 | 0.383 | 1.08E-06 | 0 |
| Pabpc4 | 3.78E-11 | -0.13823 | 0.046 | 0.134 | 1.17E-06 | 0 |
| Acbd3 | 3.79E-11 | -0.18613 | 0.113 | 0.241 | 1.18E-06 | 0 |
| Iglc2 | 4.18E-11 | -1.02906 | 0.506 | 0.668 | 1.30E-06 | 0 |
| Cmah | 4.35E-11 | -0.18421 | 0.229 | 0.402 | 1.35E-06 | 0 |
| C130026I21Rik | 4.44E-11 | -0.13103 | 0.056 | 0.15 | 1.38E-06 | 0 |
| Zfp62 | 4.83E-11 | -0.16768 | 0.061 | 0.158 | 1.50E-06 | 0 |
| Paxbp1 | 5.09E-11 | -0.17001 | 0.087 | 0.199 | 1.58E-06 | 0 |
| Tet2 | 5.13E-11 | -0.13975 | 0.075 | 0.181 | 1.59E-06 | 0 |
| Chd1 | 5.33E-11 | -0.17669 | 0.139 | 0.277 | 1.65E-06 | 0 |
| Rnf10 | 5.39E-11 | -0.1532 | 0.109 | 0.235 | 1.67E-06 | 0 |
| Rnf213 | 5.40E-11 | -0.22256 | 0.053 | 0.143 | 1.68E-06 | 0 |
| Hnrnpul1 | 5.85E-11 | -0.13812 | 0.13 | 0.267 | 1.82E-06 | 0 |
| Iglc1 | 5.89E-11 | -0.38743 | 0.198 | 0.34 | 1.83E-06 | 0 |
| Phgdh | 6.27E-11 | -0.16145 | 0.081 | 0.189 | 1.95E-06 | 0 |
| Cdc37 | 6.58E-11 | -0.16375 | 0.186 | 0.346 | 2.04E-06 | 0 |
| Mybbp1a | 6.60E-11 | -0.1678 | 0.098 | 0.215 | 2.05E-06 | 0 |
| Smarca5 | 7.70E-11 | -0.18209 | 0.32 | 0.519 | 2.39E-06 | 0 |
| Gm47283 | 7.90E-11 | -0.17079 | 0.185 | 0.34 | 2.45E-06 | 0 |
| Huwe1 | 8.11E-11 | -0.18062 | 0.211 | 0.375 | 2.52E-06 | 0 |
| Akap13 | 8.50E-11 | -0.23589 | 0.517 | 0.7 | 2.64E-06 | 0 |
| Kdm5b | 8.54E-11 | -0.14479 | 0.051 | 0.142 | 2.65E-06 | 0 |
| Ccnd2 | 8.80E-11 | -0.26246 | 0.094 | 0.203 | 2.73E-06 | 0 |
| Fkbp2 | 8.99E-11 | -0.17784 | 0.088 | 0.198 | 2.79E-06 | 0 |
| Lyst | 9.43E-11 | -0.18703 | 0.147 | 0.283 | 2.93E-06 | 0 |
| Supt16 | 1.03E-10 | -0.22074 | 0.113 | 0.233 | 3.21E-06 | 0 |
| Zfp706 | 1.04E-10 | -0.20299 | 0.273 | 0.453 | 3.23E-06 | 0 |
| Sf3a3 | 1.05E-10 | -0.13183 | 0.065 | 0.165 | 3.25E-06 | 0 |
| Rasgrp1 | 1.08E-10 | -0.15837 | 0.065 | 0.163 | 3.35E-06 | 0 |
| Gm20186 | 1.25E-10 | -0.11265 | 0.036 | 0.116 | 3.88E-06 | 0 |
| Dnajc2 | 1.31E-10 | -0.15652 | 0.141 | 0.277 | 4.07E-06 | 0 |
| Purb | 1.48E-10 | -0.21012 | 0.315 | 0.507 | 4.58E-06 | 0 |
| Mdn1 | 1.65E-10 | -0.19471 | 0.073 | 0.172 | 5.14E-06 | 0 |
| Cacna1e | 1.71E-10 | -0.13325 | 0.037 | 0.116 | 5.32E-06 | 0 |
| Eef1g | 1.75E-10 | -0.20023 | 0.71 | 0.848 | 5.42E-06 | 0 |
| Kdm5a | 1.80E-10 | -0.14536 | 0.217 | 0.385 | 5.60E-06 | 0 |
| Setx | 1.82E-10 | -0.17003 | 0.109 | 0.228 | 5.64E-06 | 0 |
| Acp5 | 1.94E-10 | -0.2331 | 0.254 | 0.424 | 6.01E-06 | 0 |
| Tram1 | 1.95E-10 | -0.16082 | 0.15 | 0.289 | 6.06E-06 | 0 |
| Zfp106 | 2.07E-10 | -0.18858 | 0.216 | 0.378 | 6.41E-06 | 0 |
| Smc1a | 2.08E-10 | -0.17499 | 0.158 | 0.301 | 6.45E-06 | 0 |
| Pdap1 | 2.13E-10 | -0.2024 | 0.121 | 0.24 | 6.61E-06 | 0 |
| Bdp1 | 2.13E-10 | -0.15776 | 0.109 | 0.229 | 6.62E-06 | 0 |
| Ptk2b | 2.38E-10 | -0.15068 | 0.201 | 0.358 | 7.39E-06 | 0 |
| Arpc5l | 2.61E-10 | -0.18137 | 0.262 | 0.439 | 8.11E-06 | 0 |
| Med13 | 2.68E-10 | -0.20739 | 0.266 | 0.434 | 8.33E-06 | 0 |
| Taok3 | 2.71E-10 | -0.12345 | 0.142 | 0.278 | 8.43E-06 | 0 |
| Abce1 | 2.80E-10 | -0.13839 | 0.085 | 0.191 | 8.69E-06 | 0 |
| Smarcb1 | 2.82E-10 | -0.12803 | 0.072 | 0.172 | 8.76E-06 | 0 |
| Chd3 | 3.17E-10 | -0.19849 | 0.141 | 0.267 | 9.85E-06 | 0 |
| Gpbp1l1 | 3.32E-10 | -0.18405 | 0.111 | 0.229 | 1.03E-05 | 0 |
| Tars | 3.49E-10 | -0.1301 | 0.033 | 0.106 | 1.09E-05 | 0 |
| Ebf1 | 3.68E-10 | 0.177445 | 0.955 | 0.959 | 1.14E-05 | 0 |
| Hspa5 | 3.73E-10 | -0.31525 | 0.58 | 0.747 | 1.16E-05 | 0 |
| Akap9 | 3.82E-10 | -0.19048 | 0.153 | 0.285 | 1.19E-05 | 0 |
| Hsp90b1 | 3.91E-10 | -0.38056 | 0.446 | 0.634 | 1.21E-05 | 0 |
| Pml | 4.16E-10 | -0.17607 | 0.092 | 0.198 | 1.29E-05 | 0 |
| Golga4 | 4.35E-10 | -0.17355 | 0.09 | 0.196 | 1.35E-05 | 0 |
| March1 | 4.43E-10 | -0.19706 | 0.235 | 0.391 | 1.38E-05 | 0 |
| Baz1b | 4.55E-10 | -0.14861 | 0.159 | 0.298 | 1.41E-05 | 0 |
| Foxo1 | 4.90E-10 | -0.18395 | 0.246 | 0.408 | 1.52E-05 | 0 |
| Aco2 | 5.49E-10 | -0.11938 | 0.088 | 0.195 | 1.71E-05 | 0 |
| Nme1 | 5.58E-10 | -0.25425 | 0.245 | 0.4 | 1.73E-05 | 0 |
| Nars | 5.65E-10 | -0.13223 | 0.092 | 0.2 | 1.75E-05 | 0 |
| Suco | 6.04E-10 | -0.13672 | 0.08 | 0.184 | 1.87E-05 | 0 |
| Birc6 | 7.04E-10 | -0.13984 | 0.252 | 0.427 | 2.19E-05 | 0 |
| Stk4 | 7.19E-10 | -0.17724 | 0.21 | 0.359 | 2.23E-05 | 0 |
| Grb2 | 7.30E-10 | -0.2069 | 0.345 | 0.533 | 2.27E-05 | 0 |
| Ifi27l2a | 7.56E-10 | -0.31727 | 0.269 | 0.425 | 2.35E-05 | 0 |
| Brwd1 | 7.65E-10 | -0.15406 | 0.123 | 0.245 | 2.38E-05 | 0 |
| Lcp1 | 8.54E-10 | -0.21797 | 0.495 | 0.673 | 2.65E-05 | 0 |
| Rpl23a | 8.54E-10 | 0.138519 | 0.978 | 0.974 | 2.65E-05 | 0 |
| Nup50 | 8.62E-10 | -0.13445 | 0.066 | 0.16 | 2.68E-05 | 0 |
| Riok1 | 8.68E-10 | -0.13886 | 0.08 | 0.18 | 2.70E-05 | 0 |
| Napsa | 8.88E-10 | -0.20678 | 0.566 | 0.735 | 2.76E-05 | 0 |
| Dync1h1 | 8.92E-10 | -0.16115 | 0.118 | 0.236 | 2.77E-05 | 0 |
| Calm1 | 9.06E-10 | -0.17832 | 0.775 | 0.912 | 2.81E-05 | 0 |
| Nfkbia | 9.09E-10 | -0.20697 | 0.503 | 0.696 | 2.82E-05 | 0 |
| Itpr2 | 9.54E-10 | -0.16807 | 0.143 | 0.268 | 2.96E-05 | 0 |
| Eif4g1 | 9.89E-10 | -0.13941 | 0.148 | 0.282 | 3.07E-05 | 0 |
| Dbnl | 1.01E-09 | -0.14124 | 0.16 | 0.298 | 3.13E-05 | 0 |
| Rdx | 1.02E-09 | -0.14546 | 0.141 | 0.268 | 3.18E-05 | 0 |
| Traf5 | 1.09E-09 | -0.15924 | 0.097 | 0.205 | 3.38E-05 | 0 |
| Hnrnpc | 1.14E-09 | -0.18838 | 0.366 | 0.556 | 3.53E-05 | 0 |
| Hnrnpul2 | 1.14E-09 | -0.16538 | 0.191 | 0.34 | 3.55E-05 | 0 |
| Rrp1 | 1.19E-09 | -0.14719 | 0.17 | 0.311 | 3.71E-05 | 0 |
| Klf2 | 1.20E-09 | -0.31833 | 0.474 | 0.667 | 3.73E-05 | 0 |
| S100a4 | 1.29E-09 | 0.275951 | 0.205 | 0.107 | 4.00E-05 | 0 |
| Ktn1 | 1.36E-09 | -0.20051 | 0.164 | 0.295 | 4.24E-05 | 0 |
| Rbm34 | 1.37E-09 | -0.14753 | 0.059 | 0.146 | 4.25E-05 | 0 |
| Eif3d | 1.40E-09 | -0.1646 | 0.151 | 0.279 | 4.33E-05 | 0 |
| Pik3cd | 1.44E-09 | -0.13209 | 0.126 | 0.248 | 4.49E-05 | 0 |
| Sars | 1.48E-09 | -0.11435 | 0.112 | 0.229 | 4.61E-05 | 0 |
| Prdm2 | 1.51E-09 | -0.17318 | 0.133 | 0.253 | 4.70E-05 | 0 |
| Parp1 | 1.57E-09 | -0.17332 | 0.222 | 0.379 | 4.88E-05 | 0 |
| Rpl5 | 1.58E-09 | 0.115485 | 0.993 | 0.993 | 4.92E-05 | 0 |
| Kmt5b | 1.62E-09 | -0.161 | 0.127 | 0.247 | 5.04E-05 | 0 |
| Wdfy4 | 1.63E-09 | -0.13844 | 0.162 | 0.301 | 5.05E-05 | 0 |
| Abcf1 | 1.64E-09 | -0.10444 | 0.127 | 0.252 | 5.09E-05 | 0 |
| Zfp407 | 1.68E-09 | -0.13302 | 0.036 | 0.109 | 5.23E-05 | 0 |
| Cabin1 | 1.69E-09 | -0.11532 | 0.064 | 0.153 | 5.23E-05 | 0 |
| Denr | 1.89E-09 | -0.15673 | 0.112 | 0.224 | 5.88E-05 | 0 |
| Specc1l | 1.95E-09 | -0.11856 | 0.08 | 0.181 | 6.04E-05 | 0 |
| Rps5 | 1.95E-09 | 0.108258 | 0.999 | 0.995 | 6.07E-05 | 0 |
| Rbm27 | 2.04E-09 | -0.14848 | 0.123 | 0.241 | 6.34E-05 | 0 |
| Suz12 | 2.06E-09 | -0.15385 | 0.117 | 0.232 | 6.41E-05 | 0 |
| Zfp644 | 2.08E-09 | -0.18648 | 0.198 | 0.34 | 6.47E-05 | 0 |
| Itsn2 | 2.16E-09 | -0.1837 | 0.251 | 0.406 | 6.69E-05 | 0 |
| Strn3 | 2.17E-09 | -0.12884 | 0.138 | 0.261 | 6.73E-05 | 0 |
| Top2b | 2.27E-09 | -0.16787 | 0.264 | 0.436 | 7.05E-05 | 0 |
| Zfp148 | 2.32E-09 | -0.12877 | 0.128 | 0.251 | 7.22E-05 | 0 |
| Heatr6 | 2.48E-09 | -0.15652 | 0.05 | 0.129 | 7.70E-05 | 0 |
| Smc3 | 2.48E-09 | -0.16977 | 0.175 | 0.31 | 7.71E-05 | 0 |
| Ep400 | 2.49E-09 | -0.13581 | 0.167 | 0.304 | 7.73E-05 | 0 |
| Rbck1 | 2.57E-09 | -0.10872 | 0.081 | 0.18 | 7.99E-05 | 0 |
| Serbp1 | 2.63E-09 | -0.20224 | 0.589 | 0.771 | 8.17E-05 | 0 |
| Iws1 | 2.87E-09 | -0.11883 | 0.106 | 0.216 | 8.90E-05 | 0 |
| Stat1 | 2.91E-09 | -0.22183 | 0.172 | 0.302 | 9.04E-05 | 0 |
| Arfgef1 | 2.92E-09 | -0.16302 | 0.191 | 0.332 | 9.06E-05 | 0 |
| 1700017B05Rik | 3.03E-09 | -0.13709 | 0.099 | 0.205 | 9.42E-05 | 0 |
| Unc93b1 | 3.05E-09 | -0.18632 | 0.395 | 0.586 | 9.47E-05 | 0 |
| Cd63 | 3.20E-09 | 0.355184 | 0.15 | 0.068 | 9.95E-05 | 0 |
| Siglecg | 3.24E-09 | -0.20948 | 0.321 | 0.489 | 0.000101 | 0 |
| Ankib1 | 3.25E-09 | -0.14832 | 0.061 | 0.146 | 0.000101 | 0 |
| AW112010 | 3.35E-09 | 0.417309 | 0.266 | 0.169 | 0.000104 | 0 |
| Rpl6 | 3.42E-09 | 0.117366 | 0.993 | 0.994 | 0.000106 | 0 |
| Bin2 | 3.48E-09 | -0.13209 | 0.103 | 0.21 | 0.000108 | 0 |
| Mrpl57 | 3.51E-09 | -0.12627 | 0.109 | 0.221 | 0.000109 | 0 |
| Trabd | 3.61E-09 | -0.13148 | 0.07 | 0.16 | 0.000112 | 0 |
| Sbk1 | 3.72E-09 | -0.14213 | 0.09 | 0.191 | 0.000115 | 0 |
| Chd4 | 3.72E-09 | -0.19869 | 0.333 | 0.502 | 0.000116 | 0 |
| Rpsa | 3.92E-09 | 0.098364 | 0.998 | 1 | 0.000122 | 0 |
| Kdm1a | 4.17E-09 | -0.11585 | 0.109 | 0.221 | 0.00013 | 0 |
| Itpr1 | 4.23E-09 | -0.16982 | 0.148 | 0.271 | 0.000131 | 0 |
| Scaf11 | 4.41E-09 | -0.17419 | 0.378 | 0.551 | 0.000137 | 0 |
| Cntrl | 4.61E-09 | -0.13536 | 0.144 | 0.267 | 0.000143 | 0 |
| Sun2 | 4.70E-09 | -0.122 | 0.126 | 0.245 | 0.000146 | 0 |
| Psme2 | 5.09E-09 | -0.19517 | 0.175 | 0.305 | 0.000158 | 0 |
| Gpnmb | 5.13E-09 | 0.282028 | 0.106 | 0.036 | 0.000159 | 0 |
| Smarcc1 | 5.41E-09 | -0.12368 | 0.073 | 0.165 | 0.000168 | 0 |
| Lpgat1 | 5.50E-09 | -0.16028 | 0.145 | 0.268 | 0.000171 | 0 |
| Ripor2 | 5.51E-09 | -0.17506 | 0.37 | 0.547 | 0.000171 | 0 |
| Psmb9 | 5.85E-09 | -0.15131 | 0.173 | 0.303 | 0.000182 | 0 |
| Zyg11b | 6.00E-09 | -0.1546 | 0.103 | 0.208 | 0.000186 | 0 |
| 4833420G17Rik | 6.17E-09 | -0.11121 | 0.106 | 0.214 | 0.000192 | 0 |
| Rps15 | 6.20E-09 | 0.108329 | 0.998 | 0.999 | 0.000193 | 0 |
| Myo1g | 6.32E-09 | -0.1393 | 0.116 | 0.227 | 0.000196 | 0 |
| Metap2 | 6.41E-09 | -0.12928 | 0.164 | 0.296 | 0.000199 | 0 |
| Ebna1bp2 | 6.86E-09 | -0.14269 | 0.089 | 0.187 | 0.000213 | 0 |
| Dapp1 | 6.86E-09 | -0.12393 | 0.154 | 0.282 | 0.000213 | 0 |
| Fryl | 7.34E-09 | -0.10887 | 0.097 | 0.2 | 0.000228 | 0 |
| Chd8 | 7.36E-09 | -0.14501 | 0.136 | 0.257 | 0.000228 | 0 |
| Cmip | 7.46E-09 | -0.14625 | 0.093 | 0.191 | 0.000232 | 0 |
| Gadd45gip1 | 7.77E-09 | -0.13002 | 0.111 | 0.221 | 0.000241 | 0 |
| Clasp1 | 7.90E-09 | -0.13991 | 0.107 | 0.212 | 0.000245 | 0 |
| Flii | 7.99E-09 | -0.12456 | 0.087 | 0.184 | 0.000248 | 0 |
| Ddx24 | 8.04E-09 | -0.1592 | 0.25 | 0.409 | 0.00025 | 0 |
| Nt5c | 8.04E-09 | -0.11574 | 0.151 | 0.278 | 0.00025 | 0 |
| Arhgap4 | 8.45E-09 | -0.14585 | 0.085 | 0.181 | 0.000262 | 0 |
| Man1a2 | 8.48E-09 | -0.15187 | 0.103 | 0.206 | 0.000263 | 0 |
| Prpf40a | 8.58E-09 | -0.17175 | 0.185 | 0.319 | 0.000266 | 0 |
| Tcf20 | 8.63E-09 | -0.10815 | 0.096 | 0.199 | 0.000268 | 0 |
| Hspa4 | 8.65E-09 | -0.15328 | 0.195 | 0.335 | 0.000268 | 0 |
| Susd6 | 9.04E-09 | -0.14034 | 0.141 | 0.261 | 0.000281 | 0 |
| Upf2 | 9.06E-09 | -0.14384 | 0.105 | 0.21 | 0.000281 | 0 |
| Itga4 | 9.21E-09 | -0.20043 | 0.396 | 0.57 | 0.000286 | 0 |
| Phf12 | 9.46E-09 | -0.11415 | 0.071 | 0.16 | 0.000294 | 0 |
| Cerk | 9.58E-09 | -0.15004 | 0.198 | 0.34 | 0.000297 | 0 |
| Pfdn6 | 9.79E-09 | -0.09919 | 0.119 | 0.233 | 0.000304 | 0 |
| Cyld | 1.00E-08 | -0.1261 | 0.18 | 0.316 | 0.000311 | 0 |
| Il17ra | 1.01E-08 | -0.12707 | 0.1 | 0.203 | 0.000313 | 0 |
| Slc7a1 | 1.01E-08 | -0.11819 | 0.067 | 0.153 | 0.000315 | 0 |
| Tmpo | 1.02E-08 | -0.15929 | 0.116 | 0.224 | 0.000317 | 0 |
| Dcaf1 | 1.06E-08 | -0.11435 | 0.039 | 0.109 | 0.000329 | 0 |
| Exoc3 | 1.08E-08 | -0.14145 | 0.07 | 0.156 | 0.000336 | 0 |
| Abhd17a | 1.09E-08 | -0.14294 | 0.107 | 0.21 | 0.000339 | 0 |
| Uvrag | 1.11E-08 | -0.13061 | 0.181 | 0.32 | 0.000344 | 0 |
| Ddx5 | 1.14E-08 | -0.14972 | 0.952 | 0.965 | 0.000354 | 0 |
| Lsm12 | 1.16E-08 | -0.15984 | 0.16 | 0.286 | 0.00036 | 0 |
| Tmf1 | 1.16E-08 | -0.1022 | 0.108 | 0.214 | 0.000361 | 0 |
| mt-Nd4l | 1.20E-08 | 0.31502 | 0.591 | 0.569 | 0.000373 | 0 |
| Chtop | 1.21E-08 | -0.10059 | 0.115 | 0.226 | 0.000377 | 0 |
| Prkcb | 1.26E-08 | -0.14033 | 0.275 | 0.443 | 0.00039 | 0 |
| Sec11c | 1.26E-08 | -0.20662 | 0.26 | 0.419 | 0.000391 | 0 |
| Zc3h13 | 1.27E-08 | -0.14013 | 0.123 | 0.237 | 0.000395 | 0 |
| Nolc1 | 1.34E-08 | -0.16157 | 0.097 | 0.196 | 0.000416 | 0 |
| Tcof1 | 1.34E-08 | -0.14421 | 0.142 | 0.263 | 0.000416 | 0 |
| Gga2 | 1.35E-08 | -0.13839 | 0.179 | 0.313 | 0.000419 | 0 |
| Kcnq1ot1 | 1.35E-08 | -0.30482 | 0.2 | 0.327 | 0.00042 | 0 |
| Stk17b | 1.37E-08 | 0.161376 | 0.959 | 0.953 | 0.000426 | 0 |
| Filip1l | 1.49E-08 | -0.18012 | 0.329 | 0.499 | 0.000461 | 0 |
| Spen | 1.56E-08 | -0.11929 | 0.069 | 0.155 | 0.000486 | 0 |
| Rsbn1l | 1.68E-08 | -0.14653 | 0.307 | 0.473 | 0.000521 | 0 |
| Lrrc58 | 1.72E-08 | -0.09842 | 0.041 | 0.111 | 0.000534 | 0 |
| Rnps1 | 1.72E-08 | -0.12138 | 0.121 | 0.233 | 0.000534 | 0 |
| B9d2 | 1.83E-08 | -0.11497 | 0.075 | 0.163 | 0.000568 | 0 |
| Dhx9 | 1.86E-08 | -0.11593 | 0.233 | 0.388 | 0.000578 | 0 |
| Safb | 1.91E-08 | -0.12946 | 0.15 | 0.27 | 0.000592 | 0 |
| Lsg1 | 1.91E-08 | -0.10879 | 0.068 | 0.154 | 0.000594 | 0 |
| Odc1 | 1.95E-08 | -0.14407 | 0.123 | 0.233 | 0.000606 | 0 |
| Tcea1 | 2.04E-08 | -0.10648 | 0.194 | 0.334 | 0.000634 | 0 |
| Park7 | 2.04E-08 | -0.14615 | 0.255 | 0.411 | 0.000634 | 0 |
| Ncor1 | 2.07E-08 | -0.14201 | 0.348 | 0.539 | 0.000644 | 0 |
| Pum3 | 2.10E-08 | -0.11518 | 0.047 | 0.119 | 0.000652 | 0 |
| Eif4b | 2.12E-08 | -0.14777 | 0.194 | 0.328 | 0.000657 | 0 |
| Atp2a2 | 2.20E-08 | -0.11951 | 0.101 | 0.202 | 0.000682 | 0 |
| Brpf1 | 2.21E-08 | -0.10107 | 0.05 | 0.124 | 0.000685 | 0 |
| Ncf4 | 2.21E-08 | -0.13998 | 0.22 | 0.365 | 0.000687 | 0 |
| Rest | 2.25E-08 | -0.1611 | 0.152 | 0.271 | 0.000698 | 0 |
| Edf1 | 2.32E-08 | -0.13697 | 0.251 | 0.415 | 0.000721 | 0 |
| Cd79a | 2.52E-08 | -0.14109 | 0.972 | 0.99 | 0.000781 | 0 |
| Rps14 | 2.65E-08 | 0.078003 | 1 | 0.999 | 0.000822 | 0 |
| Prps2 | 2.71E-08 | -0.10681 | 0.038 | 0.105 | 0.000841 | 0 |
| Arhgap30 | 2.85E-08 | -0.13963 | 0.328 | 0.5 | 0.000886 | 0 |
| Fam43a | 2.87E-08 | -0.12865 | 0.158 | 0.28 | 0.000893 | 0 |
| Lsp1 | 2.93E-08 | -0.18469 | 0.499 | 0.677 | 0.000909 | 0 |
| Prkce | 2.96E-08 | -0.14985 | 0.085 | 0.175 | 0.000918 | 0 |
| Plcg2 | 2.98E-08 | -0.12675 | 0.163 | 0.286 | 0.000926 | 0 |
| D10Wsu102e | 2.98E-08 | -0.17736 | 0.063 | 0.142 | 0.000927 | 0 |
| Grk6 | 2.99E-08 | -0.11624 | 0.227 | 0.384 | 0.000928 | 0 |
| Dynlrb1 | 2.99E-08 | -0.11445 | 0.167 | 0.292 | 0.000929 | 0 |
| Aqr | 2.99E-08 | -0.12416 | 0.113 | 0.218 | 0.000929 | 0 |
| Rcsd1 | 3.04E-08 | -0.1622 | 0.3 | 0.471 | 0.000943 | 0 |
| Mark3 | 3.08E-08 | -0.15473 | 0.122 | 0.228 | 0.000956 | 0 |
| Tab2 | 3.13E-08 | -0.08982 | 0.137 | 0.254 | 0.000972 | 0 |
| Cap1 | 3.23E-08 | -0.12694 | 0.136 | 0.251 | 0.001002 | 0 |
| Appbp2 | 3.30E-08 | -0.1201 | 0.088 | 0.18 | 0.001025 | 0 |
| Scfd1 | 3.34E-08 | -0.14142 | 0.088 | 0.179 | 0.001038 | 0 |
| Hnrnpu | 3.61E-08 | -0.15597 | 0.435 | 0.622 | 0.001121 | 0 |
| Rere | 3.70E-08 | -0.09635 | 0.113 | 0.22 | 0.00115 | 0 |
| Acin1 | 3.74E-08 | -0.14428 | 0.304 | 0.477 | 0.001162 | 0 |
| Eif3l | 3.96E-08 | -0.08962 | 0.105 | 0.206 | 0.001229 | 0 |
| Rexo1 | 3.96E-08 | -0.12101 | 0.104 | 0.204 | 0.001231 | 0 |
| Grpel1 | 3.99E-08 | -0.11909 | 0.086 | 0.178 | 0.001239 | 0 |
| Ccdc88c | 4.03E-08 | -0.10701 | 0.157 | 0.28 | 0.001251 | 0 |
| Rala | 4.06E-08 | -0.09806 | 0.115 | 0.222 | 0.00126 | 0 |
| Hnrnpd | 4.11E-08 | -0.15316 | 0.268 | 0.422 | 0.001278 | 0 |
| Cbx1 | 4.16E-08 | -0.15077 | 0.173 | 0.3 | 0.001292 | 0 |
| Ppp1r15b | 4.18E-08 | -0.09846 | 0.08 | 0.168 | 0.001297 | 0 |
| Trim11 | 4.28E-08 | -0.11867 | 0.085 | 0.175 | 0.001329 | 0 |
| Atm | 4.32E-08 | -0.12665 | 0.045 | 0.113 | 0.00134 | 0 |
| Zbtb20 | 4.41E-08 | -0.32904 | 0.237 | 0.365 | 0.001369 | 0 |
| Map2k2 | 4.42E-08 | -0.11925 | 0.165 | 0.29 | 0.001371 | 0 |
| Srpk1 | 4.49E-08 | -0.09279 | 0.122 | 0.232 | 0.001393 | 0 |
| Tmem230 | 4.60E-08 | -0.12585 | 0.043 | 0.11 | 0.001427 | 0 |
| Strbp | 4.71E-08 | -0.16937 | 0.185 | 0.313 | 0.001463 | 0 |
| Erlec1 | 4.72E-08 | -0.12217 | 0.056 | 0.131 | 0.001466 | 0 |
| Gimap7 | 4.82E-08 | -0.14363 | 0.06 | 0.136 | 0.001497 | 0 |
| Etv3 | 4.85E-08 | -0.12751 | 0.095 | 0.19 | 0.001507 | 0 |
| Zmynd8 | 4.87E-08 | -0.08754 | 0.132 | 0.245 | 0.001511 | 0 |
| Cd2 | 4.88E-08 | -0.13092 | 0.253 | 0.406 | 0.001516 | 0 |
| Zfp830 | 5.06E-08 | -0.12485 | 0.08 | 0.167 | 0.001571 | 0 |
| Magt1 | 5.10E-08 | -0.12158 | 0.102 | 0.199 | 0.001582 | 0 |
| Hsp90aa1 | 5.18E-08 | 0.27581 | 0.807 | 0.81 | 0.00161 | 0 |
| Rpl13 | 5.28E-08 | 0.084453 | 1 | 0.998 | 0.001638 | 0 |
| Secisbp2 | 5.39E-08 | -0.10529 | 0.049 | 0.119 | 0.001674 | 0 |
| Plk2 | 5.49E-08 | -0.23183 | 0.081 | 0.165 | 0.001706 | 0 |
| Wdr33 | 5.51E-08 | -0.11151 | 0.04 | 0.106 | 0.001712 | 0 |
| Ahnak | 5.57E-08 | -0.31937 | 0.154 | 0.259 | 0.001731 | 0 |
| Ice1 | 5.76E-08 | -0.10132 | 0.051 | 0.123 | 0.001788 | 0 |
| Dnajc8 | 5.88E-08 | -0.17391 | 0.277 | 0.426 | 0.001825 | 0 |
| Srpr | 5.88E-08 | -0.13281 | 0.123 | 0.226 | 0.001825 | 0 |
| Clec4d | 5.99E-08 | 0.285428 | 0.152 | 0.075 | 0.00186 | 0 |
| Eml4 | 6.10E-08 | -0.17271 | 0.259 | 0.403 | 0.001895 | 0 |
| Trim25 | 6.18E-08 | -0.20945 | 0.243 | 0.376 | 0.001919 | 0 |
| Xrn2 | 6.26E-08 | -0.14764 | 0.275 | 0.431 | 0.001944 | 0 |
| Sec24b | 6.46E-08 | -0.10266 | 0.055 | 0.13 | 0.002007 | 0 |
| Snx9 | 6.48E-08 | -0.12305 | 0.145 | 0.26 | 0.002013 | 0 |
| Eloa | 6.48E-08 | -0.1163 | 0.081 | 0.169 | 0.002014 | 0 |
| Trim33 | 6.56E-08 | -0.10654 | 0.095 | 0.19 | 0.002037 | 0 |
| Esco1 | 6.57E-08 | -0.14292 | 0.111 | 0.211 | 0.002041 | 0 |
| Rsbn1 | 6.59E-08 | -0.12809 | 0.137 | 0.248 | 0.002048 | 0 |
| Rgs19 | 6.61E-08 | -0.10793 | 0.095 | 0.191 | 0.002053 | 0 |
| Zfp831 | 6.65E-08 | -0.09953 | 0.041 | 0.107 | 0.002064 | 0 |
| Pfdn5 | 6.69E-08 | 0.182928 | 0.813 | 0.84 | 0.002076 | 0 |
| Chchd3 | 6.98E-08 | -0.10479 | 0.102 | 0.199 | 0.002167 | 0 |
| Ppp4r3b | 7.03E-08 | -0.10275 | 0.213 | 0.353 | 0.002184 | 0 |
| Lrmp | 7.13E-08 | -0.1181 | 0.113 | 0.216 | 0.002215 | 0 |
| Galnt11 | 7.14E-08 | -0.13963 | 0.13 | 0.237 | 0.002218 | 0 |
| Trim26 | 7.23E-08 | -0.13555 | 0.107 | 0.205 | 0.002245 | 0 |
| Dhx36 | 7.54E-08 | -0.13727 | 0.142 | 0.253 | 0.00234 | 0 |
| Nfya | 7.75E-08 | -0.12713 | 0.048 | 0.117 | 0.002407 | 0 |
| Eif2ak3 | 7.84E-08 | -0.14735 | 0.14 | 0.249 | 0.002435 | 0 |
| Gcc2 | 7.91E-08 | -0.13281 | 0.077 | 0.161 | 0.002456 | 0 |
| Rpf1 | 8.21E-08 | -0.07688 | 0.076 | 0.161 | 0.002548 | 0 |
| Fchsd2 | 8.29E-08 | -0.21402 | 0.343 | 0.499 | 0.002573 | 0 |
| Fam162a | 8.30E-08 | -0.10809 | 0.051 | 0.121 | 0.002577 | 0 |
| Ube2m | 8.42E-08 | -0.11726 | 0.121 | 0.226 | 0.002614 | 0 |
| Ctcf | 8.50E-08 | -0.15124 | 0.282 | 0.438 | 0.00264 | 0 |
| Rbpj | 8.54E-08 | -0.09452 | 0.055 | 0.129 | 0.002652 | 0 |
| Bod1l | 8.62E-08 | -0.17474 | 0.219 | 0.35 | 0.002676 | 0 |
| Sf3b2 | 8.69E-08 | -0.16037 | 0.343 | 0.517 | 0.002699 | 0 |
| Atg16l2 | 8.75E-08 | -0.10369 | 0.054 | 0.128 | 0.002716 | 0 |
| Yme1l1 | 8.81E-08 | -0.09591 | 0.142 | 0.259 | 0.002736 | 0 |
| Cops2 | 8.97E-08 | -0.11588 | 0.046 | 0.113 | 0.002784 | 0 |
| Ddx27 | 9.01E-08 | -0.11352 | 0.082 | 0.168 | 0.002799 | 0 |
| Rpl14 | 9.04E-08 | 0.114272 | 0.985 | 0.988 | 0.002808 | 0 |
| Ppp6r1 | 9.22E-08 | -0.07656 | 0.09 | 0.183 | 0.002863 | 0 |
| Sbno2 | 9.37E-08 | -0.12473 | 0.168 | 0.29 | 0.002911 | 0 |
| Asap1 | 9.40E-08 | -0.12061 | 0.09 | 0.179 | 0.002919 | 0 |
| Add3 | 9.57E-08 | -0.15924 | 0.3 | 0.457 | 0.002972 | 0 |
| 1810058I24Rik | 9.58E-08 | -0.13009 | 0.146 | 0.258 | 0.002976 | 0 |
| Nol8 | 9.77E-08 | -0.11741 | 0.081 | 0.167 | 0.003034 | 0 |
| Nono | 9.86E-08 | -0.09541 | 0.21 | 0.354 | 0.003062 | 0 |
| Snap29 | 1.01E-07 | -0.14782 | 0.101 | 0.193 | 0.003139 | 0 |
| Fkbp4 | 1.02E-07 | -0.12395 | 0.157 | 0.276 | 0.003158 | 0 |
| Hdgf | 1.02E-07 | -0.12061 | 0.185 | 0.316 | 0.003181 | 0 |
| Wbp11 | 1.02E-07 | -0.1019 | 0.17 | 0.294 | 0.003182 | 0 |
| Tcerg1 | 1.03E-07 | -0.11339 | 0.074 | 0.156 | 0.003186 | 0 |
| Cmtm7 | 1.03E-07 | -0.09579 | 0.232 | 0.378 | 0.0032 | 0 |
| Parp14 | 1.03E-07 | -0.15256 | 0.108 | 0.203 | 0.003202 | 0 |
| Mllt10 | 1.05E-07 | -0.13014 | 0.148 | 0.258 | 0.003251 | 0 |
| Eif2s1 | 1.06E-07 | -0.08666 | 0.117 | 0.222 | 0.003297 | 0 |
| Nup210 | 1.07E-07 | -0.10618 | 0.073 | 0.155 | 0.003317 | 0 |
| Hcfc1 | 1.07E-07 | -0.10198 | 0.065 | 0.142 | 0.003335 | 0 |
| Cd38 | 1.08E-07 | -0.12869 | 0.225 | 0.363 | 0.00335 | 0 |
| Clint1 | 1.09E-07 | -0.13232 | 0.176 | 0.301 | 0.003374 | 0 |
| Ubr5 | 1.09E-07 | -0.09516 | 0.099 | 0.195 | 0.003398 | 0 |
| Lars | 1.11E-07 | -0.1197 | 0.048 | 0.116 | 0.003437 | 0 |
| Prpf8 | 1.12E-07 | -0.11005 | 0.142 | 0.255 | 0.003483 | 0 |
| Senp6 | 1.12E-07 | -0.13005 | 0.19 | 0.321 | 0.003489 | 0 |
| Fkbp1a | 1.15E-07 | -0.11263 | 0.1 | 0.195 | 0.003566 | 0 |
| Eif3b | 1.15E-07 | -0.07448 | 0.113 | 0.216 | 0.003575 | 0 |
| Gar1 | 1.18E-07 | -0.08391 | 0.062 | 0.138 | 0.003664 | 0 |
| Stk10 | 1.19E-07 | -0.12466 | 0.123 | 0.227 | 0.003699 | 0 |
| Hbs1l | 1.24E-07 | -0.12762 | 0.045 | 0.111 | 0.003845 | 0 |
| Pecam1 | 1.26E-07 | -0.14874 | 0.195 | 0.32 | 0.003907 | 0 |
| Ptbp1 | 1.26E-07 | -0.1296 | 0.178 | 0.298 | 0.003912 | 0 |
| Rab8a | 1.26E-07 | -0.09875 | 0.168 | 0.294 | 0.003919 | 0 |
| Smg6 | 1.26E-07 | -0.11475 | 0.103 | 0.198 | 0.003925 | 0 |
| Rapgef6 | 1.30E-07 | -0.14749 | 0.298 | 0.455 | 0.004049 | 0 |
| Sec31a | 1.33E-07 | -0.1006 | 0.088 | 0.177 | 0.004144 | 0 |
| Akna | 1.34E-07 | -0.12257 | 0.144 | 0.253 | 0.004157 | 0 |
| Ifi209 | 1.34E-07 | -0.15972 | 0.24 | 0.377 | 0.004175 | 0 |
| Arid4b | 1.36E-07 | -0.16377 | 0.312 | 0.468 | 0.004225 | 0 |
| Nckap1l | 1.39E-07 | -0.0924 | 0.134 | 0.243 | 0.004311 | 0 |
| Nxf1 | 1.40E-07 | -0.10749 | 0.161 | 0.282 | 0.004345 | 0 |
| Bptf | 1.41E-07 | -0.15886 | 0.376 | 0.55 | 0.00437 | 0 |
| Sdad1 | 1.42E-07 | -0.11124 | 0.052 | 0.123 | 0.004408 | 0 |
| Psmc5 | 1.42E-07 | -0.11972 | 0.109 | 0.208 | 0.004421 | 0 |
| Sirt7 | 1.43E-07 | -0.09953 | 0.11 | 0.209 | 0.004439 | 0 |
| Grn | 1.44E-07 | 0.33247 | 0.181 | 0.104 | 0.004473 | 0 |
| Canx | 1.45E-07 | -0.11637 | 0.239 | 0.385 | 0.004503 | 0 |
| Cd79b | 1.48E-07 | -0.21993 | 0.723 | 0.858 | 0.004592 | 0 |
| Prorsd1 | 1.48E-07 | -0.12607 | 0.08 | 0.165 | 0.004595 | 0 |
| Psmd3 | 1.53E-07 | -0.08478 | 0.08 | 0.165 | 0.004761 | 0 |
| Snrnp70 | 1.54E-07 | -0.14001 | 0.37 | 0.557 | 0.004773 | 0 |
| Cdk11b | 1.54E-07 | -0.10683 | 0.225 | 0.37 | 0.004774 | 0 |
| Pkn1 | 1.55E-07 | -0.10148 | 0.201 | 0.334 | 0.004817 | 0 |
| Ehmt2 | 1.58E-07 | -0.08468 | 0.066 | 0.144 | 0.004898 | 0 |
| Herc2 | 1.58E-07 | -0.13978 | 0.068 | 0.146 | 0.004906 | 0 |
| Ctss | 1.63E-07 | 0.210129 | 0.742 | 0.739 | 0.005052 | 0 |
| Mgat1 | 1.63E-07 | -0.11237 | 0.08 | 0.163 | 0.005072 | 0 |
| Nol7 | 1.64E-07 | -0.15299 | 0.328 | 0.494 | 0.005096 | 0 |
| Galnt1 | 1.64E-07 | -0.09162 | 0.14 | 0.249 | 0.005101 | 0 |
| Jak2 | 1.68E-07 | -0.10852 | 0.159 | 0.277 | 0.005226 | 0 |
| Srsf5 | 1.70E-07 | -0.18157 | 0.624 | 0.784 | 0.005294 | 0 |
| Aff4 | 1.72E-07 | -0.16427 | 0.304 | 0.458 | 0.005339 | 0 |
| Bag1 | 1.76E-07 | -0.09678 | 0.169 | 0.292 | 0.005473 | 0 |
| Impdh2 | 1.76E-07 | -0.13158 | 0.157 | 0.272 | 0.005479 | 0 |
| Prkcsh | 1.80E-07 | -0.07705 | 0.054 | 0.125 | 0.005576 | 0 |
| Dusp5 | 1.83E-07 | -0.1226 | 0.106 | 0.199 | 0.005683 | 0 |
| Commd1 | 1.87E-07 | -0.10042 | 0.074 | 0.154 | 0.005814 | 0 |
| Kmt2c | 1.88E-07 | -0.13661 | 0.197 | 0.32 | 0.005831 | 0 |
| Card11 | 1.92E-07 | -0.12999 | 0.109 | 0.205 | 0.00596 | 0 |
| Smu1 | 1.94E-07 | -0.07336 | 0.089 | 0.179 | 0.006033 | 0 |
| Mlec | 1.94E-07 | -0.11058 | 0.077 | 0.159 | 0.006034 | 0 |
| Mien1 | 2.00E-07 | -0.0832 | 0.128 | 0.234 | 0.006202 | 0 |
| Tmem160 | 2.02E-07 | -0.0988 | 0.167 | 0.289 | 0.006269 | 0 |
| Hip1r | 2.06E-07 | -0.12103 | 0.148 | 0.258 | 0.006393 | 0 |
| Mat2a | 2.09E-07 | 0.345969 | 0.487 | 0.436 | 0.006487 | 0 |
| Vcp | 2.16E-07 | -0.13889 | 0.214 | 0.347 | 0.006719 | 0 |
| Cdk12 | 2.19E-07 | -0.11107 | 0.21 | 0.348 | 0.006799 | 0 |
| Sec61g | 2.19E-07 | 0.260528 | 0.604 | 0.579 | 0.006812 | 0 |
| Zfp646 | 2.21E-07 | -0.10866 | 0.088 | 0.174 | 0.006858 | 0 |
| Helz | 2.21E-07 | -0.12684 | 0.089 | 0.174 | 0.006876 | 0 |
| Cpm | 2.23E-07 | -0.16189 | 0.08 | 0.161 | 0.00692 | 0 |
| Gnl3 | 2.23E-07 | -0.1638 | 0.218 | 0.348 | 0.006934 | 0 |
| Dnajb11 | 2.24E-07 | -0.12711 | 0.109 | 0.204 | 0.006944 | 0 |
| Ppp2r5a | 2.26E-07 | -0.09619 | 0.195 | 0.327 | 0.007013 | 0 |
| Cd40 | 2.28E-07 | -0.07601 | 0.083 | 0.168 | 0.00708 | 0 |
| Rnaset2a | 2.30E-07 | -0.10294 | 0.127 | 0.23 | 0.007146 | 0 |
| Syncrip | 2.32E-07 | -0.11053 | 0.207 | 0.342 | 0.007207 | 0 |
| Msh2 | 2.35E-07 | -0.12528 | 0.045 | 0.109 | 0.007307 | 0 |
| Faf2 | 2.36E-07 | -0.08034 | 0.05 | 0.117 | 0.007314 | 0 |
| Eif3j1 | 2.36E-07 | -0.12007 | 0.172 | 0.294 | 0.007317 | 0 |
| Ddx17 | 2.38E-07 | -0.12262 | 0.222 | 0.359 | 0.007404 | 0 |
| Tgs1 | 2.41E-07 | -0.11427 | 0.057 | 0.128 | 0.007471 | 0 |
| Mrps17 | 2.42E-07 | -0.06479 | 0.065 | 0.141 | 0.007513 | 0 |
| Hnrnpdl | 2.45E-07 | -0.1581 | 0.332 | 0.49 | 0.007609 | 0 |
| Slc4a1ap | 2.49E-07 | -0.11021 | 0.067 | 0.143 | 0.007733 | 0 |
| Ufc1 | 2.50E-07 | -0.11528 | 0.107 | 0.198 | 0.007777 | 0 |
| Akap8 | 2.51E-07 | -0.12457 | 0.111 | 0.208 | 0.007789 | 0 |
| Akap8l | 2.55E-07 | -0.13285 | 0.104 | 0.196 | 0.007931 | 0 |
| Sh3pxd2a | 2.62E-07 | -0.13476 | 0.051 | 0.117 | 0.00815 | 0 |
| Satb1 | 2.67E-07 | 0.227193 | 0.687 | 0.612 | 0.008277 | 0 |
| Madd | 2.70E-07 | -0.12036 | 0.079 | 0.16 | 0.008391 | 0 |
| Ppp1r14b | 2.76E-07 | -0.14104 | 0.124 | 0.223 | 0.008557 | 0 |
| Baz1a | 2.78E-07 | -0.1298 | 0.297 | 0.455 | 0.008625 | 0 |
| Cnn2 | 2.83E-07 | -0.12368 | 0.236 | 0.381 | 0.008797 | 0 |
| Zbtb8os | 2.87E-07 | -0.09369 | 0.063 | 0.136 | 0.008912 | 0 |
| Cdk13 | 2.91E-07 | -0.08401 | 0.167 | 0.285 | 0.009033 | 0 |
| Dip2b | 2.91E-07 | -0.11452 | 0.122 | 0.221 | 0.00904 | 0 |
| Rb1 | 2.95E-07 | -0.10467 | 0.081 | 0.163 | 0.009156 | 0 |
| Rpl19 | 2.95E-07 | 0.07578 | 1 | 0.999 | 0.009164 | 0 |
| Ralgapa1 | 3.06E-07 | -0.08316 | 0.073 | 0.153 | 0.009508 | 0 |
| Nsd3 | 3.08E-07 | -0.09611 | 0.269 | 0.432 | 0.009561 | 0 |
| Utp23 | 3.15E-07 | -0.09019 | 0.041 | 0.103 | 0.009768 | 0 |
| Fam208b | 3.19E-07 | -0.11823 | 0.11 | 0.205 | 0.009919 | 0 |
| Rgs14 | 3.20E-07 | -0.11249 | 0.047 | 0.111 | 0.009924 | 0 |
| Anapc5 | 3.21E-07 | -0.07235 | 0.149 | 0.261 | 0.009982 | 0 |
| Usp10 | 3.24E-07 | -0.09564 | 0.064 | 0.137 | 0.010058 | 0 |
| Ncoa3 | 3.30E-07 | -0.09761 | 0.134 | 0.24 | 0.010249 | 0 |
| Dmac2 | 3.33E-07 | -0.13987 | 0.082 | 0.163 | 0.010348 | 0 |
| Cfdp1 | 3.36E-07 | -0.08122 | 0.107 | 0.2 | 0.010423 | 0 |
| Pa2g4 | 3.40E-07 | -0.12486 | 0.125 | 0.226 | 0.010557 | 0 |
| Creb1 | 3.46E-07 | -0.13183 | 0.138 | 0.243 | 0.010729 | 0 |
| Rnf20 | 3.46E-07 | -0.10664 | 0.099 | 0.19 | 0.010759 | 0 |
| Carhsp1 | 3.64E-07 | -0.08356 | 0.044 | 0.107 | 0.011305 | 0 |
| Eif4g3 | 3.66E-07 | -0.12119 | 0.154 | 0.267 | 0.01138 | 0 |
| Flna | 3.67E-07 | -0.15275 | 0.192 | 0.314 | 0.011393 | 0 |
| Asxl1 | 3.76E-07 | -0.09248 | 0.043 | 0.105 | 0.011676 | 0 |
| Senp7 | 3.86E-07 | -0.11299 | 0.119 | 0.214 | 0.011974 | 0 |
| Nsrp1 | 3.88E-07 | -0.16893 | 0.096 | 0.18 | 0.012048 | 0 |
| 4930523C07Rik | 3.89E-07 | -0.12899 | 0.457 | 0.641 | 0.012092 | 0 |
| Spib | 3.90E-07 | -0.16457 | 0.196 | 0.315 | 0.012107 | 0 |
| Dnttip2 | 3.90E-07 | -0.12012 | 0.139 | 0.245 | 0.012116 | 0 |
| Lypla2 | 3.97E-07 | -0.09273 | 0.066 | 0.141 | 0.012318 | 0 |
| Irf8 | 3.99E-07 | -0.16656 | 0.314 | 0.461 | 0.012383 | 0 |
| Aggf1 | 4.06E-07 | -0.09194 | 0.061 | 0.132 | 0.012611 | 0 |
| Sdhb | 4.10E-07 | -0.07416 | 0.118 | 0.218 | 0.012741 | 0 |
| Rif1 | 4.22E-07 | -0.12086 | 0.078 | 0.156 | 0.013103 | 0 |
| Polg | 4.34E-07 | -0.08722 | 0.046 | 0.11 | 0.013473 | 0 |
| Cltc | 4.47E-07 | -0.09012 | 0.213 | 0.347 | 0.013866 | 0 |
| Ctsb | 4.47E-07 | 0.361304 | 0.349 | 0.285 | 0.013891 | 0 |
| Smc4 | 4.50E-07 | -0.15798 | 0.141 | 0.241 | 0.013969 | 0 |
| Mrps24 | 4.51E-07 | -0.09549 | 0.196 | 0.326 | 0.014008 | 0 |
| Copb2 | 4.52E-07 | -0.0863 | 0.079 | 0.159 | 0.01403 | 0 |
| Ddx10 | 4.56E-07 | -0.07887 | 0.072 | 0.15 | 0.014146 | 0 |
| Arhgef6 | 4.57E-07 | -0.10105 | 0.094 | 0.181 | 0.014182 | 0 |
| Nek7 | 4.59E-07 | -0.08567 | 0.142 | 0.253 | 0.014241 | 0 |
| Chd9 | 4.66E-07 | -0.14604 | 0.125 | 0.221 | 0.014477 | 0 |
| Larp4 | 4.69E-07 | -0.08477 | 0.157 | 0.272 | 0.014578 | 0 |
| Rer1 | 4.72E-07 | -0.08099 | 0.155 | 0.268 | 0.014645 | 0 |
| Phf20 | 4.80E-07 | -0.09971 | 0.065 | 0.138 | 0.0149 | 0 |
| Szrd1 | 4.90E-07 | -0.07616 | 0.094 | 0.181 | 0.015209 | 0 |
| Ptp4a3 | 4.98E-07 | -0.17772 | 0.282 | 0.42 | 0.015464 | 0 |
| Cux1 | 4.98E-07 | -0.10817 | 0.161 | 0.272 | 0.015476 | 0 |
| AC125149.3 | 5.07E-07 | -0.07464 | 0.084 | 0.168 | 0.015739 | 0 |
| Rwdd1 | 5.28E-07 | -0.10765 | 0.149 | 0.258 | 0.016395 | 0 |
| Tnfaip8 | 5.28E-07 | -0.12632 | 0.18 | 0.297 | 0.016405 | 0 |
| Ube2h | 5.31E-07 | -0.13246 | 0.128 | 0.226 | 0.016481 | 0 |
| Set | 5.46E-07 | -0.16582 | 0.5 | 0.673 | 0.016968 | 0 |
| Uhrf1bp1l | 5.51E-07 | -0.10444 | 0.068 | 0.142 | 0.017101 | 0 |
| Fgfr1op2 | 5.60E-07 | -0.08194 | 0.22 | 0.36 | 0.017393 | 0 |
| Ube2r2 | 5.63E-07 | -0.10649 | 0.134 | 0.235 | 0.017497 | 0 |
| Ccdc47 | 5.65E-07 | -0.09106 | 0.089 | 0.172 | 0.017546 | 0 |
| Itgb1 | 5.71E-07 | -0.11697 | 0.08 | 0.159 | 0.017725 | 0 |
| Suclg2 | 5.72E-07 | -0.07663 | 0.041 | 0.101 | 0.017767 | 0 |
| Siva1 | 5.72E-07 | -0.10614 | 0.054 | 0.121 | 0.017767 | 0 |
| Senp1 | 5.76E-07 | -0.09768 | 0.051 | 0.116 | 0.017875 | 0 |
| Rpp25l | 5.79E-07 | -0.08975 | 0.042 | 0.103 | 0.017981 | 0 |
| Pkn2 | 5.82E-07 | -0.10333 | 0.178 | 0.3 | 0.018085 | 0 |
| Taf15 | 5.83E-07 | -0.13237 | 0.165 | 0.276 | 0.018093 | 0 |
| Nfkb2 | 5.86E-07 | -0.10667 | 0.11 | 0.204 | 0.018194 | 0 |
| Cbx5 | 6.02E-07 | -0.10994 | 0.058 | 0.126 | 0.01869 | 0 |
| Axin1 | 6.13E-07 | -0.07753 | 0.051 | 0.118 | 0.019034 | 0 |
| Sla | 6.15E-07 | -0.1198 | 0.203 | 0.326 | 0.019093 | 0 |
| Emg1 | 6.22E-07 | -0.0502 | 0.204 | 0.338 | 0.019307 | 0 |
| Ncoa2 | 6.28E-07 | -0.10197 | 0.093 | 0.178 | 0.019493 | 0 |
| Yy1 | 6.31E-07 | -0.09351 | 0.262 | 0.412 | 0.019599 | 0 |
| Cyc1 | 6.33E-07 | -0.089 | 0.138 | 0.245 | 0.019657 | 0 |
| Arcn1 | 6.34E-07 | -0.09141 | 0.157 | 0.271 | 0.019691 | 0 |
| Fam114a2 | 6.34E-07 | -0.10189 | 0.053 | 0.119 | 0.019697 | 0 |
| Il21r | 6.35E-07 | -0.151 | 0.121 | 0.212 | 0.019717 | 0 |
| Mettl23 | 6.53E-07 | -0.10253 | 0.202 | 0.328 | 0.020276 | 0 |
| Hmox2 | 6.55E-07 | -0.0686 | 0.06 | 0.131 | 0.020331 | 0 |
| Gars | 6.66E-07 | -0.06945 | 0.05 | 0.115 | 0.020667 | 0 |
| Btk | 6.66E-07 | -0.08667 | 0.187 | 0.31 | 0.020668 | 0 |
| Tmem71 | 6.69E-07 | -0.10894 | 0.051 | 0.115 | 0.020764 | 0 |
| Aplp2 | 6.89E-07 | -0.13398 | 0.116 | 0.209 | 0.021396 | 0 |
| Uqcrc1 | 6.95E-07 | -0.0861 | 0.132 | 0.234 | 0.021573 | 0 |
| Sept11 | 7.07E-07 | -0.12662 | 0.116 | 0.208 | 0.021967 | 0 |
| Plekha2 | 7.21E-07 | -0.11217 | 0.225 | 0.357 | 0.022376 | 0 |
| Ola1 | 7.22E-07 | -0.1052 | 0.1 | 0.186 | 0.022417 | 0 |
| Cul3 | 7.22E-07 | -0.08121 | 0.181 | 0.302 | 0.022425 | 0 |
| AA467197 | 7.38E-07 | 0.308642 | 0.145 | 0.075 | 0.022906 | 0 |
| Prpf38b | 7.61E-07 | -0.1169 | 0.355 | 0.523 | 0.023632 | 0 |
| Bmp2k | 7.64E-07 | -0.18194 | 0.204 | 0.319 | 0.023731 | 0 |
| Tmed9 | 7.70E-07 | -0.11723 | 0.254 | 0.394 | 0.023906 | 0 |
| Banf1 | 7.78E-07 | -0.11963 | 0.171 | 0.286 | 0.024172 | 0 |
| Pmm2 | 7.88E-07 | -0.09541 | 0.044 | 0.104 | 0.024468 | 0 |
| Topors | 7.96E-07 | -0.10981 | 0.1 | 0.186 | 0.024724 | 0 |
| Rpn1 | 8.17E-07 | -0.09842 | 0.082 | 0.162 | 0.025366 | 0 |
| Ly9 | 8.21E-07 | -0.08776 | 0.094 | 0.179 | 0.025504 | 0 |
| Snx30 | 8.40E-07 | -0.14148 | 0.123 | 0.218 | 0.02608 | 0 |
| Zcchc11 | 8.52E-07 | -0.17448 | 0.466 | 0.629 | 0.026467 | 0 |
| Tmem131 | 8.72E-07 | -0.07381 | 0.067 | 0.141 | 0.027088 | 0 |
| St6gal1 | 8.88E-07 | -0.11679 | 0.11 | 0.2 | 0.027578 | 0 |
| Ttc7 | 9.10E-07 | -0.10223 | 0.09 | 0.172 | 0.028254 | 0 |
| Eif4g2 | 9.14E-07 | -0.12949 | 0.485 | 0.662 | 0.028393 | 0 |
| Gdi1 | 9.16E-07 | -0.06867 | 0.067 | 0.141 | 0.028446 | 0 |
| Ppfia1 | 9.23E-07 | -0.09025 | 0.065 | 0.136 | 0.028674 | 0 |
| Nucks1 | 9.52E-07 | -0.10565 | 0.281 | 0.44 | 0.029566 | 0 |
| Mapre2 | 9.58E-07 | -0.05965 | 0.068 | 0.142 | 0.029761 | 0 |
| Phf11b | 9.63E-07 | -0.07015 | 0.052 | 0.117 | 0.029906 | 0 |
| Polr2j | 9.65E-07 | -0.10099 | 0.125 | 0.222 | 0.029965 | 0 |
| Hivep1 | 9.66E-07 | -0.07773 | 0.078 | 0.155 | 0.030001 | 0 |
| Bend5 | 9.66E-07 | -0.12001 | 0.062 | 0.13 | 0.030008 | 0 |
| Larp1 | 9.69E-07 | -0.05317 | 0.134 | 0.237 | 0.030084 | 0 |
| Taf7 | 9.70E-07 | -0.08978 | 0.11 | 0.203 | 0.030108 | 0 |
| Vgll4 | 9.70E-07 | -0.12968 | 0.152 | 0.255 | 0.030115 | 0 |
| Aak1 | 9.74E-07 | -0.11224 | 0.055 | 0.121 | 0.03024 | 0 |
| Zmynd11 | 9.92E-07 | -0.12395 | 0.211 | 0.34 | 0.030802 | 0 |
| Rps28 | 1.00E-06 | 0.114485 | 0.993 | 0.989 | 0.031132 | 0 |
| Fos | 1.00E-06 | 0.211998 | 0.623 | 0.568 | 0.031144 | 0 |
| Grap | 1.01E-06 | -0.09064 | 0.24 | 0.379 | 0.031363 | 0 |
| Ino80 | 1.03E-06 | -0.07806 | 0.063 | 0.132 | 0.031927 | 0 |
| Cox4i1 | 1.03E-06 | 0.17487 | 0.818 | 0.838 | 0.032017 | 0 |
| Pkd1 | 1.04E-06 | -0.08381 | 0.051 | 0.116 | 0.032209 | 0 |
| Ifi203 | 1.05E-06 | -0.18023 | 0.408 | 0.566 | 0.032505 | 0 |
| Bclaf1 | 1.05E-06 | -0.09451 | 0.368 | 0.545 | 0.032539 | 0 |
| Rbl2 | 1.05E-06 | -0.08264 | 0.07 | 0.144 | 0.032606 | 0 |
| Ddx42 | 1.06E-06 | -0.14495 | 0.095 | 0.178 | 0.032878 | 0 |
| Uimc1 | 1.10E-06 | -0.07869 | 0.088 | 0.171 | 0.034219 | 0 |
| Was | 1.11E-06 | -0.10837 | 0.141 | 0.245 | 0.03456 | 0 |
| Lrrc59 | 1.13E-06 | -0.08486 | 0.049 | 0.111 | 0.034971 | 0 |
| Gon4l | 1.13E-06 | -0.09493 | 0.075 | 0.15 | 0.035202 | 0 |
| Tapbp | 1.14E-06 | -0.07228 | 0.237 | 0.384 | 0.035428 | 0 |
| Pip4k2a | 1.14E-06 | -0.0911 | 0.092 | 0.175 | 0.035472 | 0 |
| Zpr1 | 1.15E-06 | -0.09231 | 0.053 | 0.118 | 0.03563 | 0 |
| Ppp1cc | 1.15E-06 | -0.07019 | 0.222 | 0.359 | 0.03586 | 0 |
| Rps6kb1 | 1.16E-06 | -0.10436 | 0.163 | 0.273 | 0.035985 | 0 |
| Ywhaq | 1.16E-06 | -0.14258 | 0.26 | 0.4 | 0.036146 | 0 |
| Snd1 | 1.19E-06 | -0.07463 | 0.046 | 0.106 | 0.036834 | 0 |
| Brd8 | 1.19E-06 | -0.08494 | 0.156 | 0.267 | 0.037047 | 0 |
| Phip | 1.21E-06 | -0.13997 | 0.33 | 0.479 | 0.037528 | 0 |
| Ptpn2 | 1.23E-06 | -0.08658 | 0.092 | 0.174 | 0.038272 | 0 |
| Zfp507 | 1.24E-06 | -0.11518 | 0.043 | 0.101 | 0.038435 | 0 |
| Rabep1 | 1.24E-06 | -0.07854 | 0.079 | 0.156 | 0.038574 | 0 |
| Tbcb | 1.24E-06 | -0.08742 | 0.101 | 0.189 | 0.038642 | 0 |
| Herpud2 | 1.25E-06 | -0.12271 | 0.076 | 0.15 | 0.038728 | 0 |
| Trrap | 1.26E-06 | -0.08051 | 0.088 | 0.169 | 0.039012 | 0 |
| Ndufs6 | 1.26E-06 | -0.05381 | 0.156 | 0.271 | 0.039049 | 0 |
| Nck1 | 1.27E-06 | -0.11737 | 0.074 | 0.147 | 0.03951 | 0 |
| Sltm | 1.27E-06 | -0.11414 | 0.289 | 0.436 | 0.039518 | 0 |
| Srp72 | 1.29E-06 | -0.06892 | 0.154 | 0.265 | 0.040055 | 0 |
| Copb1 | 1.29E-06 | -0.08612 | 0.149 | 0.254 | 0.040155 | 0 |
| Trak2 | 1.30E-06 | -0.10125 | 0.064 | 0.132 | 0.040227 | 0 |
| Epb41l2 | 1.30E-06 | -0.08944 | 0.11 | 0.2 | 0.040291 | 0 |
| Atxn7l3b | 1.30E-06 | -0.09537 | 0.178 | 0.292 | 0.040309 | 0 |
| Acap2 | 1.30E-06 | -0.11998 | 0.089 | 0.168 | 0.040388 | 0 |
| Tmem147 | 1.33E-06 | -0.09387 | 0.058 | 0.124 | 0.041303 | 0 |
| Gripap1 | 1.33E-06 | -0.10064 | 0.138 | 0.237 | 0.041438 | 0 |
| Clic4 | 1.34E-06 | -0.10123 | 0.099 | 0.184 | 0.041691 | 0 |
| Mrpl43 | 1.36E-06 | -0.04865 | 0.111 | 0.205 | 0.042193 | 0 |
| Setd5 | 1.41E-06 | -0.07778 | 0.155 | 0.265 | 0.043859 | 0 |
| Mrpl11 | 1.42E-06 | -0.06475 | 0.075 | 0.15 | 0.04404 | 0 |
| Ibtk | 1.43E-06 | -0.10304 | 0.075 | 0.149 | 0.044335 | 0 |
| Tap1 | 1.43E-06 | -0.06676 | 0.173 | 0.29 | 0.044549 | 0 |
| Rab11fip2 | 1.44E-06 | -0.10573 | 0.061 | 0.128 | 0.044626 | 0 |
| Atp5l | 1.44E-06 | 0.201688 | 0.684 | 0.716 | 0.044853 | 0 |
| Gimap8 | 1.45E-06 | -0.10054 | 0.143 | 0.246 | 0.044872 | 0 |
| Sipa1 | 1.45E-06 | -0.10787 | 0.236 | 0.364 | 0.045026 | 0 |
| Ppp1r12a | 1.46E-06 | -0.06016 | 0.216 | 0.353 | 0.045248 | 0 |
| Mysm1 | 1.46E-06 | -0.09513 | 0.138 | 0.239 | 0.045344 | 0 |
| Clcn3 | 1.48E-06 | -0.10647 | 0.119 | 0.21 | 0.046012 | 0 |
| Pkig | 1.49E-06 | -0.13961 | 0.413 | 0.588 | 0.046176 | 0 |
| Trpc4ap | 1.49E-06 | -0.09009 | 0.135 | 0.233 | 0.046229 | 0 |
| Rpl10a | 1.49E-06 | 0.103964 | 0.992 | 0.993 | 0.046392 | 0 |
| Sh3bp5 | 1.51E-06 | -0.11319 | 0.224 | 0.352 | 0.046742 | 0 |
| Dctn1 | 1.51E-06 | -0.05829 | 0.047 | 0.109 | 0.046817 | 0 |
| Epc2 | 1.52E-06 | -0.12112 | 0.124 | 0.217 | 0.047176 | 0 |
| Tec | 1.52E-06 | -0.12563 | 0.129 | 0.222 | 0.047186 | 0 |
| Psmb8 | 1.54E-06 | -0.13451 | 0.408 | 0.578 | 0.047761 | 0 |
| Srgn | 1.55E-06 | 0.179654 | 0.917 | 0.93 | 0.048056 | 0 |
| Ybx3 | 1.55E-06 | -0.069 | 0.084 | 0.163 | 0.048068 | 0 |
| Crebbp | 1.56E-06 | -0.10645 | 0.262 | 0.397 | 0.048561 | 0 |
| Col4a3bp | 1.58E-06 | -0.08205 | 0.065 | 0.135 | 0.048932 | 0 |
| Cnot3 | 1.59E-06 | -0.07542 | 0.125 | 0.222 | 0.049435 | 0 |
| Xbp1 | 1.61E-06 | -0.21233 | 0.087 | 0.163 | 0.050097 | 0 |
| Zcchc6 | 1.62E-06 | -0.12007 | 0.309 | 0.457 | 0.050342 | 0 |
| Snrnp40 | 1.64E-06 | -0.08079 | 0.089 | 0.169 | 0.050957 | 0 |
| Pi4ka | 1.67E-06 | -0.08406 | 0.061 | 0.128 | 0.051718 | 0 |
| Il16 | 1.69E-06 | -0.10412 | 0.111 | 0.2 | 0.052581 | 0 |
| Hcls1 | 1.70E-06 | -0.10798 | 0.259 | 0.4 | 0.052733 | 0 |
| Cxcr5 | 1.70E-06 | -0.13892 | 0.231 | 0.357 | 0.052922 | 0 |
| Vps4b | 1.72E-06 | -0.07222 | 0.122 | 0.217 | 0.05335 | 0 |
| Sipa1l1 | 1.72E-06 | -0.08718 | 0.125 | 0.221 | 0.053517 | 0 |
| Man2b1 | 1.75E-06 | -0.09373 | 0.187 | 0.304 | 0.054405 | 0 |
| Dut | 1.76E-06 | -0.09817 | 0.073 | 0.144 | 0.054664 | 0 |
| Rps12-ps3 | 1.77E-06 | -0.07112 | 0.09 | 0.171 | 0.054892 | 0 |
| Faf1 | 1.77E-06 | -0.08994 | 0.049 | 0.11 | 0.054941 | 0 |
| Bcl7c | 1.77E-06 | -0.05898 | 0.136 | 0.237 | 0.055074 | 0 |
| Pes1 | 1.82E-06 | -0.06022 | 0.051 | 0.113 | 0.05656 | 0 |
| Rfx7 | 1.82E-06 | -0.09432 | 0.152 | 0.258 | 0.056623 | 0 |
| Prpf6 | 1.84E-06 | -0.12141 | 0.064 | 0.13 | 0.057028 | 0 |
| Nrip1 | 1.85E-06 | -0.10111 | 0.082 | 0.158 | 0.057578 | 0 |
| Ssrp1 | 1.85E-06 | -0.09287 | 0.126 | 0.221 | 0.057598 | 0 |
| Arid5b | 1.88E-06 | -0.10236 | 0.17 | 0.279 | 0.058336 | 0 |
| Pten | 1.88E-06 | -0.09631 | 0.323 | 0.482 | 0.058504 | 0 |
| Naa15 | 1.89E-06 | -0.07539 | 0.145 | 0.247 | 0.058651 | 0 |
| E2f4 | 1.92E-06 | -0.06177 | 0.053 | 0.117 | 0.059605 | 0 |
| Uqcrc2 | 1.94E-06 | -0.07111 | 0.161 | 0.274 | 0.06029 | 0 |
| Fcrl1 | 1.94E-06 | -0.14222 | 0.205 | 0.322 | 0.060345 | 0 |
| Got2 | 1.98E-06 | -0.06583 | 0.08 | 0.158 | 0.061621 | 0 |
| Clk3 | 1.99E-06 | -0.08083 | 0.082 | 0.159 | 0.061677 | 0 |
| Ccng1 | 2.00E-06 | -0.05229 | 0.052 | 0.116 | 0.062021 | 0 |
| Txlna | 2.00E-06 | -0.04803 | 0.077 | 0.153 | 0.062205 | 0 |
| Psmb5 | 2.01E-06 | -0.05846 | 0.212 | 0.347 | 0.062299 | 0 |
| Elf4 | 2.02E-06 | -0.09485 | 0.056 | 0.121 | 0.062706 | 0 |
| Nmt1 | 2.02E-06 | -0.08237 | 0.087 | 0.166 | 0.062777 | 0 |
| Bnip2 | 2.03E-06 | -0.06132 | 0.107 | 0.195 | 0.063133 | 0 |
| Ciita | 2.04E-06 | -0.10573 | 0.215 | 0.335 | 0.063398 | 0 |
| Suclg1 | 2.08E-06 | -0.09236 | 0.139 | 0.239 | 0.064453 | 0 |
| Arid1b | 2.09E-06 | -0.0515 | 0.131 | 0.232 | 0.064843 | 0 |
| Tfam | 2.10E-06 | -0.13677 | 0.182 | 0.292 | 0.065199 | 0 |
| Pdcd6ip | 2.11E-06 | -0.07944 | 0.144 | 0.248 | 0.065549 | 0 |
| Helz2 | 2.12E-06 | -0.1023 | 0.146 | 0.247 | 0.065713 | 0 |
| Ctsl | 2.14E-06 | 0.296888 | 0.167 | 0.099 | 0.066479 | 0 |
| Snrnp200 | 2.15E-06 | -0.09949 | 0.101 | 0.184 | 0.06687 | 0 |
| Clcn4 | 2.15E-06 | -0.05837 | 0.061 | 0.128 | 0.066882 | 0 |
| Selenot | 2.17E-06 | -0.08884 | 0.156 | 0.26 | 0.067407 | 0 |
| Pmf1 | 2.17E-06 | -0.08284 | 0.128 | 0.226 | 0.067505 | 0 |
| Ndufa13 | 2.18E-06 | -0.10829 | 0.355 | 0.523 | 0.067615 | 0 |
| Tnrc6b | 2.20E-06 | -0.12706 | 0.334 | 0.482 | 0.068165 | 0 |
| Rassf5 | 2.22E-06 | -0.08324 | 0.087 | 0.165 | 0.068865 | 0 |
| Zranb2 | 2.26E-06 | -0.0751 | 0.105 | 0.191 | 0.070071 | 0 |
| Cct3 | 2.29E-06 | -0.08848 | 0.206 | 0.331 | 0.071247 | 0 |
| Aamp | 2.30E-06 | -0.06172 | 0.14 | 0.242 | 0.07154 | 0 |
| Itpr3 | 2.32E-06 | -0.08896 | 0.121 | 0.214 | 0.071892 | 0 |
| Senp2 | 2.33E-06 | -0.06417 | 0.161 | 0.272 | 0.072476 | 0 |
| Ccz1 | 2.34E-06 | -0.0869 | 0.101 | 0.185 | 0.072567 | 0 |
| Ppwd1 | 2.36E-06 | -0.09818 | 0.083 | 0.158 | 0.0734 | 0 |
| Ezr | 2.54E-06 | -0.12702 | 0.435 | 0.598 | 0.078843 | 0 |
| Mrps26 | 2.54E-06 | -0.09542 | 0.096 | 0.177 | 0.078956 | 0 |
| Cyth4 | 2.55E-06 | -0.06795 | 0.089 | 0.168 | 0.079212 | 0 |
| Zfp36 | 2.55E-06 | 0.269478 | 0.628 | 0.626 | 0.079251 | 0 |
| Lat2 | 2.63E-06 | -0.09784 | 0.156 | 0.26 | 0.081604 | 0 |
| Colgalt1 | 2.65E-06 | -0.09297 | 0.107 | 0.191 | 0.082382 | 0 |
| Utp3 | 2.65E-06 | -0.10525 | 0.124 | 0.215 | 0.082437 | 0 |
| Guk1 | 2.70E-06 | -0.06922 | 0.07 | 0.141 | 0.083777 | 0 |
| Larp7 | 2.74E-06 | -0.10049 | 0.075 | 0.147 | 0.085124 | 0 |
| Nudc | 2.76E-06 | -0.10294 | 0.171 | 0.282 | 0.085583 | 0 |
| Caprin1 | 2.76E-06 | -0.07641 | 0.19 | 0.314 | 0.085761 | 0 |
| Ino80e | 2.79E-06 | -0.0633 | 0.054 | 0.117 | 0.08662 | 0 |
| Tnks2 | 2.83E-06 | -0.096 | 0.152 | 0.254 | 0.088004 | 0 |
| Smarce1 | 2.87E-06 | -0.07299 | 0.154 | 0.26 | 0.088995 | 0 |
| Kpna4 | 2.91E-06 | -0.06973 | 0.187 | 0.308 | 0.090402 | 0 |
| Spag7 | 2.92E-06 | -0.04922 | 0.09 | 0.171 | 0.090622 | 0 |
| Cep170 | 2.94E-06 | -0.11678 | 0.073 | 0.142 | 0.091373 | 0 |
| Dek | 2.98E-06 | -0.14378 | 0.326 | 0.468 | 0.092403 | 0 |
| Cep57 | 2.98E-06 | -0.10676 | 0.097 | 0.177 | 0.092404 | 0 |
| Cggbp1 | 2.98E-06 | -0.10601 | 0.266 | 0.402 | 0.092523 | 0 |
| Ppa1 | 2.99E-06 | -0.09218 | 0.052 | 0.113 | 0.092706 | 0 |
| Copg1 | 3.03E-06 | -0.03422 | 0.058 | 0.124 | 0.094075 | 0 |
| Stag2 | 3.04E-06 | -0.10242 | 0.189 | 0.302 | 0.094498 | 0 |
| Eif4h | 3.06E-06 | -0.09453 | 0.203 | 0.323 | 0.095018 | 0 |
| Clptm1l | 3.07E-06 | -0.10234 | 0.101 | 0.184 | 0.095398 | 0 |
| Mapkap1 | 3.09E-06 | -0.06035 | 0.069 | 0.14 | 0.095804 | 0 |
| Znrf2 | 3.09E-06 | -0.09409 | 0.134 | 0.229 | 0.095854 | 0 |
| Zfc3h1 | 3.10E-06 | -0.09657 | 0.068 | 0.136 | 0.096281 | 0 |
| Setd2 | 3.12E-06 | -0.11897 | 0.219 | 0.347 | 0.096747 | 0 |
| 2810004N23Rik | 3.16E-06 | -0.10619 | 0.062 | 0.126 | 0.098063 | 0 |
| Lrrfip1 | 3.17E-06 | -0.06412 | 0.167 | 0.277 | 0.098378 | 0 |
| Ftsj3 | 3.17E-06 | -0.07167 | 0.054 | 0.117 | 0.0984 | 0 |
| Zc3h18 | 3.18E-06 | -0.07161 | 0.082 | 0.159 | 0.098697 | 0 |
| Uchl3 | 3.19E-06 | -0.09272 | 0.166 | 0.272 | 0.099015 | 0 |
| Eps15 | 3.21E-06 | -0.08131 | 0.102 | 0.184 | 0.099697 | 0 |
| Puf60 | 3.22E-06 | -0.06173 | 0.19 | 0.311 | 0.100098 | 0 |
| Usp15 | 3.28E-06 | -0.09422 | 0.19 | 0.308 | 0.101927 | 0 |
| Vpreb3 | 3.29E-06 | -0.20453 | 0.347 | 0.47 | 0.102289 | 0 |
| Brd4 | 3.32E-06 | -0.04889 | 0.234 | 0.372 | 0.103196 | 0 |
| Hypk | 3.35E-06 | -0.07661 | 0.108 | 0.193 | 0.103922 | 0 |
| Auh | 3.43E-06 | -0.08344 | 0.053 | 0.115 | 0.106645 | 0 |
| Zfp131 | 3.43E-06 | -0.0835 | 0.098 | 0.179 | 0.106666 | 0 |
| Trim12a | 3.44E-06 | -0.06874 | 0.217 | 0.341 | 0.106718 | 0 |
| Ndufab1 | 3.46E-06 | -0.0781 | 0.158 | 0.264 | 0.107373 | 0 |
| Baz2b | 3.51E-06 | -0.11047 | 0.316 | 0.462 | 0.108868 | 0 |
| Rab24 | 3.51E-06 | -0.08069 | 0.068 | 0.136 | 0.109124 | 0 |
| Tmem123 | 3.57E-06 | -0.12065 | 0.326 | 0.471 | 0.11092 | 0 |
| Mbtps1 | 3.59E-06 | -0.11313 | 0.08 | 0.153 | 0.111414 | 0 |
| Fbrs | 3.59E-06 | -0.06884 | 0.104 | 0.189 | 0.111435 | 0 |
| Vrk1 | 3.62E-06 | -0.07126 | 0.072 | 0.142 | 0.112495 | 0 |
| Egln1 | 3.65E-06 | -0.07141 | 0.047 | 0.105 | 0.113253 | 0 |
| Zeb1 | 3.68E-06 | -0.10864 | 0.114 | 0.2 | 0.114205 | 0 |
| Ik | 3.69E-06 | -0.08439 | 0.158 | 0.264 | 0.114578 | 0 |
| Phf3 | 3.74E-06 | -0.11389 | 0.272 | 0.406 | 0.116093 | 0 |
| Gabpb2 | 3.74E-06 | -0.11019 | 0.177 | 0.283 | 0.11628 | 0 |
| Setbp1 | 3.75E-06 | -0.13155 | 0.051 | 0.109 | 0.116537 | 0 |
| Wipf1 | 3.76E-06 | -0.0931 | 0.141 | 0.239 | 0.11668 | 0 |
| Gle1 | 3.76E-06 | -0.10499 | 0.085 | 0.16 | 0.116788 | 0 |
| Rbm26 | 3.77E-06 | -0.09503 | 0.168 | 0.277 | 0.117209 | 0 |
| Msi2 | 3.78E-06 | -0.08463 | 0.193 | 0.31 | 0.117231 | 0 |
| Cebpz | 3.79E-06 | -0.09312 | 0.254 | 0.395 | 0.117653 | 0 |
| Pum1 | 3.80E-06 | -0.09686 | 0.185 | 0.298 | 0.118101 | 0 |
| Zkscan3 | 3.81E-06 | -0.05045 | 0.066 | 0.135 | 0.118463 | 0 |
| Btaf1 | 3.84E-06 | -0.09488 | 0.05 | 0.109 | 0.119102 | 0 |
| Psme4 | 3.85E-06 | -0.0729 | 0.07 | 0.138 | 0.119608 | 0 |
| Eef1a1 | 3.86E-06 | 0.086923 | 1 | 0.998 | 0.119841 | 0 |
| Apobec1 | 3.87E-06 | -0.09135 | 0.076 | 0.147 | 0.120177 | 0 |
| Yif1b | 3.88E-06 | -0.07605 | 0.047 | 0.104 | 0.120591 | 0 |
| Apc | 3.89E-06 | -0.1027 | 0.123 | 0.212 | 0.120678 | 0 |
| Aida | 3.89E-06 | -0.07326 | 0.087 | 0.165 | 0.120923 | 0 |
| Brd1 | 3.91E-06 | -0.13114 | 0.204 | 0.32 | 0.12153 | 0 |
| Psmd2 | 3.95E-06 | -0.08247 | 0.104 | 0.187 | 0.122612 | 0 |
| Glud1 | 3.96E-06 | -0.04056 | 0.151 | 0.255 | 0.122827 | 0 |
| Rassf4 | 3.97E-06 | -0.0982 | 0.051 | 0.11 | 0.123361 | 0 |
| Tmem245 | 3.99E-06 | -0.07136 | 0.115 | 0.203 | 0.123766 | 0 |
| Myl12a | 4.01E-06 | -0.07066 | 0.288 | 0.446 | 0.124604 | 0 |
| Map4 | 4.03E-06 | -0.06265 | 0.071 | 0.141 | 0.125126 | 0 |
| Ythdc1 | 4.17E-06 | -0.10214 | 0.309 | 0.458 | 0.129461 | 0 |
| Tmem131l | 4.21E-06 | -0.11057 | 0.196 | 0.31 | 0.130796 | 0 |
| Vps13b | 4.36E-06 | -0.07565 | 0.077 | 0.149 | 0.135344 | 0 |
| Stag1 | 4.36E-06 | -0.10462 | 0.159 | 0.26 | 0.135514 | 0 |
| Rbm22 | 4.42E-06 | -0.0433 | 0.132 | 0.229 | 0.137322 | 0 |
| Mrpl17 | 4.47E-06 | -0.05874 | 0.051 | 0.11 | 0.138783 | 0 |
| Fkbp15 | 4.47E-06 | -0.05175 | 0.083 | 0.159 | 0.138952 | 0 |
| Dennd4b | 4.48E-06 | -0.06182 | 0.051 | 0.11 | 0.139024 | 0 |
| Spop | 4.48E-06 | -0.07258 | 0.143 | 0.243 | 0.139121 | 0 |
| Asnsd1 | 4.49E-06 | -0.07381 | 0.076 | 0.147 | 0.139421 | 0 |
| Ep300 | 4.54E-06 | -0.10153 | 0.212 | 0.334 | 0.140896 | 0 |
| Ss18 | 4.60E-06 | -0.08953 | 0.076 | 0.146 | 0.142844 | 0 |
| Fam117b | 4.64E-06 | -0.08875 | 0.138 | 0.232 | 0.14412 | 0 |
| Kdm3b | 4.65E-06 | -0.06996 | 0.08 | 0.153 | 0.144268 | 0 |
| Idh3b | 4.65E-06 | -0.07391 | 0.11 | 0.196 | 0.144523 | 0 |
| Ergic2 | 4.66E-06 | -0.07419 | 0.105 | 0.189 | 0.144635 | 0 |
| Snf8 | 4.74E-06 | -0.05466 | 0.098 | 0.179 | 0.147074 | 0 |
| Smarca4 | 4.74E-06 | -0.04265 | 0.114 | 0.204 | 0.147075 | 0 |
| Brk1 | 4.75E-06 | -0.09814 | 0.268 | 0.411 | 0.147383 | 0 |
| Zc3h15 | 4.84E-06 | -0.09191 | 0.242 | 0.37 | 0.150342 | 0 |
| Tbl1xr1 | 4.85E-06 | -0.05573 | 0.112 | 0.199 | 0.150509 | 0 |
| Dnajc1 | 5.01E-06 | -0.09681 | 0.071 | 0.138 | 0.155507 | 0 |
| Plxnc1 | 5.05E-06 | -0.06404 | 0.047 | 0.104 | 0.156687 | 0 |
| Psme3 | 5.13E-06 | -0.06591 | 0.067 | 0.135 | 0.15944 | 0 |
| Il6st | 5.14E-06 | -0.05934 | 0.062 | 0.126 | 0.159595 | 0 |
| Sash3 | 5.22E-06 | -0.08318 | 0.119 | 0.206 | 0.162233 | 0 |
| Ddb1 | 5.24E-06 | -0.05559 | 0.082 | 0.156 | 0.162828 | 0 |
| Atp5g1 | 5.25E-06 | -0.12028 | 0.302 | 0.446 | 0.163147 | 0 |
| Ssbp1 | 5.27E-06 | -0.08516 | 0.122 | 0.21 | 0.163582 | 0 |
| H2-Q4 | 5.29E-06 | 0.299318 | 0.425 | 0.395 | 0.164277 | 0 |
| Ndufa12 | 5.34E-06 | -0.07243 | 0.133 | 0.228 | 0.16592 | 0 |
| Ubr4 | 5.35E-06 | -0.04149 | 0.099 | 0.183 | 0.166023 | 0 |
| Fh1 | 5.37E-06 | -0.06106 | 0.075 | 0.146 | 0.166605 | 0 |
| Hnrnpm | 5.39E-06 | -0.09659 | 0.266 | 0.408 | 0.167336 | 0 |
| Paics | 5.39E-06 | -0.05483 | 0.068 | 0.136 | 0.167445 | 0 |
| Cblb | 5.45E-06 | -0.12954 | 0.206 | 0.317 | 0.16913 | 0 |
| Psmd14 | 5.48E-06 | -0.09866 | 0.103 | 0.183 | 0.170049 | 0 |
| Mrpl18 | 5.53E-06 | -0.07859 | 0.109 | 0.193 | 0.171683 | 0 |
| Eif5a | 5.53E-06 | -0.15735 | 0.471 | 0.622 | 0.171687 | 0 |
| March2 | 5.54E-06 | -0.0559 | 0.199 | 0.325 | 0.171968 | 0 |
| Mcrip1 | 5.65E-06 | -0.07015 | 0.092 | 0.169 | 0.175432 | 0 |
| Hmox1 | 5.68E-06 | 0.252192 | 0.119 | 0.061 | 0.176477 | 0 |
| Cyfip2 | 5.69E-06 | -0.07014 | 0.207 | 0.329 | 0.176743 | 0 |
| Psmb7 | 5.71E-06 | -0.07146 | 0.123 | 0.215 | 0.177449 | 0 |
| Rpl22 | 5.73E-06 | 0.108585 | 0.989 | 0.99 | 0.177784 | 0 |
| Trib1 | 5.73E-06 | -0.08584 | 0.072 | 0.14 | 0.17784 | 0 |
| Cd19 | 5.73E-06 | -0.10678 | 0.36 | 0.523 | 0.178032 | 0 |
| Txnl4a | 5.76E-06 | -0.07526 | 0.051 | 0.109 | 0.178714 | 0 |
| Mphosph10 | 5.78E-06 | -0.10387 | 0.119 | 0.204 | 0.179526 | 0 |
| Mri1 | 5.80E-06 | -0.05384 | 0.059 | 0.122 | 0.180112 | 0 |
| Anapc15 | 5.94E-06 | -0.06912 | 0.05 | 0.107 | 0.184415 | 0 |
| Nop58 | 6.10E-06 | -0.08685 | 0.147 | 0.246 | 0.18928 | 0 |
| Ppp4c | 6.10E-06 | -0.08583 | 0.139 | 0.235 | 0.189491 | 0 |
| Anxa7 | 6.17E-06 | -0.08758 | 0.082 | 0.154 | 0.19163 | 0 |
| Ivns1abp | 6.24E-06 | -0.04376 | 0.199 | 0.319 | 0.193751 | 0 |
| Gxylt1 | 6.25E-06 | -0.07529 | 0.09 | 0.165 | 0.194168 | 0 |
| Snx8 | 6.28E-06 | -0.07041 | 0.089 | 0.165 | 0.194931 | 0 |
| Mrto4 | 6.28E-06 | -0.08134 | 0.051 | 0.109 | 0.19506 | 0 |
| Ddx41 | 6.31E-06 | -0.08165 | 0.122 | 0.209 | 0.196081 | 0 |
| Ppip5k2 | 6.31E-06 | -0.07153 | 0.047 | 0.103 | 0.19609 | 0 |
| Rgs1 | 6.34E-06 | 0.252661 | 0.154 | 0.089 | 0.196921 | 0 |
| Csnk2a1 | 6.34E-06 | -0.10139 | 0.181 | 0.288 | 0.196948 | 0 |
| Nfu1 | 6.35E-06 | -0.04933 | 0.08 | 0.152 | 0.19717 | 0 |
| Larp4b | 6.42E-06 | -0.09239 | 0.154 | 0.253 | 0.199334 | 0 |
| Lrba | 6.45E-06 | -0.09123 | 0.057 | 0.118 | 0.200257 | 0 |
| Ddx39b | 6.46E-06 | -0.10294 | 0.2 | 0.314 | 0.200485 | 0 |
| Ifngr1 | 6.49E-06 | -0.07061 | 0.142 | 0.237 | 0.201487 | 0 |
| Hspa9 | 6.51E-06 | -0.07947 | 0.145 | 0.243 | 0.202003 | 0 |
| Ap1g1 | 6.56E-06 | -0.07264 | 0.064 | 0.128 | 0.20374 | 0 |
| Dmxl1 | 6.58E-06 | -0.10367 | 0.333 | 0.481 | 0.204318 | 0 |
| Nr2c2 | 6.59E-06 | -0.0889 | 0.109 | 0.191 | 0.204766 | 0 |
| Ppan | 6.61E-06 | -0.11079 | 0.05 | 0.106 | 0.205339 | 0 |
| Parg | 6.63E-06 | -0.11791 | 0.053 | 0.111 | 0.205941 | 0 |
| Brix1 | 6.66E-06 | -0.11764 | 0.082 | 0.153 | 0.206855 | 0 |
| Htt | 6.69E-06 | -0.0476 | 0.058 | 0.121 | 0.207886 | 0 |
| Arhgdib | 6.75E-06 | -0.12916 | 0.767 | 0.883 | 0.209549 | 0 |
| Csk | 6.89E-06 | -0.10145 | 0.287 | 0.432 | 0.21403 | 0 |
| Rpl27 | 6.90E-06 | 0.098945 | 0.973 | 0.979 | 0.214156 | 0 |
| Atrx | 6.90E-06 | -0.10723 | 0.332 | 0.477 | 0.214161 | 0 |
| Mrps36 | 6.96E-06 | -0.0638 | 0.087 | 0.161 | 0.216007 | 0 |
| Slc35b1 | 6.98E-06 | -0.08589 | 0.06 | 0.122 | 0.216864 | 0 |
| Phf20l1 | 7.07E-06 | -0.08876 | 0.232 | 0.362 | 0.21962 | 0 |
| Chd7 | 7.19E-06 | -0.11687 | 0.152 | 0.247 | 0.223294 | 0 |
| Dync1i2 | 7.21E-06 | -0.06827 | 0.168 | 0.272 | 0.223741 | 0 |
| Ccnk | 7.21E-06 | -0.07165 | 0.047 | 0.103 | 0.223985 | 0 |
| Nol11 | 7.27E-06 | -0.08235 | 0.057 | 0.118 | 0.225618 | 0 |
| Hbp1 | 7.28E-06 | -0.07407 | 0.168 | 0.273 | 0.226112 | 0 |
| Zcchc2 | 7.30E-06 | -0.0618 | 0.05 | 0.107 | 0.226636 | 0 |
| Ucp2 | 7.38E-06 | -0.11483 | 0.384 | 0.537 | 0.229107 | 0 |
| Taf1 | 7.38E-06 | -0.09232 | 0.093 | 0.168 | 0.229284 | 0 |
| Ap1m1 | 7.43E-06 | -0.04379 | 0.093 | 0.171 | 0.23081 | 0 |
| Pigt | 7.48E-06 | -0.04771 | 0.066 | 0.132 | 0.232338 | 0 |
| Arhgef1 | 7.60E-06 | -0.11271 | 0.335 | 0.486 | 0.236131 | 0 |
| Ltv1 | 7.65E-06 | -0.08255 | 0.055 | 0.115 | 0.237659 | 0 |
| Cd2bp2 | 7.69E-06 | -0.06217 | 0.058 | 0.119 | 0.238698 | 0 |
| AI314180 | 7.70E-06 | -0.08048 | 0.087 | 0.161 | 0.239238 | 0 |
| H13 | 7.79E-06 | -0.13508 | 0.118 | 0.2 | 0.24183 | 0 |
| Odf2 | 7.91E-06 | -0.09211 | 0.066 | 0.13 | 0.245731 | 0 |
| Ebag9 | 8.07E-06 | -0.05328 | 0.06 | 0.123 | 0.25073 | 0 |
| Cers2 | 8.18E-06 | -0.0574 | 0.118 | 0.205 | 0.254135 | 0 |
| Lsm7 | 8.21E-06 | -0.09571 | 0.073 | 0.14 | 0.255087 | 0 |
| Pik3cg | 8.23E-06 | -0.06749 | 0.048 | 0.104 | 0.255563 | 0 |
| Zfp622 | 8.41E-06 | -0.09556 | 0.138 | 0.232 | 0.261047 | 0 |
| Gpd1l | 8.64E-06 | -0.07948 | 0.09 | 0.165 | 0.268177 | 0 |
| Exosc3 | 8.68E-06 | -0.0657 | 0.085 | 0.159 | 0.269434 | 0 |
| Adprh | 8.69E-06 | -0.0803 | 0.057 | 0.117 | 0.269765 | 0 |
| Psmb10 | 8.70E-06 | -0.05618 | 0.18 | 0.288 | 0.270297 | 0 |
| Pdzd8 | 8.71E-06 | -0.0885 | 0.082 | 0.153 | 0.27053 | 0 |
| Inpp5k | 8.72E-06 | -0.07909 | 0.079 | 0.148 | 0.270629 | 0 |
| Smad4 | 8.91E-06 | -0.06892 | 0.11 | 0.192 | 0.276635 | 0 |
| Taldo1 | 8.92E-06 | -0.09604 | 0.32 | 0.476 | 0.276962 | 0 |
| Ubald2 | 8.94E-06 | -0.07589 | 0.187 | 0.298 | 0.277492 | 0 |
| Ddx54 | 8.96E-06 | -0.05862 | 0.184 | 0.295 | 0.27815 | 0 |
| L3mbtl3 | 9.12E-06 | -0.10651 | 0.056 | 0.115 | 0.283167 | 0 |
| Pkp3 | 9.13E-06 | -0.05904 | 0.05 | 0.106 | 0.283585 | 0 |
| Trip12 | 9.26E-06 | -0.06951 | 0.191 | 0.305 | 0.287664 | 0 |
| Eif5b | 9.34E-06 | -0.1259 | 0.273 | 0.4 | 0.289952 | 0 |
| Hectd1 | 9.34E-06 | -0.10062 | 0.229 | 0.35 | 0.289965 | 0 |
| Dcaf12 | 9.35E-06 | -0.06325 | 0.065 | 0.13 | 0.2905 | 0 |
| Ctdnep1 | 9.42E-06 | -0.08071 | 0.081 | 0.152 | 0.292474 | 0 |
| Ccl5 | 9.43E-06 | 0.312721 | 0.172 | 0.109 | 0.292698 | 0 |
| Ptpn1 | 9.46E-06 | -0.12293 | 0.356 | 0.508 | 0.293879 | 0 |
| Slc39a7 | 9.48E-06 | -0.07971 | 0.051 | 0.107 | 0.294397 | 0 |
| Rad21 | 9.50E-06 | -0.06136 | 0.161 | 0.267 | 0.295138 | 0 |
| Orc4 | 9.52E-06 | -0.07716 | 0.065 | 0.129 | 0.295658 | 0 |
| Smg7 | 9.60E-06 | -0.08988 | 0.08 | 0.149 | 0.29821 | 0 |
| Ccdc50 | 9.70E-06 | -0.062 | 0.141 | 0.237 | 0.30111 | 0 |
| Erbin | 9.73E-06 | -0.09832 | 0.149 | 0.246 | 0.302063 | 0 |
| Sf3b1 | 9.73E-06 | -0.13217 | 0.529 | 0.708 | 0.302122 | 0 |
| Rnf113a2 | 9.78E-06 | -0.08757 | 0.052 | 0.11 | 0.303553 | 0 |
| Scml4 | 9.83E-06 | -0.08169 | 0.057 | 0.117 | 0.305285 | 0 |
| Slc38a10 | 9.83E-06 | -0.06127 | 0.051 | 0.107 | 0.30532 | 0 |
| Ttc5 | 9.94E-06 | -0.08186 | 0.064 | 0.126 | 0.308721 | 0 |
| Mrpl50 | 9.96E-06 | -0.06503 | 0.051 | 0.107 | 0.309351 | 0 |
| Anp32a | 9.98E-06 | -0.10687 | 0.312 | 0.461 | 0.309834 | 0 |
| Sec24c | 1.00E-05 | -0.08425 | 0.07 | 0.135 | 0.311439 | 0 |
| Lsm6 | 1.01E-05 | -0.11103 | 0.173 | 0.273 | 0.314669 | 0 |
| Txndc5 | 1.03E-05 | -0.21495 | 0.114 | 0.195 | 0.320121 | 0 |
| Rasal3 | 1.03E-05 | -0.0908 | 0.087 | 0.159 | 0.32054 | 0 |
| Wdr61 | 1.04E-05 | -0.07869 | 0.06 | 0.121 | 0.322409 | 0 |
| R3hdm2 | 1.06E-05 | -0.12659 | 0.129 | 0.214 | 0.329815 | 0 |
| Otulin | 1.07E-05 | -0.06373 | 0.167 | 0.272 | 0.332351 | 0 |
| Snrpc | 1.07E-05 | -0.07033 | 0.152 | 0.251 | 0.332592 | 0 |
| Appl1 | 1.07E-05 | -0.06119 | 0.095 | 0.172 | 0.33329 | 0 |
| Cir1 | 1.08E-05 | -0.10451 | 0.094 | 0.168 | 0.335057 | 0 |
| Sar1a | 1.09E-05 | -0.05131 | 0.133 | 0.226 | 0.337171 | 0 |
| Twistnb | 1.09E-05 | -0.09246 | 0.137 | 0.228 | 0.33747 | 0 |
| Cyfip1 | 1.09E-05 | -0.05775 | 0.065 | 0.128 | 0.337747 | 0 |
| Selenop | 1.10E-05 | 0.258923 | 0.142 | 0.082 | 0.341632 | 0 |
| Rhof | 1.10E-05 | -0.05318 | 0.051 | 0.109 | 0.342708 | 0 |
| Mrpl21 | 1.11E-05 | -0.08308 | 0.097 | 0.174 | 0.343882 | 0 |
| Lnpep | 1.11E-05 | -0.08582 | 0.231 | 0.353 | 0.34449 | 0 |
| Fam168b | 1.11E-05 | -0.05913 | 0.163 | 0.265 | 0.345484 | 0 |
| Trpm7 | 1.11E-05 | -0.09738 | 0.135 | 0.223 | 0.345755 | 0 |
| Psma4 | 1.12E-05 | -0.08556 | 0.184 | 0.294 | 0.349174 | 0 |
| Eif2s2 | 1.13E-05 | -0.10798 | 0.408 | 0.558 | 0.349407 | 0 |
| Washc2 | 1.13E-05 | -0.12296 | 0.113 | 0.192 | 0.351391 | 0 |
| Gnl2 | 1.14E-05 | -0.09395 | 0.061 | 0.122 | 0.354526 | 0 |
| Zmym2 | 1.17E-05 | -0.06477 | 0.069 | 0.135 | 0.362869 | 0 |
| Bag5 | 1.18E-05 | -0.08391 | 0.054 | 0.112 | 0.365105 | 0 |
| Exosc5 | 1.18E-05 | -0.07033 | 0.072 | 0.137 | 0.366766 | 0 |
| Rc3h2 | 1.18E-05 | -0.06502 | 0.119 | 0.204 | 0.36736 | 0 |
| Psmd13 | 1.19E-05 | -0.04912 | 0.113 | 0.197 | 0.370352 | 0 |
| Rbm28 | 1.20E-05 | -0.08365 | 0.103 | 0.181 | 0.372527 | 0 |
| Rubcn | 1.20E-05 | -0.08852 | 0.091 | 0.165 | 0.372565 | 0 |
| Ets1 | 1.20E-05 | -0.1758 | 0.7 | 0.801 | 0.374061 | 0 |
| Syvn1 | 1.22E-05 | -0.08104 | 0.102 | 0.18 | 0.378211 | 0 |
| Topbp1 | 1.22E-05 | -0.06097 | 0.078 | 0.146 | 0.378848 | 0 |
| Stk11 | 1.22E-05 | -0.03181 | 0.126 | 0.217 | 0.37991 | 0 |
| Ube2q1 | 1.23E-05 | -0.07125 | 0.123 | 0.211 | 0.383154 | 0 |
| Ganab | 1.25E-05 | -0.03138 | 0.063 | 0.125 | 0.387491 | 0 |
| Pds5a | 1.25E-05 | -0.06538 | 0.175 | 0.28 | 0.388693 | 0 |
| Dkc1 | 1.26E-05 | -0.06824 | 0.064 | 0.125 | 0.391145 | 0 |
| Camk1d | 1.26E-05 | -0.07165 | 0.059 | 0.118 | 0.391962 | 0 |
| Prkacb | 1.27E-05 | -0.06006 | 0.085 | 0.156 | 0.392898 | 0 |
| Golgb1 | 1.29E-05 | -0.10171 | 0.094 | 0.167 | 0.399959 | 0 |
| Ppil4 | 1.29E-05 | -0.09591 | 0.124 | 0.209 | 0.40207 | 0 |
| Ppid | 1.30E-05 | -0.07668 | 0.062 | 0.122 | 0.403244 | 0 |
| Ssr2 | 1.31E-05 | -0.12309 | 0.119 | 0.2 | 0.406728 | 0 |
| Msl2 | 1.33E-05 | -0.05958 | 0.118 | 0.204 | 0.414112 | 0 |
| Lmnb1 | 1.33E-05 | -0.09181 | 0.065 | 0.126 | 0.414158 | 0 |
| Mtch1 | 1.34E-05 | -0.04241 | 0.094 | 0.171 | 0.414847 | 0 |
| Pafah1b1 | 1.34E-05 | -0.08675 | 0.34 | 0.493 | 0.41711 | 0 |
| Taf10 | 1.35E-05 | -0.1048 | 0.281 | 0.418 | 0.419148 | 0 |
| Gmip | 1.36E-05 | -0.06398 | 0.128 | 0.216 | 0.422594 | 0 |
| Med10 | 1.37E-05 | -0.06082 | 0.088 | 0.16 | 0.424962 | 0 |
| Ddx18 | 1.38E-05 | -0.12472 | 0.146 | 0.236 | 0.427219 | 0 |
| Atxn7 | 1.38E-05 | -0.07902 | 0.081 | 0.15 | 0.428922 | 0 |
| Acadl | 1.39E-05 | -0.03664 | 0.096 | 0.172 | 0.430475 | 0 |
| AI467606 | 1.40E-05 | -0.08477 | 0.077 | 0.143 | 0.433367 | 0 |
| Ppdpf | 1.40E-05 | -0.07797 | 0.261 | 0.399 | 0.434284 | 0 |
| Glul | 1.40E-05 | -0.07638 | 0.097 | 0.173 | 0.434467 | 0 |
| Gpcpd1 | 1.40E-05 | -0.06342 | 0.231 | 0.359 | 0.435544 | 0 |
| Cdc42se2 | 1.40E-05 | -0.08698 | 0.224 | 0.34 | 0.436048 | 0 |
| Dpp8 | 1.41E-05 | -0.10003 | 0.1 | 0.175 | 0.43667 | 0 |
| Eri1 | 1.42E-05 | -0.05176 | 0.081 | 0.152 | 0.441893 | 0 |
| Dnm1l | 1.43E-05 | -0.08536 | 0.077 | 0.143 | 0.443227 | 0 |
| Rad23a | 1.44E-05 | -0.05809 | 0.073 | 0.138 | 0.445828 | 0 |
| Cnp | 1.44E-05 | -0.13197 | 0.243 | 0.363 | 0.446573 | 0 |
| Cdc40 | 1.44E-05 | -0.09821 | 0.182 | 0.285 | 0.448359 | 0 |
| Ankhd1 | 1.45E-05 | -0.06555 | 0.136 | 0.226 | 0.448889 | 0 |
| Dgkz | 1.45E-05 | -0.09092 | 0.075 | 0.141 | 0.451104 | 0 |
| Tex261 | 1.46E-05 | -0.05538 | 0.096 | 0.172 | 0.454619 | 0 |
| Cobll1 | 1.47E-05 | -0.08794 | 0.053 | 0.11 | 0.45665 | 0 |
| Ehd4 | 1.47E-05 | -0.04095 | 0.063 | 0.125 | 0.457169 | 0 |
| Tgfbi | 1.48E-05 | 0.267872 | 0.141 | 0.082 | 0.458191 | 0 |
| Parp4 | 1.48E-05 | -0.0772 | 0.052 | 0.109 | 0.45997 | 0 |
| Cxxc5 | 1.49E-05 | -0.11339 | 0.088 | 0.158 | 0.461781 | 0 |
| Stk24 | 1.49E-05 | -0.07311 | 0.301 | 0.445 | 0.462768 | 0 |
| Nfrkb | 1.52E-05 | -0.06951 | 0.065 | 0.126 | 0.472751 | 0 |
| Timm44 | 1.54E-05 | -0.08724 | 0.057 | 0.115 | 0.478648 | 0 |
| Txn2 | 1.54E-05 | -0.09248 | 0.123 | 0.206 | 0.478809 | 0 |
| Ppp2ca | 1.57E-05 | -0.07524 | 0.287 | 0.43 | 0.488543 | 0 |
| Ubr1 | 1.58E-05 | -0.08796 | 0.053 | 0.11 | 0.489882 | 0 |
| Rsl1d1 | 1.58E-05 | -0.07216 | 0.128 | 0.215 | 0.490227 | 0 |
| Arih1 | 1.59E-05 | -0.06529 | 0.193 | 0.304 | 0.492399 | 0 |
| Cs | 1.59E-05 | -0.06179 | 0.108 | 0.187 | 0.492728 | 0 |
| Snw1 | 1.59E-05 | -0.09203 | 0.24 | 0.362 | 0.49326 | 0 |
| Asxl2 | 1.59E-05 | -0.06731 | 0.148 | 0.242 | 0.494065 | 0 |
| Zufsp | 1.60E-05 | -0.08621 | 0.138 | 0.226 | 0.496172 | 0 |
| Ash1l | 1.63E-05 | -0.1037 | 0.332 | 0.481 | 0.506968 | 0 |
| March7 | 1.64E-05 | -0.06684 | 0.256 | 0.387 | 0.50773 | 0 |
| Ptpn11 | 1.64E-05 | -0.05104 | 0.059 | 0.118 | 0.5104 | 0 |
| Dhx8 | 1.66E-05 | -0.07439 | 0.072 | 0.136 | 0.514178 | 0 |
| Gpatch8 | 1.66E-05 | -0.06539 | 0.18 | 0.286 | 0.516407 | 0 |
| Prelid1 | 1.68E-05 | -0.0623 | 0.174 | 0.278 | 0.522596 | 0 |
| Fem1c | 1.70E-05 | -0.07596 | 0.055 | 0.112 | 0.526698 | 0 |
| Smg1 | 1.70E-05 | -0.1139 | 0.313 | 0.45 | 0.527485 | 0 |
| Becn1 | 1.72E-05 | -0.05855 | 0.098 | 0.174 | 0.534824 | 0 |
| Dido1 | 1.72E-05 | -0.05976 | 0.102 | 0.18 | 0.535353 | 0 |
| Usp25 | 1.73E-05 | -0.07178 | 0.124 | 0.209 | 0.536734 | 0 |
| Bsdc1 | 1.73E-05 | -0.05657 | 0.075 | 0.142 | 0.538682 | 0 |
| Glrx3 | 1.78E-05 | -0.05081 | 0.118 | 0.202 | 0.553115 | 0 |
| Slmap | 1.80E-05 | -0.0687 | 0.054 | 0.111 | 0.558355 | 0 |
| Slc9a8 | 1.81E-05 | -0.0856 | 0.049 | 0.103 | 0.561117 | 0 |
| Dusp11 | 1.82E-05 | -0.07442 | 0.275 | 0.413 | 0.566121 | 0 |
| Cox20 | 1.84E-05 | -0.08605 | 0.102 | 0.178 | 0.572718 | 0 |
| Lsm4 | 1.85E-05 | -0.06819 | 0.264 | 0.397 | 0.574271 | 0 |
| Dhx38 | 1.85E-05 | -0.06928 | 0.056 | 0.113 | 0.575357 | 0 |
| Ankrd13a | 1.85E-05 | -0.03274 | 0.115 | 0.199 | 0.575685 | 0 |
| Cybb | 1.88E-05 | 0.304794 | 0.493 | 0.47 | 0.583504 | 0 |
| Irf2bp2 | 1.89E-05 | -0.0933 | 0.158 | 0.251 | 0.585572 | 0 |
| Ifnar1 | 1.89E-05 | -0.08332 | 0.123 | 0.206 | 0.586209 | 0 |
| Trim30a | 1.89E-05 | -0.14149 | 0.109 | 0.185 | 0.586909 | 0 |
| Rac2 | 1.89E-05 | -0.11809 | 0.558 | 0.723 | 0.586921 | 0 |
| Ndufaf4 | 1.89E-05 | -0.06909 | 0.051 | 0.105 | 0.587099 | 0 |
| Ap4s1 | 1.91E-05 | -0.0504 | 0.054 | 0.111 | 0.59403 | 0 |
| Fip1l1 | 1.93E-05 | -0.06855 | 0.191 | 0.297 | 0.59944 | 0 |
| Pcf11 | 1.93E-05 | -0.10157 | 0.224 | 0.34 | 0.600478 | 0 |
| 2900097C17Rik | 1.94E-05 | -0.05318 | 0.088 | 0.159 | 0.603356 | 0 |
| Vps13d | 1.94E-05 | -0.09806 | 0.061 | 0.119 | 0.603884 | 0 |
| Jtb | 1.95E-05 | -0.06221 | 0.15 | 0.243 | 0.606334 | 0 |
| Sp100 | 1.95E-05 | -0.14264 | 0.407 | 0.551 | 0.606468 | 0 |
| Dtymk | 1.96E-05 | -0.06625 | 0.051 | 0.105 | 0.608577 | 0 |
| Zfand6 | 1.96E-05 | -0.06087 | 0.191 | 0.3 | 0.608968 | 0 |
| Nme2 | 1.96E-05 | -0.04383 | 0.053 | 0.11 | 0.609359 | 0 |
| Dlst | 1.97E-05 | -0.04767 | 0.107 | 0.185 | 0.61119 | 0 |
| Rrm2b | 1.97E-05 | -0.09657 | 0.062 | 0.121 | 0.612363 | 0 |
| Rnf7 | 1.97E-05 | -0.06155 | 0.123 | 0.208 | 0.612411 | 0 |
| G3bp1 | 1.97E-05 | -0.07941 | 0.241 | 0.362 | 0.612702 | 0 |
| Rnf139 | 1.98E-05 | -0.06927 | 0.064 | 0.124 | 0.615806 | 0 |
| Pdcd2 | 2.03E-05 | -0.0687 | 0.051 | 0.105 | 0.630024 | 0 |
| Rps27rt | 2.04E-05 | 0.179536 | 0.784 | 0.822 | 0.632432 | 0 |
| Stard7 | 2.05E-05 | -0.06702 | 0.058 | 0.116 | 0.637097 | 0 |
| Ybx1 | 2.06E-05 | -0.14156 | 0.63 | 0.783 | 0.640377 | 0 |
| Pdcd11 | 2.07E-05 | -0.04824 | 0.054 | 0.11 | 0.64386 | 0 |
| Psma6 | 2.07E-05 | -0.06699 | 0.163 | 0.26 | 0.643969 | 0 |
| Prpf18 | 2.07E-05 | -0.05433 | 0.051 | 0.105 | 0.644188 | 0 |
| Gtpbp4 | 2.09E-05 | -0.0416 | 0.16 | 0.259 | 0.648258 | 0 |
| Chuk | 2.11E-05 | -0.06324 | 0.095 | 0.169 | 0.655827 | 0 |
| Stk38 | 2.13E-05 | -0.09121 | 0.186 | 0.29 | 0.661109 | 0 |
| Ostc | 2.14E-05 | -0.08589 | 0.162 | 0.257 | 0.664147 | 0 |
| Khsrp | 2.14E-05 | -0.06952 | 0.072 | 0.136 | 0.665216 | 0 |
| Eif1ax | 2.14E-05 | -0.05897 | 0.117 | 0.198 | 0.665504 | 0 |
| Med8 | 2.16E-05 | -0.05096 | 0.06 | 0.119 | 0.669987 | 0 |
| Ndufb6 | 2.16E-05 | -0.09501 | 0.109 | 0.184 | 0.67069 | 0 |
| Ptpa | 2.21E-05 | -0.08177 | 0.09 | 0.16 | 0.687549 | 0 |
| Nelfb | 2.24E-05 | -0.06683 | 0.059 | 0.117 | 0.697122 | 0 |
| Taf3 | 2.29E-05 | -0.09025 | 0.083 | 0.15 | 0.710238 | 0 |
| Qars | 2.30E-05 | -0.06918 | 0.071 | 0.134 | 0.713465 | 0 |
| Zfp800 | 2.30E-05 | -0.07909 | 0.109 | 0.186 | 0.714328 | 0 |
| H2afy | 2.31E-05 | -0.10336 | 0.33 | 0.477 | 0.717354 | 0 |
| Htatsf1 | 2.35E-05 | -0.09268 | 0.051 | 0.105 | 0.7289 | 0 |
| Gm16286 | 2.38E-05 | -0.07897 | 0.165 | 0.261 | 0.739853 | 0 |
| Rprd2 | 2.38E-05 | -0.04311 | 0.113 | 0.195 | 0.740598 | 0 |
| Dcps | 2.39E-05 | -0.08052 | 0.063 | 0.122 | 0.741894 | 0 |
| Mrps23 | 2.39E-05 | -0.04519 | 0.07 | 0.134 | 0.742063 | 0 |
| Fibp | 2.39E-05 | -0.06476 | 0.051 | 0.106 | 0.743693 | 0 |
| Eftud2 | 2.40E-05 | -0.09528 | 0.056 | 0.111 | 0.746201 | 0 |
| Pik3c2b | 2.41E-05 | -0.04969 | 0.051 | 0.106 | 0.748844 | 0 |
| Casp8ap2 | 2.42E-05 | -0.0621 | 0.092 | 0.162 | 0.751954 | 0 |
| Idh3g | 2.45E-05 | -0.02869 | 0.166 | 0.27 | 0.761206 | 0 |
| Dynll1 | 2.45E-05 | 0.25032 | 0.604 | 0.604 | 0.762118 | 0 |
| Ap1b1 | 2.46E-05 | -0.08591 | 0.051 | 0.104 | 0.765067 | 0 |
| Cdk19 | 2.49E-05 | -0.05436 | 0.082 | 0.152 | 0.772758 | 0 |
| Phrf1 | 2.50E-05 | -0.04946 | 0.068 | 0.131 | 0.776624 | 0 |
| Gimap4 | 2.55E-05 | -0.12811 | 0.244 | 0.358 | 0.791703 | 0 |
| Med12 | 2.55E-05 | -0.06092 | 0.08 | 0.147 | 0.792969 | 0 |
| Ccdc12 | 2.57E-05 | -0.07376 | 0.215 | 0.331 | 0.797634 | 0 |
| Nsun2 | 2.57E-05 | -0.03019 | 0.067 | 0.13 | 0.79903 | 0 |
| B3gat3 | 2.59E-05 | -0.07163 | 0.055 | 0.111 | 0.804177 | 0 |
| Ero1lb | 2.60E-05 | -0.07007 | 0.202 | 0.315 | 0.806666 | 0 |
| Mgat2 | 2.61E-05 | -0.06394 | 0.085 | 0.153 | 0.809589 | 0 |
| Rftn1 | 2.61E-05 | -0.07284 | 0.092 | 0.162 | 0.811224 | 0 |
| Cat | 2.62E-05 | -0.06808 | 0.094 | 0.166 | 0.812279 | 0 |
| Carnmt1 | 2.63E-05 | -0.05675 | 0.054 | 0.11 | 0.817996 | 0 |
| Ddx46 | 2.65E-05 | -0.09132 | 0.214 | 0.325 | 0.821499 | 0 |
| BC005624 | 2.67E-05 | -0.09905 | 0.197 | 0.301 | 0.828478 | 0 |
| Pak1ip1 | 2.69E-05 | -0.07974 | 0.12 | 0.202 | 0.836519 | 0 |
| Snrpa1 | 2.70E-05 | -0.0565 | 0.095 | 0.168 | 0.837706 | 0 |
| Os9 | 2.71E-05 | -0.02668 | 0.075 | 0.141 | 0.843077 | 0 |
| Srrm2 | 2.73E-05 | -0.09455 | 0.59 | 0.759 | 0.84734 | 0 |
| Gm26740 | 2.73E-05 | 0.238939 | 0.442 | 0.396 | 0.848175 | 0 |
| Akt3 | 2.74E-05 | -0.11493 | 0.051 | 0.103 | 0.852102 | 0 |
| Ndufb8 | 2.75E-05 | -0.07634 | 0.2 | 0.309 | 0.853618 | 0 |
| Lactb | 2.77E-05 | -0.05747 | 0.101 | 0.175 | 0.858939 | 0 |
| Timm17a | 2.80E-05 | -0.04356 | 0.086 | 0.155 | 0.869394 | 0 |
| Pde3b | 2.84E-05 | -0.04558 | 0.16 | 0.257 | 0.88241 | 0 |
| Cwc22 | 2.86E-05 | -0.06139 | 0.055 | 0.111 | 0.888033 | 0 |
| Mrpl4 | 2.87E-05 | -0.04134 | 0.072 | 0.135 | 0.890649 | 0 |
| Mtss1 | 2.88E-05 | -0.10312 | 0.289 | 0.419 | 0.893672 | 0 |
| Psip1 | 2.90E-05 | -0.09209 | 0.145 | 0.235 | 0.899235 | 0 |
| Slu7 | 2.93E-05 | -0.06851 | 0.094 | 0.166 | 0.910708 | 0 |
| Vav1 | 2.95E-05 | -0.07443 | 0.097 | 0.169 | 0.916675 | 0 |
| Ssb | 2.96E-05 | -0.08919 | 0.311 | 0.447 | 0.918093 | 0 |
| Amfr | 2.96E-05 | -0.06799 | 0.08 | 0.144 | 0.918825 | 0 |
| Dennd1b | 2.97E-05 | -0.10198 | 0.183 | 0.284 | 0.922378 | 0 |
| Osbpl9 | 2.97E-05 | -0.05021 | 0.162 | 0.259 | 0.922662 | 0 |
| Ppig | 2.98E-05 | -0.08745 | 0.218 | 0.329 | 0.925429 | 0 |
| Arhgef18 | 3.05E-05 | -0.08715 | 0.162 | 0.258 | 0.946474 | 0 |
| Hp1bp3 | 3.08E-05 | -0.05739 | 0.205 | 0.316 | 0.954912 | 0 |
| Sav1 | 3.11E-05 | -0.04636 | 0.061 | 0.119 | 0.967047 | 0 |
| Mia3 | 3.12E-05 | -0.05007 | 0.124 | 0.208 | 0.968207 | 0 |
| Zfp260 | 3.16E-05 | -0.08295 | 0.05 | 0.101 | 0.980217 | 0 |
| Sh3bgrl | 3.16E-05 | -0.06293 | 0.139 | 0.228 | 0.981209 | 0 |
| Vti1b | 3.17E-05 | -0.05541 | 0.082 | 0.149 | 0.983111 | 0 |
| Aff1 | 3.20E-05 | -0.06464 | 0.152 | 0.246 | 0.992272 | 0 |
| Gtf3a | 3.22E-05 | -0.05614 | 0.054 | 0.109 | 0.998721 | 0 |
| Wapl | 3.22E-05 | -0.0816 | 0.179 | 0.278 | 1 | 0 |
| Chmp1a | 3.23E-05 | -0.07267 | 0.054 | 0.109 | 1 | 0 |
| Stx4a | 3.24E-05 | -0.06835 | 0.092 | 0.161 | 1 | 0 |
| Prkcd | 3.25E-05 | -0.08095 | 0.19 | 0.297 | 1 | 0 |
| Rex1bd | 3.27E-05 | -0.07023 | 0.076 | 0.14 | 1 | 0 |
| Exosc10 | 3.28E-05 | -0.05001 | 0.057 | 0.113 | 1 | 0 |
| Nap1l1 | 3.30E-05 | -0.1204 | 0.456 | 0.613 | 1 | 0 |
| Zbtb7a | 3.30E-05 | -0.08622 | 0.215 | 0.323 | 1 | 0 |
| Usp1 | 3.30E-05 | -0.06675 | 0.09 | 0.159 | 1 | 0 |
| Kars | 3.32E-05 | -0.03034 | 0.055 | 0.111 | 1 | 0 |
| Strap | 3.32E-05 | -0.04767 | 0.121 | 0.204 | 1 | 0 |
| Sdf4 | 3.32E-05 | -0.04374 | 0.169 | 0.268 | 1 | 0 |
| Arfrp1 | 3.33E-05 | -0.04358 | 0.081 | 0.149 | 1 | 0 |
| Ddx23 | 3.34E-05 | -0.0693 | 0.093 | 0.163 | 1 | 0 |
| Etf1 | 3.34E-05 | -0.02704 | 0.131 | 0.218 | 1 | 0 |
| Uhmk1 | 3.36E-05 | -0.0494 | 0.078 | 0.143 | 1 | 0 |
| Arpp19 | 3.36E-05 | -0.07943 | 0.321 | 0.473 | 1 | 0 |
| Bcl7b | 3.42E-05 | -0.06132 | 0.123 | 0.204 | 1 | 0 |
| Cdk9 | 3.45E-05 | -0.04398 | 0.149 | 0.241 | 1 | 0 |
| Trim27 | 3.48E-05 | -0.05094 | 0.102 | 0.177 | 1 | 0 |
| Esf1 | 3.48E-05 | -0.07943 | 0.123 | 0.202 | 1 | 0 |
| Lgmn | 3.49E-05 | 0.297521 | 0.264 | 0.209 | 1 | 0 |
| Hook3 | 3.50E-05 | -0.03779 | 0.107 | 0.184 | 1 | 0 |
| Tspan32 | 3.53E-05 | -0.11256 | 0.157 | 0.246 | 1 | 0 |
| 1700020I14Rik | 3.53E-05 | -0.0975 | 0.186 | 0.285 | 1 | 0 |
| Bms1 | 3.59E-05 | -0.0899 | 0.069 | 0.129 | 1 | 0 |
| Mrps21 | 3.62E-05 | -0.05515 | 0.159 | 0.254 | 1 | 0 |
| Wasf2 | 3.63E-05 | -0.0543 | 0.274 | 0.408 | 1 | 0 |
| Pcnt | 3.64E-05 | -0.0875 | 0.064 | 0.122 | 1 | 0 |
| Snx2 | 3.64E-05 | -0.12399 | 0.381 | 0.526 | 1 | 0 |
| 2610507B11Rik | 3.64E-05 | -0.07448 | 0.082 | 0.148 | 1 | 0 |
| Prmt1 | 3.68E-05 | -0.05444 | 0.152 | 0.247 | 1 | 0 |
| Malt1 | 3.73E-05 | -0.12634 | 0.389 | 0.523 | 1 | 0 |
| Irf5 | 3.73E-05 | -0.04367 | 0.1 | 0.173 | 1 | 0 |
| Nfkb1 | 3.73E-05 | -0.12346 | 0.296 | 0.412 | 1 | 0 |
| Tpp2 | 3.74E-05 | -0.03494 | 0.163 | 0.261 | 1 | 0 |
| Usp19 | 3.75E-05 | -0.06131 | 0.129 | 0.214 | 1 | 0 |
| Lats1 | 3.76E-05 | -0.07471 | 0.051 | 0.104 | 1 | 0 |
| Arpc1b | 3.78E-05 | -0.11537 | 0.53 | 0.68 | 1 | 0 |
| Arhgap17 | 3.80E-05 | -0.08588 | 0.233 | 0.351 | 1 | 0 |
| Pold1 | 3.81E-05 | -0.04718 | 0.071 | 0.132 | 1 | 0 |
| Pxk | 3.82E-05 | -0.13479 | 0.281 | 0.403 | 1 | 0 |
| Arfgap2 | 3.86E-05 | -0.0821 | 0.11 | 0.185 | 1 | 0 |
| Ranbp1 | 3.90E-05 | -0.12929 | 0.21 | 0.315 | 1 | 0 |
| Snx6 | 3.90E-05 | -0.0406 | 0.105 | 0.179 | 1 | 0 |
| Ap2m1 | 3.92E-05 | -0.05356 | 0.138 | 0.228 | 1 | 0 |
| Pabpc1 | 3.94E-05 | -0.1243 | 0.659 | 0.815 | 1 | 0 |
| Utp14a | 3.94E-05 | -0.06904 | 0.09 | 0.158 | 1 | 0 |
| Atp1a1 | 3.98E-05 | -0.03374 | 0.15 | 0.243 | 1 | 0 |
| Spsb3 | 3.98E-05 | -0.06842 | 0.05 | 0.101 | 1 | 0 |
| Fopnl | 4.00E-05 | -0.06759 | 0.051 | 0.104 | 1 | 0 |
| Sfxn1 | 4.01E-05 | -0.06915 | 0.054 | 0.107 | 1 | 0 |
| Eif1 | 4.01E-05 | 0.095759 | 0.982 | 0.992 | 1 | 0 |
| Sec61a1 | 4.04E-05 | -0.04373 | 0.12 | 0.202 | 1 | 0 |
| Scaf8 | 4.05E-05 | -0.08058 | 0.079 | 0.142 | 1 | 0 |
| Selenos | 4.07E-05 | -0.09318 | 0.196 | 0.295 | 1 | 0 |
| Eci1 | 4.09E-05 | -0.06362 | 0.065 | 0.123 | 1 | 0 |
| Snrpa | 4.12E-05 | -0.05815 | 0.079 | 0.142 | 1 | 0 |
| Pou2f1 | 4.12E-05 | -0.0738 | 0.121 | 0.2 | 1 | 0 |
| Stat4 | 4.13E-05 | -0.11231 | 0.278 | 0.396 | 1 | 0 |
| Usp8 | 4.13E-05 | -0.06599 | 0.074 | 0.136 | 1 | 0 |
| Zfp740 | 4.16E-05 | -0.05322 | 0.069 | 0.13 | 1 | 0 |
| Isg20l2 | 4.16E-05 | -0.08324 | 0.055 | 0.109 | 1 | 0 |
| 4932438A13Rik | 4.18E-05 | -0.08705 | 0.237 | 0.347 | 1 | 0 |
| Nhp2 | 4.19E-05 | -0.05322 | 0.1 | 0.172 | 1 | 0 |
| Bccip | 4.19E-05 | -0.06262 | 0.116 | 0.195 | 1 | 0 |
| Supt5 | 4.20E-05 | -0.05732 | 0.162 | 0.258 | 1 | 0 |
| Aurkaip1 | 4.22E-05 | -0.02796 | 0.152 | 0.248 | 1 | 0 |
| Cd68 | 4.22E-05 | 0.232767 | 0.114 | 0.063 | 1 | 0 |
| Ifnar2 | 4.36E-05 | -0.06024 | 0.061 | 0.117 | 1 | 0 |
| Nr1h2 | 4.36E-05 | -0.02902 | 0.096 | 0.168 | 1 | 0 |
| Rnaset2b | 4.41E-05 | -0.0579 | 0.074 | 0.136 | 1 | 0 |
| Snapc5 | 4.43E-05 | -0.0546 | 0.069 | 0.129 | 1 | 0 |
| Slc28a2 | 4.45E-05 | -0.06637 | 0.06 | 0.116 | 1 | 0 |
| Atp5f1 | 4.49E-05 | -0.09635 | 0.355 | 0.502 | 1 | 0 |
| Cenpa | 4.50E-05 | -0.09353 | 0.212 | 0.32 | 1 | 0 |
| Rock2 | 4.52E-05 | -0.06798 | 0.22 | 0.335 | 1 | 0 |
| Trim12c | 4.55E-05 | -0.07113 | 0.101 | 0.173 | 1 | 0 |
| Nudt3 | 4.56E-05 | -0.04305 | 0.063 | 0.121 | 1 | 0 |
| Herpud1 | 4.57E-05 | -0.11775 | 0.196 | 0.297 | 1 | 0 |
| Fkbp8 | 4.57E-05 | -0.03653 | 0.165 | 0.263 | 1 | 0 |
| Calu | 4.59E-05 | -0.04515 | 0.05 | 0.101 | 1 | 0 |
| Ap2s1 | 4.61E-05 | -0.07329 | 0.136 | 0.22 | 1 | 0 |
| Zcchc9 | 4.65E-05 | -0.07325 | 0.084 | 0.149 | 1 | 0 |
| Tank | 4.68E-05 | -0.03542 | 0.124 | 0.208 | 1 | 0 |
| Kpnb1 | 4.70E-05 | -0.0264 | 0.11 | 0.189 | 1 | 0 |
| Ndufa3 | 4.72E-05 | -0.08805 | 0.268 | 0.395 | 1 | 0 |
| Rlim | 4.75E-05 | -0.08869 | 0.192 | 0.294 | 1 | 0 |
| Ap3b1 | 4.82E-05 | -0.05357 | 0.071 | 0.132 | 1 | 0 |
| Manf | 4.86E-05 | -0.21982 | 0.247 | 0.359 | 1 | 0 |
| Slain2 | 4.92E-05 | -0.05296 | 0.078 | 0.141 | 1 | 0 |
| Usp14 | 4.92E-05 | -0.05185 | 0.075 | 0.137 | 1 | 0 |
| Ubl5 | 4.99E-05 | 0.217538 | 0.568 | 0.564 | 1 | 0 |
| Plec | 5.00E-05 | -0.06523 | 0.076 | 0.137 | 1 | 0 |
| Usp12 | 5.01E-05 | -0.0564 | 0.053 | 0.106 | 1 | 0 |
| Epb41 | 5.02E-05 | -0.06752 | 0.15 | 0.239 | 1 | 0 |
| Ndufa9 | 5.05E-05 | -0.05956 | 0.07 | 0.13 | 1 | 0 |
| Ythdc2 | 5.06E-05 | -0.07116 | 0.061 | 0.117 | 1 | 0 |
| Hadha | 5.07E-05 | -0.08062 | 0.148 | 0.233 | 1 | 0 |
| Cers4 | 5.08E-05 | -0.07963 | 0.092 | 0.159 | 1 | 0 |
| Cul1 | 5.09E-05 | -0.0689 | 0.132 | 0.214 | 1 | 0 |
| Tcp1 | 5.09E-05 | -0.05844 | 0.25 | 0.375 | 1 | 0 |
| Vamp4 | 5.09E-05 | -0.05114 | 0.079 | 0.142 | 1 | 0 |
| Ndufa11 | 5.12E-05 | -0.08601 | 0.185 | 0.28 | 1 | 0 |
| Mprip | 5.13E-05 | -0.08033 | 0.09 | 0.158 | 1 | 0 |
| Rabgap1 | 5.22E-05 | -0.07154 | 0.064 | 0.121 | 1 | 0 |
| Ppp2r2a | 5.22E-05 | -0.07623 | 0.2 | 0.308 | 1 | 0 |
| Faim | 5.23E-05 | -0.05569 | 0.066 | 0.125 | 1 | 0 |
| Dhps | 5.24E-05 | -0.06779 | 0.101 | 0.172 | 1 | 0 |
| Drg1 | 5.34E-05 | -0.03095 | 0.051 | 0.104 | 1 | 0 |
| 0610030E20Rik | 5.38E-05 | -0.08928 | 0.096 | 0.166 | 1 | 0 |
| Sdhaf2 | 5.40E-05 | -0.05944 | 0.058 | 0.112 | 1 | 0 |
| Spopl | 5.43E-05 | -0.04488 | 0.077 | 0.14 | 1 | 0 |
| Cwf19l2 | 5.47E-05 | -0.0412 | 0.106 | 0.178 | 1 | 0 |
| Ndufs5 | 5.49E-05 | -0.02707 | 0.17 | 0.268 | 1 | 0 |
| Cdk4 | 5.50E-05 | -0.07303 | 0.072 | 0.131 | 1 | 0 |
| Gpr132 | 5.50E-05 | -0.08992 | 0.198 | 0.297 | 1 | 0 |
| Stx16 | 5.55E-05 | -0.06562 | 0.09 | 0.156 | 1 | 0 |
| Ikzf1 | 5.56E-05 | -0.05402 | 0.246 | 0.37 | 1 | 0 |
| Mta2 | 5.57E-05 | -0.03333 | 0.109 | 0.185 | 1 | 0 |
| Nr4a1 | 5.57E-05 | 0.292504 | 0.499 | 0.474 | 1 | 0 |
| Bcl2 | 5.59E-05 | -0.10174 | 0.183 | 0.274 | 1 | 0 |
| Cdt1 | 5.61E-05 | -0.07586 | 0.128 | 0.208 | 1 | 0 |
| Cyb5b | 5.63E-05 | -0.03883 | 0.076 | 0.138 | 1 | 0 |
| Cd81 | 5.65E-05 | -0.03669 | 0.279 | 0.419 | 1 | 0 |
| Sarnp | 5.66E-05 | -0.0678 | 0.184 | 0.285 | 1 | 0 |
| Trappc2l | 5.67E-05 | -0.03867 | 0.105 | 0.178 | 1 | 0 |
| Slfn2 | 5.67E-05 | -0.18811 | 0.395 | 0.512 | 1 | 0 |
| Lyn | 5.68E-05 | -0.09051 | 0.388 | 0.548 | 1 | 0 |
| Slc25a11 | 5.71E-05 | -0.08584 | 0.086 | 0.149 | 1 | 0 |
| Sacs | 5.76E-05 | -0.0537 | 0.057 | 0.111 | 1 | 0 |
| 1110038F14Rik | 5.79E-05 | -0.04758 | 0.062 | 0.118 | 1 | 0 |
| Chst3 | 5.81E-05 | -0.07708 | 0.096 | 0.165 | 1 | 0 |
| Zfp638 | 5.82E-05 | -0.04369 | 0.182 | 0.285 | 1 | 0 |
| Eif3g | 5.84E-05 | -0.06295 | 0.208 | 0.317 | 1 | 0 |
| Dmac1 | 5.84E-05 | -0.0515 | 0.095 | 0.165 | 1 | 0 |
| Dap3 | 5.97E-05 | -0.07403 | 0.089 | 0.154 | 1 | 0 |
| Zfand3 | 5.99E-05 | -0.04453 | 0.069 | 0.129 | 1 | 0 |
| Mrpl40 | 6.03E-05 | -0.01832 | 0.051 | 0.104 | 1 | 0 |
| Eif4enif1 | 6.08E-05 | -0.06097 | 0.061 | 0.116 | 1 | 0 |
| Mtmr3 | 6.17E-05 | -0.04363 | 0.105 | 0.18 | 1 | 0 |
| Rabep2 | 6.17E-05 | -0.07294 | 0.107 | 0.179 | 1 | 0 |
| Commd2 | 6.20E-05 | -0.05557 | 0.053 | 0.105 | 1 | 0 |
| Atr | 6.21E-05 | -0.08425 | 0.055 | 0.107 | 1 | 0 |
| Immt | 6.25E-05 | -0.06806 | 0.138 | 0.22 | 1 | 0 |
| Eif4e2 | 6.29E-05 | -0.04227 | 0.156 | 0.251 | 1 | 0 |
| Dgka | 6.31E-05 | -0.06713 | 0.102 | 0.174 | 1 | 0 |
| Snrpd3 | 6.34E-05 | -0.08889 | 0.206 | 0.31 | 1 | 0 |
| Brd7 | 6.37E-05 | -0.08318 | 0.108 | 0.179 | 1 | 0 |
| Anp32b | 6.39E-05 | -0.13727 | 0.349 | 0.481 | 1 | 0 |
| Hdac7 | 6.56E-05 | -0.05044 | 0.072 | 0.132 | 1 | 0 |
| Sdha | 6.59E-05 | -0.03014 | 0.173 | 0.271 | 1 | 0 |
| Polr2f | 6.69E-05 | -0.07648 | 0.15 | 0.234 | 1 | 0 |
| Brcc3 | 6.73E-05 | -0.05674 | 0.054 | 0.106 | 1 | 0 |
| Pds5b | 6.76E-05 | -0.07109 | 0.076 | 0.136 | 1 | 0 |
| Pgp | 6.86E-05 | -0.04112 | 0.08 | 0.142 | 1 | 0 |
| Etfb | 6.91E-05 | -0.02227 | 0.099 | 0.171 | 1 | 0 |
| Psma2 | 6.93E-05 | -0.1045 | 0.301 | 0.436 | 1 | 0 |
| G3bp2 | 6.98E-05 | -0.05427 | 0.209 | 0.317 | 1 | 0 |
| Dck | 6.98E-05 | -0.07716 | 0.07 | 0.128 | 1 | 0 |
| Add1 | 6.99E-05 | -0.07563 | 0.135 | 0.215 | 1 | 0 |
| Polr2g | 7.00E-05 | -0.06178 | 0.11 | 0.184 | 1 | 0 |
| Zcchc7 | 7.12E-05 | -0.11125 | 0.343 | 0.486 | 1 | 0 |
| Hnrnpr | 7.12E-05 | -0.05277 | 0.183 | 0.285 | 1 | 0 |
| Mrpl15 | 7.20E-05 | -0.04716 | 0.105 | 0.178 | 1 | 0 |
| Tcf4 | 7.23E-05 | -0.10754 | 0.315 | 0.445 | 1 | 0 |
| Xiap | 7.25E-05 | -0.05299 | 0.16 | 0.249 | 1 | 0 |
| Arhgef3 | 7.27E-05 | -0.06888 | 0.096 | 0.165 | 1 | 0 |
| Zfp91 | 7.28E-05 | -0.0547 | 0.234 | 0.35 | 1 | 0 |
| Secisbp2l | 7.29E-05 | -0.06982 | 0.066 | 0.123 | 1 | 0 |
| Icam2 | 7.29E-05 | -0.07773 | 0.077 | 0.137 | 1 | 0 |
| Samm50 | 7.30E-05 | -0.06198 | 0.087 | 0.152 | 1 | 0 |
| Uggt1 | 7.33E-05 | -0.05993 | 0.077 | 0.137 | 1 | 0 |
| Hivep2 | 7.33E-05 | -0.12068 | 0.097 | 0.162 | 1 | 0 |
| Pfkp | 7.38E-05 | -0.04258 | 0.072 | 0.131 | 1 | 0 |
| Gm26917 | 7.47E-05 | -0.1122 | 0.309 | 0.446 | 1 | 0 |
| Ccr6 | 7.51E-05 | -0.10347 | 0.076 | 0.134 | 1 | 0 |
| Txndc11 | 7.60E-05 | -0.05525 | 0.109 | 0.183 | 1 | 0 |
| Pcm1 | 7.68E-05 | -0.07016 | 0.137 | 0.217 | 1 | 0 |
| Dazap1 | 7.85E-05 | -0.0389 | 0.123 | 0.203 | 1 | 0 |
| Ruvbl1 | 7.90E-05 | -0.03168 | 0.082 | 0.146 | 1 | 0 |
| Cd72 | 7.90E-05 | -0.14401 | 0.228 | 0.325 | 1 | 0 |
| Mxd4 | 7.91E-05 | -0.08917 | 0.134 | 0.211 | 1 | 0 |
| Txlng | 7.92E-05 | -0.07932 | 0.054 | 0.105 | 1 | 0 |
| Cnot1 | 7.94E-05 | -0.04218 | 0.186 | 0.285 | 1 | 0 |
| Mrpl9 | 7.96E-05 | -0.04393 | 0.067 | 0.125 | 1 | 0 |
| Rab1b | 8.00E-05 | -0.04285 | 0.089 | 0.154 | 1 | 0 |
| Trappc5 | 8.05E-05 | -0.04678 | 0.107 | 0.178 | 1 | 0 |
| Tnip1 | 8.11E-05 | -0.04002 | 0.069 | 0.128 | 1 | 0 |
| Strn4 | 8.12E-05 | -0.04726 | 0.055 | 0.107 | 1 | 0 |
| Ndufv1 | 8.13E-05 | -0.0519 | 0.087 | 0.152 | 1 | 0 |
| Preb | 8.20E-05 | -0.06391 | 0.052 | 0.103 | 1 | 0 |
| Blnk | 8.21E-05 | -0.08273 | 0.298 | 0.436 | 1 | 0 |
| Polr2b | 8.21E-05 | -0.0461 | 0.075 | 0.135 | 1 | 0 |
| Mak16 | 8.23E-05 | -0.02616 | 0.136 | 0.221 | 1 | 0 |
| Csnk1d | 8.25E-05 | -0.05021 | 0.104 | 0.177 | 1 | 0 |
| Rab10 | 8.35E-05 | -0.04294 | 0.212 | 0.321 | 1 | 0 |
| Erp44 | 8.37E-05 | -0.05891 | 0.144 | 0.229 | 1 | 0 |
| Alox5ap | 8.38E-05 | 0.250442 | 0.137 | 0.085 | 1 | 0 |
| Srsf2 | 8.48E-05 | 0.288777 | 0.528 | 0.557 | 1 | 0 |
| Zfr | 8.52E-05 | -0.0485 | 0.111 | 0.185 | 1 | 0 |
| Bach1 | 8.54E-05 | -0.04282 | 0.065 | 0.121 | 1 | 0 |
| Ewsr1 | 8.58E-05 | -0.05756 | 0.203 | 0.308 | 1 | 0 |
| Rab3gap1 | 8.60E-05 | -0.07793 | 0.117 | 0.191 | 1 | 0 |
| Ifi27 | 8.67E-05 | -0.06272 | 0.101 | 0.171 | 1 | 0 |
| Chil3 | 8.70E-05 | 0.37952 | 0.414 | 0.393 | 1 | 0 |
| Mob2 | 8.73E-05 | -0.06832 | 0.059 | 0.112 | 1 | 0 |
| Acp1 | 8.78E-05 | -0.00628 | 0.099 | 0.171 | 1 | 0 |
| Nmd3 | 8.78E-05 | -0.02589 | 0.075 | 0.136 | 1 | 0 |
| Sec24a | 8.81E-05 | -0.04729 | 0.078 | 0.14 | 1 | 0 |
| Lbh | 8.82E-05 | -0.05572 | 0.233 | 0.352 | 1 | 0 |
| Rin3 | 8.84E-05 | -0.08031 | 0.07 | 0.126 | 1 | 0 |
| Dock11 | 8.87E-05 | -0.07725 | 0.17 | 0.264 | 1 | 0 |
| Ube4a | 8.88E-05 | -0.05001 | 0.051 | 0.101 | 1 | 0 |
| Fam214a | 8.97E-05 | -0.05362 | 0.096 | 0.165 | 1 | 0 |
| Prrc2a | 8.98E-05 | -0.05719 | 0.215 | 0.325 | 1 | 0 |
| Psma7 | 8.98E-05 | -0.07935 | 0.328 | 0.473 | 1 | 0 |
| Snx27 | 9.00E-05 | -0.0658 | 0.053 | 0.104 | 1 | 0 |
| Zranb1 | 9.06E-05 | -0.03764 | 0.135 | 0.217 | 1 | 0 |
| Ascc3 | 9.08E-05 | -0.03235 | 0.126 | 0.205 | 1 | 0 |
| Khdrbs1 | 9.11E-05 | -0.02016 | 0.224 | 0.341 | 1 | 0 |
| Ythdf3 | 9.11E-05 | -0.04726 | 0.091 | 0.156 | 1 | 0 |
| Ak2 | 9.14E-05 | -0.05318 | 0.129 | 0.205 | 1 | 0 |
| Gak | 9.17E-05 | -0.05747 | 0.071 | 0.13 | 1 | 0 |
| Bud23 | 9.25E-05 | -0.07385 | 0.057 | 0.109 | 1 | 0 |
| Ppp3cb | 9.31E-05 | -0.05172 | 0.11 | 0.184 | 1 | 0 |
| Actl6a | 9.35E-05 | -0.06495 | 0.107 | 0.177 | 1 | 0 |
| Cnot2 | 9.40E-05 | -0.02459 | 0.113 | 0.187 | 1 | 0 |
| Gpr65 | 9.40E-05 | -0.07706 | 0.067 | 0.123 | 1 | 0 |
| Def6 | 9.41E-05 | -0.0383 | 0.059 | 0.112 | 1 | 0 |
| Zmat5 | 9.42E-05 | -0.05211 | 0.061 | 0.115 | 1 | 0 |
| Rplp1 | 9.42E-05 | 0.065792 | 0.999 | 1 | 1 | 0 |
| Eps15l1 | 9.42E-05 | -0.05725 | 0.052 | 0.103 | 1 | 0 |
| Pbdc1 | 9.46E-05 | -0.05318 | 0.094 | 0.161 | 1 | 0 |
| Tlk1 | 9.53E-05 | -0.03351 | 0.146 | 0.23 | 1 | 0 |
| Mfsd4a | 9.58E-05 | -0.08644 | 0.094 | 0.16 | 1 | 0 |
| Srp14 | 9.63E-05 | -0.07249 | 0.296 | 0.438 | 1 | 0 |
| 9930021J03Rik | 9.69E-05 | -0.08366 | 0.08 | 0.138 | 1 | 0 |
| Fnbp1 | 9.71E-05 | -0.04784 | 0.157 | 0.246 | 1 | 0 |
| Ylpm1 | 9.74E-05 | -0.04851 | 0.085 | 0.148 | 1 | 0 |
| Ndufb10 | 9.78E-05 | -0.08891 | 0.274 | 0.401 | 1 | 0 |
| Klf7 | 9.80E-05 | -0.06302 | 0.097 | 0.163 | 1 | 0 |
| Srsf6 | 9.89E-05 | -0.08102 | 0.246 | 0.36 | 1 | 0 |
| Noc2l | 9.91E-05 | -0.07859 | 0.078 | 0.137 | 1 | 0 |
| Zbtb44 | 9.92E-05 | -0.04103 | 0.092 | 0.159 | 1 | 0 |
| Plekho1 | 9.93E-05 | -0.09042 | 0.182 | 0.274 | 1 | 0 |
| Ap1ar | 9.94E-05 | -0.04123 | 0.051 | 0.101 | 1 | 0 |
| Zc3h14 | 9.95E-05 | -0.04294 | 0.083 | 0.147 | 1 | 0 |
| Rnase6 | 1.00E-04 | -0.05991 | 0.142 | 0.223 | 1 | 0 |
| B230219D22Rik | 0.00010119 | -0.06328 | 0.135 | 0.214 | 1 | 0 |
| Zdhhc5 | 0.000101379 | -0.03895 | 0.058 | 0.111 | 1 | 0 |
| Rexo2 | 0.0001015 | -0.10879 | 0.119 | 0.191 | 1 | 0 |
| Uso1 | 0.00010199 | -0.05679 | 0.081 | 0.142 | 1 | 0 |
| Sp3 | 0.00010324 | -0.07591 | 0.135 | 0.214 | 1 | 0 |
| Ripk1 | 0.000103807 | -0.05313 | 0.08 | 0.142 | 1 | 0 |
| Irf9 | 0.000104428 | -0.07606 | 0.109 | 0.179 | 1 | 0 |
| Larp1b | 0.000107119 | -0.02347 | 0.07 | 0.128 | 1 | 0 |
| Ptp4a2 | 0.000107701 | -0.10091 | 0.492 | 0.649 | 1 | 0 |
| Traf1 | 0.000108887 | -0.01779 | 0.113 | 0.185 | 1 | 0 |
| Rap2c | 0.000110243 | -0.07315 | 0.091 | 0.154 | 1 | 0 |
| Lpp | 0.000110517 | -0.06434 | 0.12 | 0.193 | 1 | 0 |
| Cbl | 0.00011095 | -0.0565 | 0.201 | 0.304 | 1 | 0 |
| Dgkd | 0.000111126 | -0.07594 | 0.297 | 0.425 | 1 | 0 |
| Phax | 0.000111384 | -0.06228 | 0.063 | 0.116 | 1 | 0 |
| Commd7 | 0.00011172 | -0.03519 | 0.089 | 0.154 | 1 | 0 |
| Fermt3 | 0.00011364 | -0.03052 | 0.176 | 0.271 | 1 | 0 |
| Kif21b | 0.000113725 | -0.0306 | 0.143 | 0.229 | 1 | 0 |
| Ndufs1 | 0.000114224 | -0.06152 | 0.059 | 0.111 | 1 | 0 |
| Cib1 | 0.000114375 | -0.04639 | 0.108 | 0.178 | 1 | 0 |
| Synrg | 0.000114421 | -0.05643 | 0.098 | 0.166 | 1 | 0 |
| Unc119 | 0.00011538 | -0.06487 | 0.082 | 0.142 | 1 | 0 |
| Ccdc174 | 0.000116287 | -0.04524 | 0.102 | 0.169 | 1 | 0 |
| Prpf38a | 0.000116879 | -0.05356 | 0.088 | 0.152 | 1 | 0 |
| Rb1cc1 | 0.00011715 | -0.10823 | 0.251 | 0.363 | 1 | 0 |
| Cd55 | 0.000117822 | -0.16858 | 0.493 | 0.609 | 1 | 0 |
| Elk4 | 0.000119975 | -0.02936 | 0.19 | 0.291 | 1 | 0 |
| Mkln1 | 0.000120795 | -0.08027 | 0.196 | 0.295 | 1 | 0 |
| Taok2 | 0.00012123 | -0.05466 | 0.066 | 0.122 | 1 | 0 |
| Snap23 | 0.000121437 | -0.06875 | 0.088 | 0.15 | 1 | 0 |
| Atp8a1 | 0.000121944 | -0.07106 | 0.104 | 0.172 | 1 | 0 |
| Zfand5 | 0.000122016 | -0.03374 | 0.239 | 0.357 | 1 | 0 |
| Arl1 | 0.000122314 | -0.05872 | 0.099 | 0.166 | 1 | 0 |
| Fxyd5 | 0.000123038 | 0.29664 | 0.355 | 0.31 | 1 | 0 |
| Mrps18c | 0.000124455 | -0.01768 | 0.102 | 0.172 | 1 | 0 |
| A630001G21Rik | 0.000124464 | -0.04527 | 0.199 | 0.301 | 1 | 0 |
| Sp110 | 0.000126246 | -0.05468 | 0.216 | 0.322 | 1 | 0 |
| Gatad2a | 0.000126883 | -0.00372 | 0.11 | 0.184 | 1 | 0 |
| Ubap2 | 0.000126997 | -0.02765 | 0.065 | 0.119 | 1 | 0 |
| Kdm6a | 0.000127152 | -0.05411 | 0.065 | 0.118 | 1 | 0 |
| Prpsap1 | 0.00012722 | -0.0689 | 0.07 | 0.125 | 1 | 0 |
| Trim56 | 0.000128715 | -0.0663 | 0.065 | 0.119 | 1 | 0 |
| Ube2v2 | 0.000129537 | -0.03091 | 0.117 | 0.192 | 1 | 0 |
| Hspa8 | 0.000129944 | 0.137723 | 0.957 | 0.959 | 1 | 0 |
| Cers5 | 0.000130088 | -0.03639 | 0.059 | 0.111 | 1 | 0 |
| Lman2 | 0.000131695 | -0.04718 | 0.161 | 0.251 | 1 | 0 |
| Man2a1 | 0.000133953 | -0.03632 | 0.133 | 0.211 | 1 | 0 |
| Rap1a | 0.000134014 | -0.09059 | 0.303 | 0.433 | 1 | 0 |
| Nfe2l1 | 0.000134176 | -0.03908 | 0.084 | 0.147 | 1 | 0 |
| Psmc2 | 0.000134275 | -0.03671 | 0.105 | 0.174 | 1 | 0 |
| Snrpb | 0.000134489 | -0.06215 | 0.353 | 0.501 | 1 | 0 |
| Prpf4 | 0.000135875 | -0.04071 | 0.052 | 0.101 | 1 | 0 |
| Btbd7 | 0.000136738 | -0.0711 | 0.133 | 0.209 | 1 | 0 |
| Trim28 | 0.000137336 | -0.03301 | 0.094 | 0.161 | 1 | 0 |
| Nkap | 0.000138106 | -0.03455 | 0.069 | 0.125 | 1 | 0 |
| Dhx15 | 0.000138917 | -0.03696 | 0.196 | 0.3 | 1 | 0 |
| Akr1b3 | 0.000139703 | -0.06125 | 0.167 | 0.258 | 1 | 0 |
| Cisd1 | 0.000140692 | -0.04627 | 0.066 | 0.121 | 1 | 0 |
| Ptpra | 0.000141297 | -0.06205 | 0.094 | 0.158 | 1 | 0 |
| Stim1 | 0.000141327 | -0.04549 | 0.126 | 0.203 | 1 | 0 |
| A530040E14Rik | 0.000141573 | -0.02697 | 0.099 | 0.168 | 1 | 0 |
| Necap2 | 0.000141602 | -0.06557 | 0.097 | 0.162 | 1 | 0 |
| C1galt1 | 0.000141983 | -0.08616 | 0.136 | 0.212 | 1 | 0 |
| Arid3b | 0.000144612 | -0.02143 | 0.068 | 0.124 | 1 | 0 |
| Psmd6 | 0.000144635 | -0.02257 | 0.098 | 0.166 | 1 | 0 |
| Igkc | 0.000146904 | -0.53579 | 0.966 | 0.955 | 1 | 0 |
| Hmgb2 | 0.000148279 | -0.12857 | 0.434 | 0.581 | 1 | 0 |
| Prr5 | 0.000148412 | -0.04541 | 0.058 | 0.109 | 1 | 0 |
| Cited2 | 0.000148588 | -0.02874 | 0.127 | 0.204 | 1 | 0 |
| Tmbim6 | 0.000148804 | -0.08427 | 0.363 | 0.511 | 1 | 0 |
| Dpm2 | 0.000149281 | -0.06282 | 0.06 | 0.111 | 1 | 0 |
| Utp18 | 0.000151076 | -0.03531 | 0.079 | 0.138 | 1 | 0 |
| Smchd1 | 0.000151845 | -0.08089 | 0.365 | 0.494 | 1 | 0 |
| Xpo1 | 0.00015205 | -0.06941 | 0.082 | 0.142 | 1 | 0 |
| Mob1a | 0.000152051 | -0.02848 | 0.211 | 0.321 | 1 | 0 |
| Gps2 | 0.000152333 | -0.06441 | 0.122 | 0.195 | 1 | 0 |
| Ikzf5 | 0.000152477 | -0.048 | 0.065 | 0.118 | 1 | 0 |
| Smarca2 | 0.00015295 | -0.04811 | 0.105 | 0.172 | 1 | 0 |
| Ctdsp1 | 0.000155916 | -0.05131 | 0.077 | 0.135 | 1 | 0 |
| Tbc1d20 | 0.000156567 | -0.05943 | 0.109 | 0.179 | 1 | 0 |
| Lin7c | 0.000157135 | -0.04025 | 0.095 | 0.161 | 1 | 0 |
| Sp140 | 0.000157146 | -0.04829 | 0.198 | 0.3 | 1 | 0 |
| Rsrc2 | 0.000157436 | -0.11107 | 0.484 | 0.641 | 1 | 0 |
| Cyhr1 | 0.000158223 | -0.04245 | 0.079 | 0.137 | 1 | 0 |
| Csnk1g2 | 0.00015885 | -0.06382 | 0.136 | 0.214 | 1 | 0 |
| 5031439G07Rik | 0.000159471 | -0.05715 | 0.073 | 0.129 | 1 | 0 |
| Smarcad1 | 0.000159875 | -0.04362 | 0.075 | 0.132 | 1 | 0 |
| Ncaph2 | 0.000160251 | -0.05039 | 0.056 | 0.106 | 1 | 0 |
| Mpp6 | 0.00016356 | -0.03029 | 0.067 | 0.122 | 1 | 0 |
| Ccdc25 | 0.000164044 | -0.07635 | 0.067 | 0.121 | 1 | 0 |
| Zfp592 | 0.000164054 | -0.08236 | 0.08 | 0.138 | 1 | 0 |
| Usp3 | 0.000164644 | -0.06805 | 0.11 | 0.179 | 1 | 0 |
| Tax1bp1 | 0.000166329 | -0.07822 | 0.444 | 0.605 | 1 | 0 |
| AC149090.1 | 0.000167099 | -0.08726 | 0.169 | 0.255 | 1 | 0 |
| Smg5 | 0.000168107 | -0.04215 | 0.076 | 0.134 | 1 | 0 |
| Kdelr1 | 0.000169501 | -0.03433 | 0.107 | 0.175 | 1 | 0 |
| Kdm5c | 0.000169636 | -0.03374 | 0.066 | 0.121 | 1 | 0 |
| Ist1 | 0.000169693 | -0.08187 | 0.074 | 0.13 | 1 | 0 |
| Chst15 | 0.000170268 | -0.06232 | 0.073 | 0.129 | 1 | 0 |
| Crbn | 0.000170439 | -0.0306 | 0.081 | 0.142 | 1 | 0 |
| Ttc3 | 0.000170585 | -0.04406 | 0.091 | 0.154 | 1 | 0 |
| Atad2 | 0.00017106 | -0.0722 | 0.102 | 0.167 | 1 | 0 |
| Scand1 | 0.000171725 | -0.07727 | 0.348 | 0.494 | 1 | 0 |
| Cuta | 0.00017192 | -0.05553 | 0.094 | 0.158 | 1 | 0 |
| Ccm2 | 0.000173 | -0.05405 | 0.178 | 0.271 | 1 | 0 |
| Ablim1 | 0.00017426 | -0.08021 | 0.357 | 0.493 | 1 | 0 |
| H2afv | 0.00017541 | -0.03183 | 0.228 | 0.346 | 1 | 0 |
| Fto | 0.000175837 | -0.07505 | 0.081 | 0.138 | 1 | 0 |
| Cct2 | 0.000176457 | -0.0888 | 0.336 | 0.486 | 1 | 0 |
| Arap2 | 0.000176501 | -0.08066 | 0.328 | 0.447 | 1 | 0 |
| Prpf19 | 0.000178256 | -0.06522 | 0.065 | 0.117 | 1 | 0 |
| Ddost | 0.000178526 | -0.05386 | 0.113 | 0.184 | 1 | 0 |
| Dars | 0.000179558 | -0.04359 | 0.059 | 0.11 | 1 | 0 |
| Chd6 | 0.000179582 | -0.06997 | 0.196 | 0.288 | 1 | 0 |
| Ate1 | 0.00017967 | -0.0547 | 0.084 | 0.144 | 1 | 0 |
| Map4k4 | 0.000180314 | -0.01732 | 0.18 | 0.277 | 1 | 0 |
| Nin | 0.000181791 | -0.04465 | 0.093 | 0.156 | 1 | 0 |
| Smarcc2 | 0.000182696 | -0.03417 | 0.094 | 0.159 | 1 | 0 |
| Ndufb9 | 0.000182957 | -0.02056 | 0.186 | 0.285 | 1 | 0 |
| Pfdn2 | 0.000184605 | -0.03732 | 0.224 | 0.338 | 1 | 0 |
| Nemf | 0.000186757 | -0.01037 | 0.158 | 0.248 | 1 | 0 |
| Rab22a | 0.000189482 | -0.03635 | 0.083 | 0.143 | 1 | 0 |
| Supt6 | 0.00019045 | -0.04979 | 0.094 | 0.158 | 1 | 0 |
| Pnpla8 | 0.000193516 | -0.04149 | 0.167 | 0.258 | 1 | 0 |
| Ctr9 | 0.00019359 | -0.01589 | 0.053 | 0.103 | 1 | 0 |
| Ppp1r18 | 0.000194096 | -0.02813 | 0.158 | 0.247 | 1 | 0 |
| Bicra | 0.000194207 | -0.06045 | 0.055 | 0.104 | 1 | 0 |
| Med21 | 0.00019555 | -0.04377 | 0.082 | 0.142 | 1 | 0 |
| Nvl | 0.000195662 | -0.04622 | 0.063 | 0.115 | 1 | 0 |
| Mbd3 | 0.000196352 | -0.01103 | 0.108 | 0.177 | 1 | 0 |
| Smagp | 0.00019689 | -0.09334 | 0.068 | 0.121 | 1 | 0 |
| Eif1a | 0.000197926 | -0.03086 | 0.123 | 0.197 | 1 | 0 |
| Son | 0.000199775 | -0.1217 | 0.708 | 0.833 | 1 | 0 |
| Tial1 | 0.000200214 | -0.03028 | 0.118 | 0.191 | 1 | 0 |
| Lonp2 | 0.000200418 | -0.07765 | 0.064 | 0.115 | 1 | 0 |
| Cr2 | 0.000200693 | -0.0883 | 0.164 | 0.245 | 1 | 0 |
| Dicer1 | 0.000200866 | -0.05239 | 0.123 | 0.197 | 1 | 0 |
| Cnot7 | 0.00020093 | -0.0313 | 0.08 | 0.14 | 1 | 0 |
| Trnau1ap | 0.000201374 | -0.07471 | 0.061 | 0.111 | 1 | 0 |
| Adipor2 | 0.000201934 | -0.04854 | 0.105 | 0.172 | 1 | 0 |
| Stxbp2 | 0.000202546 | -0.02609 | 0.072 | 0.128 | 1 | 0 |
| Ergic3 | 0.000202914 | -0.0341 | 0.093 | 0.156 | 1 | 0 |
| Fmnl1 | 0.000203235 | -0.03061 | 0.138 | 0.22 | 1 | 0 |
| Atic | 0.000204635 | -0.00857 | 0.061 | 0.113 | 1 | 0 |
| Cetn3 | 0.000205185 | -0.04644 | 0.168 | 0.258 | 1 | 0 |
| Chp1 | 0.000205549 | -0.03395 | 0.07 | 0.125 | 1 | 0 |
| Tbc1d15 | 0.000205769 | -0.06506 | 0.132 | 0.208 | 1 | 0 |
| Ing3 | 0.000206179 | -0.06298 | 0.083 | 0.142 | 1 | 0 |
| Apbb1ip | 0.000207104 | -0.0595 | 0.254 | 0.364 | 1 | 0 |
| Scp2 | 0.000208564 | -0.04036 | 0.225 | 0.333 | 1 | 0 |
| Api5 | 0.000209585 | -0.01854 | 0.101 | 0.169 | 1 | 0 |
| Otud5 | 0.000209809 | -0.01554 | 0.086 | 0.148 | 1 | 0 |
| Naa50 | 0.000210784 | -0.009 | 0.152 | 0.24 | 1 | 0 |
| S100a11 | 0.000212704 | 0.236536 | 0.48 | 0.47 | 1 | 0 |
| Atf7ip | 0.000213927 | -0.05182 | 0.21 | 0.308 | 1 | 0 |
| Ufl1 | 0.000214388 | -0.07326 | 0.057 | 0.105 | 1 | 0 |
| Zmat2 | 0.000215646 | -0.03366 | 0.16 | 0.247 | 1 | 0 |
| Pbxip1 | 0.000215792 | -0.07848 | 0.176 | 0.263 | 1 | 0 |
| Polr2c | 0.000215947 | -0.04718 | 0.098 | 0.163 | 1 | 0 |
| Nup214 | 0.000219918 | -0.05177 | 0.066 | 0.119 | 1 | 0 |
| Trappc1 | 0.000220444 | -0.02731 | 0.055 | 0.104 | 1 | 0 |
| Chtf8 | 0.000220492 | -0.07285 | 0.063 | 0.113 | 1 | 0 |
| Arf4 | 0.000221065 | -0.06065 | 0.29 | 0.412 | 1 | 0 |
| Trappc6a | 0.000222378 | -0.00938 | 0.064 | 0.116 | 1 | 0 |
| Gngt2 | 0.000223802 | -0.02305 | 0.185 | 0.279 | 1 | 0 |
| Cops4 | 0.000225354 | -0.03868 | 0.116 | 0.186 | 1 | 0 |
| Srsf9 | 0.00022631 | -0.04523 | 0.162 | 0.251 | 1 | 0 |
| Tlk2 | 0.000226934 | -0.04703 | 0.077 | 0.134 | 1 | 0 |
| Pex7 | 0.00023206 | -0.04551 | 0.06 | 0.11 | 1 | 0 |
| Emc2 | 0.000233122 | -0.03577 | 0.089 | 0.15 | 1 | 0 |
| Slpi | 0.000233592 | -0.11316 | 0.179 | 0.122 | 1 | 0 |
| Mrps5 | 0.000234819 | -0.0536 | 0.063 | 0.113 | 1 | 0 |
| Csnk1g1 | 0.00023562 | -0.02999 | 0.079 | 0.136 | 1 | 0 |
| Pebp1 | 0.000236165 | -0.02634 | 0.226 | 0.338 | 1 | 0 |
| Zfp68 | 0.00023687 | -0.06488 | 0.071 | 0.125 | 1 | 0 |
| Exoc4 | 0.000237138 | -0.05318 | 0.073 | 0.128 | 1 | 0 |
| Brd3 | 0.000239147 | -0.0277 | 0.097 | 0.162 | 1 | 0 |
| Plekhm3 | 0.000239156 | -0.01765 | 0.102 | 0.168 | 1 | 0 |
| Zhx1 | 0.000239515 | -0.02713 | 0.062 | 0.113 | 1 | 0 |
| Ppp2r5e | 0.000240592 | -0.04808 | 0.072 | 0.126 | 1 | 0 |
| Epc1 | 0.000241345 | -0.05315 | 0.14 | 0.218 | 1 | 0 |
| Snx20 | 0.000242241 | -0.06961 | 0.265 | 0.377 | 1 | 0 |
| Srrm1 | 0.000243142 | -0.05747 | 0.337 | 0.48 | 1 | 0 |
| Pim1 | 0.000243734 | -0.07527 | 0.405 | 0.519 | 1 | 0 |
| Elf1 | 0.000245227 | -0.07674 | 0.451 | 0.617 | 1 | 0 |
| Usp16 | 0.000247016 | -0.02497 | 0.133 | 0.21 | 1 | 0 |
| Uqcc2 | 0.00024883 | -0.01444 | 0.125 | 0.202 | 1 | 0 |
| Wipi2 | 0.000249041 | -0.02283 | 0.102 | 0.168 | 1 | 0 |
| Herc1 | 0.00025004 | -0.0161 | 0.164 | 0.251 | 1 | 0 |
| Skiv2l2 | 0.000250044 | -0.05297 | 0.077 | 0.132 | 1 | 0 |
| Ankrd17 | 0.000250911 | -0.02609 | 0.247 | 0.363 | 1 | 0 |
| Cnot10 | 0.000251111 | -0.05619 | 0.075 | 0.13 | 1 | 0 |
| Hck | 0.000251603 | -0.0606 | 0.069 | 0.122 | 1 | 0 |
| Arglu1 | 0.000252286 | -0.05867 | 0.337 | 0.471 | 1 | 0 |
| Cldnd1 | 0.000252698 | -0.05071 | 0.09 | 0.15 | 1 | 0 |
| Nktr | 0.000253704 | -0.05772 | 0.324 | 0.455 | 1 | 0 |
| Cnih4 | 0.00025385 | -0.01834 | 0.091 | 0.153 | 1 | 0 |
| Mkrn1 | 0.000254417 | -0.01452 | 0.153 | 0.24 | 1 | 0 |
| Snrpd1 | 0.000256918 | -0.06004 | 0.17 | 0.258 | 1 | 0 |
| Srrt | 0.000257753 | -0.03025 | 0.127 | 0.202 | 1 | 0 |
| Trappc4 | 0.00025837 | -0.0371 | 0.152 | 0.234 | 1 | 0 |
| Dld | 0.000258948 | -0.02675 | 0.067 | 0.121 | 1 | 0 |
| Arhgap26 | 0.000259294 | -0.05299 | 0.113 | 0.181 | 1 | 0 |
| Lrrc8c | 0.000259309 | -0.03411 | 0.065 | 0.117 | 1 | 0 |
| Hsd17b10 | 0.000259746 | -0.08786 | 0.106 | 0.168 | 1 | 0 |
| Oxct1 | 0.000259922 | -0.04223 | 0.054 | 0.101 | 1 | 0 |
| Atxn10 | 0.000260149 | -0.02688 | 0.062 | 0.112 | 1 | 0 |
| 1110004F10Rik | 0.000260347 | -0.00736 | 0.171 | 0.265 | 1 | 0 |
| Atp6ap1 | 0.000260954 | -0.0489 | 0.14 | 0.218 | 1 | 0 |
| Psmb6 | 0.000264665 | -0.03676 | 0.235 | 0.347 | 1 | 0 |
| Nae1 | 0.000265399 | -0.02802 | 0.097 | 0.161 | 1 | 0 |
| Fbxw7 | 0.00026793 | -0.03129 | 0.063 | 0.113 | 1 | 0 |
| Coa3 | 0.000268103 | -0.02589 | 0.075 | 0.13 | 1 | 0 |
| Tle3 | 0.0002685 | -0.01315 | 0.074 | 0.13 | 1 | 0 |
| Mrpl48 | 0.000268833 | -0.03515 | 0.064 | 0.115 | 1 | 0 |
| Fam172a | 0.000270378 | -0.03868 | 0.099 | 0.162 | 1 | 0 |
| Dcaf8 | 0.000270755 | -0.06148 | 0.156 | 0.236 | 1 | 0 |
| Tiparp | 0.000276125 | -0.02493 | 0.157 | 0.24 | 1 | 0 |
| C1qbp | 0.000276513 | -0.05533 | 0.152 | 0.232 | 1 | 0 |
| Phf14 | 0.000279955 | -0.06608 | 0.139 | 0.215 | 1 | 0 |
| Nr2c2ap | 0.000281408 | -0.04836 | 0.057 | 0.105 | 1 | 0 |
| Tap2 | 0.000282288 | -0.06306 | 0.124 | 0.195 | 1 | 0 |
| Hmgcr | 0.000283066 | -0.03541 | 0.073 | 0.128 | 1 | 0 |
| Trmt1 | 0.000283498 | -0.0421 | 0.095 | 0.158 | 1 | 0 |
| Coro1a | 0.000283889 | -0.09934 | 0.831 | 0.908 | 1 | 0 |
| Slc1a5 | 0.00028434 | -0.02445 | 0.14 | 0.221 | 1 | 0 |
| Cpsf7 | 0.000285775 | -0.03766 | 0.098 | 0.162 | 1 | 0 |
| Zc3h10 | 0.000288312 | -0.03759 | 0.054 | 0.101 | 1 | 0 |
| Cdk2ap1 | 0.000289188 | -0.04066 | 0.107 | 0.173 | 1 | 0 |
| Donson | 0.000290211 | -0.03467 | 0.071 | 0.124 | 1 | 0 |
| Tug1 | 0.000291952 | -0.05322 | 0.121 | 0.191 | 1 | 0 |
| Pdhb | 0.000293123 | -0.05371 | 0.122 | 0.193 | 1 | 0 |
| Nfx1 | 0.000293557 | -0.06318 | 0.098 | 0.16 | 1 | 0 |
| Letm1 | 0.000293762 | -0.06021 | 0.079 | 0.134 | 1 | 0 |
| Actn4 | 0.000293844 | -0.00368 | 0.13 | 0.208 | 1 | 0 |
| Bank1 | 0.000294268 | -0.10798 | 0.624 | 0.753 | 1 | 0 |
| Ubtf | 0.000294904 | -0.0435 | 0.165 | 0.249 | 1 | 0 |
| Inpp5f | 0.000295487 | -0.04783 | 0.068 | 0.121 | 1 | 0 |
| Ctdspl2 | 0.000296995 | -0.06085 | 0.1 | 0.163 | 1 | 0 |
| Nol12 | 0.000298335 | -0.04904 | 0.063 | 0.112 | 1 | 0 |
| Cdk2ap2 | 0.000298897 | -0.05518 | 0.242 | 0.354 | 1 | 0 |
| Dapk3 | 0.000299692 | -0.03895 | 0.069 | 0.122 | 1 | 0 |
| Ccnl1 | 0.000300471 | -0.06446 | 0.418 | 0.566 | 1 | 0 |
| Nuak2 | 0.000300513 | -0.03361 | 0.11 | 0.179 | 1 | 0 |
| Poldip3 | 0.000302563 | -0.06296 | 0.138 | 0.212 | 1 | 0 |
| Aimp1 | 0.000304882 | -0.03378 | 0.175 | 0.267 | 1 | 0 |
| Safb2 | 0.000306762 | -0.05359 | 0.244 | 0.352 | 1 | 0 |
| Elmo1 | 0.000307276 | -0.05438 | 0.254 | 0.366 | 1 | 0 |
| Atp5b | 0.0003106 | -0.08237 | 0.474 | 0.637 | 1 | 0 |
| Txnl1 | 0.00031066 | -0.04118 | 0.151 | 0.232 | 1 | 0 |
| Ggnbp2 | 0.000314043 | -0.01951 | 0.187 | 0.283 | 1 | 0 |
| Mplkip | 0.000314219 | -0.06405 | 0.127 | 0.199 | 1 | 0 |
| Tnpo3 | 0.00031433 | -0.02824 | 0.119 | 0.189 | 1 | 0 |
| Zeb2 | 0.000315562 | -0.0504 | 0.153 | 0.234 | 1 | 0 |
| Kansl2 | 0.000316514 | -0.03115 | 0.065 | 0.117 | 1 | 0 |
| Fam32a | 0.000318932 | -0.04191 | 0.153 | 0.234 | 1 | 0 |
| Eif2a | 0.000320225 | -0.02819 | 0.091 | 0.152 | 1 | 0 |
| Ppm1a | 0.000321859 | -0.04789 | 0.12 | 0.19 | 1 | 0 |
| Mrps33 | 0.000322269 | -0.03229 | 0.159 | 0.242 | 1 | 0 |
| Arf6 | 0.0003225 | -0.02095 | 0.239 | 0.352 | 1 | 0 |
| Seh1l | 0.000323262 | -0.0392 | 0.068 | 0.121 | 1 | 0 |
| Agfg1 | 0.000324302 | -0.0404 | 0.11 | 0.178 | 1 | 0 |
| Polr1c | 0.000325038 | -0.04463 | 0.067 | 0.119 | 1 | 0 |
| Zmym4 | 0.000325888 | -0.05999 | 0.057 | 0.104 | 1 | 0 |
| Ubxn4 | 0.000327826 | -0.03532 | 0.19 | 0.28 | 1 | 0 |
| Nrf1 | 0.000328788 | -0.02905 | 0.097 | 0.161 | 1 | 0 |
| Hdac1 | 0.000328828 | -0.02363 | 0.142 | 0.221 | 1 | 0 |
| Chchd10 | 0.000329084 | -0.13345 | 0.37 | 0.488 | 1 | 0 |
| Rpa1 | 0.000329784 | -0.0501 | 0.062 | 0.111 | 1 | 0 |
| Ptbp2 | 0.000330029 | -0.06554 | 0.068 | 0.119 | 1 | 0 |
| Ehd1 | 0.000330542 | -0.05706 | 0.184 | 0.273 | 1 | 0 |
| Lrp10 | 0.000331682 | 0.005494 | 0.2 | 0.301 | 1 | 0 |
| Rlf | 0.000332699 | -0.0018 | 0.138 | 0.22 | 1 | 0 |
| Tuba1c | 0.000333797 | -0.01818 | 0.089 | 0.149 | 1 | 0 |
| Man1a | 0.000334245 | -0.06272 | 0.399 | 0.547 | 1 | 0 |
| Cnot4 | 0.000334363 | -0.04622 | 0.152 | 0.233 | 1 | 0 |
| Rapgef1 | 0.000336247 | -0.0432 | 0.113 | 0.18 | 1 | 0 |
| mt-Cytb | 0.000336342 | 0.071552 | 1 | 1 | 1 | 0 |
| Arl6ip6 | 0.000336566 | -0.05506 | 0.074 | 0.128 | 1 | 0 |
| Llph | 0.00033667 | -0.03631 | 0.269 | 0.388 | 1 | 0 |
| Vamp3 | 0.000342534 | -0.02658 | 0.077 | 0.132 | 1 | 0 |
| Irf3 | 0.000342755 | -0.04583 | 0.102 | 0.166 | 1 | 0 |
| Fam220a | 0.000344423 | -0.04521 | 0.066 | 0.117 | 1 | 0 |
| Tmed7 | 0.000344928 | -0.03878 | 0.108 | 0.173 | 1 | 0 |
| Ccdc9 | 0.000347861 | -0.04614 | 0.063 | 0.112 | 1 | 0 |
| Cd2ap | 0.00034893 | -0.04447 | 0.215 | 0.315 | 1 | 0 |
| Bax | 0.000351893 | -0.05725 | 0.214 | 0.308 | 1 | 0 |
| Pias1 | 0.000353895 | -0.00192 | 0.134 | 0.211 | 1 | 0 |
| Bcl7a | 0.000355642 | -0.10181 | 0.074 | 0.125 | 1 | 0 |
| Usp38 | 0.00035614 | -0.04983 | 0.094 | 0.154 | 1 | 0 |
| Trappc10 | 0.000356333 | -0.07314 | 0.082 | 0.137 | 1 | 0 |
| Rassf2 | 0.000357185 | -0.04343 | 0.156 | 0.237 | 1 | 0 |
| Glrx5 | 0.000358125 | -0.04324 | 0.093 | 0.153 | 1 | 0 |
| Tes | 0.000358637 | -0.07585 | 0.091 | 0.149 | 1 | 0 |
| Ywhag | 0.000359002 | -0.04057 | 0.114 | 0.181 | 1 | 0 |
| Naa10 | 0.000359763 | -0.03197 | 0.077 | 0.131 | 1 | 0 |
| Tm9sf3 | 0.000360445 | -0.01825 | 0.269 | 0.391 | 1 | 0 |
| Acbd6 | 0.000365739 | -0.02912 | 0.094 | 0.155 | 1 | 0 |
| Ifi35 | 0.000365808 | -0.05619 | 0.091 | 0.149 | 1 | 0 |
| Jmjd1c | 0.000366434 | -0.04391 | 0.382 | 0.539 | 1 | 0 |
| Trp53 | 0.000367213 | -0.06982 | 0.176 | 0.261 | 1 | 0 |
| Cyp4f18 | 0.000369231 | -0.10285 | 0.24 | 0.344 | 1 | 0 |
| Etnk1 | 0.00037058 | -0.01841 | 0.317 | 0.445 | 1 | 0 |
| Uba2 | 0.000370925 | -0.03221 | 0.094 | 0.155 | 1 | 0 |
| Rbmxl1 | 0.000373694 | -0.02475 | 0.155 | 0.237 | 1 | 0 |
| Tbc1d5 | 0.000375397 | -0.02681 | 0.087 | 0.146 | 1 | 0 |
| Pank2 | 0.000381643 | -0.06099 | 0.069 | 0.119 | 1 | 0 |
| Srsf10 | 0.000381766 | -0.05304 | 0.32 | 0.455 | 1 | 0 |
| Tgfbr2 | 0.000383251 | -0.03848 | 0.23 | 0.335 | 1 | 0 |
| Arhgap31 | 0.000383462 | -0.09283 | 0.079 | 0.131 | 1 | 0 |
| Bfsp2 | 0.000384353 | -0.07688 | 0.088 | 0.143 | 1 | 0 |
| Phf5a | 0.000385956 | -0.03161 | 0.159 | 0.243 | 1 | 0 |
| Cct4 | 0.000389244 | -0.03887 | 0.28 | 0.405 | 1 | 0 |
| Atp5o.1 | 0.000390093 | -0.04429 | 0.294 | 0.412 | 1 | 0 |
| Lgals9 | 0.000391176 | -0.0816 | 0.065 | 0.112 | 1 | 0 |
| Haus3 | 0.000392023 | -0.05384 | 0.074 | 0.126 | 1 | 0 |
| Ensa | 0.000394488 | -0.02975 | 0.155 | 0.236 | 1 | 0 |
| Nufip2 | 0.000402315 | -0.05701 | 0.26 | 0.37 | 1 | 0 |
| Smim15 | 0.000402498 | -0.04159 | 0.077 | 0.131 | 1 | 0 |
| Tmco1 | 0.000402579 | -0.03008 | 0.101 | 0.165 | 1 | 0 |
| Ckap4 | 0.000404058 | -0.019 | 0.056 | 0.103 | 1 | 0 |
| Pak2 | 0.000405104 | -0.0758 | 0.31 | 0.444 | 1 | 0 |
| Sf3b5 | 0.000409052 | -0.04732 | 0.199 | 0.295 | 1 | 0 |
| Med11 | 0.000409534 | -0.05107 | 0.062 | 0.11 | 1 | 0 |
| Slc16a6 | 0.000413262 | -0.06798 | 0.085 | 0.141 | 1 | 0 |
| Kat6b | 0.000416466 | -0.04628 | 0.071 | 0.123 | 1 | 0 |
| Sfr1 | 0.000420317 | -0.08125 | 0.328 | 0.45 | 1 | 0 |
| Lrrk2 | 0.000421822 | -0.07721 | 0.207 | 0.296 | 1 | 0 |
| Nsmce2 | 0.000422035 | -0.02289 | 0.076 | 0.13 | 1 | 0 |
| Pitpnb | 0.000422597 | -0.03488 | 0.067 | 0.118 | 1 | 0 |
| Bbx | 0.000422779 | -0.03364 | 0.152 | 0.232 | 1 | 0 |
| Chchd2 | 0.000424558 | 0.13547 | 0.758 | 0.789 | 1 | 0 |
| Naa35 | 0.000435555 | -0.03483 | 0.059 | 0.106 | 1 | 0 |
| Erlin1 | 0.00043722 | -0.06679 | 0.065 | 0.113 | 1 | 0 |
| Serpinb1a | 0.000440602 | -0.0945 | 0.263 | 0.359 | 1 | 0 |
| Mydgf | 0.000442531 | -0.06751 | 0.073 | 0.124 | 1 | 0 |
| Rps6ka5 | 0.000445571 | -0.06432 | 0.063 | 0.111 | 1 | 0 |
| Skap2 | 0.000446613 | -0.05811 | 0.115 | 0.181 | 1 | 0 |
| Otub1 | 0.000451175 | -0.02514 | 0.062 | 0.11 | 1 | 0 |
| Rsf1 | 0.00045341 | -0.06001 | 0.188 | 0.276 | 1 | 0 |
| Ppm1m | 0.000453809 | -0.04021 | 0.064 | 0.112 | 1 | 0 |
| Cenpb | 0.000453902 | -0.04229 | 0.088 | 0.146 | 1 | 0 |
| Cd180 | 0.000456223 | -0.06502 | 0.078 | 0.13 | 1 | 0 |
| Rnf13 | 0.000460508 | -0.01 | 0.112 | 0.18 | 1 | 0 |
| Mrpl34 | 0.000461691 | -0.02499 | 0.105 | 0.169 | 1 | 0 |
| Bicral | 0.000462712 | -0.05922 | 0.061 | 0.109 | 1 | 0 |
| Usf3 | 0.000463103 | -0.03877 | 0.08 | 0.134 | 1 | 0 |
| Arhgap9 | 0.000464122 | -0.04113 | 0.076 | 0.129 | 1 | 0 |
| Cst3 | 0.000467372 | 0.205251 | 0.524 | 0.538 | 1 | 0 |
| Ccl6 | 0.000467976 | 0.334644 | 0.2 | 0.158 | 1 | 0 |
| Lpcat1 | 0.000469143 | -0.04571 | 0.085 | 0.141 | 1 | 0 |
| Rnf44 | 0.000472254 | -0.02858 | 0.101 | 0.165 | 1 | 0 |
| Ift20 | 0.000472642 | -0.07138 | 0.109 | 0.169 | 1 | 0 |
| Fam193a | 0.00047441 | -0.05207 | 0.121 | 0.189 | 1 | 0 |
| H3f3a | 0.000476384 | 0.064446 | 0.996 | 0.989 | 1 | 0 |
| Tsn | 0.000477312 | -0.00099 | 0.236 | 0.342 | 1 | 0 |
| Rasgrp2 | 0.000479696 | -0.0852 | 0.309 | 0.424 | 1 | 0 |
| Stx8 | 0.000484912 | -0.02185 | 0.083 | 0.14 | 1 | 0 |
| Srp19 | 0.000487141 | -0.01178 | 0.141 | 0.216 | 1 | 0 |
| Clk4 | 0.000491159 | -0.04802 | 0.223 | 0.32 | 1 | 0 |
| Cdc37l1 | 0.000494258 | -0.03534 | 0.151 | 0.227 | 1 | 0 |
| Dcaf11 | 0.000498267 | -0.02912 | 0.069 | 0.121 | 1 | 0 |
| Mrpl30 | 0.000499572 | -0.04748 | 0.229 | 0.331 | 1 | 0 |
| N4bp2l1 | 0.000501863 | -0.05598 | 0.118 | 0.184 | 1 | 0 |
| Gemin7 | 0.000504211 | -0.04013 | 0.116 | 0.183 | 1 | 0 |
| Ddx52 | 0.000504221 | -0.05947 | 0.063 | 0.11 | 1 | 0 |
| Pdcl3 | 0.000504931 | -0.03198 | 0.084 | 0.14 | 1 | 0 |
| Med28 | 0.000508602 | -0.03771 | 0.116 | 0.183 | 1 | 0 |
| Lcor | 0.00050904 | -0.01756 | 0.075 | 0.129 | 1 | 0 |
| Ghitm | 0.000509668 | -0.04752 | 0.21 | 0.303 | 1 | 0 |
| Sdhd | 0.000509854 | -0.01906 | 0.094 | 0.155 | 1 | 0 |
| Chfr | 0.000510202 | -0.03914 | 0.105 | 0.167 | 1 | 0 |
| Pld4 | 0.000510781 | -0.01729 | 0.133 | 0.204 | 1 | 0 |
| Ufd1 | 0.000514254 | -0.06041 | 0.07 | 0.121 | 1 | 0 |
| Ppm1g | 0.000516204 | -0.02911 | 0.2 | 0.294 | 1 | 0 |
| Dcaf13 | 0.000516405 | -0.02359 | 0.077 | 0.13 | 1 | 0 |
| Rpia | 0.000516478 | -0.01149 | 0.079 | 0.132 | 1 | 0 |
| Ppp2r5c | 0.000517221 | -0.0324 | 0.142 | 0.216 | 1 | 0 |
| Zfp292 | 0.000520494 | -0.01386 | 0.245 | 0.354 | 1 | 0 |
| Cox8a | 0.00052221 | 0.151232 | 0.762 | 0.784 | 1 | 0 |
| Adipor1 | 0.00052381 | 0.022298 | 0.156 | 0.242 | 1 | 0 |
| Vdac1 | 0.000526177 | -0.03322 | 0.107 | 0.169 | 1 | 0 |
| Capzb | 0.000527987 | -0.05276 | 0.356 | 0.501 | 1 | 0 |
| Slk | 0.000531603 | -0.06227 | 0.078 | 0.13 | 1 | 0 |
| Ankrd10 | 0.00053451 | -0.03636 | 0.096 | 0.156 | 1 | 0 |
| Scd1 | 0.000535998 | -0.11092 | 0.514 | 0.652 | 1 | 0 |
| Usp9x | 0.000536716 | -0.05317 | 0.215 | 0.308 | 1 | 0 |
| Mrpl13 | 0.00053775 | -0.03726 | 0.066 | 0.116 | 1 | 0 |
| Gabarap | 0.000537939 | 0.163565 | 0.572 | 0.603 | 1 | 0 |
| Eif4ebp2 | 0.000539825 | -0.00917 | 0.15 | 0.229 | 1 | 0 |
| Pycard | 0.000549621 | -0.00378 | 0.135 | 0.209 | 1 | 0 |
| St8sia4 | 0.000551136 | -0.0321 | 0.348 | 0.479 | 1 | 0 |
| Mat2b | 0.000551659 | -0.0492 | 0.178 | 0.263 | 1 | 0 |
| Psmd12 | 0.000552068 | -0.03693 | 0.116 | 0.181 | 1 | 0 |
| Cop1 | 0.000554468 | -0.02379 | 0.163 | 0.245 | 1 | 0 |
| Arid5a | 0.000554876 | -0.07318 | 0.305 | 0.421 | 1 | 0 |
| Rbms1 | 0.000563376 | 0.010921 | 0.193 | 0.286 | 1 | 0 |
| Emc6 | 0.000567198 | -0.03384 | 0.208 | 0.305 | 1 | 0 |
| Rad50 | 0.000568521 | -0.06 | 0.094 | 0.15 | 1 | 0 |
| Ing1 | 0.000570914 | -0.02127 | 0.16 | 0.242 | 1 | 0 |
| Pik3r1 | 0.000571619 | -0.04063 | 0.132 | 0.203 | 1 | 0 |
| Srcap | 0.000573875 | -0.03944 | 0.091 | 0.148 | 1 | 0 |
| Prkar1a | 0.000576562 | -0.01982 | 0.311 | 0.444 | 1 | 0 |
| Ndufs8 | 0.000577716 | -0.02391 | 0.152 | 0.229 | 1 | 0 |
| Ndufa8 | 0.000583092 | -0.01521 | 0.136 | 0.209 | 1 | 0 |
| Plcl2 | 0.000588439 | -0.03176 | 0.069 | 0.119 | 1 | 0 |
| Psmc4 | 0.000590485 | -0.01418 | 0.145 | 0.223 | 1 | 0 |
| Fnbp4 | 0.000590627 | -0.04381 | 0.153 | 0.229 | 1 | 0 |
| Lst1 | 0.000590835 | 0.217594 | 0.126 | 0.081 | 1 | 0 |
| Usp36 | 0.000592412 | -0.04253 | 0.065 | 0.113 | 1 | 0 |
| Bcl2a1b | 0.000592526 | 0.241892 | 0.342 | 0.303 | 1 | 0 |
| Arih2 | 0.000594034 | -0.05374 | 0.091 | 0.148 | 1 | 0 |
| Mon2 | 0.000596272 | -0.01751 | 0.103 | 0.167 | 1 | 0 |
| H2-Aa | 0.000602138 | 0.082936 | 0.988 | 0.981 | 1 | 0 |
| Phb2 | 0.000609696 | -0.03348 | 0.203 | 0.298 | 1 | 0 |
| Hsd17b12 | 0.000610667 | -0.0238 | 0.081 | 0.136 | 1 | 0 |
| Phf21a | 0.0006138 | -0.03002 | 0.115 | 0.181 | 1 | 0 |
| Pomp | 0.000618569 | -0.02384 | 0.278 | 0.4 | 1 | 0 |
| Pop5 | 0.000619718 | -0.02261 | 0.123 | 0.191 | 1 | 0 |
| Tfeb | 0.000621299 | -0.03742 | 0.067 | 0.116 | 1 | 0 |
| Eif4a3 | 0.00062324 | -0.02285 | 0.154 | 0.234 | 1 | 0 |
| Dusp1 | 0.000625052 | 0.166636 | 0.543 | 0.529 | 1 | 0 |
| Nub1 | 0.000625696 | -0.03471 | 0.114 | 0.179 | 1 | 0 |
| Pttg1ip | 0.000628594 | -0.05943 | 0.069 | 0.118 | 1 | 0 |
| Egln2 | 0.000632787 | -0.06894 | 0.23 | 0.327 | 1 | 0 |
| Ncoa1 | 0.000637456 | -0.08635 | 0.088 | 0.142 | 1 | 0 |
| Polr2h | 0.000638532 | -0.04483 | 0.058 | 0.104 | 1 | 0 |
| Psma5 | 0.000638839 | -0.01569 | 0.176 | 0.265 | 1 | 0 |
| Spg7 | 0.000652662 | -0.06133 | 0.062 | 0.107 | 1 | 0 |
| Anapc1 | 0.000656678 | -0.05877 | 0.065 | 0.113 | 1 | 0 |
| D17Wsu92e | 0.000658749 | -0.03351 | 0.065 | 0.113 | 1 | 0 |
| Rbm8a | 0.00065903 | -0.00825 | 0.216 | 0.317 | 1 | 0 |
| Ing5 | 0.000659357 | -0.04171 | 0.075 | 0.126 | 1 | 0 |
| Slc25a4 | 0.000659623 | -0.04941 | 0.132 | 0.199 | 1 | 0 |
| Ech1 | 0.000663767 | -0.02012 | 0.087 | 0.143 | 1 | 0 |
| Anapc16 | 0.000664991 | -0.03319 | 0.185 | 0.277 | 1 | 0 |
| Fkbp3 | 0.000665218 | -0.0403 | 0.234 | 0.333 | 1 | 0 |
| Nr3c1 | 0.000669633 | -0.05996 | 0.201 | 0.289 | 1 | 0 |
| Sesn3 | 0.000674177 | -0.06351 | 0.182 | 0.266 | 1 | 0 |
| Nxt1 | 0.000674208 | -0.02946 | 0.063 | 0.11 | 1 | 0 |
| Nrd1 | 0.00067504 | -0.01823 | 0.196 | 0.288 | 1 | 0 |
| Calm3 | 0.000676909 | -0.02497 | 0.19 | 0.28 | 1 | 0 |
| Ccdc124 | 0.000678127 | -0.01981 | 0.078 | 0.13 | 1 | 0 |
| Ranbp2 | 0.000688085 | -0.07801 | 0.265 | 0.372 | 1 | 0 |
| Spcs2 | 0.000691088 | -0.08833 | 0.302 | 0.416 | 1 | 0 |
| Fosl2 | 0.00069367 | 0.293333 | 0.155 | 0.11 | 1 | 0 |
| Cct8 | 0.000698324 | -0.03627 | 0.241 | 0.344 | 1 | 0 |
| 4921524J17Rik | 0.000704106 | -0.02922 | 0.106 | 0.167 | 1 | 0 |
| Polr2m | 0.000706833 | -0.03942 | 0.148 | 0.222 | 1 | 0 |
| Mdp1 | 0.000708235 | -0.02638 | 0.098 | 0.156 | 1 | 0 |
| Imp3 | 0.000709479 | -0.05495 | 0.237 | 0.335 | 1 | 0 |
| Cops6 | 0.000713688 | -0.03193 | 0.11 | 0.173 | 1 | 0 |
| Wdr44 | 0.000714327 | -0.04056 | 0.072 | 0.122 | 1 | 0 |
| Tmed10 | 0.000715382 | -0.0452 | 0.294 | 0.405 | 1 | 0 |
| Coro1b | 0.000716853 | -0.03099 | 0.074 | 0.125 | 1 | 0 |
| Nmi | 0.00072115 | -0.02901 | 0.089 | 0.144 | 1 | 0 |
| Vcpip1 | 0.000724306 | -0.02369 | 0.09 | 0.147 | 1 | 0 |
| Snrpb2 | 0.00072725 | -0.01445 | 0.096 | 0.155 | 1 | 0 |
| Srsf4 | 0.000727687 | -0.06072 | 0.066 | 0.113 | 1 | 0 |
| Mef2a | 0.000727957 | -0.0337 | 0.154 | 0.229 | 1 | 0 |
| Sec16a | 0.000728153 | -0.04698 | 0.065 | 0.111 | 1 | 0 |
| Cfl2 | 0.000748036 | -0.04534 | 0.06 | 0.105 | 1 | 0 |
| Cct7 | 0.000750156 | -0.0626 | 0.269 | 0.378 | 1 | 0 |
| Smap1 | 0.000751462 | -0.03813 | 0.15 | 0.224 | 1 | 0 |
| Rabgap1l | 0.000752019 | -0.10082 | 0.355 | 0.464 | 1 | 0 |
| Maea | 0.000754635 | -0.02601 | 0.085 | 0.141 | 1 | 0 |
| Emc3 | 0.000760159 | -0.03014 | 0.15 | 0.224 | 1 | 0 |
| Zfx | 0.000760343 | -0.0549 | 0.095 | 0.152 | 1 | 0 |
| Ctnnbl1 | 0.000767958 | -0.03599 | 0.059 | 0.104 | 1 | 0 |
| Aftph | 0.000775094 | -0.0294 | 0.097 | 0.155 | 1 | 0 |
| Ier3ip1 | 0.0007773 | -0.00504 | 0.186 | 0.277 | 1 | 0 |
| Cdc34 | 0.000777456 | -0.01955 | 0.076 | 0.128 | 1 | 0 |
| Nras | 0.000777648 | -0.00729 | 0.118 | 0.185 | 1 | 0 |
| Pikfyve | 0.000778688 | -0.00248 | 0.1 | 0.161 | 1 | 0 |
| Cic | 0.000780901 | -0.01346 | 0.097 | 0.156 | 1 | 0 |
| Rnf111 | 0.000782439 | -0.04519 | 0.158 | 0.234 | 1 | 0 |
| Morc3 | 0.000785599 | -0.03762 | 0.137 | 0.206 | 1 | 0 |
| Derl1 | 0.000787616 | -0.0243 | 0.123 | 0.19 | 1 | 0 |
| Ociad1 | 0.000787768 | -0.02792 | 0.162 | 0.242 | 1 | 0 |
| Tomm22 | 0.000789188 | -0.04171 | 0.262 | 0.37 | 1 | 0 |
| Supt20 | 0.000790877 | -0.02582 | 0.146 | 0.22 | 1 | 0 |
| Bsg | 0.000794859 | -0.01983 | 0.178 | 0.265 | 1 | 0 |
| Taf1d | 0.000802682 | -0.06443 | 0.326 | 0.45 | 1 | 0 |
| Ctps2 | 0.000805025 | -0.03325 | 0.071 | 0.121 | 1 | 0 |
| Pcmtd1 | 0.000808015 | -0.02662 | 0.223 | 0.316 | 1 | 0 |
| Rmnd5a | 0.000809473 | -0.02073 | 0.085 | 0.14 | 1 | 0 |
| Ypel3 | 0.000810669 | -0.05945 | 0.26 | 0.362 | 1 | 0 |
| Ly6a | 0.000821043 | -0.2166 | 0.336 | 0.42 | 1 | 0 |
| Bcl11a | 0.000822505 | -0.06032 | 0.277 | 0.382 | 1 | 0 |
| Pcbd2 | 0.000825631 | -0.04747 | 0.066 | 0.113 | 1 | 0 |
| Fam49b | 0.000827685 | -0.07426 | 0.418 | 0.562 | 1 | 0 |
| Slc12a6 | 0.000829593 | -0.01835 | 0.244 | 0.353 | 1 | 0 |
| Foxp1 | 0.000833536 | -0.11774 | 0.746 | 0.848 | 1 | 0 |
| Uba5 | 0.000839368 | -0.02918 | 0.061 | 0.106 | 1 | 0 |
| AW549877 | 0.00084125 | -0.00655 | 0.067 | 0.116 | 1 | 0 |
| Twf2 | 0.000849946 | -0.03927 | 0.094 | 0.15 | 1 | 0 |
| Lrrc8d | 0.000857386 | -0.04173 | 0.09 | 0.144 | 1 | 0 |
| Gsk3a | 0.000863543 | -0.03762 | 0.096 | 0.154 | 1 | 0 |
| Prps1 | 0.000864638 | -0.03674 | 0.064 | 0.11 | 1 | 0 |
| Tnfrsf13c | 0.00086711 | -0.05893 | 0.219 | 0.31 | 1 | 0 |
| Myadm | 0.000870464 | -0.01237 | 0.073 | 0.123 | 1 | 0 |
| Cpsf2 | 0.000870891 | -0.03582 | 0.122 | 0.185 | 1 | 0 |
| Rcor1 | 0.000877024 | -0.05781 | 0.079 | 0.129 | 1 | 0 |
| Sptlc2 | 0.000879014 | -0.02199 | 0.069 | 0.118 | 1 | 0 |
| Tmx1 | 0.000881207 | -0.02361 | 0.075 | 0.125 | 1 | 0 |
| Gtf3c2 | 0.000886924 | -0.02779 | 0.095 | 0.153 | 1 | 0 |
| Virma | 0.000891067 | -0.04904 | 0.078 | 0.129 | 1 | 0 |
| Acap1 | 0.000895733 | -0.03288 | 0.122 | 0.187 | 1 | 0 |
| Taok1 | 0.000898474 | 0.004353 | 0.168 | 0.251 | 1 | 0 |
| Atxn7l1 | 0.000899788 | -0.04413 | 0.061 | 0.105 | 1 | 0 |
| Mrpl51 | 0.000905943 | -0.03156 | 0.109 | 0.169 | 1 | 0 |
| Rps2 | 0.000911591 | -0.09394 | 0.995 | 0.999 | 1 | 0 |
| Golph3 | 0.000911678 | -0.03333 | 0.099 | 0.158 | 1 | 0 |
| Mrps18b | 0.000921744 | -0.02716 | 0.059 | 0.104 | 1 | 0 |
| Dnm2 | 0.000922158 | -0.01326 | 0.153 | 0.23 | 1 | 0 |
| Lyl1 | 0.00092425 | -0.08038 | 0.081 | 0.131 | 1 | 0 |
| Thrap3 | 0.000925186 | -0.03375 | 0.28 | 0.395 | 1 | 0 |
| Polr3k | 0.000928992 | -0.04763 | 0.062 | 0.106 | 1 | 0 |
| Sema4d | 0.000929953 | -0.05625 | 0.114 | 0.174 | 1 | 0 |
| Ythdf1 | 0.000934529 | -0.04414 | 0.075 | 0.124 | 1 | 0 |
| Nubp1 | 0.000939285 | -0.02337 | 0.116 | 0.18 | 1 | 0 |
| Kmt5a | 0.000946183 | 0.003512 | 0.119 | 0.185 | 1 | 0 |
| Ncf1 | 0.000946289 | -0.00212 | 0.19 | 0.28 | 1 | 0 |
| Ndufv2 | 0.000946465 | -0.03367 | 0.129 | 0.198 | 1 | 0 |
| Cep295 | 0.000951088 | -0.05207 | 0.064 | 0.109 | 1 | 0 |
| Ttc14 | 0.000954948 | -0.0228 | 0.3 | 0.425 | 1 | 0 |
| Gpatch11 | 0.00095712 | -0.06873 | 0.059 | 0.101 | 1 | 0 |
| Abi3 | 0.000957268 | -0.0482 | 0.059 | 0.103 | 1 | 0 |
| Rrbp1 | 0.000962619 | -0.04527 | 0.328 | 0.452 | 1 | 0 |
| Zswim8 | 0.000967891 | -0.02531 | 0.063 | 0.109 | 1 | 0 |
| Lmo2 | 0.000976799 | -0.09003 | 0.267 | 0.362 | 1 | 0 |
| Ccar1 | 0.000980756 | -0.02586 | 0.231 | 0.332 | 1 | 0 |
| Ubr3 | 0.000985014 | -0.04698 | 0.079 | 0.129 | 1 | 0 |
| Uqcrh | 0.000996338 | 0.133976 | 0.789 | 0.844 | 1 | 0 |
| Nop14 | 0.000997033 | -0.01231 | 0.094 | 0.152 | 1 | 0 |
| Nap1l4 | 0.001000622 | -0.04855 | 0.147 | 0.218 | 1 | 0 |
| Gimap1 | 0.001007258 | -0.07244 | 0.339 | 0.462 | 1 | 0 |
| Dnajc9 | 0.00100821 | -0.05279 | 0.156 | 0.229 | 1 | 0 |
| Wac | 0.001009229 | -0.0025 | 0.24 | 0.345 | 1 | 0 |
| Napa | 0.001009788 | -0.01741 | 0.109 | 0.171 | 1 | 0 |
| Mrps16 | 0.001012008 | -0.00346 | 0.167 | 0.248 | 1 | 0 |
| Swi5 | 0.001012128 | -0.05302 | 0.214 | 0.307 | 1 | 0 |
| Hist1h1c | 0.0010127 | -0.11371 | 0.149 | 0.214 | 1 | 0 |
| Rassf3 | 0.001018497 | -0.03635 | 0.162 | 0.239 | 1 | 0 |
| Surf4 | 0.001021593 | -0.0221 | 0.095 | 0.153 | 1 | 0 |
| Nudt21 | 0.0010275 | -0.0121 | 0.139 | 0.212 | 1 | 0 |
| Hipk1 | 0.001031163 | 0.016333 | 0.241 | 0.352 | 1 | 0 |
| Map2k1 | 0.001033435 | -0.01026 | 0.119 | 0.184 | 1 | 0 |
| Fam111a | 0.001037419 | -0.06119 | 0.191 | 0.273 | 1 | 0 |
| Gnas | 0.001037683 | -0.10786 | 0.692 | 0.81 | 1 | 0 |
| Commd6 | 0.001038297 | -0.02933 | 0.098 | 0.155 | 1 | 0 |
| Tor1aip2 | 0.001057655 | -0.02738 | 0.083 | 0.136 | 1 | 0 |
| Zfp397 | 0.001061723 | -0.05656 | 0.069 | 0.116 | 1 | 0 |
| Sept6 | 0.001062955 | -0.02367 | 0.183 | 0.266 | 1 | 0 |
| Zfp326 | 0.001064342 | -0.02953 | 0.119 | 0.183 | 1 | 0 |
| Rars | 0.001067348 | -0.01674 | 0.06 | 0.104 | 1 | 0 |
| Csnk1g3 | 0.001067526 | -0.01995 | 0.272 | 0.381 | 1 | 0 |
| Tmx3 | 0.001067957 | -0.01662 | 0.081 | 0.134 | 1 | 0 |
| Rbm17 | 0.0010684 | -0.0132 | 0.125 | 0.191 | 1 | 0 |
| Pum2 | 0.001082056 | -0.05906 | 0.282 | 0.388 | 1 | 0 |
| Ubxn1 | 0.001083624 | 0.0037 | 0.272 | 0.391 | 1 | 0 |
| Zzz3 | 0.001096543 | -0.02365 | 0.095 | 0.152 | 1 | 0 |
| Pip5k1c | 0.001096825 | -0.05091 | 0.074 | 0.122 | 1 | 0 |
| Gmps | 0.001100153 | -0.01327 | 0.096 | 0.153 | 1 | 0 |
| Eif4e | 0.001102553 | -0.03776 | 0.101 | 0.159 | 1 | 0 |
| Dock10 | 0.001104607 | -0.05385 | 0.316 | 0.422 | 1 | 0 |
| Rcc2 | 0.001105973 | -0.01538 | 0.181 | 0.265 | 1 | 0 |
| Erp29 | 0.001107255 | -0.07063 | 0.433 | 0.563 | 1 | 0 |
| Uqcc3 | 0.001107338 | -0.02696 | 0.101 | 0.158 | 1 | 0 |
| Syk | 0.001110129 | -0.10995 | 0.554 | 0.677 | 1 | 0 |
| Csnk1a1 | 0.001119232 | -0.03372 | 0.34 | 0.473 | 1 | 0 |
| Ccdc71l | 0.001142666 | -0.01766 | 0.06 | 0.104 | 1 | 0 |
| Prpf31 | 0.001143513 | -0.03043 | 0.063 | 0.107 | 1 | 0 |
| Snn | 0.001148644 | -0.05753 | 0.186 | 0.268 | 1 | 0 |
| Rpl7 | 0.001149327 | 0.067089 | 0.993 | 0.996 | 1 | 0 |
| Kbtbd2 | 0.001149957 | -0.06193 | 0.075 | 0.123 | 1 | 0 |
| Themis2 | 0.001151026 | -0.03998 | 0.086 | 0.138 | 1 | 0 |
| Dpp4 | 0.001162225 | -0.07547 | 0.074 | 0.121 | 1 | 0 |
| Chmp2a | 0.001166435 | -0.04677 | 0.21 | 0.297 | 1 | 0 |
| Ino80d | 0.001177471 | -0.00638 | 0.17 | 0.252 | 1 | 0 |
| Psd4 | 0.001185858 | -0.02312 | 0.06 | 0.104 | 1 | 0 |
| Sfswap | 0.001195888 | -0.04803 | 0.121 | 0.185 | 1 | 0 |
| Prmt7 | 0.001198066 | -0.03686 | 0.06 | 0.103 | 1 | 0 |
| Cox6c | 0.001212266 | 0.183674 | 0.6 | 0.625 | 1 | 0 |
| Eif3i | 0.001224359 | -0.05609 | 0.358 | 0.493 | 1 | 0 |
| Xylt1 | 0.001225272 | -0.02058 | 0.125 | 0.191 | 1 | 0 |
| Hsf1 | 0.001228979 | -0.00645 | 0.066 | 0.113 | 1 | 0 |
| Ifitm3 | 0.001251293 | 0.182976 | 0.118 | 0.076 | 1 | 0 |
| Mrps25 | 0.00125254 | -0.03294 | 0.065 | 0.111 | 1 | 0 |
| Cdkn1b | 0.001262989 | -0.04131 | 0.326 | 0.449 | 1 | 0 |
| Atad2b | 0.001266504 | -0.05232 | 0.126 | 0.19 | 1 | 0 |
| Farsb | 0.001274339 | -0.01691 | 0.156 | 0.229 | 1 | 0 |
| Actr3 | 0.001299465 | -0.11497 | 0.575 | 0.699 | 1 | 0 |
| Atp6v0b | 0.001301643 | -0.02778 | 0.403 | 0.561 | 1 | 0 |
| Papd4 | 0.001302325 | -0.01265 | 0.07 | 0.117 | 1 | 0 |
| Pfdn1 | 0.001310221 | -0.00971 | 0.11 | 0.171 | 1 | 0 |
| Man2a2 | 0.001310577 | -0.00584 | 0.067 | 0.115 | 1 | 0 |
| Spty2d1 | 0.00131778 | -0.01998 | 0.154 | 0.228 | 1 | 0 |
| Capza1 | 0.001320466 | -0.04014 | 0.209 | 0.296 | 1 | 0 |
| Nudcd3 | 0.001322823 | -0.01015 | 0.151 | 0.224 | 1 | 0 |
| Psme1 | 0.001324708 | -0.08377 | 0.435 | 0.579 | 1 | 0 |
| Tm9sf2 | 0.001331117 | -0.01048 | 0.12 | 0.185 | 1 | 0 |
| Ubac2 | 0.001342674 | -0.01941 | 0.088 | 0.141 | 1 | 0 |
| Rnf187 | 0.001347443 | -0.02971 | 0.182 | 0.265 | 1 | 0 |
| Grina | 0.001350269 | 0.214602 | 0.158 | 0.115 | 1 | 0 |
| Ap3d1 | 0.001357762 | -0.01781 | 0.065 | 0.111 | 1 | 0 |
| Fgd2 | 0.001360806 | -0.03589 | 0.1 | 0.156 | 1 | 0 |
| Vps28 | 0.001362342 | -0.03058 | 0.191 | 0.273 | 1 | 0 |
| Dennd6a | 0.001362462 | -0.03382 | 0.084 | 0.135 | 1 | 0 |
| BC004004 | 0.001365058 | -0.02606 | 0.088 | 0.141 | 1 | 0 |
| Glyr1 | 0.001367498 | -0.03281 | 0.156 | 0.23 | 1 | 0 |
| Tbrg1 | 0.001371 | -0.03888 | 0.156 | 0.229 | 1 | 0 |
| 1810022K09Rik | 0.001371996 | -0.02644 | 0.148 | 0.22 | 1 | 0 |
| Rsrp1 | 0.001373369 | 0.137996 | 0.686 | 0.715 | 1 | 0 |
| Ankrd13c | 0.001375597 | -0.02817 | 0.107 | 0.166 | 1 | 0 |
| Eapp | 0.001382438 | -0.01385 | 0.141 | 0.21 | 1 | 0 |
| Cnot6l | 0.001387675 | -0.04537 | 0.299 | 0.415 | 1 | 0 |
| Ubxn2a | 0.001399563 | -0.07545 | 0.078 | 0.125 | 1 | 0 |
| Plekho2 | 0.001409991 | -0.02511 | 0.143 | 0.211 | 1 | 0 |
| Wipf2 | 0.001411706 | -0.04475 | 0.073 | 0.121 | 1 | 0 |
| Slamf6 | 0.001418493 | -0.05804 | 0.178 | 0.255 | 1 | 0 |
| Gtpbp2 | 0.001425356 | -0.05363 | 0.14 | 0.206 | 1 | 0 |
| Gramd3 | 0.001425524 | -0.05159 | 0.235 | 0.323 | 1 | 0 |
| Arap1 | 0.001426445 | -0.0232 | 0.113 | 0.174 | 1 | 0 |
| Csnk2b | 0.001430094 | -0.00879 | 0.169 | 0.251 | 1 | 0 |
| Exoc5 | 0.001439585 | 0.003699 | 0.11 | 0.171 | 1 | 0 |
| Xpr1 | 0.001444964 | -0.02571 | 0.161 | 0.235 | 1 | 0 |
| 2010007H06Rik | 0.001447147 | -0.03312 | 0.059 | 0.101 | 1 | 0 |
| Snx1 | 0.001448478 | -0.05001 | 0.073 | 0.119 | 1 | 0 |
| Ntan1 | 0.001454363 | -0.0167 | 0.133 | 0.198 | 1 | 0 |
| Got1 | 0.001456033 | -0.02195 | 0.176 | 0.255 | 1 | 0 |
| Atp5a1 | 0.001475438 | -0.07607 | 0.417 | 0.557 | 1 | 0 |
| Tomm40 | 0.001476527 | -0.03029 | 0.088 | 0.14 | 1 | 0 |
| Btg1 | 0.001486143 | 0.092859 | 0.949 | 0.952 | 1 | 0 |
| Mff | 0.001489178 | 0.002069 | 0.137 | 0.206 | 1 | 0 |
| Fcer2a | 0.001493596 | -0.14715 | 0.31 | 0.393 | 1 | 0 |
| Pptc7 | 0.001496869 | -0.03234 | 0.074 | 0.122 | 1 | 0 |
| Txn1 | 0.001500325 | 0.215457 | 0.43 | 0.425 | 1 | 0 |
| Mapk1 | 0.001513425 | -0.01797 | 0.21 | 0.3 | 1 | 0 |
| 2310035C23Rik | 0.001514488 | 0.008905 | 0.089 | 0.143 | 1 | 0 |
| Ppil2 | 0.001517586 | -0.03651 | 0.103 | 0.159 | 1 | 0 |
| Sppl2a | 0.001517771 | 0.0034 | 0.187 | 0.272 | 1 | 0 |
| Gcn1l1 | 0.001520352 | -0.02689 | 0.088 | 0.141 | 1 | 0 |
| Psen1 | 0.001529147 | -0.01639 | 0.059 | 0.101 | 1 | 0 |
| Brd9 | 0.001532641 | -0.05562 | 0.123 | 0.185 | 1 | 0 |
| Yipf3 | 0.001538229 | -0.00625 | 0.087 | 0.14 | 1 | 0 |
| Sra1 | 0.001542864 | -0.03087 | 0.129 | 0.195 | 1 | 0 |
| Trim35 | 0.001552589 | -0.06159 | 0.161 | 0.229 | 1 | 0 |
| Terf1 | 0.001555924 | -0.0284 | 0.083 | 0.134 | 1 | 0 |
| Fam133b | 0.001556046 | 0.015783 | 0.192 | 0.28 | 1 | 0 |
| Hmgn1 | 0.001567048 | -0.06909 | 0.362 | 0.476 | 1 | 0 |
| Brap | 0.001578547 | -0.02584 | 0.08 | 0.129 | 1 | 0 |
| Plac8 | 0.001581423 | -0.17412 | 0.471 | 0.538 | 1 | 0 |
| Fyn | 0.001601572 | -0.0367 | 0.068 | 0.113 | 1 | 0 |
| Mast4 | 0.001605425 | -0.03379 | 0.123 | 0.186 | 1 | 0 |
| Sharpin | 0.001608461 | -0.01014 | 0.094 | 0.149 | 1 | 0 |
| Ube2e1 | 0.001626396 | -0.01698 | 0.125 | 0.189 | 1 | 0 |
| Fxr1 | 0.001629975 | -0.02157 | 0.189 | 0.272 | 1 | 0 |
| Edem1 | 0.001631518 | -0.09123 | 0.071 | 0.116 | 1 | 0 |
| Lims1 | 0.001647937 | -0.0243 | 0.111 | 0.171 | 1 | 0 |
| Arid1a | 0.001655526 | -0.01937 | 0.269 | 0.383 | 1 | 0 |
| Mbnl2 | 0.001658874 | -0.02275 | 0.191 | 0.276 | 1 | 0 |
| Tsc22d4 | 0.001675351 | -0.0417 | 0.301 | 0.421 | 1 | 0 |
| Nisch | 0.001680356 | -0.0364 | 0.19 | 0.273 | 1 | 0 |
| Zfp871 | 0.001685061 | 0.002769 | 0.144 | 0.215 | 1 | 0 |
| Acat1 | 0.001710687 | -0.04884 | 0.068 | 0.112 | 1 | 0 |
| Ndufs3 | 0.001723283 | -0.01346 | 0.146 | 0.22 | 1 | 0 |
| Ube2k | 0.001729876 | 0.021438 | 0.219 | 0.319 | 1 | 0 |
| Lrch1 | 0.001735635 | -0.05421 | 0.072 | 0.117 | 1 | 0 |
| Maz | 0.001735793 | -0.03324 | 0.108 | 0.165 | 1 | 0 |
| 2510039O18Rik | 0.001737086 | -0.02958 | 0.073 | 0.119 | 1 | 0 |
| St3gal1 | 0.001753237 | -0.00416 | 0.129 | 0.195 | 1 | 0 |
| Uchl5 | 0.001756351 | -0.0135 | 0.069 | 0.115 | 1 | 0 |
| Usp7 | 0.001757643 | 0.007137 | 0.198 | 0.286 | 1 | 0 |
| Osbpl8 | 0.001762407 | -0.01017 | 0.148 | 0.217 | 1 | 0 |
| Phb | 0.00176651 | -0.02285 | 0.069 | 0.115 | 1 | 0 |
| Commd8 | 0.001768449 | -0.02104 | 0.095 | 0.149 | 1 | 0 |
| Ccser2 | 0.001795624 | -0.01548 | 0.065 | 0.109 | 1 | 0 |
| Rev3l | 0.0018001 | -0.03714 | 0.074 | 0.121 | 1 | 0 |
| Scpep1 | 0.001803017 | 0.005333 | 0.102 | 0.16 | 1 | 0 |
| Ctbp1 | 0.001832663 | 0.00987 | 0.178 | 0.259 | 1 | 0 |
| Smc5 | 0.001833474 | -0.04828 | 0.067 | 0.111 | 1 | 0 |
| Azin1 | 0.001835334 | -0.02082 | 0.148 | 0.218 | 1 | 0 |
| Selenok | 0.001844198 | 0.219526 | 0.466 | 0.483 | 1 | 0 |
| Ell2 | 0.001888739 | -0.00046 | 0.118 | 0.179 | 1 | 0 |
| Mrps15 | 0.001911929 | -0.02733 | 0.085 | 0.135 | 1 | 0 |
| Ly86 | 0.00192376 | -0.05675 | 0.469 | 0.609 | 1 | 0 |
| Prkd3 | 0.001929794 | -0.02633 | 0.065 | 0.107 | 1 | 0 |
| Gnai3 | 0.001930489 | 0.00893 | 0.142 | 0.211 | 1 | 0 |
| Cfap43 | 0.00194786 | -0.04579 | 0.123 | 0.183 | 1 | 0 |
| Hikeshi | 0.001950716 | -0.02024 | 0.068 | 0.112 | 1 | 0 |
| Sec13 | 0.001953855 | -0.0341 | 0.08 | 0.128 | 1 | 0 |
| Gpi1 | 0.001957872 | -0.03146 | 0.307 | 0.428 | 1 | 0 |
| Epsti1 | 0.001965539 | -0.0404 | 0.132 | 0.195 | 1 | 0 |
| Limd2 | 0.001977336 | -0.06585 | 0.425 | 0.554 | 1 | 0 |
| Prdx5 | 0.001993373 | 0.243504 | 0.407 | 0.401 | 1 | 0 |
| Tbc1d1 | 0.00199703 | -0.01534 | 0.152 | 0.223 | 1 | 0 |
| Hint3 | 0.002011052 | -0.03014 | 0.072 | 0.117 | 1 | 0 |
| Zcrb1 | 0.002011905 | -0.01403 | 0.192 | 0.277 | 1 | 0 |
| Npm3 | 0.002014153 | 0.000433 | 0.103 | 0.159 | 1 | 0 |
| Kat7 | 0.002043099 | -0.01268 | 0.072 | 0.118 | 1 | 0 |
| Nfkbid | 0.002046493 | 0.268272 | 0.335 | 0.305 | 1 | 0 |
| Stat6 | 0.00204815 | -0.04263 | 0.111 | 0.171 | 1 | 0 |
| Rps12 | 0.002062272 | 0.076405 | 0.998 | 0.995 | 1 | 0 |
| Esyt2 | 0.002063065 | -0.01496 | 0.08 | 0.13 | 1 | 0 |
| Mapk14 | 0.002077991 | -0.02651 | 0.063 | 0.105 | 1 | 0 |
| Sorl1 | 0.002089919 | -0.08997 | 0.407 | 0.52 | 1 | 0 |
| Dhx40 | 0.002092351 | -0.05203 | 0.148 | 0.212 | 1 | 0 |
| Pdha1 | 0.002093573 | -0.02399 | 0.087 | 0.137 | 1 | 0 |
| Gatd1 | 0.00211352 | -0.01753 | 0.066 | 0.11 | 1 | 0 |
| Bbip1 | 0.002121952 | -0.04264 | 0.086 | 0.135 | 1 | 0 |
| Ccdc186 | 0.002142591 | -0.06842 | 0.069 | 0.112 | 1 | 0 |
| Tulp4 | 0.002145203 | 0.000597 | 0.093 | 0.146 | 1 | 0 |
| Cox16 | 0.002147657 | -0.0094 | 0.069 | 0.113 | 1 | 0 |
| Smc6 | 0.002154396 | -0.05649 | 0.285 | 0.384 | 1 | 0 |
| Mpdu1 | 0.002159294 | -0.01474 | 0.069 | 0.113 | 1 | 0 |
| Tmem168 | 0.00218432 | -0.04482 | 0.065 | 0.106 | 1 | 0 |
| Ndufb7 | 0.002185158 | -0.00995 | 0.261 | 0.364 | 1 | 0 |
| Gsk3b | 0.002209484 | -0.04872 | 0.173 | 0.249 | 1 | 0 |
| Serf2 | 0.002218432 | -0.05791 | 0.745 | 0.851 | 1 | 0 |
| Ccnt2 | 0.002220771 | -0.0004 | 0.135 | 0.202 | 1 | 0 |
| Kmt2b | 0.002224359 | -0.01426 | 0.102 | 0.158 | 1 | 0 |
| Dnmt1 | 0.002237124 | -0.03508 | 0.062 | 0.103 | 1 | 0 |
| Zdhhc20 | 0.002245952 | -0.00098 | 0.174 | 0.251 | 1 | 0 |
| Commd4 | 0.00225261 | -0.0188 | 0.128 | 0.192 | 1 | 0 |
| Tubb4b | 0.002253091 | -0.03304 | 0.295 | 0.405 | 1 | 0 |
| Ccni | 0.002270393 | 0.002473 | 0.154 | 0.224 | 1 | 0 |
| Psmd9 | 0.002270884 | -0.03338 | 0.074 | 0.119 | 1 | 0 |
| Ubr2 | 0.002282466 | 0.016653 | 0.133 | 0.199 | 1 | 0 |
| Ssr1 | 0.002292071 | 0.021665 | 0.135 | 0.203 | 1 | 0 |
| Ssna1 | 0.002294707 | -0.01323 | 0.066 | 0.11 | 1 | 0 |
| Lsm1 | 0.002313438 | -0.01551 | 0.085 | 0.135 | 1 | 0 |
| Eea1 | 0.002321136 | -0.06906 | 0.1 | 0.152 | 1 | 0 |
| Jakmip1 | 0.002329107 | -0.03161 | 0.097 | 0.149 | 1 | 0 |
| Kif5b | 0.002339128 | -0.07151 | 0.312 | 0.421 | 1 | 0 |
| Cyb5r4 | 0.002348582 | -0.02865 | 0.081 | 0.129 | 1 | 0 |
| Gabpa | 0.002349545 | -0.01756 | 0.065 | 0.109 | 1 | 0 |
| Vps13a | 0.002357645 | -0.03643 | 0.158 | 0.23 | 1 | 0 |
| Tmem234 | 0.002359643 | -0.00662 | 0.315 | 0.436 | 1 | 0 |
| Mllt6 | 0.00237079 | -0.03252 | 0.1 | 0.154 | 1 | 0 |
| Spg21 | 0.002375258 | 0.014125 | 0.11 | 0.169 | 1 | 0 |
| Dot1l | 0.002387718 | -0.01421 | 0.073 | 0.118 | 1 | 0 |
| Trim5 | 0.002398973 | -0.02149 | 0.093 | 0.143 | 1 | 0 |
| Rab5a | 0.002408499 | 0.000865 | 0.11 | 0.168 | 1 | 0 |
| Pon2 | 0.00242241 | -0.00703 | 0.118 | 0.177 | 1 | 0 |
| Dph5 | 0.002425302 | -0.04824 | 0.064 | 0.105 | 1 | 0 |
| Med1 | 0.002449992 | -0.01239 | 0.15 | 0.22 | 1 | 0 |
| Retreg1 | 0.002455768 | -0.01002 | 0.191 | 0.273 | 1 | 0 |
| Snrpe | 0.002456119 | -0.07404 | 0.481 | 0.605 | 1 | 0 |
| Calm2 | 0.0024591 | 0.177541 | 0.588 | 0.61 | 1 | 0 |
| Myo9b | 0.002460838 | -0.03965 | 0.066 | 0.109 | 1 | 0 |
| Rpl10-ps3 | 0.002469007 | -0.03376 | 0.268 | 0.374 | 1 | 0 |
| Zmiz1 | 0.002476484 | 0.002993 | 0.146 | 0.215 | 1 | 0 |
| Fam204a | 0.002478867 | 0.001845 | 0.187 | 0.268 | 1 | 0 |
| Arf3 | 0.002485559 | -0.03356 | 0.075 | 0.121 | 1 | 0 |
| Psmd4 | 0.002487276 | -0.00978 | 0.24 | 0.34 | 1 | 0 |
| Glo1 | 0.002501011 | 0.000215 | 0.065 | 0.109 | 1 | 0 |
| Pnn | 0.002503128 | -0.0261 | 0.3 | 0.409 | 1 | 0 |
| Sel1l | 0.002507996 | -0.01936 | 0.075 | 0.121 | 1 | 0 |
| Parl | 0.002510397 | -0.00186 | 0.074 | 0.119 | 1 | 0 |
| Ddx50 | 0.002512155 | -0.02473 | 0.314 | 0.428 | 1 | 0 |
| Sidt2 | 0.002514777 | -0.00059 | 0.131 | 0.196 | 1 | 0 |
| Kri1 | 0.002517304 | -0.02389 | 0.072 | 0.117 | 1 | 0 |
| Tacc1 | 0.002517958 | -0.02841 | 0.239 | 0.333 | 1 | 0 |
| Rictor | 0.002533949 | -0.02859 | 0.194 | 0.273 | 1 | 0 |
| Card6 | 0.00253858 | -0.07076 | 0.094 | 0.143 | 1 | 0 |
| Cast | 0.00255369 | -0.03649 | 0.212 | 0.298 | 1 | 0 |
| Adam10 | 0.002559465 | -0.0134 | 0.119 | 0.179 | 1 | 0 |
| Atf2 | 0.002591842 | -0.01583 | 0.104 | 0.159 | 1 | 0 |
| Nasp | 0.002592087 | -0.03755 | 0.243 | 0.335 | 1 | 0 |
| H2afj | 0.002601894 | -0.04322 | 0.326 | 0.436 | 1 | 0 |
| Uba1 | 0.002614245 | -0.00044 | 0.121 | 0.181 | 1 | 0 |
| Rela | 0.002620825 | 0.015471 | 0.109 | 0.167 | 1 | 0 |
| Ubap2l | 0.002621552 | -0.04318 | 0.335 | 0.457 | 1 | 0 |
| Plekhf2 | 0.00262661 | 0.003319 | 0.086 | 0.136 | 1 | 0 |
| Coa5 | 0.002650353 | -0.03088 | 0.063 | 0.104 | 1 | 0 |
| Copz1 | 0.002658063 | -0.02872 | 0.083 | 0.13 | 1 | 0 |
| Hif1a | 0.002658603 | -0.01584 | 0.158 | 0.229 | 1 | 0 |
| Dlg1 | 0.002663584 | -0.04093 | 0.081 | 0.129 | 1 | 0 |
| Anp32e | 0.002665026 | -0.01435 | 0.21 | 0.301 | 1 | 0 |
| Mcrs1 | 0.002679831 | -0.00243 | 0.065 | 0.107 | 1 | 0 |
| Tigd2 | 0.002702074 | -0.03736 | 0.107 | 0.16 | 1 | 0 |
| Iqgap1 | 0.002702211 | -0.07278 | 0.577 | 0.718 | 1 | 0 |
| D1Ertd622e | 0.002703512 | 0.009611 | 0.127 | 0.19 | 1 | 0 |
| Knop1 | 0.002721226 | -0.02077 | 0.065 | 0.107 | 1 | 0 |
| Gm26669 | 0.002728825 | 0.205501 | 0.123 | 0.085 | 1 | 0 |
| Smarcd1 | 0.002730426 | -0.03493 | 0.065 | 0.106 | 1 | 0 |
| Avl9 | 0.002732706 | -0.02656 | 0.249 | 0.347 | 1 | 0 |
| Selenof | 0.002737898 | -0.02923 | 0.276 | 0.385 | 1 | 0 |
| Ahctf1 | 0.002738382 | -0.02663 | 0.149 | 0.215 | 1 | 0 |
| Fam120a | 0.00275164 | -0.00503 | 0.099 | 0.153 | 1 | 0 |
| Sap30l | 0.002760399 | -0.01564 | 0.065 | 0.106 | 1 | 0 |
| Usp48 | 0.002774333 | -0.0401 | 0.073 | 0.117 | 1 | 0 |
| Cebpg | 0.002791932 | -0.0233 | 0.112 | 0.168 | 1 | 0 |
| Arl6ip5 | 0.002799949 | -0.00087 | 0.109 | 0.167 | 1 | 0 |
| Psmb4 | 0.002801252 | -0.04002 | 0.256 | 0.356 | 1 | 0 |
| Cd274 | 0.00280516 | -0.03789 | 0.074 | 0.118 | 1 | 0 |
| Hspa14 | 0.002806397 | 0.010469 | 0.112 | 0.171 | 1 | 0 |
| Arpc4 | 0.002814019 | -0.01341 | 0.268 | 0.378 | 1 | 0 |
| Tet3 | 0.002825272 | -0.02705 | 0.108 | 0.162 | 1 | 0 |
| Alkbh1 | 0.002837865 | -0.02279 | 0.166 | 0.235 | 1 | 0 |
| Psmc6 | 0.00284488 | 0.004666 | 0.157 | 0.229 | 1 | 0 |
| Ywhae | 0.002868888 | 0.011734 | 0.326 | 0.452 | 1 | 0 |
| Gapvd1 | 0.002869221 | -0.02433 | 0.14 | 0.205 | 1 | 0 |
| March6 | 0.002874606 | -0.03044 | 0.069 | 0.112 | 1 | 0 |
| Rbbp6 | 0.002889012 | -0.01374 | 0.366 | 0.501 | 1 | 0 |
| Wdr26 | 0.002905563 | -0.0064 | 0.242 | 0.338 | 1 | 0 |
| Wdr82 | 0.002914652 | -0.01618 | 0.092 | 0.142 | 1 | 0 |
| Itch | 0.002915319 | -0.02134 | 0.232 | 0.328 | 1 | 0 |
| Rnf6 | 0.002919094 | -0.04006 | 0.12 | 0.175 | 1 | 0 |
| Araf | 0.002934688 | -0.0396 | 0.086 | 0.134 | 1 | 0 |
| Cuedc2 | 0.002957693 | 0.025779 | 0.095 | 0.148 | 1 | 0 |
| Pik3c2a | 0.002966967 | -0.03059 | 0.108 | 0.161 | 1 | 0 |
| Rad23b | 0.002981707 | -0.01764 | 0.149 | 0.216 | 1 | 0 |
| Glrx2 | 0.00299753 | -0.0098 | 0.068 | 0.111 | 1 | 0 |
| Map2k7 | 0.003009654 | -0.04858 | 0.064 | 0.104 | 1 | 0 |
| Ilf2 | 0.003011782 | 0.000943 | 0.162 | 0.234 | 1 | 0 |
| Trim24 | 0.003020057 | -0.04467 | 0.113 | 0.168 | 1 | 0 |
| Lamtor2 | 0.00303829 | 0.007392 | 0.266 | 0.378 | 1 | 0 |
| Fcho2 | 0.003059698 | 0.00116 | 0.115 | 0.173 | 1 | 0 |
| Gnai2 | 0.003062422 | -0.08804 | 0.581 | 0.721 | 1 | 0 |
| Plpbp | 0.003064825 | -0.01054 | 0.09 | 0.14 | 1 | 0 |
| Laptm5 | 0.003069677 | 0.129577 | 0.723 | 0.743 | 1 | 0 |
| Tle4 | 0.003101968 | -0.01702 | 0.185 | 0.261 | 1 | 0 |
| Pim3 | 0.003121508 | -0.02509 | 0.104 | 0.158 | 1 | 0 |
| Birc3 | 0.003123499 | -0.02258 | 0.335 | 0.456 | 1 | 0 |
| Gatad1 | 0.003126494 | -0.04093 | 0.069 | 0.111 | 1 | 0 |
| Tbl1x | 0.003128776 | 0.01613 | 0.094 | 0.146 | 1 | 0 |
| Cds2 | 0.003134655 | -0.02223 | 0.071 | 0.115 | 1 | 0 |
| AI837181 | 0.00313509 | -0.024 | 0.07 | 0.113 | 1 | 0 |
| Rad17 | 0.003135248 | -0.04603 | 0.076 | 0.119 | 1 | 0 |
| Fuca1 | 0.003173278 | 0.007933 | 0.171 | 0.245 | 1 | 0 |
| Pcsk7 | 0.00317493 | -0.00988 | 0.07 | 0.113 | 1 | 0 |
| Gimap9 | 0.003190619 | -0.0764 | 0.149 | 0.209 | 1 | 0 |
| Ogfr | 0.00320372 | -0.02571 | 0.077 | 0.122 | 1 | 0 |
| Slc38a1 | 0.003208452 | -0.06376 | 0.36 | 0.47 | 1 | 0 |
| Paf1 | 0.003216604 | -0.04667 | 0.097 | 0.147 | 1 | 0 |
| Ublcp1 | 0.003217017 | -0.03309 | 0.101 | 0.153 | 1 | 0 |
| Ndufs2 | 0.003220029 | 0.000134 | 0.136 | 0.2 | 1 | 0 |
| Sh3kbp1 | 0.003252769 | -0.03361 | 0.204 | 0.282 | 1 | 0 |
| Usp4 | 0.003258598 | -0.02206 | 0.08 | 0.126 | 1 | 0 |
| Zfp451 | 0.003264513 | -0.00574 | 0.08 | 0.126 | 1 | 0 |
| Mpc2 | 0.003281863 | 0.009054 | 0.182 | 0.26 | 1 | 0 |
| Mfsd14a | 0.003353808 | 0.012639 | 0.105 | 0.16 | 1 | 0 |
| Sp1 | 0.003360901 | -0.02329 | 0.129 | 0.189 | 1 | 0 |
| Tox4 | 0.003373478 | 0.002446 | 0.147 | 0.216 | 1 | 0 |
| Churc1 | 0.003391252 | -0.004 | 0.152 | 0.221 | 1 | 0 |
| Anxa6 | 0.003400692 | -0.01038 | 0.129 | 0.189 | 1 | 0 |
| Psen2 | 0.003420832 | -0.02383 | 0.089 | 0.137 | 1 | 0 |
| Ahcyl2 | 0.003426954 | -0.01522 | 0.083 | 0.129 | 1 | 0 |
| Clec2d | 0.003443354 | -0.00397 | 0.164 | 0.233 | 1 | 0 |
| Tnpo1 | 0.003443966 | -0.00913 | 0.143 | 0.208 | 1 | 0 |
| Sirt1 | 0.003456474 | -0.02109 | 0.117 | 0.174 | 1 | 0 |
| Snx4 | 0.00348429 | -0.01905 | 0.065 | 0.106 | 1 | 0 |
| A530032D15Rik | 0.003515794 | -0.02068 | 0.062 | 0.101 | 1 | 0 |
| Smarcd2 | 0.003518612 | -0.01008 | 0.109 | 0.163 | 1 | 0 |
| Exosc7 | 0.003579208 | -0.0305 | 0.066 | 0.107 | 1 | 0 |
| Tex10 | 0.003586274 | -0.01989 | 0.063 | 0.103 | 1 | 0 |
| Chd2 | 0.003586294 | -0.0394 | 0.309 | 0.416 | 1 | 0 |
| Tcf12 | 0.003592771 | -0.03967 | 0.231 | 0.319 | 1 | 0 |
| Cyth2 | 0.003593264 | -0.01378 | 0.09 | 0.14 | 1 | 0 |
| Map4k2 | 0.003602337 | -0.02975 | 0.162 | 0.233 | 1 | 0 |
| Aes | 0.003602521 | -0.04697 | 0.379 | 0.513 | 1 | 0 |
| Epn1 | 0.003625547 | -0.00359 | 0.161 | 0.234 | 1 | 0 |
| Pcmtd2 | 0.003628427 | -0.04483 | 0.08 | 0.124 | 1 | 0 |
| Rbbp7 | 0.003636402 | -0.00478 | 0.144 | 0.209 | 1 | 0 |
| Elovl1 | 0.00365206 | -0.00907 | 0.065 | 0.106 | 1 | 0 |
| Bloc1s2 | 0.003659693 | -0.00545 | 0.08 | 0.125 | 1 | 0 |
| Bst2 | 0.003660288 | 0.025438 | 0.144 | 0.208 | 1 | 0 |
| Cpsf3 | 0.00366125 | -0.00697 | 0.065 | 0.105 | 1 | 0 |
| Dnaja2 | 0.003668657 | -0.0134 | 0.239 | 0.331 | 1 | 0 |
| Vma21 | 0.003699648 | -0.02299 | 0.071 | 0.113 | 1 | 0 |
| Nsf | 0.003720887 | -0.03878 | 0.205 | 0.283 | 1 | 0 |
| H2-DMb2 | 0.003721539 | -0.06952 | 0.544 | 0.673 | 1 | 0 |
| Snrnp25 | 0.003725654 | -0.04482 | 0.086 | 0.131 | 1 | 0 |
| Gng2 | 0.003736329 | -0.02711 | 0.202 | 0.283 | 1 | 0 |
| Tbk1 | 0.003739114 | -0.0152 | 0.1 | 0.152 | 1 | 0 |
| Spcs3 | 0.003756166 | -0.0374 | 0.1 | 0.15 | 1 | 0 |
| Gatad2b | 0.003768618 | -0.00567 | 0.269 | 0.364 | 1 | 0 |
| Mrpl24 | 0.003769672 | 0.030305 | 0.205 | 0.29 | 1 | 0 |
| Slc38a9 | 0.003779509 | -0.02828 | 0.109 | 0.161 | 1 | 0 |
| Psmd11 | 0.003782286 | -0.00011 | 0.172 | 0.247 | 1 | 0 |
| Kif2a | 0.003787238 | 0.001354 | 0.13 | 0.19 | 1 | 0 |
| Cdk8 | 0.003804507 | -0.0185 | 0.066 | 0.107 | 1 | 0 |
| Sin3a | 0.00382192 | -0.02833 | 0.075 | 0.118 | 1 | 0 |
| Sec23b | 0.003825189 | -0.01667 | 0.062 | 0.101 | 1 | 0 |
| Tma7 | 0.003826949 | -0.05088 | 0.512 | 0.666 | 1 | 0 |
| Gpr183 | 0.003844706 | 0.271879 | 0.27 | 0.237 | 1 | 0 |
| Tmem55b | 0.003856377 | -0.05272 | 0.181 | 0.253 | 1 | 0 |
| Sept1 | 0.003860852 | -0.00106 | 0.277 | 0.389 | 1 | 0 |
| Zfp593 | 0.00386638 | -0.01308 | 0.063 | 0.103 | 1 | 0 |
| Rpl15 | 0.003883423 | 0.05385 | 0.987 | 0.989 | 1 | 0 |
| Tmem208 | 0.003898311 | -0.02616 | 0.114 | 0.169 | 1 | 0 |
| Ube2j2 | 0.00391942 | 0.008897 | 0.116 | 0.174 | 1 | 0 |
| Phc3 | 0.003923598 | -0.01082 | 0.083 | 0.129 | 1 | 0 |
| Hist1h2bc | 0.003927878 | -0.02806 | 0.072 | 0.115 | 1 | 0 |
| Asf1a | 0.003929908 | -0.01122 | 0.064 | 0.104 | 1 | 0 |
| Ubc | 0.003934445 | 0.118793 | 0.871 | 0.888 | 1 | 0 |
| Cnot6 | 0.003952023 | -0.00579 | 0.136 | 0.197 | 1 | 0 |
| Ifitm2 | 0.003956852 | 0.206791 | 0.164 | 0.123 | 1 | 0 |
| Psmc3 | 0.003960094 | -0.01448 | 0.17 | 0.243 | 1 | 0 |
| Prdx3 | 0.003980564 | 0.010197 | 0.075 | 0.119 | 1 | 0 |
| BC005561 | 0.004008982 | -0.02794 | 0.065 | 0.104 | 1 | 0 |
| Cdipt | 0.00402231 | -0.02963 | 0.067 | 0.107 | 1 | 0 |
| Trp53i11 | 0.004028022 | -0.01672 | 0.224 | 0.308 | 1 | 0 |
| Otud4 | 0.004044754 | -0.00735 | 0.089 | 0.137 | 1 | 0 |
| Gimap3 | 0.004048175 | -0.02688 | 0.268 | 0.363 | 1 | 0 |
| Lyar | 0.004049656 | -0.04356 | 0.094 | 0.141 | 1 | 0 |
| Ndfip2 | 0.00406966 | 0.015874 | 0.104 | 0.158 | 1 | 0 |
| Ufm1 | 0.004078666 | -0.0115 | 0.129 | 0.19 | 1 | 0 |
| Bcap31 | 0.004086616 | -0.02541 | 0.267 | 0.365 | 1 | 0 |
| Srpk3 | 0.004092284 | -0.0436 | 0.074 | 0.116 | 1 | 0 |
| Cnpy3 | 0.004126907 | 0.008317 | 0.118 | 0.174 | 1 | 0 |
| Cdkn2aip | 0.004128736 | -0.01523 | 0.091 | 0.138 | 1 | 0 |
| Rtf2 | 0.004133049 | -0.03229 | 0.102 | 0.153 | 1 | 0 |
| Cmtm6 | 0.004146862 | 0.000536 | 0.12 | 0.179 | 1 | 0 |
| Pom121 | 0.00416338 | -0.01708 | 0.093 | 0.141 | 1 | 0 |
| Vps36 | 0.004164308 | -0.00166 | 0.113 | 0.168 | 1 | 0 |
| Rnf216 | 0.004221519 | -0.03559 | 0.155 | 0.22 | 1 | 0 |
| Arid2 | 0.004259685 | -0.00739 | 0.094 | 0.142 | 1 | 0 |
| Pip5k1a | 0.004282069 | 0.005305 | 0.078 | 0.122 | 1 | 0 |
| Nmt2 | 0.004299608 | -0.0392 | 0.066 | 0.106 | 1 | 0 |
| Bag6 | 0.004314864 | 0.006507 | 0.075 | 0.119 | 1 | 0 |
| Hpf1 | 0.004324982 | -0.00018 | 0.091 | 0.14 | 1 | 0 |
| Zmiz2 | 0.004336905 | -0.03082 | 0.147 | 0.21 | 1 | 0 |
| Sar1b | 0.004339603 | 0.0135 | 0.093 | 0.143 | 1 | 0 |
| Atg5 | 0.00434527 | -0.02738 | 0.072 | 0.113 | 1 | 0 |
| Icam1 | 0.004358186 | 0.004715 | 0.112 | 0.165 | 1 | 0 |
| Arhgap45 | 0.004372628 | -0.0103 | 0.346 | 0.464 | 1 | 0 |
| Atp5c1 | 0.004410682 | -0.03506 | 0.34 | 0.459 | 1 | 0 |
| Dad1 | 0.004411093 | 0.153641 | 0.625 | 0.671 | 1 | 0 |
| Bcl2l11 | 0.004431188 | -0.05067 | 0.163 | 0.226 | 1 | 0 |
| Rpl7l1 | 0.004444105 | -0.0245 | 0.114 | 0.167 | 1 | 0 |
| Timm13 | 0.004467864 | -0.05155 | 0.236 | 0.326 | 1 | 0 |
| Yipf1 | 0.004498195 | -0.03565 | 0.071 | 0.112 | 1 | 0 |
| Tiprl | 0.004509949 | -0.01418 | 0.089 | 0.136 | 1 | 0 |
| D16Ertd472e | 0.004511846 | -0.0207 | 0.066 | 0.106 | 1 | 0 |
| Ifngr2 | 0.004536775 | -0.05202 | 0.115 | 0.166 | 1 | 0 |
| Pias4 | 0.004562631 | -0.03268 | 0.066 | 0.106 | 1 | 0 |
| Tmem57 | 0.004564539 | 0.021837 | 0.096 | 0.147 | 1 | 0 |
| Rae1 | 0.00458924 | 0.003738 | 0.072 | 0.115 | 1 | 0 |
| Tsg101 | 0.004596387 | 0.001548 | 0.08 | 0.124 | 1 | 0 |
| Ppp1r21 | 0.004606841 | -0.01701 | 0.205 | 0.282 | 1 | 0 |
| Cpeb4 | 0.004758693 | -0.03341 | 0.078 | 0.121 | 1 | 0 |
| Sqstm1 | 0.004764986 | -0.01457 | 0.209 | 0.29 | 1 | 0 |
| Rpl36a-ps1 | 0.004791694 | -0.02209 | 0.07 | 0.111 | 1 | 0 |
| Ostf1 | 0.004792757 | -0.03082 | 0.31 | 0.419 | 1 | 0 |
| Rbm18 | 0.004808739 | -0.03128 | 0.071 | 0.111 | 1 | 0 |
| Sde2 | 0.004810674 | 0.001463 | 0.113 | 0.168 | 1 | 0 |
| Btf3 | 0.004819867 | 0.106379 | 0.788 | 0.82 | 1 | 0 |
| Nfkbie | 0.004821814 | -0.01029 | 0.099 | 0.148 | 1 | 0 |
| Hif1an | 0.004841818 | -0.01166 | 0.09 | 0.137 | 1 | 0 |
| Nedd8 | 0.004850638 | -0.04207 | 0.331 | 0.451 | 1 | 0 |
| Sik3 | 0.004872999 | -0.02896 | 0.064 | 0.101 | 1 | 0 |
| Chmp3 | 0.004889087 | 0.005559 | 0.106 | 0.159 | 1 | 0 |
| Pank3 | 0.004891157 | -0.04866 | 0.065 | 0.104 | 1 | 0 |
| Ran | 0.004896571 | -0.06227 | 0.375 | 0.492 | 1 | 0 |
| Vdac3 | 0.00491902 | 0.014704 | 0.135 | 0.196 | 1 | 0 |
| Gse1 | 0.004965207 | -0.02043 | 0.072 | 0.113 | 1 | 0 |
| Tmem11 | 0.0049898 | -0.02984 | 0.084 | 0.129 | 1 | 0 |
| Crebzf | 0.005003247 | 0.003069 | 0.094 | 0.143 | 1 | 0 |
| Cyth1 | 0.005058103 | -0.01849 | 0.296 | 0.403 | 1 | 0 |
| Lamp2 | 0.005060438 | 0.251826 | 0.265 | 0.239 | 1 | 0 |
| Tkt | 0.005061865 | -0.02957 | 0.218 | 0.301 | 1 | 0 |
| Fcf1 | 0.005072399 | 0.003067 | 0.131 | 0.191 | 1 | 0 |
| Cab39l | 0.005083588 | -0.01773 | 0.083 | 0.128 | 1 | 0 |
| Pck2 | 0.005088468 | -0.01552 | 0.08 | 0.124 | 1 | 0 |
| Mlxip | 0.005106407 | -0.01726 | 0.18 | 0.252 | 1 | 0 |
| Kpna1 | 0.005110914 | -0.00624 | 0.138 | 0.198 | 1 | 0 |
| Usp6nl | 0.005131529 | -0.0182 | 0.072 | 0.113 | 1 | 0 |
| Arl6ip4 | 0.00514251 | -0.02211 | 0.086 | 0.13 | 1 | 0 |
| Irf1 | 0.005169205 | -0.0546 | 0.249 | 0.329 | 1 | 0 |
| Sart1 | 0.005182493 | 0.006798 | 0.097 | 0.147 | 1 | 0 |
| Rnf149 | 0.005195254 | 0.232152 | 0.147 | 0.112 | 1 | 0 |
| Mob1b | 0.005250468 | 0.025916 | 0.078 | 0.122 | 1 | 0 |
| Adh5 | 0.005301884 | -0.0114 | 0.115 | 0.168 | 1 | 0 |
| Crlf2 | 0.005308089 | -0.00817 | 0.188 | 0.263 | 1 | 0 |
| Casc3 | 0.005333311 | -0.00471 | 0.072 | 0.113 | 1 | 0 |
| Zfp280d | 0.005367641 | -0.01744 | 0.126 | 0.183 | 1 | 0 |
| Pbrm1 | 0.005394446 | -0.01869 | 0.261 | 0.348 | 1 | 0 |
| Nus1 | 0.005402069 | -0.03318 | 0.095 | 0.142 | 1 | 0 |
| Kansl1 | 0.005413643 | -0.019 | 0.222 | 0.305 | 1 | 0 |
| Mdh1 | 0.005414519 | -0.00425 | 0.234 | 0.325 | 1 | 0 |
| Taf13 | 0.00541841 | -0.03398 | 0.087 | 0.131 | 1 | 0 |
| Chmp4b | 0.005446706 | -0.007 | 0.227 | 0.319 | 1 | 0 |
| Mrps9 | 0.005450585 | -0.00982 | 0.084 | 0.129 | 1 | 0 |
| Mrps12 | 0.005485436 | 0.024844 | 0.065 | 0.104 | 1 | 0 |
| Ago2 | 0.005529934 | -0.01798 | 0.244 | 0.334 | 1 | 0 |
| Fam60a | 0.005542607 | -0.02276 | 0.071 | 0.111 | 1 | 0 |
| Nom1 | 0.005547469 | -0.03004 | 0.075 | 0.116 | 1 | 0 |
| Ugcg | 0.00555651 | -0.00276 | 0.172 | 0.241 | 1 | 0 |
| Ddi2 | 0.00556291 | -0.02118 | 0.071 | 0.111 | 1 | 0 |
| Naa20 | 0.005577051 | -0.01593 | 0.065 | 0.104 | 1 | 0 |
| Rab30 | 0.005579083 | -0.04277 | 0.08 | 0.122 | 1 | 0 |
| Hipk3 | 0.00559654 | 0.008846 | 0.073 | 0.115 | 1 | 0 |
| Fkbp5 | 0.005602158 | -0.01265 | 0.109 | 0.16 | 1 | 0 |
| Elof1 | 0.005604945 | -0.01619 | 0.095 | 0.143 | 1 | 0 |
| Bmyc | 0.005613079 | -0.01523 | 0.085 | 0.129 | 1 | 0 |
| Sft2d1 | 0.005622263 | 0.000219 | 0.144 | 0.205 | 1 | 0 |
| Ets2 | 0.00562685 | -0.0276 | 0.123 | 0.178 | 1 | 0 |
| Aldoa | 0.005674663 | 0.194356 | 0.465 | 0.488 | 1 | 0 |
| Bfar | 0.005710224 | -0.03796 | 0.114 | 0.166 | 1 | 0 |
| Dtx1 | 0.005716832 | -0.0091 | 0.101 | 0.15 | 1 | 0 |
| Lgals8 | 0.005752396 | -0.031 | 0.098 | 0.146 | 1 | 0 |
| Ndufc2 | 0.00576625 | -0.00539 | 0.165 | 0.234 | 1 | 0 |
| Rab35 | 0.005798977 | -0.05333 | 0.08 | 0.121 | 1 | 0 |
| Hilpda | 0.005825653 | 0.224076 | 0.169 | 0.134 | 1 | 0 |
| H2-Ke6 | 0.005841605 | 0.015925 | 0.085 | 0.131 | 1 | 0 |
| Srek1 | 0.005907565 | -0.00735 | 0.123 | 0.179 | 1 | 0 |
| Prkag1 | 0.005974178 | -0.01816 | 0.066 | 0.105 | 1 | 0 |
| Med13l | 0.006006268 | 0.001602 | 0.147 | 0.21 | 1 | 0 |
| Rpl18 | 0.006025788 | 0.041734 | 0.997 | 0.996 | 1 | 0 |
| Zcchc17 | 0.006124772 | -0.0254 | 0.106 | 0.155 | 1 | 0 |
| Rc3h1 | 0.006177712 | 0.004605 | 0.134 | 0.193 | 1 | 0 |
| Cycs | 0.006197669 | -0.02555 | 0.256 | 0.345 | 1 | 0 |
| Ogdh | 0.006215076 | 0.015229 | 0.185 | 0.261 | 1 | 0 |
| Ndufaf8 | 0.006235614 | -0.00818 | 0.107 | 0.158 | 1 | 0 |
| Abhd17b | 0.006254271 | 0.008107 | 0.199 | 0.28 | 1 | 0 |
| Krtcap2 | 0.006306857 | -0.04658 | 0.327 | 0.43 | 1 | 0 |
| Dennd5a | 0.006331569 | -0.01079 | 0.083 | 0.126 | 1 | 0 |
| Vasp | 0.00635606 | -0.03622 | 0.295 | 0.388 | 1 | 0 |
| Ndufa4 | 0.006383211 | -0.04556 | 0.411 | 0.531 | 1 | 0 |
| Myo1c | 0.006386536 | 0.009341 | 0.163 | 0.23 | 1 | 0 |
| Rpp21 | 0.006425053 | -0.03042 | 0.125 | 0.179 | 1 | 0 |
| Zfp445 | 0.006429909 | -0.02614 | 0.091 | 0.135 | 1 | 0 |
| Qrich1 | 0.006433706 | 0.008511 | 0.106 | 0.156 | 1 | 0 |
| Exosc8 | 0.006447054 | -0.03112 | 0.101 | 0.149 | 1 | 0 |
| Rtcb | 0.006494968 | -0.01576 | 0.174 | 0.243 | 1 | 0 |
| Tfrc | 0.00654771 | -0.03939 | 0.084 | 0.126 | 1 | 0 |
| Brd2 | 0.006552186 | -0.01912 | 0.383 | 0.508 | 1 | 0 |
| Gm15987 | 0.006556681 | -0.03898 | 0.162 | 0.226 | 1 | 0 |
| Tuba1b | 0.00659051 | -0.06666 | 0.299 | 0.39 | 1 | 0 |
| Qk | 0.00666487 | 0.024072 | 0.232 | 0.32 | 1 | 0 |
| Clock | 0.006684528 | -0.00759 | 0.067 | 0.106 | 1 | 0 |
| Hexim1 | 0.006727584 | -0.01633 | 0.209 | 0.282 | 1 | 0 |
| Fbl | 0.00673297 | -0.03329 | 0.223 | 0.301 | 1 | 0 |
| Il4ra | 0.006741744 | 0.021854 | 0.167 | 0.234 | 1 | 0 |
| Dnajb1 | 0.006761287 | 0.219499 | 0.36 | 0.347 | 1 | 0 |
| S100a10 | 0.006807123 | -0.10684 | 0.457 | 0.57 | 1 | 0 |
| Ythdf2 | 0.006833766 | -0.00444 | 0.093 | 0.14 | 1 | 0 |
| Tmem106b | 0.006872404 | -0.04807 | 0.073 | 0.112 | 1 | 0 |
| AI504432 | 0.006887799 | -0.03576 | 0.079 | 0.119 | 1 | 0 |
| Pafah1b3 | 0.006888718 | -0.01786 | 0.126 | 0.179 | 1 | 0 |
| Mepce | 0.006893667 | 0.037531 | 0.156 | 0.224 | 1 | 0 |
| Skp1a | 0.006916369 | 0.008827 | 0.126 | 0.183 | 1 | 0 |
| Zfp746 | 0.006954179 | -0.05267 | 0.07 | 0.109 | 1 | 0 |
| Sms | 0.006978298 | 0.013508 | 0.167 | 0.237 | 1 | 0 |
| Tmem163 | 0.007016701 | -0.02396 | 0.074 | 0.113 | 1 | 0 |
| Azi2 | 0.007050748 | -0.00283 | 0.095 | 0.143 | 1 | 0 |
| Pwwp2a | 0.007055923 | -0.01282 | 0.094 | 0.141 | 1 | 0 |
| Rgs2 | 0.007166198 | -0.10986 | 0.289 | 0.366 | 1 | 0 |
| Chmp2b | 0.007189341 | -0.02388 | 0.071 | 0.11 | 1 | 0 |
| Cr1l | 0.007225233 | 0.01727 | 0.209 | 0.289 | 1 | 0 |
| Fam208a | 0.007237683 | 0.023886 | 0.224 | 0.307 | 1 | 0 |
| Sub1 | 0.00723811 | 0.103585 | 0.807 | 0.832 | 1 | 0 |
| Wfdc21 | 0.007238807 | 0.205544 | 0.273 | 0.229 | 1 | 0 |
| Ccnd3 | 0.007319754 | 0.034368 | 0.247 | 0.338 | 1 | 0 |
| Wbp4 | 0.007342384 | -0.02148 | 0.168 | 0.233 | 1 | 0 |
| Clns1a | 0.007373536 | -0.01811 | 0.079 | 0.119 | 1 | 0 |
| Hnrnpab | 0.007374351 | -0.01978 | 0.386 | 0.511 | 1 | 0 |
| Crebrf | 0.007435481 | -0.05126 | 0.298 | 0.389 | 1 | 0 |
| Ppm1d | 0.007437891 | -0.00889 | 0.073 | 0.112 | 1 | 0 |
| Mirt1 | 0.007444381 | -0.02261 | 0.136 | 0.191 | 1 | 0 |
| Rps9 | 0.007450856 | 0.042897 | 1 | 0.998 | 1 | 0 |
| Atxn2l | 0.007501803 | 0.015911 | 0.193 | 0.268 | 1 | 0 |
| Mrpl54 | 0.007506631 | 0.016456 | 0.138 | 0.197 | 1 | 0 |
| Plgrkt | 0.007539334 | -0.02737 | 0.171 | 0.235 | 1 | 0 |
| Usp47 | 0.007547331 | -0.0188 | 0.081 | 0.123 | 1 | 0 |
| Ncoa6 | 0.007560777 | -0.0256 | 0.066 | 0.104 | 1 | 0 |
| Ahsa1 | 0.007571181 | 0.253464 | 0.281 | 0.261 | 1 | 0 |
| Mrgbp | 0.007625526 | -0.04072 | 0.066 | 0.103 | 1 | 0 |
| Cbll1 | 0.007653609 | -0.0079 | 0.071 | 0.11 | 1 | 0 |
| Lamtor4 | 0.007672149 | 0.025252 | 0.128 | 0.186 | 1 | 0 |
| Lsm2 | 0.007700985 | -0.01276 | 0.086 | 0.129 | 1 | 0 |
| Cope | 0.007707711 | -0.02184 | 0.16 | 0.223 | 1 | 0 |
| Frat2 | 0.007753426 | -0.06833 | 0.079 | 0.118 | 1 | 0 |
| Sri | 0.007787313 | -0.00489 | 0.277 | 0.381 | 1 | 0 |
| Top1 | 0.007809245 | -0.05925 | 0.402 | 0.512 | 1 | 0 |
| Dda1 | 0.007809846 | -0.00316 | 0.123 | 0.178 | 1 | 0 |
| Klhl6 | 0.007822011 | -0.05036 | 0.163 | 0.223 | 1 | 0 |
| Diablo | 0.007835825 | -0.00428 | 0.074 | 0.113 | 1 | 0 |
| Tmcc1 | 0.007886118 | -0.02568 | 0.099 | 0.144 | 1 | 0 |
| Tpd52l2 | 0.007915827 | -0.0061 | 0.095 | 0.141 | 1 | 0 |
| Kctd12 | 0.007918127 | 0.005494 | 0.317 | 0.424 | 1 | 0 |
| Btbd1 | 0.007926405 | 0.015829 | 0.118 | 0.172 | 1 | 0 |
| Nup153 | 0.007992974 | -0.0094 | 0.101 | 0.148 | 1 | 0 |
| Ptprcap | 0.008119932 | -0.03538 | 0.525 | 0.644 | 1 | 0 |
| Emc7 | 0.008168603 | 0.002285 | 0.139 | 0.198 | 1 | 0 |
| Utp11 | 0.008191686 | 0.00154 | 0.098 | 0.144 | 1 | 0 |
| Crtc2 | 0.008248906 | -0.01756 | 0.07 | 0.109 | 1 | 0 |
| Timm8b | 0.008254021 | 0.006002 | 0.118 | 0.169 | 1 | 0 |
| Ddx3x | 0.0082556 | -0.02438 | 0.378 | 0.498 | 1 | 0 |
| Ankrd12 | 0.008262428 | -0.01209 | 0.259 | 0.341 | 1 | 0 |
| Hp | 0.008276865 | 0.194931 | 0.212 | 0.173 | 1 | 0 |
| Ppp4r2 | 0.008323734 | 0.000603 | 0.171 | 0.237 | 1 | 0 |
| 0610012G03Rik | 0.008324938 | 0.022806 | 0.105 | 0.154 | 1 | 0 |
| Rbm33 | 0.008376024 | 0.010038 | 0.154 | 0.218 | 1 | 0 |
| Dmtf1 | 0.008391054 | -0.0023 | 0.112 | 0.162 | 1 | 0 |
| Ppp6r3 | 0.008414879 | 0.006309 | 0.181 | 0.251 | 1 | 0 |
| Iscu | 0.008451728 | -0.00901 | 0.103 | 0.15 | 1 | 0 |
| Psma3 | 0.008520248 | -0.01659 | 0.423 | 0.557 | 1 | 0 |
| Mgrn1 | 0.008528159 | 0.009598 | 0.096 | 0.143 | 1 | 0 |
| Arnt | 0.008551666 | -0.02193 | 0.085 | 0.128 | 1 | 0 |
| Prkx | 0.008560936 | 0.000936 | 0.073 | 0.112 | 1 | 0 |
| Sae1 | 0.008566079 | -0.0056 | 0.095 | 0.141 | 1 | 0 |
| H2-Q6 | 0.008573419 | 0.239927 | 0.401 | 0.403 | 1 | 0 |
| Mthfsl | 0.008590683 | 0.002155 | 0.072 | 0.111 | 1 | 0 |
| Zfp639 | 0.008602392 | -0.02639 | 0.069 | 0.106 | 1 | 0 |
| Ankle2 | 0.008603764 | -0.04382 | 0.075 | 0.113 | 1 | 0 |
| Mzb1 | 0.008620546 | -0.23411 | 0.428 | 0.521 | 1 | 0 |
| Slc30a5 | 0.00869546 | -0.03514 | 0.104 | 0.15 | 1 | 0 |
| Dr1 | 0.008706364 | 0.003846 | 0.073 | 0.112 | 1 | 0 |
| Rtn4 | 0.008761522 | 0.022356 | 0.185 | 0.261 | 1 | 0 |
| U2af1 | 0.008794564 | 0.008234 | 0.184 | 0.258 | 1 | 0 |
| Tgif1 | 0.008816311 | 0.236119 | 0.423 | 0.432 | 1 | 0 |
| Atg3 | 0.00887804 | 0.035824 | 0.135 | 0.193 | 1 | 0 |
| Dennd5b | 0.008892548 | -0.0047 | 0.15 | 0.21 | 1 | 0 |
| Raly | 0.008897247 | -0.0079 | 0.257 | 0.356 | 1 | 0 |
| Ier5 | 0.008926198 | -0.08725 | 0.425 | 0.541 | 1 | 0 |
| St13 | 0.00893644 | -0.03755 | 0.284 | 0.378 | 1 | 0 |
| Arhgdia | 0.00898224 | -0.00076 | 0.361 | 0.498 | 1 | 0 |
| Dnajc21 | 0.00901835 | -0.02541 | 0.128 | 0.181 | 1 | 0 |
| Runx1 | 0.009078989 | -0.00907 | 0.186 | 0.255 | 1 | 0 |
| Rpn2 | 0.009082885 | 0.011294 | 0.141 | 0.202 | 1 | 0 |
| P2ry10b | 0.009102049 | -0.02501 | 0.069 | 0.106 | 1 | 0 |
| Cep192 | 0.009156111 | -0.0466 | 0.079 | 0.117 | 1 | 0 |
| Acaa1a | 0.009167967 | 0.02008 | 0.094 | 0.141 | 1 | 0 |
| Tmem108 | 0.009174543 | -0.07558 | 0.124 | 0.172 | 1 | 0 |
| Mafk | 0.009197332 | -0.02124 | 0.074 | 0.112 | 1 | 0 |
| Cdc5l | 0.009205467 | 0.006626 | 0.221 | 0.302 | 1 | 0 |
| Ndufa10 | 0.009330964 | -0.00732 | 0.114 | 0.163 | 1 | 0 |
| Pnrc2 | 0.009340509 | -0.01393 | 0.124 | 0.178 | 1 | 0 |
| Shoc2 | 0.009359598 | -0.01014 | 0.067 | 0.104 | 1 | 0 |
| Dpm3 | 0.009419527 | 0.01889 | 0.196 | 0.271 | 1 | 0 |
| Rsrc1 | 0.009426849 | -0.00938 | 0.086 | 0.128 | 1 | 0 |
| Dcaf7 | 0.009429192 | 0.024505 | 0.08 | 0.122 | 1 | 0 |
| Grk2 | 0.009446592 | -0.0236 | 0.354 | 0.486 | 1 | 0 |
| Ypel5 | 0.0094933 | 0.01395 | 0.123 | 0.177 | 1 | 0 |
| Mapkapk2 | 0.009535221 | -0.0155 | 0.119 | 0.169 | 1 | 0 |
| Gimap6 | 0.009560138 | 0.136695 | 0.707 | 0.734 | 1 | 0 |
| Tecr | 0.009584772 | 0.001116 | 0.171 | 0.237 | 1 | 0 |
| Cmpk1 | 0.009599398 | 0.011709 | 0.195 | 0.268 | 1 | 0 |
| 2510002D24Rik | 0.009600533 | -0.01288 | 0.096 | 0.141 | 1 | 0 |
| Fam120b | 0.009611751 | -0.0224 | 0.073 | 0.111 | 1 | 0 |
| Papola | 0.009616618 | 0.029047 | 0.247 | 0.34 | 1 | 0 |
| Sfpq | 0.009635319 | -0.06093 | 0.547 | 0.66 | 1 | 0 |
| Med4 | 0.009668011 | -0.00454 | 0.117 | 0.167 | 1 | 0 |
| Ngdn | 0.00969553 | 0.007026 | 0.11 | 0.16 | 1 | 0 |
| Vps35 | 0.009825067 | 0.015827 | 0.095 | 0.141 | 1 | 0 |
| Fndc3a | 0.009846649 | -0.00932 | 0.12 | 0.169 | 1 | 0 |
| Ssr4 | 0.009856571 | -0.13098 | 0.329 | 0.428 | 1 | 0 |
| Stt3b | 0.009918183 | 0.038027 | 0.214 | 0.3 | 1 | 0 |
| Ilkap | 0.009934938 | -0.00599 | 0.114 | 0.163 | 1 | 0 |
| Zbtb11 | 0.009963938 | 0.008815 | 0.124 | 0.178 | 1 | 0 |
| Cand1 | 0.009964154 | 0.00527 | 0.076 | 0.116 | 1 | 0 |
| Fgfr1op | 0.009973073 | -0.0165 | 0.138 | 0.193 | 1 | 0 |
| Lars2 | 7.81E-180 | 1.877594 | 0.983 | 0.411 | ###### | 1 |
| Gm42418 | 5.75E-173 | 2.095569 | 1 | 0.999 | ###### | 1 |
| Saa3 | 1.97E-150 | -2.40443 | 0.049 | 0.911 | ###### | 1 |
| Lyz2 | 3.84E-120 | -1.87081 | 0.481 | 0.911 | ###### | 1 |
| Fth1 | 7.94E-119 | -0.82786 | 0.994 | 1 | ###### | 1 |
| Rps15a | 9.34E-98 | -0.4991 | 1 | 0.999 | 2.90E-93 | 1 |
| Rps24 | 3.92E-87 | -0.39294 | 1 | 1 | 1.22E-82 | 1 |
| Ftl1 | 2.52E-84 | -0.76231 | 0.963 | 0.987 | 7.83E-80 | 1 |
| S100a8 | 1.69E-80 | -1.41488 | 0.244 | 0.759 | 5.24E-76 | 1 |
| Rps27 | 1.24E-76 | -0.44464 | 0.994 | 1 | 3.85E-72 | 1 |
| Rpl21 | 1.57E-74 | -0.44984 | 0.989 | 1 | 4.86E-70 | 1 |
| Rpl23 | 6.90E-73 | -0.38326 | 0.994 | 0.999 | 2.14E-68 | 1 |
| Apoe | 7.76E-73 | -1.34381 | 0.398 | 0.839 | 2.41E-68 | 1 |
| Rpl39 | 2.03E-71 | -0.48106 | 0.997 | 1 | 6.31E-67 | 1 |
| Rps27a | 1.14E-69 | -0.41741 | 0.994 | 1 | 3.53E-65 | 1 |
| Cxcl2 | 2.22E-69 | -1.36934 | 0.169 | 0.671 | 6.89E-65 | 1 |
| Macf1 | 1.65E-65 | 0.854271 | 0.9 | 0.501 | 5.14E-61 | 1 |
| Rpl8 | 2.17E-65 | -0.40177 | 0.997 | 0.999 | 6.74E-61 | 1 |
| Wfdc17 | 3.38E-65 | -1.4243 | 0.069 | 0.591 | 1.05E-60 | 1 |
| Uba52 | 1.31E-64 | -0.92124 | 0.86 | 0.926 | 4.07E-60 | 1 |
| Fau | 6.00E-64 | -0.3562 | 1 | 0.999 | 1.86E-59 | 1 |
| Rpl32 | 1.84E-62 | -0.40613 | 0.997 | 0.999 | 5.71E-58 | 1 |
| Rps10 | 3.20E-62 | -0.35047 | 0.997 | 0.999 | 9.93E-58 | 1 |
| Rpl13 | 3.34E-60 | -0.34532 | 0.994 | 1 | 1.04E-55 | 1 |
| Rps19 | 1.02E-58 | -0.35176 | 1 | 1 | 3.18E-54 | 1 |
| Malat1 | 2.15E-58 | 0.381325 | 1 | 0.997 | 6.68E-54 | 1 |
| Rps4x | 1.40E-57 | -0.37724 | 1 | 0.999 | 4.35E-53 | 1 |
| Rpl26 | 2.85E-57 | -0.38411 | 0.997 | 0.999 | 8.86E-53 | 1 |
| Rps18 | 6.02E-56 | -0.36531 | 0.991 | 0.999 | 1.87E-51 | 1 |
| Rpl29 | 1.29E-55 | -0.55164 | 0.943 | 0.978 | 3.99E-51 | 1 |
| S100a9 | 1.40E-55 | -1.11059 | 0.289 | 0.702 | 4.36E-51 | 1 |
| Rpl35 | 2.89E-55 | -0.50556 | 0.983 | 0.99 | 8.98E-51 | 1 |
| Rps16 | 6.53E-54 | -0.31191 | 0.997 | 0.999 | 2.03E-49 | 1 |
| Rpl27a | 9.82E-54 | -0.33329 | 1 | 0.999 | 3.05E-49 | 1 |
| Rpl34 | 6.00E-53 | -0.33594 | 0.991 | 1 | 1.86E-48 | 1 |
| Rpl12 | 9.88E-53 | -0.37696 | 0.994 | 0.999 | 3.07E-48 | 1 |
| Rpl36 | 1.14E-50 | -0.39909 | 0.986 | 0.997 | 3.54E-46 | 1 |
| Rpl9 | 1.76E-50 | -0.32406 | 0.997 | 0.998 | 5.47E-46 | 1 |
| Rpsa | 2.76E-49 | -0.35377 | 1 | 0.999 | 8.58E-45 | 1 |
| Il1b | 6.12E-49 | -1.13282 | 0.129 | 0.553 | 1.90E-44 | 1 |
| Ets1 | 1.39E-47 | 0.593621 | 0.963 | 0.695 | 4.31E-43 | 1 |
| Rpl24 | 1.46E-47 | -0.44205 | 0.983 | 0.988 | 4.52E-43 | 1 |
| Rps8 | 2.51E-47 | -0.29733 | 1 | 1 | 7.81E-43 | 1 |
| Rpl17 | 1.08E-46 | -0.31312 | 0.997 | 1 | 3.36E-42 | 1 |
| Rps25 | 3.27E-46 | -0.3598 | 1 | 0.996 | 1.01E-41 | 1 |
| Ddx6 | 3.73E-46 | 0.660751 | 0.857 | 0.483 | 1.16E-41 | 1 |
| Rps3a1 | 3.95E-46 | -0.29532 | 0.997 | 1 | 1.23E-41 | 1 |
| Tpt1 | 9.56E-46 | -0.26884 | 1 | 1 | 2.97E-41 | 1 |
| Rps29 | 9.29E-44 | -0.30018 | 0.994 | 1 | 2.89E-39 | 1 |
| Rpl28 | 1.63E-43 | -0.3604 | 0.994 | 0.999 | 5.05E-39 | 1 |
| Rps20 | 1.92E-43 | -0.30877 | 0.997 | 1 | 5.96E-39 | 1 |
| Rpl11 | 6.19E-42 | -0.34048 | 0.994 | 0.999 | 1.92E-37 | 1 |
| Rplp2 | 7.18E-42 | -0.29186 | 1 | 1 | 2.23E-37 | 1 |
| Rps14 | 9.17E-42 | -0.2701 | 0.997 | 1 | 2.85E-37 | 1 |
| Rps7 | 1.23E-41 | -0.29992 | 0.997 | 1 | 3.81E-37 | 1 |
| Rpl30 | 3.41E-41 | -0.29437 | 1 | 0.999 | 1.06E-36 | 1 |
| Myh9 | 1.37E-40 | 0.58594 | 0.777 | 0.364 | 4.26E-36 | 1 |
| Ighm | 5.86E-40 | 0.141058 | 1 | 0.958 | 1.82E-35 | 1 |
| Rps13 | 5.98E-38 | -0.27508 | 0.991 | 0.998 | 1.86E-33 | 1 |
| S100a6 | 1.93E-37 | -1.4453 | 0.115 | 0.474 | 5.98E-33 | 1 |
| Pax5 | 6.23E-37 | 0.567402 | 0.808 | 0.415 | 1.93E-32 | 1 |
| Rpl18a | 6.29E-37 | -0.26719 | 0.994 | 1 | 1.95E-32 | 1 |
| AY036118 | 7.92E-37 | 0.533721 | 0.954 | 0.766 | 2.46E-32 | 1 |
| Rps23 | 1.86E-36 | -0.28272 | 0.991 | 1 | 5.77E-32 | 1 |
| Rpl19 | 1.31E-35 | -0.26475 | 0.997 | 1 | 4.08E-31 | 1 |
| Rpl36a | 5.00E-35 | -0.40431 | 0.974 | 0.985 | 1.55E-30 | 1 |
| Rps5 | 8.23E-35 | -0.28684 | 0.994 | 0.998 | 2.55E-30 | 1 |
| Ubb | 6.54E-34 | -0.37278 | 0.983 | 0.991 | 2.03E-29 | 1 |
| Rpl18 | 4.11E-33 | -0.28884 | 0.994 | 0.997 | 1.28E-28 | 1 |
| Brwd1 | 4.36E-33 | 0.45719 | 0.413 | 0.123 | 1.35E-28 | 1 |
| Ctsd | 1.49E-32 | -0.93356 | 0.195 | 0.502 | 4.62E-28 | 1 |
| Cpm | 1.97E-32 | 0.49776 | 0.304 | 0.073 | 6.12E-28 | 1 |
| Rpl10a | 2.04E-32 | -0.34163 | 0.991 | 0.992 | 6.33E-28 | 1 |
| Med13 | 2.78E-32 | 0.511143 | 0.63 | 0.275 | 8.63E-28 | 1 |
| mt-Nd2 | 1.06E-31 | 0.294096 | 1 | 0.976 | 3.30E-27 | 1 |
| Rps11 | 1.34E-31 | -0.31805 | 1 | 0.997 | 4.17E-27 | 1 |
| Sptbn1 | 2.70E-31 | 0.524833 | 0.587 | 0.252 | 8.38E-27 | 1 |
| Rpl35a | 1.77E-30 | -0.22968 | 0.997 | 1 | 5.49E-26 | 1 |
| Rps26 | 2.74E-30 | -0.3045 | 0.994 | 0.994 | 8.52E-26 | 1 |
| Rplp0 | 3.14E-30 | -0.35242 | 0.983 | 0.996 | 9.76E-26 | 1 |
| Tmsb4x | 7.11E-29 | -0.27159 | 1 | 1 | 2.21E-24 | 1 |
| Rbm3 | 7.99E-29 | -0.68696 | 0.582 | 0.72 | 2.48E-24 | 1 |
| Rpl38 | 1.44E-28 | -0.26901 | 0.997 | 1 | 4.48E-24 | 1 |
| Prkcb | 3.13E-28 | 0.451642 | 0.63 | 0.286 | 9.73E-24 | 1 |
| Ier3 | 3.25E-28 | -0.85683 | 0.023 | 0.306 | 1.01E-23 | 1 |
| Foxp1 | 2.11E-27 | 0.430888 | 0.968 | 0.751 | 6.56E-23 | 1 |
| Rpl14 | 2.33E-27 | -0.32284 | 0.989 | 0.986 | 7.22E-23 | 1 |
| Rpl37 | 3.51E-27 | -0.27924 | 0.994 | 0.997 | 1.09E-22 | 1 |
| Ptprc | 3.51E-27 | 0.453814 | 0.943 | 0.691 | 1.09E-22 | 1 |
| Lcn2 | 4.21E-27 | -0.87442 | 0.017 | 0.295 | 1.31E-22 | 1 |
| Mycbp2 | 1.04E-26 | 0.513688 | 0.722 | 0.396 | 3.24E-22 | 1 |
| mt-Atp6 | 1.61E-26 | -0.18675 | 1 | 1 | 5.00E-22 | 1 |
| Foxo1 | 4.95E-26 | 0.452251 | 0.576 | 0.259 | 1.54E-21 | 1 |
| Nipbl | 4.95E-26 | 0.481285 | 0.65 | 0.336 | 1.54E-21 | 1 |
| Sbk1 | 5.14E-26 | 0.363786 | 0.318 | 0.093 | 1.60E-21 | 1 |
| Ankrd11 | 7.23E-26 | 0.458704 | 0.808 | 0.508 | 2.24E-21 | 1 |
| Cd22 | 9.25E-26 | 0.481967 | 0.59 | 0.272 | 2.87E-21 | 1 |
| Cerk | 1.11E-25 | 0.433358 | 0.504 | 0.206 | 3.45E-21 | 1 |
| Rpl6 | 2.83E-25 | -0.28016 | 0.986 | 0.996 | 8.78E-21 | 1 |
| Lrrk2 | 4.45E-25 | 0.438927 | 0.481 | 0.193 | 1.38E-20 | 1 |
| Ddx5 | 5.65E-25 | 0.31432 | 0.997 | 0.949 | 1.75E-20 | 1 |
| Mgea5 | 5.87E-25 | 0.402572 | 0.358 | 0.122 | 1.82E-20 | 1 |
| Usp24 | 6.50E-25 | 0.392384 | 0.324 | 0.102 | 2.02E-20 | 1 |
| Rps6 | 7.62E-25 | -0.26987 | 0.989 | 0.992 | 2.37E-20 | 1 |
| Rps21 | 1.93E-24 | -0.24691 | 0.994 | 0.999 | 6.00E-20 | 1 |
| Add3 | 2.00E-24 | 0.440865 | 0.633 | 0.31 | 6.21E-20 | 1 |
| Ripor2 | 2.76E-24 | 0.426052 | 0.725 | 0.385 | 8.56E-20 | 1 |
| Itsn2 | 2.94E-24 | 0.424442 | 0.567 | 0.263 | 9.12E-20 | 1 |
| Rplp1 | 3.03E-24 | -0.22935 | 1 | 0.999 | 9.40E-20 | 1 |
| Rps3 | 3.04E-24 | -0.22565 | 0.997 | 0.997 | 9.44E-20 | 1 |
| Chd3 | 3.97E-24 | 0.418521 | 0.404 | 0.15 | 1.23E-19 | 1 |
| Rpl15 | 4.21E-24 | -0.29 | 0.989 | 0.988 | 1.31E-19 | 1 |
| Itpr3 | 4.35E-24 | 0.378335 | 0.352 | 0.119 | 1.35E-19 | 1 |
| Akap13 | 4.50E-24 | 0.457836 | 0.842 | 0.543 | 1.40E-19 | 1 |
| Aff4 | 4.98E-24 | 0.459644 | 0.63 | 0.314 | 1.55E-19 | 1 |
| Cstb | 9.57E-24 | -0.77493 | 0.163 | 0.426 | 2.97E-19 | 1 |
| Dgkd | 1.01E-23 | 0.450795 | 0.61 | 0.296 | 3.12E-19 | 1 |
| Dock8 | 1.30E-23 | 0.376051 | 0.398 | 0.151 | 4.05E-19 | 1 |
| Vps37b | 1.33E-23 | 0.565458 | 0.734 | 0.448 | 4.14E-19 | 1 |
| Scd1 | 3.03E-23 | 0.451882 | 0.822 | 0.519 | 9.41E-19 | 1 |
| Fcer1g | 3.33E-23 | -0.82205 | 0.074 | 0.327 | 1.03E-18 | 1 |
| Prrc2c | 5.69E-23 | 0.39257 | 0.808 | 0.501 | 1.77E-18 | 1 |
| Nsd1 | 9.48E-23 | 0.415497 | 0.593 | 0.28 | 2.95E-18 | 1 |
| Tyrobp | 2.18E-22 | -0.78117 | 0.218 | 0.452 | 6.77E-18 | 1 |
| Arfgef1 | 5.94E-22 | 0.408921 | 0.47 | 0.204 | 1.85E-17 | 1 |
| Rasa3 | 6.59E-22 | 0.444286 | 0.447 | 0.189 | 2.05E-17 | 1 |
| Smg1 | 8.10E-22 | 0.426683 | 0.619 | 0.318 | 2.51E-17 | 1 |
| Hp | 8.90E-22 | -0.71115 | 0.011 | 0.236 | 2.76E-17 | 1 |
| Zcchc11 | 1.36E-21 | 0.422185 | 0.799 | 0.479 | 4.24E-17 | 1 |
| Sptan1 | 1.51E-21 | 0.366573 | 0.372 | 0.141 | 4.68E-17 | 1 |
| Sik1 | 1.51E-21 | 0.391358 | 0.562 | 0.264 | 4.70E-17 | 1 |
| Rock1 | 1.69E-21 | 0.402228 | 0.622 | 0.321 | 5.25E-17 | 1 |
| Fchsd2 | 2.05E-21 | 0.478611 | 0.656 | 0.357 | 6.38E-17 | 1 |
| Rpl23a | 2.12E-21 | -0.27253 | 0.974 | 0.976 | 6.58E-17 | 1 |
| Trp53inp1 | 2.18E-21 | 0.36826 | 0.324 | 0.112 | 6.76E-17 | 1 |
| Ptk2b | 2.46E-21 | 0.372506 | 0.496 | 0.22 | 7.65E-17 | 1 |
| Trim25 | 2.51E-21 | 0.480431 | 0.524 | 0.252 | 7.79E-17 | 1 |
| Wfdc21 | 3.16E-21 | -0.76033 | 0.057 | 0.298 | 9.83E-17 | 1 |
| Aff3 | 3.50E-21 | 0.374342 | 0.318 | 0.11 | 1.09E-16 | 1 |
| Rfx7 | 3.52E-21 | 0.355905 | 0.395 | 0.154 | 1.09E-16 | 1 |
| Birc6 | 3.70E-21 | 0.387206 | 0.57 | 0.275 | 1.15E-16 | 1 |
| Cd55 | 3.89E-21 | 0.431505 | 0.779 | 0.491 | 1.21E-16 | 1 |
| Cd14 | 4.77E-21 | -0.74807 | 0.049 | 0.28 | 1.48E-16 | 1 |
| Iqgap1 | 1.15E-20 | 0.38348 | 0.894 | 0.582 | 3.56E-16 | 1 |
| Dock2 | 1.23E-20 | 0.39004 | 0.679 | 0.372 | 3.82E-16 | 1 |
| Prpf4b | 1.37E-20 | 0.360108 | 0.673 | 0.35 | 4.26E-16 | 1 |
| Chd4 | 2.62E-20 | 0.43075 | 0.639 | 0.356 | 8.13E-16 | 1 |
| Lgals3 | 2.68E-20 | -0.72879 | 0.132 | 0.368 | 8.31E-16 | 1 |
| Ddx17 | 5.31E-20 | 0.355912 | 0.504 | 0.232 | 1.65E-15 | 1 |
| Bmp2k | 6.06E-20 | 0.380957 | 0.464 | 0.207 | 1.88E-15 | 1 |
| Gpr132 | 6.69E-20 | 0.394939 | 0.444 | 0.196 | 2.08E-15 | 1 |
| Itga4 | 6.73E-20 | 0.432817 | 0.719 | 0.417 | 2.09E-15 | 1 |
| mt-Co3 | 7.97E-20 | -0.16293 | 1 | 1 | 2.47E-15 | 1 |
| Syk | 8.85E-20 | 0.400758 | 0.831 | 0.558 | 2.75E-15 | 1 |
| 9930111J21Rik2 | 1.39E-19 | 0.440374 | 0.375 | 0.155 | 4.31E-15 | 1 |
| Bptf | 1.52E-19 | 0.381598 | 0.705 | 0.396 | 4.73E-15 | 1 |
| Fam208b | 1.53E-19 | 0.337499 | 0.318 | 0.115 | 4.74E-15 | 1 |
| Tmod3 | 1.58E-19 | 0.365691 | 0.67 | 0.375 | 4.89E-15 | 1 |
| Cfap43 | 2.00E-19 | 0.317616 | 0.315 | 0.112 | 6.20E-15 | 1 |
| Ralgps2 | 2.09E-19 | 0.401806 | 0.797 | 0.492 | 6.49E-15 | 1 |
| Mga | 2.45E-19 | 0.442689 | 0.364 | 0.153 | 7.61E-15 | 1 |
| Usp34 | 2.59E-19 | 0.379877 | 0.521 | 0.247 | 8.04E-15 | 1 |
| Wipi2 | 3.74E-19 | 0.280681 | 0.287 | 0.096 | 1.16E-14 | 1 |
| Dync1h1 | 3.96E-19 | 0.355325 | 0.341 | 0.132 | 1.23E-14 | 1 |
| Kdm5b | 4.98E-19 | 0.249983 | 0.221 | 0.062 | 1.55E-14 | 1 |
| Ccl3 | 5.93E-19 | -0.7169 | 0.017 | 0.222 | 1.84E-14 | 1 |
| Fcer2a | 6.37E-19 | 0.405503 | 0.582 | 0.293 | 1.98E-14 | 1 |
| Pde3b | 7.14E-19 | 0.335941 | 0.39 | 0.16 | 2.22E-14 | 1 |
| Fus | 9.44E-19 | 0.397217 | 0.871 | 0.585 | 2.93E-14 | 1 |
| Zyg11b | 1.00E-18 | 0.345182 | 0.309 | 0.113 | 3.11E-14 | 1 |
| Prdm2 | 1.10E-18 | 0.362738 | 0.361 | 0.146 | 3.42E-14 | 1 |
| Spag9 | 1.14E-18 | 0.380877 | 0.521 | 0.263 | 3.54E-14 | 1 |
| Rb1cc1 | 1.22E-18 | 0.405083 | 0.516 | 0.252 | 3.79E-14 | 1 |
| Fabp5 | 1.43E-18 | -0.70928 | 0.034 | 0.239 | 4.45E-14 | 1 |
| P2ry10 | 1.65E-18 | 0.371772 | 0.794 | 0.519 | 5.14E-14 | 1 |
| Zfp831 | 1.91E-18 | 0.283555 | 0.181 | 0.046 | 5.92E-14 | 1 |
| Plekha2 | 1.99E-18 | 0.339806 | 0.499 | 0.235 | 6.19E-14 | 1 |
| Kat6a | 2.84E-18 | 0.370124 | 0.372 | 0.155 | 8.82E-14 | 1 |
| Crebbp | 2.88E-18 | 0.363184 | 0.542 | 0.272 | 8.95E-14 | 1 |
| Fam107b | 2.96E-18 | 0.350933 | 0.888 | 0.629 | 9.19E-14 | 1 |
| Numa1 | 3.38E-18 | 0.342579 | 0.367 | 0.151 | 1.05E-13 | 1 |
| Dmxl1 | 3.48E-18 | 0.342555 | 0.639 | 0.344 | 1.08E-13 | 1 |
| Ddx3x | 3.51E-18 | 0.383755 | 0.665 | 0.378 | 1.09E-13 | 1 |
| Snx30 | 4.22E-18 | 0.342365 | 0.332 | 0.127 | 1.31E-13 | 1 |
| Prkcd | 4.38E-18 | 0.389479 | 0.433 | 0.193 | 1.36E-13 | 1 |
| Sorl1 | 6.04E-18 | 0.352471 | 0.696 | 0.403 | 1.88E-13 | 1 |
| Purb | 6.38E-18 | 0.363511 | 0.636 | 0.347 | 1.98E-13 | 1 |
| Ep400 | 7.01E-18 | 0.353671 | 0.415 | 0.185 | 2.18E-13 | 1 |
| Madd | 8.52E-18 | 0.28535 | 0.252 | 0.083 | 2.65E-13 | 1 |
| Setd2 | 1.05E-17 | 0.349993 | 0.484 | 0.228 | 3.25E-13 | 1 |
| Ptprj | 1.32E-17 | 0.317094 | 0.338 | 0.134 | 4.10E-13 | 1 |
| Helz2 | 1.32E-17 | 0.337656 | 0.364 | 0.151 | 4.11E-13 | 1 |
| Rabgap1l | 1.48E-17 | 0.410597 | 0.622 | 0.354 | 4.60E-13 | 1 |
| Susd6 | 1.71E-17 | 0.302937 | 0.37 | 0.155 | 5.30E-13 | 1 |
| Rpl37a | 1.74E-17 | -0.18518 | 0.997 | 1 | 5.40E-13 | 1 |
| Kmt2a | 2.08E-17 | 0.367063 | 0.639 | 0.354 | 6.46E-13 | 1 |
| Nedd9 | 2.09E-17 | 0.363883 | 0.375 | 0.163 | 6.50E-13 | 1 |
| Erbin | 2.58E-17 | 0.38492 | 0.361 | 0.153 | 8.02E-13 | 1 |
| Hip1r | 2.62E-17 | 0.305328 | 0.372 | 0.157 | 8.13E-13 | 1 |
| March7 | 2.69E-17 | 0.307217 | 0.536 | 0.264 | 8.34E-13 | 1 |
| Cnot1 | 3.24E-17 | 0.331302 | 0.415 | 0.188 | 1.01E-12 | 1 |
| Ccdc88c | 4.28E-17 | 0.323693 | 0.39 | 0.171 | 1.33E-12 | 1 |
| Lpgat1 | 5.08E-17 | 0.31742 | 0.375 | 0.16 | 1.58E-12 | 1 |
| Chst3 | 5.12E-17 | 0.331958 | 0.266 | 0.095 | 1.59E-12 | 1 |
| Cep350 | 5.41E-17 | 0.319565 | 0.304 | 0.116 | 1.68E-12 | 1 |
| Arid1b | 5.60E-17 | 0.286382 | 0.341 | 0.138 | 1.74E-12 | 1 |
| Kdm5a | 5.68E-17 | 0.338358 | 0.496 | 0.245 | 1.76E-12 | 1 |
| Setx | 5.72E-17 | 0.326539 | 0.318 | 0.126 | 1.78E-12 | 1 |
| Arhgef18 | 6.47E-17 | 0.33846 | 0.384 | 0.164 | 2.01E-12 | 1 |
| S100a4 | 8.17E-17 | -0.64437 | 0.014 | 0.195 | 2.54E-12 | 1 |
| Stk10 | 8.51E-17 | 0.326011 | 0.33 | 0.132 | 2.64E-12 | 1 |
| Kmt2c | 8.75E-17 | 0.348757 | 0.438 | 0.209 | 2.72E-12 | 1 |
| Rai1 | 1.01E-16 | 0.227708 | 0.117 | 0.022 | 3.13E-12 | 1 |
| Rictor | 1.16E-16 | 0.331556 | 0.415 | 0.187 | 3.60E-12 | 1 |
| Cecr2 | 1.35E-16 | 0.283741 | 0.186 | 0.053 | 4.18E-12 | 1 |
| Brd2 | 1.58E-16 | 0.331787 | 0.668 | 0.386 | 4.92E-12 | 1 |
| Txn1 | 1.62E-16 | -0.64119 | 0.289 | 0.459 | 5.02E-12 | 1 |
| Ikzf3 | 1.68E-16 | 0.370806 | 0.43 | 0.202 | 5.21E-12 | 1 |
| Rps9 | 1.69E-16 | -0.17307 | 0.997 | 0.999 | 5.26E-12 | 1 |
| Ash1l | 2.04E-16 | 0.357826 | 0.625 | 0.347 | 6.33E-12 | 1 |
| Klf13 | 2.57E-16 | 0.328958 | 0.547 | 0.286 | 7.98E-12 | 1 |
| Mllt10 | 3.00E-16 | 0.331005 | 0.361 | 0.159 | 9.33E-12 | 1 |
| Gm8369 | 3.10E-16 | -0.69204 | 0.524 | 0.644 | 9.63E-12 | 1 |
| Hspa1b | 3.21E-16 | -0.47782 | 0.109 | 0.344 | 9.98E-12 | 1 |
| Ube2h | 4.52E-16 | 0.34535 | 0.327 | 0.136 | 1.40E-11 | 1 |
| Yme1l1 | 7.01E-16 | 0.251014 | 0.367 | 0.155 | 2.18E-11 | 1 |
| Zfp148 | 7.33E-16 | 0.306222 | 0.344 | 0.146 | 2.28E-11 | 1 |
| Hnrnpul2 | 1.02E-15 | 0.295253 | 0.453 | 0.212 | 3.15E-11 | 1 |
| Scaf11 | 1.04E-15 | 0.302278 | 0.676 | 0.404 | 3.22E-11 | 1 |
| Phip | 1.12E-15 | 0.366839 | 0.61 | 0.347 | 3.49E-11 | 1 |
| Pgap1 | 1.22E-15 | 0.3055 | 0.599 | 0.332 | 3.78E-11 | 1 |
| Bicra | 1.30E-15 | 0.225029 | 0.183 | 0.053 | 4.03E-11 | 1 |
| Ciita | 1.30E-15 | 0.333724 | 0.458 | 0.225 | 4.05E-11 | 1 |
| Pten | 1.51E-15 | 0.302502 | 0.616 | 0.343 | 4.68E-11 | 1 |
| Fryl | 1.87E-15 | 0.281429 | 0.284 | 0.111 | 5.80E-11 | 1 |
| Vps13d | 1.92E-15 | 0.271151 | 0.198 | 0.062 | 5.96E-11 | 1 |
| BC005537 | 2.36E-15 | 0.316224 | 0.648 | 0.366 | 7.32E-11 | 1 |
| Rpl10 | 2.36E-15 | -0.25025 | 0.983 | 0.992 | 7.33E-11 | 1 |
| Zmynd11 | 2.48E-15 | 0.314183 | 0.461 | 0.225 | 7.72E-11 | 1 |
| S1pr1 | 2.86E-15 | 0.301076 | 0.43 | 0.206 | 8.89E-11 | 1 |
| St6gal1 | 3.42E-15 | 0.316041 | 0.292 | 0.118 | 1.06E-10 | 1 |
| Socs3 | 3.45E-15 | -0.67785 | 0.052 | 0.232 | 1.07E-10 | 1 |
| Wdfy4 | 3.53E-15 | 0.323703 | 0.393 | 0.185 | 1.10E-10 | 1 |
| Top2b | 3.65E-15 | 0.335719 | 0.55 | 0.292 | 1.13E-10 | 1 |
| Zc3h13 | 3.85E-15 | 0.280897 | 0.33 | 0.139 | 1.20E-10 | 1 |
| Sh3pxd2a | 4.54E-15 | 0.299868 | 0.183 | 0.056 | 1.41E-10 | 1 |
| Strbp | 4.88E-15 | 0.307763 | 0.427 | 0.2 | 1.52E-10 | 1 |
| Zfp740 | 7.34E-15 | 0.234652 | 0.212 | 0.07 | 2.28E-10 | 1 |
| Pde7a | 1.02E-14 | 0.286219 | 0.464 | 0.228 | 3.15E-10 | 1 |
| Bicd2 | 1.06E-14 | 0.177025 | 0.126 | 0.028 | 3.30E-10 | 1 |
| Malt1 | 1.17E-14 | 0.363868 | 0.653 | 0.402 | 3.62E-10 | 1 |
| Mid1 | 1.25E-14 | 0.183484 | 0.115 | 0.024 | 3.88E-10 | 1 |
| Smarca5 | 1.28E-14 | 0.313702 | 0.625 | 0.359 | 3.96E-10 | 1 |
| Nfatc3 | 1.34E-14 | 0.302776 | 0.628 | 0.349 | 4.15E-10 | 1 |
| Etnk1 | 1.89E-14 | 0.29786 | 0.585 | 0.326 | 5.88E-10 | 1 |
| Uhrf1bp1l | 2.22E-14 | 0.264738 | 0.215 | 0.075 | 6.91E-10 | 1 |
| Pecam1 | 2.35E-14 | 0.283575 | 0.43 | 0.209 | 7.30E-10 | 1 |
| Gxylt1 | 2.51E-14 | 0.261325 | 0.249 | 0.094 | 7.78E-10 | 1 |
| Ppia | 2.57E-14 | -0.27174 | 0.966 | 0.965 | 7.99E-10 | 1 |
| Glg1 | 2.77E-14 | 0.291286 | 0.312 | 0.134 | 8.59E-10 | 1 |
| Fam168b | 2.90E-14 | 0.264926 | 0.372 | 0.171 | 9.00E-10 | 1 |
| Cebpb | 2.91E-14 | -0.54715 | 0.547 | 0.677 | 9.05E-10 | 1 |
| Zmym2 | 3.00E-14 | 0.237759 | 0.212 | 0.073 | 9.32E-10 | 1 |
| Pfn1 | 3.12E-14 | -0.33423 | 0.871 | 0.859 | 9.67E-10 | 1 |
| Epb41 | 3.12E-14 | 0.269275 | 0.35 | 0.153 | 9.67E-10 | 1 |
| Itpr1 | 3.21E-14 | 0.302644 | 0.361 | 0.166 | 9.98E-10 | 1 |
| Huwe1 | 3.52E-14 | 0.30581 | 0.467 | 0.242 | 1.09E-09 | 1 |
| Pdzd8 | 3.74E-14 | 0.307191 | 0.232 | 0.087 | 1.16E-09 | 1 |
| Mtss1 | 4.32E-14 | 0.308957 | 0.55 | 0.3 | 1.34E-09 | 1 |
| Flii | 4.33E-14 | 0.255464 | 0.258 | 0.101 | 1.35E-09 | 1 |
| Rfc1 | 4.54E-14 | 0.260838 | 0.298 | 0.123 | 1.41E-09 | 1 |
| Baz2b | 4.75E-14 | 0.297054 | 0.59 | 0.333 | 1.47E-09 | 1 |
| Chd8 | 5.18E-14 | 0.330363 | 0.341 | 0.155 | 1.61E-09 | 1 |
| Bach2 | 5.85E-14 | 0.363051 | 0.539 | 0.298 | 1.82E-09 | 1 |
| Rpl3 | 6.61E-14 | -0.21313 | 0.991 | 0.988 | 2.05E-09 | 1 |
| Tpr | 6.90E-14 | 0.290344 | 0.696 | 0.42 | 2.14E-09 | 1 |
| mt-Nd3 | 7.90E-14 | 0.253586 | 0.963 | 0.815 | 2.45E-09 | 1 |
| Cnot6l | 8.00E-14 | 0.29484 | 0.562 | 0.303 | 2.48E-09 | 1 |
| Dennd1b | 8.07E-14 | 0.31703 | 0.393 | 0.191 | 2.50E-09 | 1 |
| C1qb | 8.52E-14 | -0.50717 | 0 | 0.142 | 2.65E-09 | 1 |
| Eef1a1 | 8.93E-14 | -0.16811 | 0.994 | 1 | 2.77E-09 | 1 |
| Nfkb1 | 1.03E-13 | 0.332459 | 0.536 | 0.304 | 3.19E-09 | 1 |
| Ncl | 1.08E-13 | 0.211124 | 0.905 | 0.64 | 3.35E-09 | 1 |
| Ppp6r1 | 1.10E-13 | 0.250079 | 0.258 | 0.102 | 3.42E-09 | 1 |
| Heatr6 | 1.10E-13 | 0.262214 | 0.186 | 0.062 | 3.42E-09 | 1 |
| Mau2 | 1.17E-13 | 0.274404 | 0.367 | 0.17 | 3.64E-09 | 1 |
| Ppp4r3a | 1.23E-13 | 0.299263 | 0.352 | 0.166 | 3.82E-09 | 1 |
| Smchd1 | 1.26E-13 | 0.305649 | 0.622 | 0.377 | 3.93E-09 | 1 |
| Cd19 | 1.37E-13 | 0.31776 | 0.648 | 0.383 | 4.26E-09 | 1 |
| Ep300 | 1.41E-13 | 0.310593 | 0.444 | 0.226 | 4.38E-09 | 1 |
| Arhgef1 | 1.42E-13 | 0.330197 | 0.607 | 0.355 | 4.41E-09 | 1 |
| Nsd3 | 1.45E-13 | 0.261636 | 0.547 | 0.295 | 4.52E-09 | 1 |
| Cep68 | 1.54E-13 | 0.184229 | 0.117 | 0.027 | 4.77E-09 | 1 |
| Ccrl2 | 1.57E-13 | -0.57241 | 0.02 | 0.171 | 4.89E-09 | 1 |
| Gm20186 | 1.64E-13 | 0.251224 | 0.163 | 0.051 | 5.10E-09 | 1 |
| Pbxip1 | 1.66E-13 | 0.317361 | 0.37 | 0.179 | 5.16E-09 | 1 |
| Trim11 | 1.84E-13 | 0.287746 | 0.246 | 0.098 | 5.72E-09 | 1 |
| Tnrc6b | 1.84E-13 | 0.34688 | 0.587 | 0.357 | 5.72E-09 | 1 |
| Dynll1 | 2.11E-13 | -0.48697 | 0.527 | 0.621 | 6.56E-09 | 1 |
| Tax1bp1 | 2.18E-13 | 0.267143 | 0.742 | 0.464 | 6.78E-09 | 1 |
| Hnrnpul1 | 2.21E-13 | 0.246198 | 0.344 | 0.156 | 6.87E-09 | 1 |
| Ago2 | 2.37E-13 | 0.250016 | 0.479 | 0.24 | 7.35E-09 | 1 |
| Rad21 | 2.39E-13 | 0.242584 | 0.372 | 0.171 | 7.41E-09 | 1 |
| Nckap1l | 2.75E-13 | 0.241182 | 0.33 | 0.149 | 8.54E-09 | 1 |
| Mon2 | 2.90E-13 | 0.210836 | 0.261 | 0.102 | 9.00E-09 | 1 |
| Plcg2 | 2.99E-13 | 0.261566 | 0.378 | 0.181 | 9.28E-09 | 1 |
| Rps12 | 3.10E-13 | -0.22226 | 0.991 | 0.998 | 9.62E-09 | 1 |
| Safb | 3.69E-13 | 0.26151 | 0.358 | 0.168 | 1.14E-08 | 1 |
| Zfp36l1 | 3.76E-13 | 0.272468 | 0.908 | 0.706 | 1.17E-08 | 1 |
| Xpr1 | 4.10E-13 | 0.255273 | 0.347 | 0.159 | 1.27E-08 | 1 |
| Aff1 | 4.17E-13 | 0.268308 | 0.347 | 0.159 | 1.30E-08 | 1 |
| Cyfip2 | 4.27E-13 | 0.267245 | 0.438 | 0.221 | 1.33E-08 | 1 |
| Cr2 | 4.57E-13 | 0.305227 | 0.352 | 0.165 | 1.42E-08 | 1 |
| AA467197 | 4.76E-13 | -0.49104 | 0.003 | 0.139 | 1.48E-08 | 1 |
| Tet2 | 4.85E-13 | 0.238813 | 0.241 | 0.095 | 1.51E-08 | 1 |
| Rpl7 | 5.11E-13 | -0.1783 | 0.997 | 0.994 | 1.59E-08 | 1 |
| Eif4g2 | 5.32E-13 | 0.290716 | 0.768 | 0.517 | 1.65E-08 | 1 |
| Ppfia1 | 5.40E-13 | 0.214582 | 0.203 | 0.072 | 1.68E-08 | 1 |
| Arhgap45 | 5.88E-13 | 0.246831 | 0.61 | 0.35 | 1.82E-08 | 1 |
| Cntrl | 6.42E-13 | 0.284194 | 0.35 | 0.164 | 1.99E-08 | 1 |
| Tmem123 | 7.13E-13 | 0.296027 | 0.585 | 0.347 | 2.22E-08 | 1 |
| Son | 7.97E-13 | 0.276617 | 0.905 | 0.731 | 2.48E-08 | 1 |
| Plk2 | 8.22E-13 | 0.388766 | 0.232 | 0.092 | 2.55E-08 | 1 |
| Rpl27 | 8.35E-13 | -0.21875 | 0.98 | 0.974 | 2.59E-08 | 1 |
| Nup210 | 8.92E-13 | 0.250366 | 0.221 | 0.084 | 2.77E-08 | 1 |
| Baz1b | 8.94E-13 | 0.216081 | 0.384 | 0.184 | 2.77E-08 | 1 |
| Arhgap25 | 9.36E-13 | 0.271934 | 0.358 | 0.175 | 2.91E-08 | 1 |
| Hspa1a | 9.64E-13 | -0.47998 | 0.106 | 0.295 | 2.99E-08 | 1 |
| Zcchc2 | 1.00E-12 | 0.197333 | 0.169 | 0.054 | 3.11E-08 | 1 |
| N4bp2 | 1.01E-12 | 0.243987 | 0.375 | 0.178 | 3.12E-08 | 1 |
| Gm10076 | 1.02E-12 | -0.53764 | 0.332 | 0.465 | 3.17E-08 | 1 |
| AW112010 | 1.04E-12 | -0.57187 | 0.089 | 0.254 | 3.24E-08 | 1 |
| Eif2ak3 | 1.05E-12 | 0.267644 | 0.332 | 0.156 | 3.25E-08 | 1 |
| Rnf20 | 1.06E-12 | 0.205939 | 0.269 | 0.11 | 3.28E-08 | 1 |
| Ncoa3 | 1.13E-12 | 0.251503 | 0.324 | 0.148 | 3.52E-08 | 1 |
| Fli1 | 1.23E-12 | 0.231641 | 0.493 | 0.254 | 3.83E-08 | 1 |
| Srsf5 | 1.37E-12 | 0.268018 | 0.874 | 0.654 | 4.24E-08 | 1 |
| Atad2b | 1.42E-12 | 0.264759 | 0.287 | 0.125 | 4.42E-08 | 1 |
| Slmap | 1.50E-12 | 0.201841 | 0.175 | 0.058 | 4.65E-08 | 1 |
| Ccm2 | 1.56E-12 | 0.26141 | 0.378 | 0.183 | 4.83E-08 | 1 |
| Msi2 | 1.58E-12 | 0.264094 | 0.41 | 0.207 | 4.92E-08 | 1 |
| Nktr | 1.77E-12 | 0.280136 | 0.579 | 0.337 | 5.50E-08 | 1 |
| Sfpq | 1.80E-12 | 0.28583 | 0.797 | 0.552 | 5.58E-08 | 1 |
| Ly6c2 | 1.88E-12 | -0.50803 | 0.026 | 0.172 | 5.84E-08 | 1 |
| Ptp4a3 | 1.92E-12 | 0.298294 | 0.53 | 0.3 | 5.96E-08 | 1 |
| Prdx1 | 1.96E-12 | -0.44023 | 0.524 | 0.603 | 6.10E-08 | 1 |
| Tram1 | 2.03E-12 | 0.277049 | 0.364 | 0.177 | 6.31E-08 | 1 |
| Ambra1 | 2.04E-12 | 0.187423 | 0.132 | 0.036 | 6.33E-08 | 1 |
| Zfp62 | 2.11E-12 | 0.215301 | 0.212 | 0.079 | 6.55E-08 | 1 |
| Parp1 | 2.16E-12 | 0.289122 | 0.47 | 0.251 | 6.70E-08 | 1 |
| Slc4a7 | 2.23E-12 | 0.267029 | 0.395 | 0.197 | 6.93E-08 | 1 |
| Herc1 | 2.29E-12 | 0.251018 | 0.35 | 0.169 | 7.10E-08 | 1 |
| Flna | 2.40E-12 | 0.344899 | 0.404 | 0.21 | 7.46E-08 | 1 |
| Rabep1 | 2.55E-12 | 0.203855 | 0.226 | 0.087 | 7.93E-08 | 1 |
| Spib | 2.59E-12 | 0.350064 | 0.407 | 0.212 | 8.04E-08 | 1 |
| Cyth1 | 2.60E-12 | 0.264081 | 0.542 | 0.298 | 8.09E-08 | 1 |
| Yy1 | 2.70E-12 | 0.226164 | 0.524 | 0.284 | 8.37E-08 | 1 |
| Kif21b | 2.75E-12 | 0.207853 | 0.33 | 0.148 | 8.53E-08 | 1 |
| Zbtb44 | 2.91E-12 | 0.215007 | 0.238 | 0.095 | 9.05E-08 | 1 |
| Ptp4a2 | 3.05E-12 | 0.270632 | 0.774 | 0.513 | 9.48E-08 | 1 |
| Rprd2 | 3.12E-12 | 0.216204 | 0.278 | 0.12 | 9.69E-08 | 1 |
| Bdp1 | 3.13E-12 | 0.245543 | 0.295 | 0.132 | 9.73E-08 | 1 |
| Il4ra | 3.25E-12 | 0.261479 | 0.341 | 0.164 | 1.01E-07 | 1 |
| Mbtps1 | 3.36E-12 | 0.24585 | 0.223 | 0.087 | 1.04E-07 | 1 |
| Rsrc2 | 4.08E-12 | 0.26392 | 0.785 | 0.501 | 1.27E-07 | 1 |
| Ptbp3 | 4.14E-12 | 0.290721 | 0.736 | 0.48 | 1.29E-07 | 1 |
| Syncrip | 4.29E-12 | 0.249334 | 0.438 | 0.228 | 1.33E-07 | 1 |
| Rassf3 | 4.68E-12 | 0.271939 | 0.341 | 0.163 | 1.45E-07 | 1 |
| Sipa1 | 4.89E-12 | 0.263197 | 0.47 | 0.252 | 1.52E-07 | 1 |
| Scml4 | 5.22E-12 | 0.184237 | 0.181 | 0.062 | 1.62E-07 | 1 |
| Cbl | 5.36E-12 | 0.231084 | 0.413 | 0.209 | 1.66E-07 | 1 |
| Ctsb | 5.79E-12 | -0.52097 | 0.189 | 0.35 | 1.80E-07 | 1 |
| Zranb1 | 6.20E-12 | 0.21746 | 0.309 | 0.14 | 1.93E-07 | 1 |
| Cux1 | 6.22E-12 | 0.291512 | 0.355 | 0.177 | 1.93E-07 | 1 |
| Cxxc5 | 6.44E-12 | 0.25998 | 0.232 | 0.093 | 2.00E-07 | 1 |
| Lbr | 6.64E-12 | 0.234285 | 0.467 | 0.247 | 2.06E-07 | 1 |
| Eloa | 6.73E-12 | 0.234731 | 0.235 | 0.094 | 2.09E-07 | 1 |
| Gapvd1 | 7.10E-12 | 0.243774 | 0.304 | 0.139 | 2.21E-07 | 1 |
| Ubr5 | 7.15E-12 | 0.231881 | 0.264 | 0.114 | 2.22E-07 | 1 |
| Prdx5 | 7.35E-12 | -0.57541 | 0.298 | 0.428 | 2.28E-07 | 1 |
| Hltf | 7.52E-12 | 0.216253 | 0.201 | 0.076 | 2.33E-07 | 1 |
| Prpf38b | 7.83E-12 | 0.272042 | 0.628 | 0.384 | 2.43E-07 | 1 |
| Usp19 | 8.68E-12 | 0.23106 | 0.301 | 0.136 | 2.69E-07 | 1 |
| Ifnar1 | 9.00E-12 | 0.270089 | 0.289 | 0.13 | 2.79E-07 | 1 |
| Zfp236 | 9.83E-12 | 0.183368 | 0.129 | 0.037 | 3.05E-07 | 1 |
| Senp1 | 9.94E-12 | 0.240186 | 0.169 | 0.059 | 3.09E-07 | 1 |
| Prim2 | 9.98E-12 | 0.196243 | 0.143 | 0.044 | 3.10E-07 | 1 |
| Pkn2 | 1.02E-11 | 0.239115 | 0.395 | 0.194 | 3.16E-07 | 1 |
| Lyst | 1.04E-11 | 0.260874 | 0.352 | 0.174 | 3.22E-07 | 1 |
| Rps17 | 1.05E-11 | -0.22933 | 0.957 | 0.961 | 3.27E-07 | 1 |
| Sun2 | 1.15E-11 | 0.215408 | 0.318 | 0.147 | 3.57E-07 | 1 |
| Fmnl1 | 1.19E-11 | 0.23226 | 0.309 | 0.144 | 3.71E-07 | 1 |
| Pi4ka | 1.31E-11 | 0.204829 | 0.186 | 0.069 | 4.07E-07 | 1 |
| B2m | 1.42E-11 | -0.23046 | 0.974 | 0.968 | 4.40E-07 | 1 |
| Gfod1 | 1.47E-11 | 0.164569 | 0.126 | 0.035 | 4.56E-07 | 1 |
| Brpf1 | 1.68E-11 | 0.194234 | 0.175 | 0.062 | 5.21E-07 | 1 |
| Rapgef6 | 1.69E-11 | 0.256682 | 0.556 | 0.325 | 5.26E-07 | 1 |
| Ccnl1 | 1.71E-11 | 0.244123 | 0.693 | 0.436 | 5.30E-07 | 1 |
| Retreg1 | 1.77E-11 | 0.207216 | 0.387 | 0.191 | 5.50E-07 | 1 |
| Arhgef3 | 1.79E-11 | 0.244758 | 0.241 | 0.101 | 5.57E-07 | 1 |
| Rubcn | 1.81E-11 | 0.226165 | 0.238 | 0.098 | 5.62E-07 | 1 |
| Lgals1 | 1.85E-11 | -0.55434 | 0.049 | 0.191 | 5.76E-07 | 1 |
| Kdm6b | 1.88E-11 | 0.286335 | 0.65 | 0.402 | 5.83E-07 | 1 |
| Snrnp70 | 1.91E-11 | 0.228675 | 0.665 | 0.404 | 5.92E-07 | 1 |
| Naca | 1.91E-11 | -0.2614 | 0.928 | 0.893 | 5.93E-07 | 1 |
| Helz | 1.91E-11 | 0.258668 | 0.238 | 0.101 | 5.93E-07 | 1 |
| Eif3a | 1.94E-11 | 0.300039 | 0.602 | 0.366 | 6.02E-07 | 1 |
| Sesn2 | 2.01E-11 | 0.157177 | 0.112 | 0.029 | 6.25E-07 | 1 |
| Trim33 | 2.02E-11 | 0.233561 | 0.255 | 0.11 | 6.26E-07 | 1 |
| Dnaja1 | 2.07E-11 | -0.49086 | 0.711 | 0.712 | 6.41E-07 | 1 |
| Swap70 | 2.17E-11 | 0.279663 | 0.47 | 0.261 | 6.75E-07 | 1 |
| Peli1 | 2.20E-11 | 0.267946 | 0.487 | 0.269 | 6.82E-07 | 1 |
| Rbms1 | 2.22E-11 | 0.168655 | 0.395 | 0.198 | 6.89E-07 | 1 |
| Zfp729a | 2.22E-11 | 0.191534 | 0.123 | 0.035 | 6.91E-07 | 1 |
| Sf3b1 | 2.25E-11 | 0.2673 | 0.797 | 0.565 | 6.97E-07 | 1 |
| Ankhd1 | 2.29E-11 | 0.253218 | 0.307 | 0.146 | 7.11E-07 | 1 |
| Gpbp1l1 | 2.31E-11 | 0.240026 | 0.292 | 0.134 | 7.19E-07 | 1 |
| Lnpep | 2.35E-11 | 0.298907 | 0.444 | 0.249 | 7.30E-07 | 1 |
| Iws1 | 2.44E-11 | 0.231996 | 0.278 | 0.126 | 7.56E-07 | 1 |
| Ncor1 | 2.45E-11 | 0.238189 | 0.65 | 0.383 | 7.61E-07 | 1 |
| Ifitm2 | 2.62E-11 | -0.49012 | 0.034 | 0.171 | 8.15E-07 | 1 |
| Atp8a1 | 2.71E-11 | 0.218653 | 0.252 | 0.107 | 8.40E-07 | 1 |
| Ccl4 | 2.78E-11 | -0.55737 | 0.037 | 0.176 | 8.62E-07 | 1 |
| Appbp2 | 2.91E-11 | 0.234357 | 0.241 | 0.103 | 9.05E-07 | 1 |
| Usp7 | 2.97E-11 | 0.239979 | 0.393 | 0.202 | 9.21E-07 | 1 |
| Hook3 | 3.07E-11 | 0.2021 | 0.261 | 0.114 | 9.52E-07 | 1 |
| Cmah | 3.07E-11 | 0.294167 | 0.476 | 0.267 | 9.53E-07 | 1 |
| Sesn3 | 3.26E-11 | 0.302329 | 0.364 | 0.187 | 1.01E-06 | 1 |
| Tgfb1 | 3.33E-11 | 0.292845 | 0.607 | 0.379 | 1.03E-06 | 1 |
| Rpl22 | 3.33E-11 | -0.19661 | 0.997 | 0.988 | 1.04E-06 | 1 |
| Kmt2e | 3.38E-11 | 0.251507 | 0.825 | 0.613 | 1.05E-06 | 1 |
| Arap1 | 3.40E-11 | 0.228813 | 0.258 | 0.114 | 1.05E-06 | 1 |
| Tcf12 | 3.46E-11 | 0.25677 | 0.433 | 0.233 | 1.07E-06 | 1 |
| Rpl5 | 3.50E-11 | -0.16577 | 0.994 | 0.992 | 1.09E-06 | 1 |
| Setd5 | 3.60E-11 | 0.230972 | 0.347 | 0.171 | 1.12E-06 | 1 |
| Myo1g | 3.62E-11 | 0.259877 | 0.289 | 0.137 | 1.12E-06 | 1 |
| Stat4 | 3.90E-11 | 0.248104 | 0.519 | 0.288 | 1.21E-06 | 1 |
| Chd7 | 3.96E-11 | 0.298931 | 0.327 | 0.164 | 1.23E-06 | 1 |
| Pgm2l1 | 4.31E-11 | 0.202425 | 0.146 | 0.047 | 1.34E-06 | 1 |
| Il10ra | 4.55E-11 | 0.243644 | 0.355 | 0.177 | 1.41E-06 | 1 |
| Pum2 | 4.69E-11 | 0.269288 | 0.507 | 0.288 | 1.46E-06 | 1 |
| Cdc37l1 | 4.92E-11 | 0.236383 | 0.321 | 0.153 | 1.53E-06 | 1 |
| Gpd1l | 5.03E-11 | 0.237201 | 0.232 | 0.098 | 1.56E-06 | 1 |
| Bod1l | 5.17E-11 | 0.340897 | 0.427 | 0.243 | 1.61E-06 | 1 |
| Sp1 | 5.19E-11 | 0.258319 | 0.278 | 0.128 | 1.61E-06 | 1 |
| Gtf3c2 | 5.29E-11 | 0.212244 | 0.229 | 0.096 | 1.64E-06 | 1 |
| Cdk12 | 5.35E-11 | 0.252177 | 0.43 | 0.236 | 1.66E-06 | 1 |
| Chchd2 | 5.38E-11 | -0.32241 | 0.779 | 0.77 | 1.67E-06 | 1 |
| Ppp2r5a | 5.38E-11 | 0.245724 | 0.41 | 0.218 | 1.67E-06 | 1 |
| Cox4i1 | 5.45E-11 | -0.30799 | 0.819 | 0.828 | 1.69E-06 | 1 |
| Jmy | 5.50E-11 | 0.193403 | 0.152 | 0.051 | 1.71E-06 | 1 |
| Clec4d | 5.91E-11 | -0.43112 | 0.017 | 0.141 | 1.84E-06 | 1 |
| Tcf20 | 6.01E-11 | 0.201411 | 0.261 | 0.115 | 1.87E-06 | 1 |
| Msl2 | 6.16E-11 | 0.221131 | 0.281 | 0.128 | 1.91E-06 | 1 |
| Suco | 6.19E-11 | 0.227765 | 0.238 | 0.101 | 1.92E-06 | 1 |
| Rack1 | 6.78E-11 | -0.17931 | 0.997 | 0.988 | 2.11E-06 | 1 |
| Specc1l | 6.93E-11 | 0.231877 | 0.235 | 0.1 | 2.15E-06 | 1 |
| Smg7 | 7.35E-11 | 0.191369 | 0.215 | 0.087 | 2.28E-06 | 1 |
| Kpna4 | 7.41E-11 | 0.224207 | 0.395 | 0.205 | 2.30E-06 | 1 |
| Cdt1 | 7.63E-11 | 0.228344 | 0.289 | 0.135 | 2.37E-06 | 1 |
| Ttc3 | 7.79E-11 | 0.249216 | 0.223 | 0.095 | 2.42E-06 | 1 |
| Chuk | 7.96E-11 | 0.209683 | 0.241 | 0.103 | 2.47E-06 | 1 |
| Wbp11 | 7.99E-11 | 0.214291 | 0.372 | 0.191 | 2.48E-06 | 1 |
| Gabpb2 | 8.11E-11 | 0.265224 | 0.37 | 0.191 | 2.52E-06 | 1 |
| Cd63 | 8.11E-11 | -0.43404 | 0.014 | 0.136 | 2.52E-06 | 1 |
| Ubl5 | 8.16E-11 | -0.40534 | 0.49 | 0.583 | 2.54E-06 | 1 |
| Prrc2a | 8.28E-11 | 0.231602 | 0.427 | 0.227 | 2.57E-06 | 1 |
| Lbh | 8.33E-11 | 0.216022 | 0.461 | 0.246 | 2.59E-06 | 1 |
| 9930021J03Rik | 8.54E-11 | 0.229547 | 0.206 | 0.083 | 2.65E-06 | 1 |
| Cd2 | 8.69E-11 | 0.252572 | 0.496 | 0.28 | 2.70E-06 | 1 |
| Arhgap4 | 8.74E-11 | 0.19163 | 0.241 | 0.102 | 2.71E-06 | 1 |
| Hnrnph1 | 8.89E-11 | 0.258733 | 0.656 | 0.42 | 2.76E-06 | 1 |
| Fam117b | 9.01E-11 | 0.265042 | 0.307 | 0.15 | 2.80E-06 | 1 |
| Slpi | 9.50E-11 | -0.61226 | 0.043 | 0.178 | 2.95E-06 | 1 |
| Cul3 | 9.55E-11 | 0.224395 | 0.384 | 0.201 | 2.97E-06 | 1 |
| Akap8 | 9.86E-11 | 0.207232 | 0.278 | 0.126 | 3.06E-06 | 1 |
| Nr2c2 | 9.98E-11 | 0.240215 | 0.261 | 0.119 | 3.10E-06 | 1 |
| Acin1 | 1.03E-10 | 0.231665 | 0.573 | 0.337 | 3.19E-06 | 1 |
| Clec4e | 1.09E-10 | -0.46609 | 0.026 | 0.151 | 3.37E-06 | 1 |
| Txndc16 | 1.10E-10 | 0.269683 | 0.464 | 0.26 | 3.43E-06 | 1 |
| Clint1 | 1.16E-10 | 0.26785 | 0.378 | 0.198 | 3.62E-06 | 1 |
| Stat5b | 1.17E-10 | 0.139561 | 0.158 | 0.055 | 3.63E-06 | 1 |
| Atf7ip | 1.18E-10 | 0.237409 | 0.41 | 0.218 | 3.65E-06 | 1 |
| Gcc2 | 1.20E-10 | 0.224533 | 0.218 | 0.091 | 3.73E-06 | 1 |
| Lmtk2 | 1.22E-10 | 0.17354 | 0.117 | 0.034 | 3.77E-06 | 1 |
| Srrm2 | 1.26E-10 | 0.233292 | 0.842 | 0.625 | 3.91E-06 | 1 |
| Fam43a | 1.26E-10 | 0.234776 | 0.355 | 0.18 | 3.92E-06 | 1 |
| Trip12 | 1.29E-10 | 0.225997 | 0.395 | 0.207 | 4.02E-06 | 1 |
| Arcn1 | 1.36E-10 | 0.209795 | 0.35 | 0.175 | 4.21E-06 | 1 |
| L3mbtl3 | 1.37E-10 | 0.213788 | 0.169 | 0.062 | 4.26E-06 | 1 |
| Cop1 | 1.39E-10 | 0.21666 | 0.338 | 0.168 | 4.31E-06 | 1 |
| Cmip | 1.40E-10 | 0.23021 | 0.249 | 0.11 | 4.34E-06 | 1 |
| Tgfbi | 1.43E-10 | -0.41967 | 0.017 | 0.137 | 4.43E-06 | 1 |
| Kmt5b | 1.52E-10 | 0.233167 | 0.309 | 0.151 | 4.73E-06 | 1 |
| Josd1 | 1.59E-10 | 0.170496 | 0.132 | 0.041 | 4.95E-06 | 1 |
| Mxd4 | 1.65E-10 | 0.242672 | 0.289 | 0.141 | 5.14E-06 | 1 |
| Hectd1 | 1.66E-10 | 0.227162 | 0.444 | 0.246 | 5.16E-06 | 1 |
| 4932438A13Rik | 1.67E-10 | 0.24163 | 0.447 | 0.249 | 5.19E-06 | 1 |
| Atg16l2 | 1.69E-10 | 0.172371 | 0.178 | 0.066 | 5.25E-06 | 1 |
| Sla | 1.69E-10 | 0.234717 | 0.407 | 0.223 | 5.26E-06 | 1 |
| Zfp110 | 1.72E-10 | 0.159521 | 0.117 | 0.034 | 5.33E-06 | 1 |
| Ccr7 | 1.73E-10 | 0.271957 | 0.811 | 0.586 | 5.36E-06 | 1 |
| Brd4 | 1.77E-10 | 0.184283 | 0.47 | 0.255 | 5.48E-06 | 1 |
| C1galt1 | 1.81E-10 | 0.275999 | 0.292 | 0.142 | 5.63E-06 | 1 |
| Zfp292 | 1.92E-10 | 0.234103 | 0.458 | 0.256 | 5.95E-06 | 1 |
| Tab2 | 1.92E-10 | 0.207988 | 0.321 | 0.159 | 5.95E-06 | 1 |
| Ablim1 | 1.96E-10 | 0.253504 | 0.61 | 0.374 | 6.08E-06 | 1 |
| Dock11 | 1.98E-10 | 0.237254 | 0.352 | 0.18 | 6.16E-06 | 1 |
| Map4k2 | 1.99E-10 | 0.229428 | 0.33 | 0.162 | 6.17E-06 | 1 |
| Lrba | 2.00E-10 | 0.215934 | 0.172 | 0.064 | 6.21E-06 | 1 |
| Hnrnpc | 2.00E-10 | 0.246549 | 0.642 | 0.406 | 6.23E-06 | 1 |
| Rpl31 | 2.06E-10 | -0.24404 | 0.934 | 0.906 | 6.39E-06 | 1 |
| Ahnak | 2.14E-10 | 0.337683 | 0.327 | 0.172 | 6.65E-06 | 1 |
| Ythdc1 | 2.17E-10 | 0.262068 | 0.55 | 0.335 | 6.73E-06 | 1 |
| Dicer1 | 2.31E-10 | 0.243312 | 0.275 | 0.128 | 7.16E-06 | 1 |
| Asxl2 | 2.32E-10 | 0.211675 | 0.324 | 0.159 | 7.21E-06 | 1 |
| Ivns1abp | 2.32E-10 | 0.199675 | 0.407 | 0.217 | 7.21E-06 | 1 |
| Herc2 | 2.34E-10 | 0.255004 | 0.198 | 0.081 | 7.26E-06 | 1 |
| Lats1 | 2.47E-10 | 0.166336 | 0.158 | 0.056 | 7.67E-06 | 1 |
| Sp100 | 2.48E-10 | 0.262362 | 0.665 | 0.427 | 7.71E-06 | 1 |
| Cyld | 2.74E-10 | 0.196121 | 0.393 | 0.205 | 8.52E-06 | 1 |
| Tnks2 | 2.81E-10 | 0.218187 | 0.332 | 0.167 | 8.73E-06 | 1 |
| Hnrnpdl | 2.82E-10 | 0.292045 | 0.573 | 0.363 | 8.75E-06 | 1 |
| Ralgapa2 | 2.83E-10 | 0.147969 | 0.103 | 0.028 | 8.80E-06 | 1 |
| Birc3 | 2.88E-10 | 0.256931 | 0.57 | 0.347 | 8.95E-06 | 1 |
| Hexim1 | 3.08E-10 | 0.255589 | 0.387 | 0.208 | 9.57E-06 | 1 |
| Esyt2 | 3.09E-10 | 0.185874 | 0.201 | 0.08 | 9.61E-06 | 1 |
| Mef2d | 3.13E-10 | 0.242662 | 0.467 | 0.268 | 9.71E-06 | 1 |
| Rflnb | 3.16E-10 | 0.154342 | 0.12 | 0.036 | 9.80E-06 | 1 |
| Usp9x | 3.50E-10 | 0.249019 | 0.407 | 0.222 | 1.09E-05 | 1 |
| Strn3 | 3.56E-10 | 0.207041 | 0.324 | 0.162 | 1.10E-05 | 1 |
| Ezr | 3.64E-10 | 0.261519 | 0.702 | 0.463 | 1.13E-05 | 1 |
| Mark3 | 3.68E-10 | 0.216135 | 0.295 | 0.14 | 1.14E-05 | 1 |
| Cabin1 | 3.71E-10 | 0.190563 | 0.198 | 0.082 | 1.15E-05 | 1 |
| Smg6 | 3.74E-10 | 0.195566 | 0.261 | 0.119 | 1.16E-05 | 1 |
| Baz2a | 3.81E-10 | 0.236903 | 0.321 | 0.164 | 1.18E-05 | 1 |
| Cdk13 | 3.93E-10 | 0.191745 | 0.361 | 0.187 | 1.22E-05 | 1 |
| Fam160b1 | 4.01E-10 | 0.164791 | 0.115 | 0.034 | 1.25E-05 | 1 |
| Sec24b | 4.06E-10 | 0.17634 | 0.178 | 0.068 | 1.26E-05 | 1 |
| Trip11 | 4.07E-10 | 0.27581 | 0.312 | 0.158 | 1.26E-05 | 1 |
| Prep | 4.10E-10 | 0.146213 | 0.115 | 0.034 | 1.27E-05 | 1 |
| Tmem127 | 4.30E-10 | 0.190952 | 0.143 | 0.049 | 1.33E-05 | 1 |
| Mtmr3 | 4.38E-10 | 0.193705 | 0.252 | 0.112 | 1.36E-05 | 1 |
| Arhgap31 | 4.65E-10 | 0.205179 | 0.198 | 0.08 | 1.44E-05 | 1 |
| Lrrc8d | 4.66E-10 | 0.192432 | 0.215 | 0.091 | 1.45E-05 | 1 |
| Tmem245 | 4.69E-10 | 0.232369 | 0.269 | 0.128 | 1.46E-05 | 1 |
| Ttc7 | 4.72E-10 | 0.191252 | 0.232 | 0.102 | 1.47E-05 | 1 |
| Osbpl8 | 4.84E-10 | 0.23057 | 0.304 | 0.15 | 1.50E-05 | 1 |
| Larp1 | 4.86E-10 | 0.183864 | 0.307 | 0.151 | 1.51E-05 | 1 |
| Slc26a2 | 4.86E-10 | 0.162518 | 0.126 | 0.04 | 1.51E-05 | 1 |
| Zbtb10 | 4.89E-10 | 0.193786 | 0.198 | 0.08 | 1.52E-05 | 1 |
| Atxn7 | 5.00E-10 | 0.204115 | 0.212 | 0.089 | 1.55E-05 | 1 |
| Golga4 | 5.07E-10 | 0.229438 | 0.244 | 0.112 | 1.57E-05 | 1 |
| Hbp1 | 5.32E-10 | 0.247107 | 0.35 | 0.184 | 1.65E-05 | 1 |
| Eml4 | 5.36E-10 | 0.259 | 0.487 | 0.286 | 1.66E-05 | 1 |
| Ralgapa1 | 5.47E-10 | 0.178102 | 0.206 | 0.086 | 1.70E-05 | 1 |
| Sh3bgrl3 | 5.67E-10 | -0.27375 | 0.908 | 0.866 | 1.76E-05 | 1 |
| Ptbp2 | 5.77E-10 | 0.190524 | 0.181 | 0.071 | 1.79E-05 | 1 |
| Usp15 | 5.80E-10 | 0.228216 | 0.39 | 0.209 | 1.80E-05 | 1 |
| Ifi27l2a | 6.10E-10 | 0.364809 | 0.499 | 0.302 | 1.90E-05 | 1 |
| Msh2 | 6.27E-10 | 0.20874 | 0.152 | 0.055 | 1.95E-05 | 1 |
| Slc12a6 | 6.28E-10 | 0.193922 | 0.464 | 0.254 | 1.95E-05 | 1 |
| Cxcr5 | 6.38E-10 | 0.239601 | 0.444 | 0.251 | 1.98E-05 | 1 |
| Arih1 | 6.60E-10 | 0.210797 | 0.39 | 0.209 | 2.05E-05 | 1 |
| Itch | 6.61E-10 | 0.207462 | 0.438 | 0.237 | 2.05E-05 | 1 |
| Ncoa1 | 6.88E-10 | 0.210707 | 0.212 | 0.089 | 2.14E-05 | 1 |
| Brd3 | 6.91E-10 | 0.185995 | 0.232 | 0.102 | 2.15E-05 | 1 |
| Irs2 | 7.47E-10 | 0.27026 | 0.398 | 0.219 | 2.32E-05 | 1 |
| Parg | 7.94E-10 | 0.207053 | 0.16 | 0.06 | 2.47E-05 | 1 |
| Btf3 | 8.14E-10 | -0.27613 | 0.84 | 0.793 | 2.53E-05 | 1 |
| Gga2 | 8.37E-10 | 0.226221 | 0.381 | 0.205 | 2.60E-05 | 1 |
| Ptbp1 | 8.61E-10 | 0.230217 | 0.367 | 0.2 | 2.67E-05 | 1 |
| Nfrkb | 8.62E-10 | 0.204216 | 0.181 | 0.072 | 2.68E-05 | 1 |
| Traf6 | 8.78E-10 | 0.15782 | 0.112 | 0.033 | 2.73E-05 | 1 |
| Mndal | 8.99E-10 | 0.242241 | 0.673 | 0.434 | 2.79E-05 | 1 |
| Rbm22 | 9.05E-10 | 0.148255 | 0.304 | 0.146 | 2.81E-05 | 1 |
| Unc93b1 | 9.14E-10 | 0.257237 | 0.676 | 0.435 | 2.84E-05 | 1 |
| Rasgrp1 | 9.25E-10 | 0.222804 | 0.203 | 0.087 | 2.87E-05 | 1 |
| Ptpn6 | 9.44E-10 | 0.227255 | 0.576 | 0.353 | 2.93E-05 | 1 |
| Sec24c | 9.60E-10 | 0.210731 | 0.189 | 0.078 | 2.98E-05 | 1 |
| Ap2b1 | 9.66E-10 | 0.196748 | 0.289 | 0.142 | 3.00E-05 | 1 |
| Usp38 | 1.00E-09 | 0.186804 | 0.223 | 0.097 | 3.12E-05 | 1 |
| Zc3hav1 | 1.01E-09 | 0.225135 | 0.433 | 0.239 | 3.13E-05 | 1 |
| Hnrnpu | 1.04E-09 | 0.251567 | 0.708 | 0.474 | 3.22E-05 | 1 |
| 4930523C07Rik | 1.04E-09 | 0.249526 | 0.734 | 0.494 | 3.22E-05 | 1 |
| Aftph | 1.05E-09 | 0.215688 | 0.223 | 0.1 | 3.28E-05 | 1 |
| Smap2 | 1.08E-09 | 0.231897 | 0.665 | 0.419 | 3.35E-05 | 1 |
| Vars | 1.13E-09 | 0.236601 | 0.476 | 0.279 | 3.52E-05 | 1 |
| Cd72 | 1.14E-09 | 0.277201 | 0.418 | 0.237 | 3.53E-05 | 1 |
| 1700017B05Rik | 1.15E-09 | 0.228087 | 0.255 | 0.121 | 3.56E-05 | 1 |
| Safb2 | 1.16E-09 | 0.204943 | 0.458 | 0.254 | 3.59E-05 | 1 |
| Tgfbr2 | 1.17E-09 | 0.166693 | 0.444 | 0.239 | 3.65E-05 | 1 |
| Pkd1 | 1.20E-09 | 0.152566 | 0.163 | 0.061 | 3.73E-05 | 1 |
| Got1 | 1.21E-09 | 0.228364 | 0.344 | 0.181 | 3.76E-05 | 1 |
| Stag1 | 1.22E-09 | 0.209409 | 0.338 | 0.173 | 3.80E-05 | 1 |
| Ankrd13a | 1.22E-09 | 0.174898 | 0.266 | 0.126 | 3.80E-05 | 1 |
| Treml2 | 1.24E-09 | 0.176037 | 0.298 | 0.142 | 3.87E-05 | 1 |
| Acbd3 | 1.25E-09 | 0.201211 | 0.292 | 0.142 | 3.89E-05 | 1 |
| Rsf1 | 1.26E-09 | 0.235226 | 0.364 | 0.196 | 3.91E-05 | 1 |
| Mysm1 | 1.31E-09 | 0.207518 | 0.309 | 0.154 | 4.07E-05 | 1 |
| Arid4a | 1.34E-09 | 0.21441 | 0.39 | 0.211 | 4.16E-05 | 1 |
| Fn1 | 1.35E-09 | -0.38205 | 0.017 | 0.128 | 4.20E-05 | 1 |
| Gm26853 | 1.37E-09 | 0.155402 | 0.112 | 0.034 | 4.26E-05 | 1 |
| Pip4k2b | 1.37E-09 | 0.146889 | 0.117 | 0.037 | 4.27E-05 | 1 |
| Ubald2 | 1.38E-09 | 0.225053 | 0.378 | 0.204 | 4.29E-05 | 1 |
| Taok1 | 1.54E-09 | 0.177303 | 0.338 | 0.175 | 4.80E-05 | 1 |
| Snap29 | 1.56E-09 | 0.251005 | 0.246 | 0.118 | 4.85E-05 | 1 |
| Cacybp | 1.56E-09 | -0.48993 | 0.16 | 0.3 | 4.85E-05 | 1 |
| Vcpip1 | 1.57E-09 | 0.206455 | 0.212 | 0.093 | 4.87E-05 | 1 |
| Pnpla8 | 1.59E-09 | 0.215285 | 0.341 | 0.177 | 4.93E-05 | 1 |
| Insr | 1.64E-09 | 0.198991 | 0.14 | 0.049 | 5.10E-05 | 1 |
| Ubn2 | 1.69E-09 | 0.21608 | 0.395 | 0.214 | 5.26E-05 | 1 |
| Sltm | 1.71E-09 | 0.228123 | 0.521 | 0.316 | 5.30E-05 | 1 |
| Rnf213 | 1.71E-09 | 0.228405 | 0.181 | 0.073 | 5.32E-05 | 1 |
| Tnrc6a | 1.73E-09 | 0.238135 | 0.433 | 0.246 | 5.37E-05 | 1 |
| Zbtb7a | 1.79E-09 | 0.264241 | 0.404 | 0.231 | 5.55E-05 | 1 |
| Tmf1 | 1.80E-09 | 0.205332 | 0.266 | 0.129 | 5.58E-05 | 1 |
| Helb | 1.94E-09 | 0.180098 | 0.132 | 0.045 | 6.02E-05 | 1 |
| Rif1 | 1.94E-09 | 0.201833 | 0.209 | 0.091 | 6.03E-05 | 1 |
| Cox8a | 1.95E-09 | -0.30017 | 0.779 | 0.77 | 6.05E-05 | 1 |
| Runx1 | 1.97E-09 | 0.189431 | 0.355 | 0.185 | 6.12E-05 | 1 |
| Axin1 | 1.98E-09 | 0.170559 | 0.163 | 0.062 | 6.14E-05 | 1 |
| Rest | 1.99E-09 | 0.232236 | 0.332 | 0.176 | 6.19E-05 | 1 |
| Arid3b | 2.02E-09 | 0.162393 | 0.181 | 0.073 | 6.27E-05 | 1 |
| Pcnt | 2.03E-09 | 0.21322 | 0.175 | 0.07 | 6.32E-05 | 1 |
| Aqr | 2.19E-09 | 0.196228 | 0.275 | 0.134 | 6.80E-05 | 1 |
| Xiap | 2.27E-09 | 0.219921 | 0.327 | 0.171 | 7.06E-05 | 1 |
| Samd9l | 2.32E-09 | 0.251211 | 0.401 | 0.226 | 7.20E-05 | 1 |
| Spopl | 2.32E-09 | 0.16706 | 0.198 | 0.083 | 7.21E-05 | 1 |
| Prkce | 2.34E-09 | 0.206287 | 0.226 | 0.102 | 7.27E-05 | 1 |
| Itgb7 | 2.40E-09 | 0.201198 | 0.264 | 0.127 | 7.47E-05 | 1 |
| Pxk | 2.51E-09 | 0.195894 | 0.516 | 0.294 | 7.79E-05 | 1 |
| Akap9 | 2.52E-09 | 0.245385 | 0.341 | 0.182 | 7.83E-05 | 1 |
| Tmem131l | 2.56E-09 | 0.196455 | 0.393 | 0.214 | 7.95E-05 | 1 |
| Csnk1a1 | 2.59E-09 | 0.224075 | 0.579 | 0.358 | 8.04E-05 | 1 |
| Acss1 | 2.59E-09 | 0.192434 | 0.14 | 0.051 | 8.05E-05 | 1 |
| Creb1 | 2.68E-09 | 0.173041 | 0.312 | 0.156 | 8.33E-05 | 1 |
| Zfc3h1 | 2.76E-09 | 0.214972 | 0.186 | 0.078 | 8.57E-05 | 1 |
| Tfrc | 2.79E-09 | 0.194868 | 0.195 | 0.082 | 8.65E-05 | 1 |
| Gpcpd1 | 2.94E-09 | 0.219723 | 0.441 | 0.253 | 9.13E-05 | 1 |
| Trak2 | 3.02E-09 | 0.192791 | 0.181 | 0.074 | 9.37E-05 | 1 |
| Kcnq1ot1 | 3.04E-09 | 0.469342 | 0.378 | 0.228 | 9.45E-05 | 1 |
| Map3k5 | 3.16E-09 | 0.162377 | 0.143 | 0.052 | 9.81E-05 | 1 |
| Sash3 | 3.22E-09 | 0.212852 | 0.269 | 0.132 | 9.98E-05 | 1 |
| Znrf2 | 3.28E-09 | 0.192652 | 0.298 | 0.148 | 0.000102 | 1 |
| H2-Ob | 3.28E-09 | 0.220617 | 0.785 | 0.535 | 0.000102 | 1 |
| Btbd7 | 3.39E-09 | 0.19515 | 0.284 | 0.14 | 0.000105 | 1 |
| Phf3 | 3.42E-09 | 0.239347 | 0.49 | 0.295 | 0.000106 | 1 |
| Larp4 | 3.42E-09 | 0.192236 | 0.338 | 0.178 | 0.000106 | 1 |
| Arhgap30 | 3.52E-09 | 0.235767 | 0.567 | 0.367 | 0.000109 | 1 |
| Mknk2 | 3.58E-09 | 0.196609 | 0.433 | 0.239 | 0.000111 | 1 |
| Phf12 | 3.62E-09 | 0.152253 | 0.206 | 0.089 | 0.000112 | 1 |
| Sirt1 | 3.68E-09 | 0.223412 | 0.249 | 0.118 | 0.000114 | 1 |
| Pde4b | 3.75E-09 | 0.240453 | 0.653 | 0.431 | 0.000116 | 1 |
| Eif4g3 | 3.76E-09 | 0.187772 | 0.335 | 0.175 | 0.000117 | 1 |
| Pcm1 | 3.81E-09 | 0.204883 | 0.289 | 0.146 | 0.000118 | 1 |
| Tob1 | 3.89E-09 | 0.185458 | 0.229 | 0.103 | 0.000121 | 1 |
| Cenpa | 3.89E-09 | 0.202815 | 0.41 | 0.226 | 0.000121 | 1 |
| Fgd3 | 3.91E-09 | 0.141219 | 0.132 | 0.046 | 0.000121 | 1 |
| Dgka | 4.01E-09 | 0.172561 | 0.241 | 0.11 | 0.000125 | 1 |
| Tcf3 | 4.22E-09 | 0.189467 | 0.292 | 0.146 | 0.000131 | 1 |
| Jak1 | 4.34E-09 | 0.267047 | 0.759 | 0.518 | 0.000135 | 1 |
| Crybg3 | 4.37E-09 | 0.163707 | 0.103 | 0.031 | 0.000136 | 1 |
| Ino80d | 4.40E-09 | 0.19804 | 0.335 | 0.177 | 0.000137 | 1 |
| Stk11 | 4.97E-09 | 0.173167 | 0.284 | 0.14 | 0.000154 | 1 |
| Snx8 | 5.10E-09 | 0.189064 | 0.221 | 0.1 | 0.000158 | 1 |
| Ddb1 | 5.13E-09 | 0.179762 | 0.209 | 0.094 | 0.000159 | 1 |
| Dhx36 | 5.30E-09 | 0.243134 | 0.312 | 0.164 | 0.000164 | 1 |
| Pdcd6ip | 5.52E-09 | 0.187577 | 0.315 | 0.162 | 0.000171 | 1 |
| Csnk2a1 | 5.60E-09 | 0.212449 | 0.361 | 0.198 | 0.000174 | 1 |
| Snx29 | 5.65E-09 | 0.285793 | 0.309 | 0.162 | 0.000176 | 1 |
| Zcchc6 | 5.75E-09 | 0.226547 | 0.539 | 0.337 | 0.000179 | 1 |
| Ice1 | 5.78E-09 | 0.151748 | 0.163 | 0.064 | 0.00018 | 1 |
| Lrmp | 5.79E-09 | 0.195312 | 0.272 | 0.133 | 0.00018 | 1 |
| Rnf145 | 6.05E-09 | 0.194317 | 0.292 | 0.144 | 0.000188 | 1 |
| Tcf25 | 6.10E-09 | 0.235433 | 0.576 | 0.372 | 0.000189 | 1 |
| Lpp | 6.14E-09 | 0.197813 | 0.261 | 0.128 | 0.000191 | 1 |
| Arid4b | 6.43E-09 | 0.244813 | 0.539 | 0.345 | 0.0002 | 1 |
| Uimc1 | 6.44E-09 | 0.165166 | 0.223 | 0.102 | 0.0002 | 1 |
| Rps2 | 6.58E-09 | -0.17082 | 0.997 | 0.997 | 0.000204 | 1 |
| Elf1 | 6.60E-09 | 0.228911 | 0.708 | 0.483 | 0.000205 | 1 |
| Xrn1 | 6.63E-09 | 0.246945 | 0.258 | 0.128 | 0.000206 | 1 |
| Tmem131 | 6.72E-09 | 0.192678 | 0.186 | 0.08 | 0.000209 | 1 |
| Ehmt2 | 6.82E-09 | 0.154739 | 0.189 | 0.081 | 0.000212 | 1 |
| Tfam | 6.83E-09 | 0.236889 | 0.367 | 0.2 | 0.000212 | 1 |
| Wipf1 | 6.97E-09 | 0.215529 | 0.304 | 0.157 | 0.000216 | 1 |
| Dhx38 | 7.18E-09 | 0.169325 | 0.16 | 0.064 | 0.000223 | 1 |
| Dennd4a | 7.26E-09 | 0.210942 | 0.822 | 0.617 | 0.000225 | 1 |
| Bbx | 7.26E-09 | 0.172236 | 0.312 | 0.159 | 0.000225 | 1 |
| Azin1 | 7.30E-09 | 0.194141 | 0.298 | 0.152 | 0.000227 | 1 |
| Topors | 7.50E-09 | 0.196178 | 0.241 | 0.115 | 0.000233 | 1 |
| Klf3 | 7.59E-09 | 0.319691 | 0.398 | 0.233 | 0.000236 | 1 |
| Grk6 | 7.63E-09 | 0.177393 | 0.458 | 0.26 | 0.000237 | 1 |
| Pik3cd | 8.12E-09 | 0.219234 | 0.295 | 0.154 | 0.000252 | 1 |
| Trrap | 8.14E-09 | 0.188283 | 0.221 | 0.102 | 0.000253 | 1 |
| Itpr2 | 8.23E-09 | 0.214358 | 0.321 | 0.171 | 0.000256 | 1 |
| Cpeb4 | 8.30E-09 | 0.174529 | 0.183 | 0.077 | 0.000258 | 1 |
| Sub1 | 8.40E-09 | -0.31391 | 0.825 | 0.816 | 0.000261 | 1 |
| Galnt1 | 8.92E-09 | 0.166412 | 0.309 | 0.161 | 0.000277 | 1 |
| Zfp871 | 9.01E-09 | 0.160874 | 0.295 | 0.148 | 0.00028 | 1 |
| Fbrs | 9.13E-09 | 0.172322 | 0.246 | 0.117 | 0.000284 | 1 |
| Smarca2 | 9.16E-09 | 0.195307 | 0.235 | 0.112 | 0.000284 | 1 |
| Hcfc1 | 9.32E-09 | 0.182417 | 0.183 | 0.08 | 0.000289 | 1 |
| Glul | 9.57E-09 | 0.177383 | 0.232 | 0.108 | 0.000297 | 1 |
| Rapgef1 | 9.64E-09 | 0.189219 | 0.246 | 0.119 | 0.000299 | 1 |
| AI504432 | 9.86E-09 | 0.170553 | 0.183 | 0.077 | 0.000306 | 1 |
| Trim12a | 1.01E-08 | 0.192131 | 0.418 | 0.239 | 0.000313 | 1 |
| Rcsd1 | 1.03E-08 | 0.182262 | 0.556 | 0.335 | 0.000319 | 1 |
| Sf1 | 1.03E-08 | 0.169746 | 0.479 | 0.276 | 0.000319 | 1 |
| Jak2 | 1.03E-08 | 0.190033 | 0.341 | 0.182 | 0.00032 | 1 |
| Fcrl1 | 1.10E-08 | 0.175392 | 0.407 | 0.223 | 0.000341 | 1 |
| Nuak2 | 1.11E-08 | 0.13504 | 0.249 | 0.116 | 0.000346 | 1 |
| Slc16a6 | 1.15E-08 | 0.24956 | 0.198 | 0.09 | 0.000358 | 1 |
| Ddx21 | 1.15E-08 | 0.172024 | 0.438 | 0.256 | 0.000358 | 1 |
| Srcap | 1.20E-08 | 0.181339 | 0.209 | 0.095 | 0.000372 | 1 |
| Rnf144a | 1.20E-08 | 0.18628 | 0.215 | 0.098 | 0.000374 | 1 |
| Ints8 | 1.22E-08 | 0.149348 | 0.112 | 0.037 | 0.000377 | 1 |
| H2-K1 | 1.22E-08 | -0.23401 | 0.954 | 0.952 | 0.000379 | 1 |
| Prpf8 | 1.26E-08 | 0.166581 | 0.315 | 0.164 | 0.000392 | 1 |
| Fancm | 1.29E-08 | 0.144276 | 0.112 | 0.037 | 0.000401 | 1 |
| Dnajc13 | 1.31E-08 | 0.209697 | 0.183 | 0.078 | 0.000405 | 1 |
| Trim56 | 1.33E-08 | 0.208028 | 0.169 | 0.071 | 0.000413 | 1 |
| Dock10 | 1.35E-08 | 0.206811 | 0.524 | 0.327 | 0.000419 | 1 |
| Aida | 1.35E-08 | 0.198327 | 0.215 | 0.1 | 0.000419 | 1 |
| Ppp1r12a | 1.35E-08 | 0.181195 | 0.424 | 0.243 | 0.000421 | 1 |
| Dido1 | 1.36E-08 | 0.171149 | 0.238 | 0.114 | 0.000422 | 1 |
| Kmt2d | 1.37E-08 | 0.21158 | 0.232 | 0.11 | 0.000425 | 1 |
| Ing3 | 1.39E-08 | 0.139808 | 0.201 | 0.089 | 0.000433 | 1 |
| Klf2 | 1.45E-08 | 0.226507 | 0.742 | 0.518 | 0.000449 | 1 |
| Reps1 | 1.45E-08 | 0.14982 | 0.126 | 0.044 | 0.00045 | 1 |
| Kdm7a | 1.45E-08 | 0.228109 | 0.544 | 0.345 | 0.000451 | 1 |
| Smc3 | 1.50E-08 | 0.186246 | 0.367 | 0.205 | 0.000466 | 1 |
| Chd9 | 1.55E-08 | 0.200185 | 0.278 | 0.142 | 0.00048 | 1 |
| Chd1 | 1.58E-08 | 0.191323 | 0.324 | 0.172 | 0.00049 | 1 |
| March1 | 1.58E-08 | 0.209617 | 0.456 | 0.27 | 0.000491 | 1 |
| Eif4b | 1.59E-08 | 0.194412 | 0.393 | 0.221 | 0.000493 | 1 |
| Mob1a | 1.60E-08 | 0.169283 | 0.404 | 0.227 | 0.000497 | 1 |
| Mdn1 | 1.63E-08 | 0.249814 | 0.206 | 0.096 | 0.000505 | 1 |
| Med13l | 1.73E-08 | 0.209018 | 0.287 | 0.15 | 0.000537 | 1 |
| Spp1 | 1.76E-08 | -0.36902 | 0.02 | 0.119 | 0.000547 | 1 |
| Adipor2 | 1.79E-08 | 0.204508 | 0.232 | 0.112 | 0.000556 | 1 |
| Grn | 1.79E-08 | -0.40187 | 0.054 | 0.168 | 0.000557 | 1 |
| Larp4b | 1.83E-08 | 0.194078 | 0.315 | 0.171 | 0.00057 | 1 |
| Il21r | 1.85E-08 | 0.217696 | 0.269 | 0.137 | 0.000575 | 1 |
| Clock | 1.95E-08 | 0.15377 | 0.163 | 0.067 | 0.000605 | 1 |
| Rc3h1 | 1.96E-08 | 0.176527 | 0.269 | 0.135 | 0.00061 | 1 |
| Xylt1 | 1.99E-08 | 0.179507 | 0.264 | 0.13 | 0.000618 | 1 |
| Stk4 | 2.07E-08 | 0.245485 | 0.41 | 0.245 | 0.000642 | 1 |
| Zfp800 | 2.11E-08 | 0.166722 | 0.246 | 0.119 | 0.000655 | 1 |
| Cpsf7 | 2.14E-08 | 0.166919 | 0.223 | 0.105 | 0.000666 | 1 |
| Irf8 | 2.15E-08 | 0.193892 | 0.55 | 0.34 | 0.000668 | 1 |
| Zdhhc20 | 2.22E-08 | 0.15027 | 0.335 | 0.179 | 0.00069 | 1 |
| Carhsp1 | 2.28E-08 | 0.152339 | 0.143 | 0.056 | 0.000707 | 1 |
| Rps15 | 2.32E-08 | -0.14006 | 1 | 0.998 | 0.00072 | 1 |
| Rps6kb1 | 2.39E-08 | 0.17424 | 0.338 | 0.183 | 0.000744 | 1 |
| Gm20696 | 2.47E-08 | 0.159085 | 0.275 | 0.137 | 0.000766 | 1 |
| Lrrc8c | 2.50E-08 | 0.171405 | 0.166 | 0.07 | 0.000775 | 1 |
| Uso1 | 2.56E-08 | 0.174302 | 0.195 | 0.089 | 0.000796 | 1 |
| Baz1a | 2.74E-08 | 0.213874 | 0.527 | 0.331 | 0.000852 | 1 |
| Eef1b2 | 2.82E-08 | -0.20396 | 0.963 | 0.932 | 0.000874 | 1 |
| Bclaf1 | 2.82E-08 | 0.191599 | 0.633 | 0.404 | 0.000875 | 1 |
| Ap1ar | 2.83E-08 | 0.142644 | 0.146 | 0.057 | 0.000878 | 1 |
| Fam208a | 2.86E-08 | 0.175493 | 0.401 | 0.228 | 0.000887 | 1 |
| Snx9 | 2.92E-08 | 0.158223 | 0.318 | 0.168 | 0.000907 | 1 |
| mt-Atp8 | 2.93E-08 | 0.203008 | 0.447 | 0.268 | 0.000911 | 1 |
| Per1 | 3.05E-08 | 0.152045 | 0.384 | 0.21 | 0.000948 | 1 |
| Cblb | 3.05E-08 | 0.214701 | 0.387 | 0.225 | 0.000948 | 1 |
| Gm26917 | 3.07E-08 | 0.156583 | 0.536 | 0.332 | 0.000952 | 1 |
| Fxyd5 | 3.12E-08 | -0.47807 | 0.244 | 0.356 | 0.000968 | 1 |
| Ss18 | 3.18E-08 | 0.180954 | 0.192 | 0.087 | 0.000989 | 1 |
| H2-DMb2 | 3.22E-08 | 0.192108 | 0.782 | 0.56 | 0.000999 | 1 |
| Smc4 | 3.39E-08 | 0.262824 | 0.295 | 0.16 | 0.001052 | 1 |
| Man2a1 | 3.48E-08 | 0.191849 | 0.272 | 0.144 | 0.00108 | 1 |
| Rpl22l1 | 3.50E-08 | -0.29971 | 0.802 | 0.791 | 0.001086 | 1 |
| Phc3 | 3.56E-08 | 0.156972 | 0.189 | 0.084 | 0.001107 | 1 |
| Selenot | 3.81E-08 | 0.191458 | 0.321 | 0.175 | 0.001183 | 1 |
| Gak | 3.97E-08 | 0.150196 | 0.181 | 0.078 | 0.001233 | 1 |
| Golga5 | 3.97E-08 | 0.176653 | 0.129 | 0.049 | 0.001233 | 1 |
| Trim26 | 4.02E-08 | 0.175423 | 0.255 | 0.126 | 0.001248 | 1 |
| Mark2 | 4.04E-08 | 0.186085 | 0.364 | 0.202 | 0.001255 | 1 |
| Mbnl1 | 4.05E-08 | 0.205838 | 0.819 | 0.604 | 0.001257 | 1 |
| Zfp646 | 4.10E-08 | 0.167488 | 0.221 | 0.105 | 0.001272 | 1 |
| Dnm2 | 4.14E-08 | 0.165403 | 0.304 | 0.161 | 0.001285 | 1 |
| Nek7 | 4.14E-08 | 0.201492 | 0.307 | 0.165 | 0.001286 | 1 |
| Prpf39 | 4.16E-08 | 0.192316 | 0.324 | 0.174 | 0.001291 | 1 |
| Zfp644 | 4.28E-08 | 0.212626 | 0.395 | 0.23 | 0.00133 | 1 |
| Ktn1 | 4.42E-08 | 0.212121 | 0.344 | 0.194 | 0.001374 | 1 |
| Dck | 4.44E-08 | 0.156699 | 0.178 | 0.077 | 0.00138 | 1 |
| Slc10a7 | 4.53E-08 | 0.139456 | 0.117 | 0.042 | 0.001408 | 1 |
| Rassf5 | 4.77E-08 | 0.163979 | 0.212 | 0.101 | 0.001482 | 1 |
| Cdk19 | 4.84E-08 | 0.159819 | 0.203 | 0.092 | 0.001502 | 1 |
| Ireb2 | 4.86E-08 | 0.167658 | 0.223 | 0.107 | 0.001508 | 1 |
| Gpr174 | 4.89E-08 | 0.21483 | 0.264 | 0.136 | 0.001517 | 1 |
| Rsbn1 | 4.90E-08 | 0.175095 | 0.301 | 0.16 | 0.001521 | 1 |
| Ptpn18 | 4.92E-08 | -0.34602 | 0.645 | 0.646 | 0.001528 | 1 |
| Dcaf11 | 5.03E-08 | 0.140524 | 0.172 | 0.074 | 0.001562 | 1 |
| Phf20l1 | 5.07E-08 | 0.174373 | 0.444 | 0.254 | 0.001573 | 1 |
| Eif4g1 | 5.07E-08 | 0.192542 | 0.327 | 0.18 | 0.001575 | 1 |
| Htt | 5.19E-08 | 0.16657 | 0.16 | 0.069 | 0.001611 | 1 |
| Gpatch8 | 5.21E-08 | 0.173612 | 0.358 | 0.197 | 0.001618 | 1 |
| Ccnk | 5.25E-08 | 0.17199 | 0.14 | 0.056 | 0.001632 | 1 |
| Slu7 | 5.26E-08 | 0.195492 | 0.218 | 0.105 | 0.001632 | 1 |
| Gsk3a | 5.30E-08 | 0.158274 | 0.215 | 0.101 | 0.001645 | 1 |
| Chd6 | 5.32E-08 | 0.212445 | 0.367 | 0.207 | 0.001651 | 1 |
| Btla | 5.40E-08 | 0.145855 | 0.513 | 0.309 | 0.001678 | 1 |
| Ctcf | 5.48E-08 | 0.168825 | 0.519 | 0.313 | 0.001702 | 1 |
| Depdc5 | 5.89E-08 | 0.177703 | 0.132 | 0.051 | 0.001829 | 1 |
| Hnrnpm | 5.93E-08 | 0.179457 | 0.487 | 0.293 | 0.001841 | 1 |
| Slc7a1 | 6.01E-08 | 0.20176 | 0.186 | 0.087 | 0.001866 | 1 |
| Cbx4 | 6.04E-08 | 0.1827 | 0.223 | 0.108 | 0.001875 | 1 |
| Foxn3 | 6.10E-08 | 0.198072 | 0.564 | 0.362 | 0.001895 | 1 |
| Rbck1 | 6.11E-08 | 0.182232 | 0.215 | 0.105 | 0.001897 | 1 |
| Zfp106 | 6.17E-08 | 0.217651 | 0.427 | 0.256 | 0.001915 | 1 |
| Rasgrp2 | 6.25E-08 | 0.172201 | 0.527 | 0.322 | 0.001942 | 1 |
| Pkn1 | 6.27E-08 | 0.190463 | 0.393 | 0.23 | 0.001946 | 1 |
| Khdrbs1 | 6.27E-08 | 0.128018 | 0.427 | 0.241 | 0.001946 | 1 |
| Fos | 6.33E-08 | -0.41257 | 0.533 | 0.614 | 0.001964 | 1 |
| Trp53bp1 | 6.38E-08 | 0.157633 | 0.123 | 0.045 | 0.001981 | 1 |
| Ltn1 | 6.45E-08 | 0.155387 | 0.135 | 0.052 | 0.002004 | 1 |
| Pdcl | 6.49E-08 | 0.144058 | 0.129 | 0.049 | 0.002016 | 1 |
| Pfdn5 | 6.54E-08 | -0.26337 | 0.848 | 0.82 | 0.00203 | 1 |
| Bcl11a | 6.74E-08 | 0.212047 | 0.473 | 0.289 | 0.002093 | 1 |
| Tnk2 | 6.76E-08 | 0.130407 | 0.109 | 0.037 | 0.002099 | 1 |
| Ing1 | 6.88E-08 | 0.151683 | 0.315 | 0.169 | 0.002138 | 1 |
| Prkdc | 6.89E-08 | 0.165802 | 0.103 | 0.035 | 0.002139 | 1 |
| Rbl2 | 6.91E-08 | 0.168675 | 0.186 | 0.084 | 0.002144 | 1 |
| Ssh2 | 7.01E-08 | 0.180327 | 0.771 | 0.561 | 0.002178 | 1 |
| Phf21a | 7.04E-08 | 0.13486 | 0.246 | 0.121 | 0.002185 | 1 |
| Pum1 | 7.14E-08 | 0.168121 | 0.364 | 0.206 | 0.002218 | 1 |
| Gdi2 | 7.15E-08 | 0.208574 | 0.831 | 0.636 | 0.00222 | 1 |
| Ppp4r3b | 7.19E-08 | 0.159246 | 0.413 | 0.244 | 0.002232 | 1 |
| Pabpc1 | 7.25E-08 | 0.224286 | 0.891 | 0.691 | 0.002252 | 1 |
| AC149090.1 | 7.29E-08 | 0.226384 | 0.324 | 0.181 | 0.002265 | 1 |
| Tob2 | 7.44E-08 | 0.179708 | 0.579 | 0.372 | 0.002311 | 1 |
| Mgat1 | 7.51E-08 | 0.191803 | 0.203 | 0.098 | 0.002331 | 1 |
| Clcn3 | 7.59E-08 | 0.22291 | 0.258 | 0.137 | 0.002356 | 1 |
| Impad1 | 7.64E-08 | 0.139996 | 0.115 | 0.041 | 0.002372 | 1 |
| Sod2 | 7.81E-08 | -0.41287 | 0.181 | 0.298 | 0.002426 | 1 |
| Acap2 | 7.93E-08 | 0.162694 | 0.215 | 0.103 | 0.002464 | 1 |
| Herpud2 | 7.93E-08 | 0.150535 | 0.195 | 0.089 | 0.002464 | 1 |
| Ppp1r15b | 8.14E-08 | 0.175928 | 0.206 | 0.099 | 0.002527 | 1 |
| Kif5b | 8.18E-08 | 0.209412 | 0.524 | 0.323 | 0.00254 | 1 |
| Stag2 | 8.21E-08 | 0.147943 | 0.372 | 0.209 | 0.00255 | 1 |
| Gmip | 8.41E-08 | 0.157848 | 0.272 | 0.143 | 0.002612 | 1 |
| Riok1 | 8.44E-08 | 0.159425 | 0.215 | 0.104 | 0.00262 | 1 |
| Rere | 8.52E-08 | 0.194456 | 0.261 | 0.137 | 0.002646 | 1 |
| Papd5 | 8.56E-08 | 0.174103 | 0.146 | 0.06 | 0.002657 | 1 |
| Nfat5 | 8.76E-08 | 0.180073 | 0.458 | 0.282 | 0.00272 | 1 |
| Cpt1a | 8.90E-08 | 0.14184 | 0.138 | 0.054 | 0.002765 | 1 |
| Ifitm3 | 8.92E-08 | -0.3046 | 0.023 | 0.117 | 0.00277 | 1 |
| Akna | 9.28E-08 | 0.190587 | 0.304 | 0.167 | 0.002881 | 1 |
| Pcf11 | 9.28E-08 | 0.209511 | 0.413 | 0.244 | 0.002882 | 1 |
| Map3k3 | 9.42E-08 | 0.143893 | 0.149 | 0.061 | 0.002925 | 1 |
| Setbp1 | 9.64E-08 | 0.175376 | 0.146 | 0.06 | 0.002992 | 1 |
| Kdm1a | 9.70E-08 | 0.13851 | 0.264 | 0.134 | 0.003011 | 1 |
| Ccar1 | 9.78E-08 | 0.160005 | 0.418 | 0.243 | 0.003038 | 1 |
| Gcn1l1 | 9.94E-08 | 0.158241 | 0.198 | 0.092 | 0.003087 | 1 |
| Smad7 | 1.01E-07 | 0.229762 | 0.476 | 0.302 | 0.003131 | 1 |
| Kdm3b | 1.01E-07 | 0.17551 | 0.198 | 0.092 | 0.003132 | 1 |
| Rtf1 | 1.02E-07 | 0.160718 | 0.427 | 0.25 | 0.003162 | 1 |
| Stt3b | 1.02E-07 | 0.138828 | 0.39 | 0.221 | 0.003182 | 1 |
| Bcl7a | 1.04E-07 | 0.232479 | 0.175 | 0.079 | 0.003235 | 1 |
| Scap | 1.05E-07 | 0.138284 | 0.12 | 0.045 | 0.003257 | 1 |
| Inpp5d | 1.05E-07 | 0.191367 | 0.648 | 0.435 | 0.003269 | 1 |
| Sipa1l1 | 1.06E-07 | 0.18263 | 0.272 | 0.144 | 0.003295 | 1 |
| Zfp24 | 1.07E-07 | 0.130674 | 0.126 | 0.047 | 0.003334 | 1 |
| Synrg | 1.08E-07 | 0.173632 | 0.221 | 0.107 | 0.003357 | 1 |
| Hsp90aa1 | 1.08E-07 | -0.34799 | 0.817 | 0.807 | 0.003357 | 1 |
| Etv3 | 1.09E-07 | 0.198866 | 0.229 | 0.116 | 0.003381 | 1 |
| Tnpo1 | 1.10E-07 | 0.153365 | 0.281 | 0.147 | 0.003414 | 1 |
| Lsp1 | 1.11E-07 | 0.194329 | 0.768 | 0.534 | 0.003434 | 1 |
| Dapp1 | 1.11E-07 | 0.182994 | 0.33 | 0.184 | 0.003447 | 1 |
| Akt3 | 1.12E-07 | 0.155062 | 0.143 | 0.058 | 0.003465 | 1 |
| Senp2 | 1.12E-07 | 0.139255 | 0.335 | 0.182 | 0.003487 | 1 |
| Tk2 | 1.13E-07 | 0.113353 | 0.126 | 0.047 | 0.003513 | 1 |
| Tbl1xr1 | 1.13E-07 | 0.164631 | 0.249 | 0.128 | 0.003517 | 1 |
| Txndc11 | 1.14E-07 | 0.169285 | 0.238 | 0.12 | 0.003528 | 1 |
| Gon4l | 1.14E-07 | 0.174594 | 0.192 | 0.089 | 0.003541 | 1 |
| Skiv2l | 1.14E-07 | 0.132273 | 0.126 | 0.048 | 0.003543 | 1 |
| Ewsr1 | 1.15E-07 | 0.217141 | 0.37 | 0.222 | 0.003564 | 1 |
| Arhgap15 | 1.19E-07 | 0.199379 | 0.693 | 0.478 | 0.003696 | 1 |
| Ints1 | 1.21E-07 | 0.156802 | 0.117 | 0.044 | 0.00377 | 1 |
| Ubr2 | 1.25E-07 | 0.141297 | 0.266 | 0.139 | 0.003874 | 1 |
| Limd1 | 1.26E-07 | 0.172091 | 0.413 | 0.238 | 0.003909 | 1 |
| Smarca4 | 1.27E-07 | 0.145295 | 0.255 | 0.131 | 0.00395 | 1 |
| Sec61g | 1.29E-07 | -0.35786 | 0.544 | 0.604 | 0.004011 | 1 |
| Arih2 | 1.31E-07 | 0.159758 | 0.203 | 0.096 | 0.004058 | 1 |
| Gatad2b | 1.33E-07 | 0.19863 | 0.45 | 0.28 | 0.004144 | 1 |
| Rps6ka5 | 1.35E-07 | 0.180588 | 0.158 | 0.067 | 0.004186 | 1 |
| Hipk1 | 1.37E-07 | 0.178397 | 0.43 | 0.259 | 0.004246 | 1 |
| Gabarap | 1.38E-07 | -0.33296 | 0.564 | 0.59 | 0.004271 | 1 |
| Usp8 | 1.40E-07 | 0.158289 | 0.183 | 0.083 | 0.004333 | 1 |
| Dennd6a | 1.40E-07 | 0.162576 | 0.189 | 0.088 | 0.004361 | 1 |
| Nup50 | 1.42E-07 | 0.142187 | 0.192 | 0.089 | 0.004396 | 1 |
| Cdc73 | 1.43E-07 | 0.137975 | 0.223 | 0.108 | 0.004426 | 1 |
| Ehd4 | 1.43E-07 | 0.158683 | 0.166 | 0.073 | 0.004426 | 1 |
| Canx | 1.45E-07 | 0.219683 | 0.441 | 0.272 | 0.004498 | 1 |
| Gls | 1.45E-07 | 0.212827 | 0.536 | 0.35 | 0.004518 | 1 |
| Hmox1 | 1.46E-07 | -0.31831 | 0.02 | 0.11 | 0.004527 | 1 |
| Ppp2r2a | 1.48E-07 | 0.137387 | 0.384 | 0.217 | 0.004607 | 1 |
| Thrap3 | 1.52E-07 | 0.16863 | 0.484 | 0.296 | 0.004712 | 1 |
| Fem1c | 1.52E-07 | 0.15527 | 0.152 | 0.064 | 0.004734 | 1 |
| Ano6 | 1.53E-07 | 0.107518 | 0.14 | 0.056 | 0.004749 | 1 |
| Gigyf1 | 1.54E-07 | 0.142111 | 0.269 | 0.139 | 0.004794 | 1 |
| Gdi1 | 1.56E-07 | 0.12977 | 0.181 | 0.082 | 0.004847 | 1 |
| Fbxl12 | 1.58E-07 | 0.096849 | 0.212 | 0.098 | 0.004914 | 1 |
| Inpp4b | 1.62E-07 | 0.185465 | 0.123 | 0.048 | 0.005038 | 1 |
| Ncoa2 | 1.71E-07 | 0.163933 | 0.221 | 0.11 | 0.005304 | 1 |
| March6 | 1.71E-07 | 0.150622 | 0.163 | 0.071 | 0.005311 | 1 |
| Bcl2a1b | 1.75E-07 | -0.33636 | 0.223 | 0.348 | 0.00542 | 1 |
| Csnk1g3 | 1.81E-07 | 0.165575 | 0.467 | 0.287 | 0.005632 | 1 |
| Wipf2 | 1.90E-07 | 0.145228 | 0.172 | 0.076 | 0.005901 | 1 |
| Sacs | 1.92E-07 | 0.139557 | 0.152 | 0.065 | 0.005947 | 1 |
| Traf5 | 1.93E-07 | 0.155855 | 0.244 | 0.123 | 0.005989 | 1 |
| Lrpprc | 1.97E-07 | 0.128982 | 0.117 | 0.044 | 0.006131 | 1 |
| Zfp638 | 1.98E-07 | 0.174213 | 0.352 | 0.2 | 0.006138 | 1 |
| Crkl | 1.98E-07 | 0.136198 | 0.14 | 0.058 | 0.006151 | 1 |
| Atox1 | 1.99E-07 | -0.42138 | 0.201 | 0.315 | 0.006188 | 1 |
| Glyr1 | 2.02E-07 | 0.164727 | 0.301 | 0.164 | 0.006274 | 1 |
| Atf6 | 2.02E-07 | 0.20593 | 0.226 | 0.117 | 0.006279 | 1 |
| Qrich1 | 2.03E-07 | 0.149051 | 0.218 | 0.108 | 0.006288 | 1 |
| Srsf6 | 2.03E-07 | 0.200677 | 0.433 | 0.266 | 0.006317 | 1 |
| Lnx2 | 2.05E-07 | 0.132925 | 0.106 | 0.037 | 0.006371 | 1 |
| Fcrla | 2.10E-07 | 0.166592 | 0.585 | 0.375 | 0.00653 | 1 |
| Scaf8 | 2.14E-07 | 0.144888 | 0.189 | 0.088 | 0.006657 | 1 |
| Mkln1 | 2.20E-07 | 0.205346 | 0.364 | 0.212 | 0.006839 | 1 |
| Banp | 2.21E-07 | 0.140691 | 0.138 | 0.056 | 0.006847 | 1 |
| Puf60 | 2.29E-07 | 0.159277 | 0.37 | 0.215 | 0.007105 | 1 |
| Arid1a | 2.36E-07 | 0.180927 | 0.473 | 0.285 | 0.007319 | 1 |
| Uhmk1 | 2.38E-07 | 0.128756 | 0.189 | 0.088 | 0.007377 | 1 |
| Inpp5k | 2.40E-07 | 0.135872 | 0.192 | 0.091 | 0.007464 | 1 |
| Ylpm1 | 2.42E-07 | 0.18601 | 0.195 | 0.094 | 0.007523 | 1 |
| Nrip1 | 2.45E-07 | 0.162783 | 0.198 | 0.097 | 0.007601 | 1 |
| Ndrg3 | 2.47E-07 | 0.110843 | 0.109 | 0.039 | 0.007685 | 1 |
| Tln1 | 2.49E-07 | 0.175441 | 0.504 | 0.322 | 0.007723 | 1 |
| Rel | 2.58E-07 | 0.276839 | 0.862 | 0.695 | 0.008016 | 1 |
| Rexo1 | 2.60E-07 | 0.156153 | 0.246 | 0.126 | 0.008062 | 1 |
| Pcmtd2 | 2.62E-07 | 0.177174 | 0.178 | 0.082 | 0.008134 | 1 |
| Wac | 2.69E-07 | 0.17318 | 0.421 | 0.256 | 0.008342 | 1 |
| Fbxo11 | 2.74E-07 | 0.115421 | 0.358 | 0.196 | 0.008499 | 1 |
| Arhgef7 | 2.79E-07 | 0.132373 | 0.115 | 0.043 | 0.008675 | 1 |
| Lst1 | 2.80E-07 | -0.36013 | 0.032 | 0.123 | 0.008701 | 1 |
| Csrp2 | 2.82E-07 | 0.151179 | 0.123 | 0.047 | 0.008754 | 1 |
| Senp6 | 2.84E-07 | 0.168782 | 0.375 | 0.219 | 0.008826 | 1 |
| Mob1b | 2.87E-07 | 0.123788 | 0.175 | 0.08 | 0.008908 | 1 |
| 1700020I14Rik | 2.88E-07 | 0.194292 | 0.35 | 0.203 | 0.008946 | 1 |
| Fbxo33 | 2.89E-07 | 0.142488 | 0.206 | 0.1 | 0.008978 | 1 |
| Ibtk | 2.95E-07 | 0.15099 | 0.189 | 0.089 | 0.009158 | 1 |
| Kmo | 2.99E-07 | 0.132419 | 0.123 | 0.048 | 0.009289 | 1 |
| Nisch | 3.01E-07 | 0.211552 | 0.347 | 0.2 | 0.009345 | 1 |
| Uvrag | 3.04E-07 | 0.119242 | 0.372 | 0.212 | 0.009426 | 1 |
| Eprs | 3.11E-07 | 0.22471 | 0.341 | 0.2 | 0.009666 | 1 |
| Rbbp6 | 3.12E-07 | 0.150742 | 0.602 | 0.386 | 0.009695 | 1 |
| Aggf1 | 3.19E-07 | 0.165839 | 0.166 | 0.076 | 0.009915 | 1 |
| Ubqln2 | 3.20E-07 | 0.170698 | 0.103 | 0.037 | 0.009926 | 1 |
| Zfr | 3.20E-07 | 0.155959 | 0.238 | 0.123 | 0.009927 | 1 |
| Dnm1l | 3.24E-07 | 0.148042 | 0.186 | 0.088 | 0.010071 | 1 |
| Tcp11l2 | 3.25E-07 | 0.143195 | 0.539 | 0.34 | 0.010087 | 1 |
| Smg5 | 3.30E-07 | 0.136439 | 0.181 | 0.083 | 0.010252 | 1 |
| Smc5 | 3.35E-07 | 0.141352 | 0.16 | 0.07 | 0.010406 | 1 |
| Mepce | 3.36E-07 | 0.116214 | 0.301 | 0.16 | 0.010447 | 1 |
| Tapbp | 3.41E-07 | 0.140935 | 0.45 | 0.268 | 0.010596 | 1 |
| Cep135 | 3.42E-07 | 0.116012 | 0.115 | 0.043 | 0.010612 | 1 |
| Gapdh | 3.44E-07 | -0.3029 | 0.754 | 0.711 | 0.010684 | 1 |
| Copb1 | 3.46E-07 | 0.18161 | 0.304 | 0.171 | 0.010754 | 1 |
| Cap1 | 3.49E-07 | 0.200128 | 0.289 | 0.163 | 0.010829 | 1 |
| Hivep2 | 3.53E-07 | 0.206848 | 0.212 | 0.107 | 0.010963 | 1 |
| Sh2b3 | 3.57E-07 | 0.155298 | 0.33 | 0.185 | 0.011087 | 1 |
| Phf14 | 3.66E-07 | 0.180038 | 0.275 | 0.15 | 0.011366 | 1 |
| Atrx | 3.67E-07 | 0.206533 | 0.544 | 0.363 | 0.01139 | 1 |
| Trp53inp2 | 3.69E-07 | 0.150465 | 0.135 | 0.055 | 0.011459 | 1 |
| Dennd5a | 3.70E-07 | 0.161627 | 0.181 | 0.085 | 0.011494 | 1 |
| Csnk1d | 3.72E-07 | 0.135759 | 0.229 | 0.115 | 0.011542 | 1 |
| Hyou1 | 3.77E-07 | 0.138669 | 0.103 | 0.037 | 0.011695 | 1 |
| Rybp | 3.78E-07 | 0.13737 | 0.266 | 0.138 | 0.011728 | 1 |
| Pbrm1 | 3.81E-07 | 0.188015 | 0.43 | 0.27 | 0.011831 | 1 |
| 5031439G07Rik | 3.82E-07 | 0.186477 | 0.172 | 0.081 | 0.011872 | 1 |
| Ercc6 | 3.86E-07 | 0.118509 | 0.109 | 0.04 | 0.011974 | 1 |
| Ccnt1 | 3.88E-07 | 0.144608 | 0.381 | 0.221 | 0.012054 | 1 |
| Rgs2 | 3.89E-07 | 0.229463 | 0.458 | 0.293 | 0.012077 | 1 |
| Pik3r1 | 3.93E-07 | 0.152242 | 0.264 | 0.141 | 0.012211 | 1 |
| Trpm7 | 3.94E-07 | 0.190188 | 0.275 | 0.151 | 0.012237 | 1 |
| Fry | 3.96E-07 | 0.150062 | 0.198 | 0.096 | 0.012282 | 1 |
| Maml2 | 4.03E-07 | 0.119843 | 0.106 | 0.038 | 0.012526 | 1 |
| Tor1aip2 | 4.14E-07 | 0.158856 | 0.186 | 0.089 | 0.012845 | 1 |
| Map4 | 4.29E-07 | 0.181881 | 0.178 | 0.085 | 0.01332 | 1 |
| B3gnt5 | 4.37E-07 | 0.14907 | 0.327 | 0.185 | 0.013564 | 1 |
| Ikzf5 | 4.40E-07 | 0.143716 | 0.16 | 0.072 | 0.013669 | 1 |
| Nudt3 | 4.43E-07 | 0.127713 | 0.16 | 0.072 | 0.013763 | 1 |
| Zfp318 | 4.47E-07 | 0.170017 | 0.504 | 0.312 | 0.01388 | 1 |
| Cat | 4.50E-07 | 0.164217 | 0.212 | 0.106 | 0.013967 | 1 |
| Mbtd1 | 4.58E-07 | 0.184734 | 0.249 | 0.131 | 0.014234 | 1 |
| Rabgap1 | 4.63E-07 | 0.176043 | 0.16 | 0.073 | 0.014387 | 1 |
| Hspa4 | 4.69E-07 | 0.189748 | 0.378 | 0.229 | 0.014577 | 1 |
| Cyb5r4 | 4.73E-07 | 0.140846 | 0.181 | 0.085 | 0.014679 | 1 |
| Chfr | 4.76E-07 | 0.15974 | 0.221 | 0.112 | 0.014791 | 1 |
| Zmynd8 | 4.80E-07 | 0.160707 | 0.284 | 0.159 | 0.014911 | 1 |
| Cers4 | 5.01E-07 | 0.166867 | 0.206 | 0.102 | 0.015544 | 1 |
| Srebf2 | 5.01E-07 | 0.133873 | 0.284 | 0.155 | 0.015572 | 1 |
| Ggnbp2 | 5.07E-07 | 0.138214 | 0.352 | 0.202 | 0.015754 | 1 |
| Ifi209 | 5.17E-07 | 0.204123 | 0.436 | 0.27 | 0.016066 | 1 |
| Ddx58 | 5.24E-07 | 0.155509 | 0.143 | 0.062 | 0.016277 | 1 |
| Gng5 | 5.24E-07 | -0.34428 | 0.599 | 0.599 | 0.016278 | 1 |
| Tpp2 | 5.25E-07 | 0.136 | 0.321 | 0.18 | 0.016312 | 1 |
| Rasgef1b | 5.39E-07 | 0.202168 | 0.289 | 0.159 | 0.016735 | 1 |
| Rock2 | 5.40E-07 | 0.151962 | 0.404 | 0.241 | 0.016775 | 1 |
| Ptpn22 | 5.41E-07 | -0.43139 | 0.261 | 0.365 | 0.0168 | 1 |
| Fnbp4 | 5.43E-07 | 0.164527 | 0.292 | 0.163 | 0.016848 | 1 |
| Slc9a8 | 5.52E-07 | 0.138228 | 0.138 | 0.058 | 0.017145 | 1 |
| Dcaf12 | 5.57E-07 | 0.121914 | 0.169 | 0.077 | 0.017311 | 1 |
| Rabep2 | 5.58E-07 | 0.139793 | 0.229 | 0.118 | 0.01733 | 1 |
| Uggt1 | 5.75E-07 | 0.149638 | 0.181 | 0.086 | 0.017859 | 1 |
| Usp4 | 5.78E-07 | 0.145884 | 0.178 | 0.083 | 0.01796 | 1 |
| Rassf2 | 5.84E-07 | 0.166362 | 0.301 | 0.168 | 0.018138 | 1 |
| Bin2 | 5.85E-07 | 0.165526 | 0.244 | 0.129 | 0.018179 | 1 |
| Pds5a | 6.12E-07 | 0.158944 | 0.338 | 0.195 | 0.018992 | 1 |
| Usp25 | 6.16E-07 | 0.14389 | 0.261 | 0.139 | 0.01913 | 1 |
| Cytip | 6.17E-07 | 0.178911 | 0.874 | 0.746 | 0.019145 | 1 |
| Cox6c | 6.32E-07 | -0.33299 | 0.602 | 0.613 | 0.019611 | 1 |
| Ranbp2 | 6.35E-07 | 0.182705 | 0.453 | 0.28 | 0.019723 | 1 |
| Apbb1ip | 6.37E-07 | 0.200402 | 0.433 | 0.273 | 0.019772 | 1 |
| Map3k14 | 6.38E-07 | 0.078871 | 0.112 | 0.042 | 0.01982 | 1 |
| Card11 | 6.45E-07 | 0.181818 | 0.244 | 0.131 | 0.020017 | 1 |
| Tifa | 6.56E-07 | 0.219244 | 0.106 | 0.04 | 0.020377 | 1 |
| Akap11 | 6.56E-07 | 0.160234 | 0.126 | 0.051 | 0.020386 | 1 |
| Add1 | 6.78E-07 | 0.195424 | 0.266 | 0.148 | 0.02106 | 1 |
| Brd8 | 6.80E-07 | 0.190524 | 0.315 | 0.18 | 0.021115 | 1 |
| Sec16a | 6.81E-07 | 0.132953 | 0.155 | 0.069 | 0.021147 | 1 |
| Ms4a1 | 7.00E-07 | 0.257888 | 0.854 | 0.693 | 0.021723 | 1 |
| Arhgap17 | 7.04E-07 | 0.179691 | 0.418 | 0.255 | 0.021856 | 1 |
| Mif | 7.07E-07 | -0.44613 | 0.364 | 0.433 | 0.021951 | 1 |
| Uba1 | 7.14E-07 | 0.124844 | 0.241 | 0.126 | 0.022184 | 1 |
| AI314180 | 7.16E-07 | 0.1446 | 0.203 | 0.101 | 0.022231 | 1 |
| Kdm6a | 7.24E-07 | 0.166153 | 0.158 | 0.073 | 0.022486 | 1 |
| Usf3 | 7.31E-07 | 0.145578 | 0.181 | 0.086 | 0.022708 | 1 |
| Eif1 | 7.38E-07 | -0.15978 | 0.994 | 0.985 | 0.022926 | 1 |
| Sgms1 | 7.40E-07 | 0.179045 | 0.372 | 0.221 | 0.022971 | 1 |
| Golgb1 | 7.43E-07 | 0.194816 | 0.209 | 0.107 | 0.023058 | 1 |
| Dcaf8 | 7.57E-07 | 0.16645 | 0.295 | 0.168 | 0.023496 | 1 |
| Wdr26 | 7.62E-07 | 0.171745 | 0.413 | 0.255 | 0.023677 | 1 |
| Eps15 | 7.67E-07 | 0.156883 | 0.226 | 0.118 | 0.023825 | 1 |
| Ccnt2 | 7.70E-07 | 0.141491 | 0.264 | 0.142 | 0.023913 | 1 |
| Rpl7a | 7.73E-07 | -0.18296 | 0.966 | 0.95 | 0.023997 | 1 |
| Ankrd17 | 8.05E-07 | 0.142312 | 0.436 | 0.267 | 0.024999 | 1 |
| Slc28a2 | 8.21E-07 | 0.125349 | 0.155 | 0.069 | 0.025503 | 1 |
| Arid5a | 8.57E-07 | 0.166253 | 0.507 | 0.322 | 0.026604 | 1 |
| Anapc1 | 8.71E-07 | 0.205006 | 0.155 | 0.071 | 0.027037 | 1 |
| Dusp5 | 8.91E-07 | 0.175134 | 0.238 | 0.126 | 0.027654 | 1 |
| Virma | 8.95E-07 | 0.163736 | 0.175 | 0.083 | 0.027796 | 1 |
| Trim28 | 9.02E-07 | 0.133526 | 0.209 | 0.105 | 0.028002 | 1 |
| Gse1 | 9.03E-07 | 0.163663 | 0.16 | 0.074 | 0.028046 | 1 |
| Cdv3 | 9.04E-07 | 0.155309 | 0.421 | 0.255 | 0.028081 | 1 |
| Dpp8 | 9.19E-07 | 0.147882 | 0.221 | 0.114 | 0.028526 | 1 |
| Cltc | 9.26E-07 | 0.172568 | 0.398 | 0.244 | 0.028753 | 1 |
| Pds5b | 9.34E-07 | 0.151776 | 0.178 | 0.085 | 0.029006 | 1 |
| Lrrfip1 | 9.35E-07 | 0.146245 | 0.33 | 0.189 | 0.029031 | 1 |
| Cnr2 | 9.40E-07 | 0.108541 | 0.109 | 0.041 | 0.02919 | 1 |
| Nfya | 9.43E-07 | 0.185074 | 0.143 | 0.064 | 0.029283 | 1 |
| Xpo6 | 9.53E-07 | 0.133204 | 0.232 | 0.119 | 0.02959 | 1 |
| Aak1 | 9.69E-07 | 0.155832 | 0.152 | 0.069 | 0.030084 | 1 |
| Togaram1 | 9.83E-07 | 0.102757 | 0.138 | 0.058 | 0.030512 | 1 |
| Qk | 9.91E-07 | 0.152075 | 0.395 | 0.243 | 0.030773 | 1 |
| Rfx1 | 9.91E-07 | 0.096279 | 0.115 | 0.044 | 0.030785 | 1 |
| Btaf1 | 1.01E-06 | 0.140165 | 0.14 | 0.061 | 0.031516 | 1 |
| Diaph1 | 1.02E-06 | 0.163539 | 0.378 | 0.226 | 0.031561 | 1 |
| Lamc1 | 1.03E-06 | 0.115937 | 0.129 | 0.053 | 0.031899 | 1 |
| Nsf | 1.03E-06 | 0.231501 | 0.35 | 0.214 | 0.032033 | 1 |
| Ctss | 1.05E-06 | -0.2625 | 0.728 | 0.743 | 0.032578 | 1 |
| S1pr4 | 1.08E-06 | 0.152683 | 0.295 | 0.164 | 0.033616 | 1 |
| Chtop | 1.08E-06 | 0.171065 | 0.258 | 0.142 | 0.033638 | 1 |
| Myadm | 1.09E-06 | 0.15421 | 0.166 | 0.079 | 0.033764 | 1 |
| Tmx3 | 1.10E-06 | 0.136151 | 0.181 | 0.087 | 0.034023 | 1 |
| Serinc3 | 1.10E-06 | 0.128548 | 0.963 | 0.829 | 0.03409 | 1 |
| Luc7l2 | 1.10E-06 | 0.161034 | 0.765 | 0.574 | 0.03422 | 1 |
| Smad4 | 1.10E-06 | 0.173476 | 0.235 | 0.126 | 0.034233 | 1 |
| Kbtbd2 | 1.11E-06 | 0.166134 | 0.169 | 0.08 | 0.03438 | 1 |
| Pim3 | 1.12E-06 | 0.112217 | 0.215 | 0.108 | 0.034917 | 1 |
| Pml | 1.14E-06 | 0.18162 | 0.223 | 0.119 | 0.0353 | 1 |
| Tulp4 | 1.15E-06 | 0.127309 | 0.198 | 0.098 | 0.035719 | 1 |
| Sp3 | 1.15E-06 | 0.153492 | 0.266 | 0.148 | 0.035719 | 1 |
| Ifi206 | 1.15E-06 | 0.116939 | 0.132 | 0.055 | 0.035797 | 1 |
| Zfp407 | 1.16E-06 | 0.148572 | 0.129 | 0.055 | 0.036055 | 1 |
| Exosc10 | 1.17E-06 | 0.123743 | 0.149 | 0.067 | 0.036306 | 1 |
| Gimap8 | 1.19E-06 | 0.176405 | 0.292 | 0.165 | 0.037037 | 1 |
| Slc4a1ap | 1.19E-06 | 0.151961 | 0.175 | 0.084 | 0.037063 | 1 |
| Zfand3 | 1.21E-06 | 0.100269 | 0.169 | 0.079 | 0.037678 | 1 |
| Sart3 | 1.22E-06 | 0.094317 | 0.109 | 0.042 | 0.037849 | 1 |
| Taok3 | 1.22E-06 | 0.156979 | 0.309 | 0.178 | 0.038032 | 1 |
| Rasal3 | 1.23E-06 | 0.135301 | 0.201 | 0.1 | 0.038043 | 1 |
| Rreb1 | 1.27E-06 | 0.128473 | 0.135 | 0.058 | 0.039403 | 1 |
| Rab3gap1 | 1.29E-06 | 0.149127 | 0.241 | 0.129 | 0.039933 | 1 |
| Klhl21 | 1.29E-06 | 0.096411 | 0.106 | 0.04 | 0.040012 | 1 |
| Ttpal | 1.30E-06 | 0.138146 | 0.332 | 0.185 | 0.040362 | 1 |
| Cacna1i | 1.30E-06 | 0.15415 | 0.123 | 0.051 | 0.040404 | 1 |
| Nufip2 | 1.31E-06 | 0.177245 | 0.436 | 0.28 | 0.040573 | 1 |
| Tacc1 | 1.33E-06 | 0.145716 | 0.413 | 0.251 | 0.041416 | 1 |
| Ythdf3 | 1.38E-06 | 0.15097 | 0.201 | 0.101 | 0.042996 | 1 |
| Atp2b1 | 1.39E-06 | 0.176089 | 0.633 | 0.443 | 0.043067 | 1 |
| Calm1 | 1.39E-06 | 0.157159 | 0.96 | 0.807 | 0.043175 | 1 |
| Siglecg | 1.47E-06 | 0.209969 | 0.547 | 0.361 | 0.045533 | 1 |
| Prkd2 | 1.47E-06 | 0.170227 | 0.238 | 0.129 | 0.045578 | 1 |
| Ints6l | 1.47E-06 | 0.091945 | 0.123 | 0.051 | 0.045602 | 1 |
| Map4k4 | 1.47E-06 | 0.111359 | 0.341 | 0.196 | 0.045702 | 1 |
| Smg9 | 1.48E-06 | 0.131752 | 0.132 | 0.056 | 0.046114 | 1 |
| Abl2 | 1.49E-06 | 0.145451 | 0.117 | 0.048 | 0.046191 | 1 |
| Hmgb2 | 1.55E-06 | 0.150359 | 0.665 | 0.461 | 0.048287 | 1 |
| Tcerg1 | 1.60E-06 | 0.154313 | 0.186 | 0.093 | 0.049827 | 1 |
| Mllt6 | 1.61E-06 | 0.15756 | 0.206 | 0.105 | 0.049878 | 1 |
| Pip5k1a | 1.62E-06 | 0.114558 | 0.169 | 0.081 | 0.050249 | 1 |
| Pink1 | 1.66E-06 | 0.107382 | 0.138 | 0.06 | 0.051645 | 1 |
| Fam193a | 1.67E-06 | 0.166913 | 0.241 | 0.13 | 0.05173 | 1 |
| Calu | 1.67E-06 | 0.115586 | 0.135 | 0.058 | 0.051798 | 1 |
| Ist1 | 1.67E-06 | 0.132079 | 0.172 | 0.082 | 0.051955 | 1 |
| Pou2f2 | 1.69E-06 | 0.22372 | 0.639 | 0.454 | 0.052401 | 1 |
| Ubr1 | 1.69E-06 | 0.13344 | 0.143 | 0.064 | 0.052409 | 1 |
| Dnajc5 | 1.69E-06 | 0.118262 | 0.347 | 0.202 | 0.052566 | 1 |
| Usp6nl | 1.69E-06 | 0.129622 | 0.16 | 0.074 | 0.052634 | 1 |
| Erlin1 | 1.72E-06 | 0.128364 | 0.155 | 0.071 | 0.053318 | 1 |
| Zmym5 | 1.75E-06 | 0.141315 | 0.301 | 0.17 | 0.054429 | 1 |
| Xpo7 | 1.77E-06 | 0.143768 | 0.129 | 0.056 | 0.054833 | 1 |
| Otud4 | 1.79E-06 | 0.109026 | 0.189 | 0.092 | 0.055506 | 1 |
| Ly6e | 1.83E-06 | 0.128865 | 0.994 | 0.929 | 0.056888 | 1 |
| Mapk8 | 1.85E-06 | 0.12022 | 0.158 | 0.073 | 0.057502 | 1 |
| Icosl | 1.86E-06 | 0.122221 | 0.198 | 0.098 | 0.057765 | 1 |
| Zufsp | 1.87E-06 | 0.160904 | 0.275 | 0.154 | 0.057945 | 1 |
| Jade2 | 1.87E-06 | 0.10818 | 0.106 | 0.041 | 0.058122 | 1 |
| Ifngr1 | 1.87E-06 | 0.141968 | 0.287 | 0.161 | 0.058164 | 1 |
| Il17ra | 1.90E-06 | 0.178695 | 0.232 | 0.126 | 0.059039 | 1 |
| Ubr4 | 1.92E-06 | 0.105101 | 0.223 | 0.116 | 0.059662 | 1 |
| Samd8 | 1.93E-06 | 0.124272 | 0.117 | 0.048 | 0.060054 | 1 |
| Satb1 | 1.96E-06 | 0.188709 | 0.808 | 0.619 | 0.060723 | 1 |
| Rc3h2 | 1.99E-06 | 0.151393 | 0.246 | 0.136 | 0.061754 | 1 |
| Kpna1 | 2.01E-06 | 0.157183 | 0.258 | 0.143 | 0.062326 | 1 |
| Tbc1d20 | 2.02E-06 | 0.120936 | 0.229 | 0.12 | 0.062875 | 1 |
| Xpo1 | 2.05E-06 | 0.166418 | 0.183 | 0.092 | 0.063579 | 1 |
| Sf3b2 | 2.08E-06 | 0.15692 | 0.576 | 0.384 | 0.064685 | 1 |
| Gramd3 | 2.10E-06 | 0.180738 | 0.393 | 0.247 | 0.06535 | 1 |
| Phf2 | 2.11E-06 | 0.152739 | 0.135 | 0.059 | 0.065632 | 1 |
| Phrf1 | 2.14E-06 | 0.152769 | 0.166 | 0.08 | 0.066597 | 1 |
| Mast4 | 2.16E-06 | 0.168772 | 0.241 | 0.131 | 0.067111 | 1 |
| Gmfb | 2.18E-06 | 0.097228 | 0.235 | 0.124 | 0.067579 | 1 |
| mt-Nd4 | 2.19E-06 | 0.11196 | 1 | 0.992 | 0.068044 | 1 |
| Ctsl | 2.21E-06 | -0.37115 | 0.063 | 0.154 | 0.068649 | 1 |
| Zfp445 | 2.23E-06 | 0.153977 | 0.186 | 0.093 | 0.069358 | 1 |
| Slfn8 | 2.24E-06 | 0.149043 | 0.106 | 0.042 | 0.069427 | 1 |
| Samhd1 | 2.24E-06 | 0.136615 | 0.696 | 0.478 | 0.069525 | 1 |
| Lrrc58 | 2.25E-06 | 0.146297 | 0.132 | 0.058 | 0.069771 | 1 |
| Rnf44 | 2.33E-06 | 0.122693 | 0.215 | 0.11 | 0.072345 | 1 |
| Copg1 | 2.34E-06 | 0.108716 | 0.155 | 0.072 | 0.072767 | 1 |
| Cst3 | 2.35E-06 | -0.33502 | 0.496 | 0.538 | 0.072881 | 1 |
| Hook1 | 2.36E-06 | 0.11083 | 0.112 | 0.045 | 0.073386 | 1 |
| Lsg1 | 2.37E-06 | 0.124232 | 0.181 | 0.089 | 0.073478 | 1 |
| Ubr3 | 2.37E-06 | 0.153633 | 0.172 | 0.085 | 0.073517 | 1 |
| Tbce | 2.38E-06 | 0.128401 | 0.115 | 0.047 | 0.073844 | 1 |
| Nono | 2.38E-06 | 0.112119 | 0.407 | 0.244 | 0.073861 | 1 |
| Khnyn | 2.38E-06 | 0.108218 | 0.172 | 0.082 | 0.073882 | 1 |
| Nxf1 | 2.47E-06 | 0.149529 | 0.324 | 0.189 | 0.076718 | 1 |
| Dbr1 | 2.62E-06 | 0.110031 | 0.115 | 0.047 | 0.081236 | 1 |
| Taf15 | 2.64E-06 | 0.13153 | 0.327 | 0.188 | 0.082016 | 1 |
| Ythdc2 | 2.66E-06 | 0.143925 | 0.152 | 0.071 | 0.082543 | 1 |
| Crtc2 | 2.69E-06 | 0.118352 | 0.155 | 0.072 | 0.08345 | 1 |
| Iqsec1 | 2.73E-06 | 0.138022 | 0.327 | 0.191 | 0.084736 | 1 |
| Cdc42se1 | 2.75E-06 | 0.157685 | 0.344 | 0.205 | 0.085463 | 1 |
| Plcl2 | 2.80E-06 | 0.109914 | 0.16 | 0.076 | 0.086898 | 1 |
| Hvcn1 | 2.82E-06 | 0.197909 | 0.653 | 0.463 | 0.087649 | 1 |
| Eif3c | 2.87E-06 | 0.185291 | 0.504 | 0.345 | 0.089187 | 1 |
| Zc3h18 | 2.88E-06 | 0.130024 | 0.195 | 0.098 | 0.089376 | 1 |
| Upf2 | 2.92E-06 | 0.159204 | 0.241 | 0.131 | 0.090714 | 1 |
| Grap | 2.93E-06 | 0.152064 | 0.43 | 0.273 | 0.090931 | 1 |
| Washc2 | 2.94E-06 | 0.211616 | 0.232 | 0.129 | 0.091266 | 1 |
| Zc3h10 | 2.94E-06 | 0.104465 | 0.138 | 0.061 | 0.091409 | 1 |
| Parp8 | 2.95E-06 | 0.105174 | 0.129 | 0.055 | 0.091603 | 1 |
| Man1a | 2.95E-06 | 0.16721 | 0.622 | 0.428 | 0.091615 | 1 |
| Atp5l | 2.99E-06 | -0.25582 | 0.742 | 0.688 | 0.092876 | 1 |
| Nin | 3.03E-06 | 0.163864 | 0.198 | 0.103 | 0.094141 | 1 |
| Dhx15 | 3.04E-06 | 0.141069 | 0.361 | 0.215 | 0.094516 | 1 |
| Rell1 | 3.04E-06 | 0.102387 | 0.195 | 0.098 | 0.094524 | 1 |
| Hdlbp | 3.07E-06 | 0.129308 | 0.258 | 0.144 | 0.095247 | 1 |
| Acsl3 | 3.12E-06 | 0.161201 | 0.117 | 0.049 | 0.09702 | 1 |
| Arhgef6 | 3.14E-06 | 0.122245 | 0.215 | 0.114 | 0.097598 | 1 |
| Rbm26 | 3.14E-06 | 0.163507 | 0.324 | 0.192 | 0.097615 | 1 |
| Man2b1 | 3.18E-06 | 0.148597 | 0.35 | 0.214 | 0.09882 | 1 |
| Rnf10 | 3.20E-06 | 0.172173 | 0.255 | 0.144 | 0.099462 | 1 |
| Smc1a | 3.25E-06 | 0.138038 | 0.332 | 0.196 | 0.100957 | 1 |
| Atp2a3 | 3.27E-06 | 0.173848 | 0.301 | 0.175 | 0.101521 | 1 |
| Snx2 | 3.29E-06 | 0.163336 | 0.607 | 0.408 | 0.102186 | 1 |
| Dhx40 | 3.32E-06 | 0.141712 | 0.272 | 0.155 | 0.103241 | 1 |
| Cnot6 | 3.36E-06 | 0.133084 | 0.255 | 0.142 | 0.104261 | 1 |
| Csnk1g2 | 3.37E-06 | 0.12022 | 0.266 | 0.148 | 0.104659 | 1 |
| Snx5 | 3.38E-06 | 0.134348 | 0.696 | 0.483 | 0.10484 | 1 |
| Lcorl | 3.42E-06 | 0.146481 | 0.169 | 0.083 | 0.106237 | 1 |
| Epc2 | 3.46E-06 | 0.114234 | 0.264 | 0.143 | 0.107489 | 1 |
| Jmjd1c | 3.65E-06 | 0.152466 | 0.622 | 0.413 | 0.113321 | 1 |
| Mad2l1bp | 3.67E-06 | 0.112079 | 0.115 | 0.047 | 0.114055 | 1 |
| Rab3gap2 | 3.76E-06 | 0.116809 | 0.106 | 0.042 | 0.116899 | 1 |
| Nfatc2ip | 3.80E-06 | 0.096199 | 0.12 | 0.051 | 0.117943 | 1 |
| Pcsk7 | 3.82E-06 | 0.091208 | 0.158 | 0.074 | 0.118669 | 1 |
| Emsy | 3.84E-06 | 0.129457 | 0.261 | 0.144 | 0.119129 | 1 |
| Colgalt1 | 3.89E-06 | 0.151658 | 0.229 | 0.125 | 0.120911 | 1 |
| Rasgrp3 | 3.91E-06 | 0.129188 | 0.281 | 0.159 | 0.121456 | 1 |
| Kctd12 | 4.03E-06 | 0.146219 | 0.504 | 0.332 | 0.12524 | 1 |
| Srf | 4.07E-06 | 0.133728 | 0.112 | 0.047 | 0.126358 | 1 |
| Zzef1 | 4.09E-06 | 0.111325 | 0.209 | 0.108 | 0.127022 | 1 |
| Scai | 4.15E-06 | 0.141277 | 0.172 | 0.085 | 0.128773 | 1 |
| Slc2a1 | 4.16E-06 | 0.134305 | 0.178 | 0.088 | 0.129046 | 1 |
| Nrf1 | 4.18E-06 | 0.11228 | 0.206 | 0.107 | 0.129751 | 1 |
| Ip6k1 | 4.18E-06 | 0.068106 | 0.203 | 0.101 | 0.129799 | 1 |
| Arl5b | 4.29E-06 | 0.209568 | 0.198 | 0.103 | 0.133287 | 1 |
| Nck1 | 4.32E-06 | 0.153355 | 0.178 | 0.09 | 0.134188 | 1 |
| Naa15 | 4.37E-06 | 0.160473 | 0.284 | 0.169 | 0.135716 | 1 |
| Parp4 | 4.40E-06 | 0.152292 | 0.138 | 0.064 | 0.136537 | 1 |
| Mapre2 | 4.42E-06 | 0.147032 | 0.169 | 0.085 | 0.137181 | 1 |
| Zbtb43 | 4.46E-06 | 0.128969 | 0.103 | 0.041 | 0.138594 | 1 |
| Neil1 | 4.55E-06 | 0.112846 | 0.146 | 0.067 | 0.141318 | 1 |
| Bag6 | 4.63E-06 | 0.118405 | 0.163 | 0.079 | 0.143877 | 1 |
| Tsc1 | 4.69E-06 | 0.141154 | 0.138 | 0.063 | 0.145756 | 1 |
| H2-DMb1 | 4.71E-06 | 0.127062 | 0.407 | 0.246 | 0.146266 | 1 |
| Cyba | 4.72E-06 | -0.24067 | 0.799 | 0.772 | 0.146572 | 1 |
| Cep120 | 4.75E-06 | 0.145095 | 0.158 | 0.075 | 0.147442 | 1 |
| Psap | 4.81E-06 | -0.36778 | 0.599 | 0.593 | 0.149517 | 1 |
| Pim1 | 4.87E-06 | 0.14827 | 0.605 | 0.422 | 0.151306 | 1 |
| Kctd3 | 4.88E-06 | 0.120656 | 0.109 | 0.045 | 0.151428 | 1 |
| Secisbp2 | 4.89E-06 | 0.155345 | 0.14 | 0.066 | 0.151922 | 1 |
| Rbm10 | 4.91E-06 | 0.112966 | 0.12 | 0.052 | 0.152508 | 1 |
| Retreg3 | 4.93E-06 | 0.111703 | 0.209 | 0.108 | 0.153209 | 1 |
| Otud5 | 5.01E-06 | 0.096967 | 0.189 | 0.096 | 0.155687 | 1 |
| Cdkn2aip | 5.13E-06 | 0.092141 | 0.189 | 0.094 | 0.159406 | 1 |
| Adgre5 | 5.14E-06 | 0.173453 | 0.444 | 0.293 | 0.159482 | 1 |
| Rnf216 | 5.16E-06 | 0.122851 | 0.284 | 0.161 | 0.160109 | 1 |
| Bach1 | 5.19E-06 | 0.128663 | 0.155 | 0.074 | 0.1613 | 1 |
| Nae1 | 5.21E-06 | 0.090743 | 0.206 | 0.107 | 0.161736 | 1 |
| Plekho1 | 5.23E-06 | 0.152197 | 0.332 | 0.198 | 0.162437 | 1 |
| Camk1d | 5.25E-06 | 0.123283 | 0.149 | 0.071 | 0.163012 | 1 |
| Atm | 5.28E-06 | 0.135831 | 0.135 | 0.062 | 0.163994 | 1 |
| Kmt2b | 5.39E-06 | 0.132344 | 0.206 | 0.108 | 0.167403 | 1 |
| Zc3h7a | 5.48E-06 | 0.149752 | 0.298 | 0.177 | 0.170183 | 1 |
| Fam193b | 5.49E-06 | 0.136654 | 0.117 | 0.051 | 0.170339 | 1 |
| Ncor2 | 5.50E-06 | 0.098968 | 0.123 | 0.053 | 0.170846 | 1 |
| Bcor | 5.51E-06 | 0.11509 | 0.112 | 0.047 | 0.171014 | 1 |
| Edem3 | 5.52E-06 | 0.125323 | 0.106 | 0.044 | 0.171555 | 1 |
| Slc1a5 | 5.55E-06 | 0.145794 | 0.269 | 0.155 | 0.172206 | 1 |
| Rbm5 | 5.72E-06 | 0.178096 | 0.607 | 0.415 | 0.17754 | 1 |
| Pafah1b1 | 5.72E-06 | 0.126401 | 0.562 | 0.372 | 0.177729 | 1 |
| Trim7 | 5.78E-06 | 0.151052 | 0.292 | 0.169 | 0.179338 | 1 |
| Orc2 | 5.78E-06 | 0.129485 | 0.126 | 0.056 | 0.179434 | 1 |
| Lpin2 | 5.78E-06 | 0.108849 | 0.123 | 0.054 | 0.179563 | 1 |
| Kansl1 | 5.93E-06 | 0.113382 | 0.384 | 0.23 | 0.184072 | 1 |
| Tank | 5.94E-06 | 0.106745 | 0.252 | 0.141 | 0.184313 | 1 |
| Larp7 | 5.96E-06 | 0.156541 | 0.178 | 0.091 | 0.18512 | 1 |
| Was | 5.97E-06 | 0.137076 | 0.287 | 0.164 | 0.185438 | 1 |
| Eif5 | 5.98E-06 | 0.178739 | 0.768 | 0.58 | 0.185812 | 1 |
| Inpp5f | 6.03E-06 | 0.106371 | 0.158 | 0.076 | 0.187348 | 1 |
| Mast3 | 6.13E-06 | 0.125617 | 0.138 | 0.062 | 0.190333 | 1 |
| Pou2af1 | 6.19E-06 | 0.217875 | 0.653 | 0.49 | 0.192113 | 1 |
| B3gnt7 | 6.19E-06 | 0.138386 | 0.115 | 0.049 | 0.192209 | 1 |
| Map2k7 | 6.20E-06 | 0.130706 | 0.143 | 0.067 | 0.19249 | 1 |
| Polr2m | 6.27E-06 | 0.133728 | 0.275 | 0.159 | 0.194748 | 1 |
| Frat2 | 6.37E-06 | 0.107963 | 0.166 | 0.08 | 0.19784 | 1 |
| Blk | 6.41E-06 | 0.161025 | 0.395 | 0.245 | 0.199017 | 1 |
| Ubp1 | 6.46E-06 | 0.097664 | 0.192 | 0.098 | 0.200747 | 1 |
| Stim1 | 6.47E-06 | 0.104746 | 0.252 | 0.139 | 0.200929 | 1 |
| Rlf | 6.52E-06 | 0.084102 | 0.275 | 0.151 | 0.202339 | 1 |
| Gsk3b | 6.58E-06 | 0.180086 | 0.309 | 0.184 | 0.204201 | 1 |
| Sppl3 | 6.62E-06 | 0.075723 | 0.138 | 0.062 | 0.205512 | 1 |
| Aplp2 | 6.84E-06 | 0.131853 | 0.246 | 0.137 | 0.212484 | 1 |
| Brap | 6.88E-06 | 0.106972 | 0.172 | 0.085 | 0.21369 | 1 |
| Klf7 | 6.97E-06 | 0.127308 | 0.203 | 0.109 | 0.216568 | 1 |
| Srgn | 7.07E-06 | -0.23237 | 0.951 | 0.916 | 0.219531 | 1 |
| Zfp68 | 7.09E-06 | 0.133716 | 0.163 | 0.08 | 0.220191 | 1 |
| Ppip5k2 | 7.27E-06 | 0.115393 | 0.129 | 0.058 | 0.22579 | 1 |
| Cd40 | 7.29E-06 | 0.108023 | 0.198 | 0.103 | 0.226428 | 1 |
| Glud1 | 7.44E-06 | 0.128169 | 0.295 | 0.175 | 0.231077 | 1 |
| Cnot3 | 7.55E-06 | 0.108593 | 0.261 | 0.147 | 0.234453 | 1 |
| Eps15l1 | 7.60E-06 | 0.093301 | 0.135 | 0.061 | 0.235996 | 1 |
| Tmem108 | 7.66E-06 | 0.122236 | 0.232 | 0.126 | 0.237923 | 1 |
| Tmsb10 | 7.83E-06 | -0.19019 | 0.977 | 0.965 | 0.243058 | 1 |
| Tet3 | 7.92E-06 | 0.135659 | 0.212 | 0.114 | 0.24582 | 1 |
| Cobll1 | 7.93E-06 | 0.157487 | 0.138 | 0.065 | 0.246241 | 1 |
| Rap1gds1 | 7.93E-06 | 0.11629 | 0.189 | 0.098 | 0.246254 | 1 |
| Parp14 | 7.97E-06 | 0.112503 | 0.238 | 0.13 | 0.247608 | 1 |
| Ctdsp1 | 8.32E-06 | 0.123988 | 0.172 | 0.087 | 0.258477 | 1 |
| Phf20 | 8.39E-06 | 0.152997 | 0.163 | 0.083 | 0.260675 | 1 |
| Ypel3 | 8.48E-06 | 0.138027 | 0.436 | 0.275 | 0.2634 | 1 |
| Dhx9 | 8.50E-06 | 0.109967 | 0.433 | 0.272 | 0.26409 | 1 |
| Tlk1 | 8.60E-06 | 0.135402 | 0.275 | 0.162 | 0.266908 | 1 |
| Map2k4 | 8.66E-06 | 0.11732 | 0.132 | 0.06 | 0.268801 | 1 |
| Nrd1 | 8.69E-06 | 0.128839 | 0.347 | 0.211 | 0.269963 | 1 |
| Uqcrh | 8.81E-06 | -0.23152 | 0.845 | 0.806 | 0.273661 | 1 |
| Myo1c | 8.83E-06 | 0.143344 | 0.289 | 0.171 | 0.274173 | 1 |
| Eif4ebp2 | 8.87E-06 | 0.106891 | 0.281 | 0.163 | 0.275286 | 1 |
| Xrn2 | 8.87E-06 | 0.187301 | 0.467 | 0.316 | 0.275367 | 1 |
| Epc1 | 8.89E-06 | 0.148611 | 0.266 | 0.154 | 0.276211 | 1 |
| Rbm27 | 9.09E-06 | 0.127962 | 0.269 | 0.153 | 0.282328 | 1 |
| Sidt2 | 9.11E-06 | 0.108336 | 0.249 | 0.139 | 0.282842 | 1 |
| Paxbp1 | 9.18E-06 | 0.113844 | 0.218 | 0.118 | 0.284987 | 1 |
| Hmgxb4 | 9.18E-06 | 0.101164 | 0.112 | 0.047 | 0.285055 | 1 |
| Efcab14 | 9.25E-06 | 0.113445 | 0.152 | 0.073 | 0.287089 | 1 |
| Strn4 | 9.37E-06 | 0.137119 | 0.138 | 0.065 | 0.290929 | 1 |
| Chka | 9.41E-06 | 0.152997 | 0.272 | 0.159 | 0.292305 | 1 |
| Rb1 | 9.48E-06 | 0.113162 | 0.192 | 0.101 | 0.294455 | 1 |
| Map3k2 | 9.53E-06 | 0.142674 | 0.192 | 0.101 | 0.295781 | 1 |
| Ly9 | 9.73E-06 | 0.134289 | 0.209 | 0.114 | 0.302074 | 1 |
| Ets2 | 9.84E-06 | 0.161439 | 0.229 | 0.129 | 0.305562 | 1 |
| Elf4 | 9.89E-06 | 0.13197 | 0.146 | 0.071 | 0.307102 | 1 |
| Pmm2 | 1.00E-05 | 0.125123 | 0.126 | 0.058 | 0.310598 | 1 |
| Avl9 | 1.01E-05 | 0.084086 | 0.433 | 0.261 | 0.312952 | 1 |
| Prkx | 1.01E-05 | 0.110132 | 0.155 | 0.076 | 0.31426 | 1 |
| Actn4 | 1.01E-05 | 0.111726 | 0.255 | 0.144 | 0.31481 | 1 |
| Fmr1 | 1.02E-05 | 0.13667 | 0.244 | 0.135 | 0.315341 | 1 |
| Acap1 | 1.02E-05 | 0.112992 | 0.238 | 0.131 | 0.316854 | 1 |
| Lgmn | 1.02E-05 | -0.3656 | 0.166 | 0.256 | 0.317865 | 1 |
| Cdk9 | 1.02E-05 | 0.112022 | 0.289 | 0.167 | 0.318 | 1 |
| Cep83 | 1.03E-05 | 0.150982 | 0.126 | 0.058 | 0.319715 | 1 |
| Rab22a | 1.03E-05 | 0.113798 | 0.181 | 0.094 | 0.320016 | 1 |
| Pou2f1 | 1.04E-05 | 0.153842 | 0.241 | 0.137 | 0.322498 | 1 |
| Ehd1 | 1.06E-05 | 0.127508 | 0.33 | 0.2 | 0.328964 | 1 |
| Zeb1 | 1.09E-05 | 0.127663 | 0.238 | 0.133 | 0.339081 | 1 |
| Tnks | 1.09E-05 | 0.115552 | 0.123 | 0.055 | 0.339182 | 1 |
| Ipmk | 1.10E-05 | 0.135529 | 0.132 | 0.062 | 0.341755 | 1 |
| Cd68 | 1.11E-05 | -0.25137 | 0.032 | 0.105 | 0.344396 | 1 |
| Ddx54 | 1.11E-05 | 0.131577 | 0.338 | 0.209 | 0.345006 | 1 |
| Lemd3 | 1.12E-05 | 0.100797 | 0.166 | 0.082 | 0.346622 | 1 |
| Kctd10 | 1.13E-05 | 0.119555 | 0.138 | 0.064 | 0.350957 | 1 |
| Sav1 | 1.14E-05 | 0.115823 | 0.149 | 0.073 | 0.353394 | 1 |
| Usp3 | 1.14E-05 | 0.153945 | 0.221 | 0.123 | 0.353757 | 1 |
| Med14 | 1.15E-05 | 0.118949 | 0.109 | 0.047 | 0.356827 | 1 |
| Maml3 | 1.16E-05 | 0.102257 | 0.103 | 0.042 | 0.360222 | 1 |
| Apc | 1.16E-05 | 0.142299 | 0.249 | 0.142 | 0.361039 | 1 |
| Csnk1g1 | 1.16E-05 | 0.11044 | 0.172 | 0.089 | 0.361267 | 1 |
| Gadd45a | 1.18E-05 | 0.139334 | 0.272 | 0.158 | 0.365613 | 1 |
| Tug1 | 1.20E-05 | 0.134322 | 0.238 | 0.132 | 0.37215 | 1 |
| Rnf167 | 1.22E-05 | 0.119584 | 0.264 | 0.151 | 0.379786 | 1 |
| Nr3c1 | 1.23E-05 | 0.162957 | 0.344 | 0.216 | 0.381857 | 1 |
| R3hdm1 | 1.23E-05 | 0.167051 | 0.404 | 0.261 | 0.38193 | 1 |
| Slain2 | 1.23E-05 | 0.120571 | 0.175 | 0.09 | 0.382675 | 1 |
| Pias1 | 1.24E-05 | 0.091992 | 0.258 | 0.148 | 0.383505 | 1 |
| Galnt11 | 1.24E-05 | 0.124148 | 0.272 | 0.156 | 0.38382 | 1 |
| Dbnl | 1.27E-05 | 0.112332 | 0.327 | 0.197 | 0.393194 | 1 |
| Calr | 1.27E-05 | 0.168669 | 0.765 | 0.547 | 0.393741 | 1 |
| Trim59 | 1.27E-05 | 0.105965 | 0.155 | 0.076 | 0.395489 | 1 |
| Ppp6r3 | 1.27E-05 | 0.112197 | 0.315 | 0.188 | 0.395574 | 1 |
| Man1a2 | 1.30E-05 | 0.153921 | 0.229 | 0.13 | 0.402524 | 1 |
| Ik | 1.32E-05 | 0.115949 | 0.304 | 0.182 | 0.411017 | 1 |
| Zfp592 | 1.34E-05 | 0.15634 | 0.175 | 0.091 | 0.416282 | 1 |
| Lrp10 | 1.37E-05 | 0.081845 | 0.361 | 0.218 | 0.424279 | 1 |
| Stim2 | 1.38E-05 | 0.100258 | 0.126 | 0.058 | 0.427661 | 1 |
| Nfkb2 | 1.39E-05 | 0.143273 | 0.235 | 0.133 | 0.433108 | 1 |
| B9d2 | 1.41E-05 | 0.10796 | 0.186 | 0.098 | 0.437816 | 1 |
| Scaf1 | 1.42E-05 | 0.08227 | 0.115 | 0.05 | 0.440849 | 1 |
| Rnf139 | 1.42E-05 | 0.090901 | 0.155 | 0.076 | 0.441742 | 1 |
| Dlg1 | 1.44E-05 | 0.163278 | 0.169 | 0.087 | 0.446398 | 1 |
| Cpsf6 | 1.44E-05 | 0.123918 | 0.344 | 0.212 | 0.44803 | 1 |
| Mt1 | 1.45E-05 | -0.33441 | 0.069 | 0.151 | 0.449122 | 1 |
| Ankib1 | 1.45E-05 | 0.150495 | 0.163 | 0.083 | 0.451487 | 1 |
| Cd79b | 1.46E-05 | 0.240669 | 0.92 | 0.752 | 0.453763 | 1 |
| Midn | 1.46E-05 | 0.094574 | 0.235 | 0.13 | 0.454479 | 1 |
| U2surp | 1.47E-05 | 0.143009 | 0.364 | 0.228 | 0.455565 | 1 |
| Esyt1 | 1.47E-05 | 0.095005 | 0.295 | 0.172 | 0.456226 | 1 |
| Tbl1x | 1.48E-05 | 0.105231 | 0.189 | 0.1 | 0.460647 | 1 |
| Mapkap1 | 1.50E-05 | 0.116213 | 0.166 | 0.085 | 0.46439 | 1 |
| Samd1 | 1.51E-05 | 0.114238 | 0.106 | 0.046 | 0.468121 | 1 |
| Dopey1 | 1.52E-05 | 0.142965 | 0.129 | 0.06 | 0.471865 | 1 |
| Notch2 | 1.53E-05 | 0.082229 | 0.275 | 0.157 | 0.474215 | 1 |
| Zswim8 | 1.53E-05 | 0.112093 | 0.143 | 0.069 | 0.476092 | 1 |
| Pabpc4 | 1.54E-05 | 0.142919 | 0.143 | 0.071 | 0.478229 | 1 |
| Sumo2 | 1.56E-05 | -0.28463 | 0.562 | 0.571 | 0.483767 | 1 |
| Fkbp15 | 1.59E-05 | 0.097497 | 0.189 | 0.1 | 0.493213 | 1 |
| Rbm6 | 1.59E-05 | 0.126619 | 0.292 | 0.172 | 0.493531 | 1 |
| Ensa | 1.59E-05 | 0.107438 | 0.287 | 0.169 | 0.4937 | 1 |
| Rmnd5a | 1.59E-05 | 0.126793 | 0.178 | 0.094 | 0.494472 | 1 |
| Ankrd13c | 1.62E-05 | 0.107251 | 0.212 | 0.115 | 0.501806 | 1 |
| Xab2 | 1.63E-05 | 0.083155 | 0.109 | 0.047 | 0.504753 | 1 |
| Ccnd3 | 1.63E-05 | 0.100661 | 0.41 | 0.259 | 0.505983 | 1 |
| Vps13b | 1.64E-05 | 0.112527 | 0.178 | 0.093 | 0.509684 | 1 |
| Otulin | 1.65E-05 | 0.091792 | 0.321 | 0.189 | 0.510974 | 1 |
| Tcea1 | 1.71E-05 | 0.146949 | 0.364 | 0.231 | 0.532199 | 1 |
| Vgll4 | 1.74E-05 | 0.119256 | 0.295 | 0.176 | 0.541115 | 1 |
| Gabpb1 | 1.81E-05 | 0.10521 | 0.106 | 0.046 | 0.561233 | 1 |
| Tuba1a | 1.81E-05 | 0.124215 | 0.364 | 0.227 | 0.562651 | 1 |
| 9030624J02Rik | 1.81E-05 | 0.09555 | 0.138 | 0.065 | 0.56323 | 1 |
| Prkar1a | 1.82E-05 | 0.117859 | 0.513 | 0.337 | 0.565908 | 1 |
| Clasp1 | 1.83E-05 | 0.142129 | 0.235 | 0.135 | 0.569569 | 1 |
| Pikfyve | 1.85E-05 | 0.089788 | 0.203 | 0.11 | 0.574629 | 1 |
| Smarce1 | 1.86E-05 | 0.128782 | 0.295 | 0.18 | 0.576783 | 1 |
| Pank2 | 1.87E-05 | 0.086291 | 0.155 | 0.077 | 0.581513 | 1 |
| Zfand5 | 1.87E-05 | 0.071382 | 0.421 | 0.261 | 0.5822 | 1 |
| Senp5 | 1.88E-05 | 0.100357 | 0.109 | 0.047 | 0.582375 | 1 |
| Ccdc174 | 1.92E-05 | 0.091065 | 0.209 | 0.114 | 0.59684 | 1 |
| Rcor1 | 1.95E-05 | 0.137623 | 0.166 | 0.086 | 0.606699 | 1 |
| Tox4 | 2.00E-05 | 0.107735 | 0.269 | 0.157 | 0.621035 | 1 |
| Foxk1 | 2.03E-05 | 0.125251 | 0.109 | 0.048 | 0.629756 | 1 |
| Ifi213 | 2.04E-05 | 0.141135 | 0.112 | 0.049 | 0.632071 | 1 |
| Dhx8 | 2.07E-05 | 0.09744 | 0.166 | 0.085 | 0.643996 | 1 |
| Itgb3 | 2.16E-05 | 0.083264 | 0.103 | 0.044 | 0.669679 | 1 |
| Luc7l3 | 2.16E-05 | 0.132446 | 0.447 | 0.288 | 0.671483 | 1 |
| Dyrk2 | 2.17E-05 | 0.121548 | 0.132 | 0.062 | 0.67396 | 1 |
| Ppp1r9b | 2.18E-05 | 0.123681 | 0.123 | 0.057 | 0.675588 | 1 |
| Myo1e | 2.19E-05 | 0.158566 | 0.327 | 0.203 | 0.679736 | 1 |
| Ankfy1 | 2.22E-05 | 0.094367 | 0.189 | 0.101 | 0.689032 | 1 |
| Mpeg1 | 2.22E-05 | -0.29305 | 0.052 | 0.128 | 0.689639 | 1 |
| Ppp3ca | 2.23E-05 | 0.145865 | 0.685 | 0.499 | 0.693542 | 1 |
| H2afy | 2.25E-05 | 0.166168 | 0.524 | 0.366 | 0.697371 | 1 |
| Psen2 | 2.26E-05 | 0.096454 | 0.181 | 0.094 | 0.700701 | 1 |
| Gadd45b | 2.27E-05 | -0.4418 | 0.212 | 0.307 | 0.704354 | 1 |
| Slc30a5 | 2.27E-05 | 0.147042 | 0.198 | 0.108 | 0.705568 | 1 |
| Abcf1 | 2.28E-05 | 0.110747 | 0.272 | 0.162 | 0.70661 | 1 |
| Ssh1 | 2.28E-05 | 0.086253 | 0.132 | 0.062 | 0.709269 | 1 |
| Mbp | 2.31E-05 | 0.128386 | 0.115 | 0.052 | 0.715825 | 1 |
| Ap1m1 | 2.34E-05 | 0.110728 | 0.201 | 0.11 | 0.726144 | 1 |
| Wapl | 2.34E-05 | 0.142843 | 0.321 | 0.2 | 0.727079 | 1 |
| Filip1l | 2.34E-05 | 0.163069 | 0.533 | 0.375 | 0.728055 | 1 |
| Naa35 | 2.39E-05 | 0.113232 | 0.138 | 0.067 | 0.742193 | 1 |
| Rlim | 2.40E-05 | 0.161598 | 0.338 | 0.214 | 0.745024 | 1 |
| Spen | 2.49E-05 | 0.155766 | 0.172 | 0.092 | 0.773367 | 1 |
| Vps11 | 2.49E-05 | 0.109183 | 0.109 | 0.048 | 0.774729 | 1 |
| Ecd | 2.50E-05 | 0.103686 | 0.123 | 0.057 | 0.777664 | 1 |
| Lrrk1 | 2.53E-05 | 0.104147 | 0.117 | 0.053 | 0.785688 | 1 |
| Csnk2a2 | 2.54E-05 | 0.138771 | 0.235 | 0.137 | 0.787672 | 1 |
| Ttc14 | 2.55E-05 | 0.136334 | 0.49 | 0.325 | 0.790748 | 1 |
| Atp5e | 2.57E-05 | -0.2263 | 0.825 | 0.761 | 0.796876 | 1 |
| Itm2b | 2.57E-05 | -0.23704 | 0.728 | 0.711 | 0.797028 | 1 |
| Usp48 | 2.58E-05 | 0.120306 | 0.155 | 0.078 | 0.801212 | 1 |
| Ythdf1 | 2.59E-05 | 0.118506 | 0.16 | 0.082 | 0.804634 | 1 |
| Spty2d1 | 2.60E-05 | 0.157427 | 0.275 | 0.167 | 0.807341 | 1 |
| Ddx23 | 2.62E-05 | 0.105289 | 0.198 | 0.107 | 0.81337 | 1 |
| Nup160 | 2.65E-05 | 0.126766 | 0.106 | 0.047 | 0.823529 | 1 |
| Immt | 2.66E-05 | 0.105134 | 0.261 | 0.154 | 0.825425 | 1 |
| Lin7c | 2.67E-05 | 0.111691 | 0.198 | 0.108 | 0.829864 | 1 |
| Ahsa1 | 2.68E-05 | -0.38171 | 0.206 | 0.287 | 0.832335 | 1 |
| Vps72 | 2.70E-05 | 0.110259 | 0.117 | 0.054 | 0.838651 | 1 |
| Elovl5 | 2.72E-05 | 0.099899 | 0.152 | 0.076 | 0.844469 | 1 |
| Abca3 | 2.72E-05 | 0.098604 | 0.115 | 0.052 | 0.845365 | 1 |
| Ly6a | 2.73E-05 | -0.41534 | 0.309 | 0.387 | 0.848706 | 1 |
| Zbtb20 | 2.75E-05 | 0.101929 | 0.401 | 0.269 | 0.854818 | 1 |
| Wasf2 | 2.78E-05 | 0.126656 | 0.464 | 0.304 | 0.86224 | 1 |
| Ptpra | 2.78E-05 | 0.09627 | 0.195 | 0.105 | 0.862685 | 1 |
| Pik3ca | 2.78E-05 | 0.123599 | 0.226 | 0.13 | 0.86372 | 1 |
| Isca1 | 2.81E-05 | 0.12461 | 0.181 | 0.096 | 0.872744 | 1 |
| Gripap1 | 2.82E-05 | 0.099605 | 0.272 | 0.161 | 0.876573 | 1 |
| Mtpap | 2.83E-05 | 0.107277 | 0.115 | 0.052 | 0.878238 | 1 |
| Rbm34 | 2.85E-05 | 0.135156 | 0.16 | 0.083 | 0.884125 | 1 |
| Id2 | 2.85E-05 | -0.30727 | 0.052 | 0.127 | 0.886166 | 1 |
| Rpia | 2.86E-05 | 0.115458 | 0.166 | 0.088 | 0.888925 | 1 |
| Zpr1 | 2.89E-05 | 0.089149 | 0.14 | 0.069 | 0.896242 | 1 |
| Fyn | 2.89E-05 | 0.120413 | 0.149 | 0.074 | 0.89644 | 1 |
| Vav1 | 2.93E-05 | 0.097093 | 0.203 | 0.112 | 0.909384 | 1 |
| Hist1h4d | 2.94E-05 | 0.104437 | 0.106 | 0.047 | 0.912545 | 1 |
| Fcho2 | 3.00E-05 | 0.102878 | 0.218 | 0.123 | 0.932709 | 1 |
| Lcor | 3.02E-05 | 0.089911 | 0.163 | 0.084 | 0.93656 | 1 |
| Csk | 3.03E-05 | 0.114932 | 0.487 | 0.32 | 0.9407 | 1 |
| Glcci1 | 3.03E-05 | 0.129721 | 0.129 | 0.062 | 0.941732 | 1 |
| Ssr4 | 3.06E-05 | -0.39184 | 0.33 | 0.383 | 0.950087 | 1 |
| Slc25a36 | 3.07E-05 | 0.104364 | 0.238 | 0.135 | 0.952774 | 1 |
| Crebrf | 3.07E-05 | 0.164384 | 0.458 | 0.311 | 0.95279 | 1 |
| Ctps2 | 3.08E-05 | 0.122053 | 0.155 | 0.079 | 0.955295 | 1 |
| Cwc22 | 3.12E-05 | 0.106602 | 0.138 | 0.067 | 0.970021 | 1 |
| Rsbn1l | 3.17E-05 | 0.116292 | 0.516 | 0.349 | 0.983895 | 1 |
| Sos1 | 3.19E-05 | 0.129605 | 0.112 | 0.051 | 0.989316 | 1 |
| Spag7 | 3.19E-05 | 0.097529 | 0.198 | 0.109 | 0.990289 | 1 |
| Atr | 3.20E-05 | 0.105743 | 0.135 | 0.065 | 0.994068 | 1 |
| Klhl24 | 3.22E-05 | 0.123542 | 0.659 | 0.469 | 0.999459 | 1 |
| Chmp1b | 3.38E-05 | 0.117674 | 0.149 | 0.074 | 1 | 1 |
| Ankle2 | 3.38E-05 | 0.151153 | 0.152 | 0.078 | 1 | 1 |
| Atp1a1 | 3.40E-05 | 0.122107 | 0.281 | 0.171 | 1 | 1 |
| Gm47283 | 3.47E-05 | 0.091786 | 0.361 | 0.229 | 1 | 1 |
| Grk2 | 3.49E-05 | 0.132009 | 0.564 | 0.377 | 1 | 1 |
| Gnai3 | 3.49E-05 | 0.109806 | 0.258 | 0.153 | 1 | 1 |
| Pes1 | 3.51E-05 | 0.08498 | 0.135 | 0.065 | 1 | 1 |
| Zbtb18 | 3.53E-05 | 0.106053 | 0.163 | 0.084 | 1 | 1 |
| Scrib | 3.58E-05 | 0.115414 | 0.112 | 0.051 | 1 | 1 |
| Ddx24 | 3.58E-05 | 0.133188 | 0.441 | 0.293 | 1 | 1 |
| Fam102a | 3.58E-05 | 0.094852 | 0.14 | 0.069 | 1 | 1 |
| Slc43a2 | 3.59E-05 | 0.097136 | 0.129 | 0.062 | 1 | 1 |
| Lrch1 | 3.64E-05 | 0.138635 | 0.152 | 0.078 | 1 | 1 |
| Zbtb37 | 3.65E-05 | 0.096996 | 0.135 | 0.065 | 1 | 1 |
| Smad2 | 3.67E-05 | 0.158703 | 0.149 | 0.077 | 1 | 1 |
| Eif4enif1 | 3.69E-05 | 0.109264 | 0.143 | 0.072 | 1 | 1 |
| Icam2 | 3.72E-05 | 0.109665 | 0.169 | 0.089 | 1 | 1 |
| Mrpl43 | 3.73E-05 | 0.070667 | 0.235 | 0.134 | 1 | 1 |
| Stat6 | 3.75E-05 | 0.113497 | 0.215 | 0.12 | 1 | 1 |
| Pwwp2a | 3.75E-05 | 0.073482 | 0.186 | 0.099 | 1 | 1 |
| Gle1 | 3.77E-05 | 0.107245 | 0.189 | 0.102 | 1 | 1 |
| Esco1 | 3.78E-05 | 0.105628 | 0.238 | 0.137 | 1 | 1 |
| Atf7 | 3.80E-05 | 0.115651 | 0.241 | 0.14 | 1 | 1 |
| Socs7 | 3.83E-05 | 0.120022 | 0.103 | 0.046 | 1 | 1 |
| Letm1 | 3.85E-05 | 0.12914 | 0.166 | 0.089 | 1 | 1 |
| Hdac7 | 3.86E-05 | 0.143709 | 0.16 | 0.085 | 1 | 1 |
| Cd79a | 3.88E-05 | 0.106711 | 0.997 | 0.976 | 1 | 1 |
| Cir1 | 3.89E-05 | 0.148505 | 0.198 | 0.111 | 1 | 1 |
| Hspa8 | 3.90E-05 | -0.18416 | 0.966 | 0.956 | 1 | 1 |
| Sec24a | 3.92E-05 | 0.116355 | 0.169 | 0.091 | 1 | 1 |
| Cebpg | 3.92E-05 | 0.130391 | 0.212 | 0.12 | 1 | 1 |
| Stk38 | 3.92E-05 | 0.132223 | 0.332 | 0.209 | 1 | 1 |
| Fbxl3 | 3.94E-05 | 0.134462 | 0.192 | 0.106 | 1 | 1 |
| Med1 | 3.96E-05 | 0.112277 | 0.269 | 0.16 | 1 | 1 |
| Ski | 3.99E-05 | 0.093042 | 0.138 | 0.069 | 1 | 1 |
| mt-Co1 | 4.03E-05 | -0.09194 | 1 | 0.999 | 1 | 1 |
| Spast | 4.05E-05 | 0.094872 | 0.123 | 0.058 | 1 | 1 |
| Cnp | 4.06E-05 | 0.17847 | 0.407 | 0.271 | 1 | 1 |
| Tsc22d2 | 4.12E-05 | 0.117599 | 0.375 | 0.239 | 1 | 1 |
| Ankrd10 | 4.14E-05 | 0.089085 | 0.195 | 0.107 | 1 | 1 |
| Pcnx | 4.14E-05 | 0.082324 | 0.115 | 0.053 | 1 | 1 |
| Mterf3 | 4.20E-05 | 0.080066 | 0.129 | 0.062 | 1 | 1 |
| Zdhhc18 | 4.21E-05 | 0.090827 | 0.281 | 0.167 | 1 | 1 |
| Slc38a1 | 4.24E-05 | 0.158112 | 0.533 | 0.381 | 1 | 1 |
| Exoc5 | 4.30E-05 | 0.094531 | 0.212 | 0.12 | 1 | 1 |
| Arap2 | 4.33E-05 | 0.116317 | 0.513 | 0.351 | 1 | 1 |
| Tagap | 4.40E-05 | 0.116596 | 0.181 | 0.098 | 1 | 1 |
| Nup153 | 4.41E-05 | 0.096383 | 0.192 | 0.106 | 1 | 1 |
| Chst15 | 4.41E-05 | 0.112334 | 0.16 | 0.083 | 1 | 1 |
| Opa1 | 4.42E-05 | 0.11137 | 0.123 | 0.059 | 1 | 1 |
| Gatad2a | 4.43E-05 | 0.065344 | 0.221 | 0.125 | 1 | 1 |
| Ctr9 | 4.47E-05 | 0.111054 | 0.129 | 0.063 | 1 | 1 |
| Ucp2 | 4.53E-05 | 0.156419 | 0.593 | 0.419 | 1 | 1 |
| Dad1 | 4.57E-05 | -0.27473 | 0.668 | 0.64 | 1 | 1 |
| Foxj3 | 4.59E-05 | 0.084954 | 0.126 | 0.06 | 1 | 1 |
| Sem1 | 4.60E-05 | -0.28323 | 0.564 | 0.553 | 1 | 1 |
| Tmem87a | 4.62E-05 | 0.099786 | 0.129 | 0.062 | 1 | 1 |
| Msrb1 | 4.67E-05 | -0.36011 | 0.146 | 0.228 | 1 | 1 |
| Kpnb1 | 4.67E-05 | 0.103256 | 0.221 | 0.128 | 1 | 1 |
| Crip1 | 4.78E-05 | -0.41635 | 0.602 | 0.637 | 1 | 1 |
| Gna13 | 4.84E-05 | 0.09435 | 0.453 | 0.295 | 1 | 1 |
| Snrk | 4.84E-05 | 0.100187 | 0.109 | 0.05 | 1 | 1 |
| Cul2 | 5.13E-05 | 0.102697 | 0.115 | 0.053 | 1 | 1 |
| Arrdc3 | 5.21E-05 | 0.136447 | 0.106 | 0.049 | 1 | 1 |
| Dnajc9 | 5.25E-05 | 0.113639 | 0.278 | 0.168 | 1 | 1 |
| Atp6ap1 | 5.31E-05 | 0.132176 | 0.258 | 0.156 | 1 | 1 |
| Rrm2b | 5.43E-05 | 0.111684 | 0.146 | 0.074 | 1 | 1 |
| Adar | 5.46E-05 | 0.097094 | 0.12 | 0.058 | 1 | 1 |
| Arfgef2 | 5.46E-05 | 0.075971 | 0.12 | 0.057 | 1 | 1 |
| Ccdc186 | 5.58E-05 | 0.135858 | 0.146 | 0.075 | 1 | 1 |
| Dnajc7 | 5.60E-05 | 0.137564 | 0.436 | 0.286 | 1 | 1 |
| Pold1 | 5.62E-05 | 0.143395 | 0.158 | 0.085 | 1 | 1 |
| Rps27rt | 5.62E-05 | -0.24304 | 0.828 | 0.795 | 1 | 1 |
| Rpl36al | 5.63E-05 | -0.21181 | 0.788 | 0.732 | 1 | 1 |
| Actr2 | 5.64E-05 | 0.12823 | 0.63 | 0.444 | 1 | 1 |
| Shisa5 | 5.71E-05 | 0.147225 | 0.88 | 0.791 | 1 | 1 |
| Prkacb | 5.74E-05 | 0.103039 | 0.183 | 0.101 | 1 | 1 |
| Smarcd2 | 5.81E-05 | 0.136326 | 0.203 | 0.117 | 1 | 1 |
| Khsrp | 5.83E-05 | 0.097454 | 0.163 | 0.086 | 1 | 1 |
| Setd1b | 5.85E-05 | 0.136346 | 0.149 | 0.078 | 1 | 1 |
| Lmbr1l | 5.87E-05 | 0.097209 | 0.158 | 0.082 | 1 | 1 |
| Wnk1 | 5.88E-05 | 0.135182 | 0.708 | 0.508 | 1 | 1 |
| Kdm4a | 5.98E-05 | 0.082782 | 0.109 | 0.05 | 1 | 1 |
| Rbm39 | 6.07E-05 | 0.087026 | 0.971 | 0.873 | 1 | 1 |
| Ppp2ca | 6.09E-05 | 0.112747 | 0.476 | 0.322 | 1 | 1 |
| Trim44 | 6.14E-05 | 0.100865 | 0.244 | 0.144 | 1 | 1 |
| Blnk | 6.18E-05 | 0.155098 | 0.484 | 0.331 | 1 | 1 |
| Git2 | 6.19E-05 | 0.113413 | 0.464 | 0.31 | 1 | 1 |
| Klhl6 | 6.21E-05 | 0.118514 | 0.281 | 0.169 | 1 | 1 |
| Ppp3cb | 6.24E-05 | 0.153208 | 0.215 | 0.126 | 1 | 1 |
| Tmed7 | 6.28E-05 | 0.10697 | 0.209 | 0.12 | 1 | 1 |
| Polg | 6.30E-05 | 0.123394 | 0.126 | 0.062 | 1 | 1 |
| Srrt | 6.31E-05 | 0.101459 | 0.241 | 0.142 | 1 | 1 |
| Bicral | 6.37E-05 | 0.123441 | 0.138 | 0.069 | 1 | 1 |
| Tfeb | 6.38E-05 | 0.097837 | 0.146 | 0.076 | 1 | 1 |
| Atxn7l3 | 6.42E-05 | 0.067813 | 0.126 | 0.061 | 1 | 1 |
| Szrd1 | 6.47E-05 | 0.113994 | 0.203 | 0.116 | 1 | 1 |
| Fcmr | 6.54E-05 | 0.128668 | 0.814 | 0.657 | 1 | 1 |
| Stat1 | 6.65E-05 | 0.140399 | 0.324 | 0.208 | 1 | 1 |
| Faf1 | 6.72E-05 | 0.102116 | 0.129 | 0.064 | 1 | 1 |
| Snrnp200 | 6.73E-05 | 0.164784 | 0.206 | 0.122 | 1 | 1 |
| Srpk1 | 6.77E-05 | 0.11027 | 0.252 | 0.151 | 1 | 1 |
| Hdac5 | 6.79E-05 | 0.071157 | 0.103 | 0.046 | 1 | 1 |
| Mcm9 | 6.85E-05 | 0.085794 | 0.106 | 0.048 | 1 | 1 |
| Pptc7 | 6.92E-05 | 0.125631 | 0.155 | 0.082 | 1 | 1 |
| Fam192a | 6.97E-05 | 0.101231 | 0.149 | 0.077 | 1 | 1 |
| 4833420G17Rik | 7.04E-05 | 0.092627 | 0.232 | 0.135 | 1 | 1 |
| Lpcat1 | 7.17E-05 | 0.092597 | 0.175 | 0.095 | 1 | 1 |
| Cbfa2t3 | 7.19E-05 | 0.123283 | 0.109 | 0.051 | 1 | 1 |
| Tnpo3 | 7.27E-05 | 0.125245 | 0.223 | 0.133 | 1 | 1 |
| Mfsd14b | 7.38E-05 | 0.073713 | 0.129 | 0.063 | 1 | 1 |
| Cic | 7.49E-05 | 0.099797 | 0.192 | 0.108 | 1 | 1 |
| Serinc1 | 7.62E-05 | 0.097929 | 0.384 | 0.243 | 1 | 1 |
| Nol11 | 7.67E-05 | 0.092804 | 0.14 | 0.071 | 1 | 1 |
| Tmem57 | 7.78E-05 | 0.078411 | 0.186 | 0.103 | 1 | 1 |
| Cdkn1b | 7.93E-05 | 0.082799 | 0.527 | 0.347 | 1 | 1 |
| Trpc4ap | 7.99E-05 | 0.144413 | 0.258 | 0.16 | 1 | 1 |
| Kctd20 | 8.11E-05 | 0.084481 | 0.117 | 0.056 | 1 | 1 |
| Dctn1 | 8.13E-05 | 0.090675 | 0.126 | 0.062 | 1 | 1 |
| Epb41l2 | 8.21E-05 | 0.127829 | 0.223 | 0.134 | 1 | 1 |
| Mical1 | 8.22E-05 | 0.093473 | 0.126 | 0.062 | 1 | 1 |
| Dip2b | 8.28E-05 | 0.115294 | 0.244 | 0.148 | 1 | 1 |
| Spata2 | 8.30E-05 | 0.057432 | 0.112 | 0.052 | 1 | 1 |
| Trio | 8.32E-05 | 0.106969 | 0.112 | 0.053 | 1 | 1 |
| Mapk14 | 8.40E-05 | 0.102364 | 0.135 | 0.069 | 1 | 1 |
| Ube2k | 8.41E-05 | 0.057008 | 0.375 | 0.237 | 1 | 1 |
| Cebpz | 8.52E-05 | 0.074445 | 0.441 | 0.288 | 1 | 1 |
| Zmiz1 | 8.57E-05 | 0.093856 | 0.261 | 0.157 | 1 | 1 |
| Wbp2 | 8.57E-05 | 0.086305 | 0.275 | 0.164 | 1 | 1 |
| Srsf10 | 8.64E-05 | 0.115874 | 0.524 | 0.347 | 1 | 1 |
| Sirt7 | 8.65E-05 | 0.112585 | 0.229 | 0.137 | 1 | 1 |
| Appl1 | 8.74E-05 | 0.114618 | 0.198 | 0.114 | 1 | 1 |
| Osbpl9 | 8.79E-05 | 0.104766 | 0.292 | 0.185 | 1 | 1 |
| Lemd2 | 8.94E-05 | 0.073345 | 0.126 | 0.062 | 1 | 1 |
| Cep44 | 8.96E-05 | 0.085704 | 0.109 | 0.051 | 1 | 1 |
| Klf16 | 9.00E-05 | 0.078744 | 0.112 | 0.053 | 1 | 1 |
| Eri1 | 9.05E-05 | 0.068233 | 0.178 | 0.098 | 1 | 1 |
| Zc3h14 | 9.10E-05 | 0.05272 | 0.178 | 0.096 | 1 | 1 |
| Fam111a | 9.13E-05 | 0.149564 | 0.318 | 0.207 | 1 | 1 |
| Mier1 | 9.16E-05 | 0.100122 | 0.461 | 0.31 | 1 | 1 |
| Afg3l1 | 9.17E-05 | 0.130251 | 0.115 | 0.056 | 1 | 1 |
| Skil | 9.18E-05 | 0.081672 | 0.43 | 0.286 | 1 | 1 |
| Kdm2a | 9.18E-05 | 0.111481 | 0.41 | 0.269 | 1 | 1 |
| Rgs1 | 9.18E-05 | -0.33214 | 0.066 | 0.139 | 1 | 1 |
| Limd2 | 9.22E-05 | 0.122197 | 0.639 | 0.446 | 1 | 1 |
| Slc25a28 | 9.22E-05 | 0.076822 | 0.158 | 0.083 | 1 | 1 |
| Cyth2 | 9.23E-05 | 0.086633 | 0.178 | 0.097 | 1 | 1 |
| Ddx27 | 9.26E-05 | 0.103733 | 0.186 | 0.105 | 1 | 1 |
| Fam49b | 9.32E-05 | 0.134997 | 0.65 | 0.444 | 1 | 1 |
| Zfp622 | 9.35E-05 | 0.112354 | 0.264 | 0.16 | 1 | 1 |
| Daxx | 9.35E-05 | 0.067446 | 0.103 | 0.047 | 1 | 1 |
| Clcn4 | 9.51E-05 | 0.106982 | 0.146 | 0.078 | 1 | 1 |
| Tle3 | 9.60E-05 | 0.116232 | 0.158 | 0.085 | 1 | 1 |
| Trappc8 | 9.68E-05 | 0.080674 | 0.149 | 0.078 | 1 | 1 |
| Atxn2l | 9.73E-05 | 0.071397 | 0.327 | 0.203 | 1 | 1 |
| Gm30054 | 9.79E-05 | 0.100047 | 0.106 | 0.049 | 1 | 1 |
| Rpa1 | 9.84E-05 | 0.109467 | 0.138 | 0.071 | 1 | 1 |
| Ncaph2 | 9.84E-05 | 0.08638 | 0.132 | 0.066 | 1 | 1 |
| Srsf3 | 0.000100011 | 0.110558 | 0.699 | 0.503 | 1 | 1 |
| Hivep1 | 0.000100244 | 0.107688 | 0.175 | 0.098 | 1 | 1 |
| Hsp90b1 | 0.000100341 | 0.03036 | 0.691 | 0.492 | 1 | 1 |
| Mrpl33 | 0.000100481 | -0.30972 | 0.181 | 0.26 | 1 | 1 |
| Dlst | 0.000102366 | 0.087243 | 0.215 | 0.125 | 1 | 1 |
| Dnajc3 | 0.000102849 | 0.114892 | 0.249 | 0.153 | 1 | 1 |
| Isg20l2 | 0.000103026 | 0.09976 | 0.132 | 0.067 | 1 | 1 |
| Dhx30 | 0.000103415 | 0.093574 | 0.169 | 0.092 | 1 | 1 |
| Kat6b | 0.000103651 | 0.109602 | 0.152 | 0.081 | 1 | 1 |
| Gem | 0.000103817 | -0.3696 | 0.158 | 0.237 | 1 | 1 |
| Fndc3a | 0.000103878 | 0.124193 | 0.212 | 0.126 | 1 | 1 |
| Cbx5 | 0.000104365 | 0.111449 | 0.143 | 0.076 | 1 | 1 |
| Scaf4 | 0.000104798 | 0.094676 | 0.112 | 0.053 | 1 | 1 |
| Jade1 | 0.000105847 | 0.101622 | 0.138 | 0.071 | 1 | 1 |
| Cggbp1 | 0.000106121 | 0.104479 | 0.444 | 0.299 | 1 | 1 |
| Lcp1 | 0.00010632 | 0.145722 | 0.725 | 0.539 | 1 | 1 |
| Cnot2 | 0.000106876 | 0.069123 | 0.221 | 0.129 | 1 | 1 |
| Sat1 | 0.000108295 | -0.32846 | 0.441 | 0.479 | 1 | 1 |
| Pitpnb | 0.000108411 | 0.088714 | 0.146 | 0.077 | 1 | 1 |
| Ankrd44 | 0.000108607 | 0.120602 | 0.562 | 0.401 | 1 | 1 |
| Zfyve26 | 0.000109312 | 0.075375 | 0.143 | 0.073 | 1 | 1 |
| Zfp608 | 0.00010959 | 0.13676 | 0.232 | 0.138 | 1 | 1 |
| Mia2 | 0.000109957 | 0.14359 | 0.266 | 0.169 | 1 | 1 |
| Tnrc18 | 0.000110586 | 0.086023 | 0.143 | 0.074 | 1 | 1 |
| Gramd1a | 0.000110824 | 0.086328 | 0.192 | 0.107 | 1 | 1 |
| Gtf2h1 | 0.000112286 | 0.10666 | 0.126 | 0.064 | 1 | 1 |
| Secisbp2l | 0.000112675 | 0.076732 | 0.149 | 0.078 | 1 | 1 |
| Tars | 0.000112783 | 0.126233 | 0.112 | 0.055 | 1 | 1 |
| Ldha | 0.000113335 | -0.35289 | 0.301 | 0.355 | 1 | 1 |
| Zdhhc5 | 0.000117204 | 0.078551 | 0.135 | 0.069 | 1 | 1 |
| Nfkbie | 0.000117694 | 0.097086 | 0.186 | 0.106 | 1 | 1 |
| Vbp1 | 0.000118332 | 0.068949 | 0.252 | 0.151 | 1 | 1 |
| Ube3c | 0.000119046 | 0.098611 | 0.149 | 0.078 | 1 | 1 |
| Rubcnl | 0.000120391 | 0.0965 | 0.106 | 0.05 | 1 | 1 |
| Mef2c | 0.000121778 | 0.111361 | 0.926 | 0.829 | 1 | 1 |
| Tnfrsf13b | 0.000122016 | 0.098159 | 0.275 | 0.171 | 1 | 1 |
| Serp1 | 0.000122412 | 0.12863 | 0.871 | 0.732 | 1 | 1 |
| Stk24 | 0.000122781 | 0.105195 | 0.493 | 0.336 | 1 | 1 |
| Tecpr1 | 0.00012437 | 0.079177 | 0.166 | 0.091 | 1 | 1 |
| Dusp11 | 0.000124412 | 0.101693 | 0.461 | 0.307 | 1 | 1 |
| Brd1 | 0.000124573 | 0.128154 | 0.355 | 0.232 | 1 | 1 |
| Ikzf1 | 0.000124805 | 0.141882 | 0.404 | 0.277 | 1 | 1 |
| Uba2 | 0.000125155 | 0.11343 | 0.186 | 0.107 | 1 | 1 |
| 2410002F23Rik | 0.000126695 | 0.064097 | 0.152 | 0.08 | 1 | 1 |
| Orc4 | 0.000127798 | 0.135524 | 0.149 | 0.081 | 1 | 1 |
| Pcmtd1 | 0.000127865 | 0.109663 | 0.364 | 0.241 | 1 | 1 |
| Ints4 | 0.000129539 | 0.097598 | 0.112 | 0.055 | 1 | 1 |
| Coro1b | 0.000129865 | 0.094364 | 0.155 | 0.083 | 1 | 1 |
| Nop53 | 0.00013 | 0.128919 | 0.716 | 0.539 | 1 | 1 |
| Dennd5b | 0.000130164 | 0.112523 | 0.258 | 0.158 | 1 | 1 |
| Cdc37 | 0.000131078 | 0.110703 | 0.358 | 0.234 | 1 | 1 |
| Cse1l | 0.000131937 | 0.104811 | 0.244 | 0.146 | 1 | 1 |
| Sppl2a | 0.000132049 | 0.065197 | 0.321 | 0.203 | 1 | 1 |
| Epn1 | 0.000132981 | 0.076834 | 0.281 | 0.173 | 1 | 1 |
| Tcf4 | 0.000133918 | 0.164299 | 0.493 | 0.345 | 1 | 1 |
| Traf3 | 0.000134845 | 0.118198 | 0.146 | 0.078 | 1 | 1 |
| Mprip | 0.000134849 | 0.109475 | 0.186 | 0.105 | 1 | 1 |
| Thap12 | 0.000134866 | 0.076741 | 0.126 | 0.064 | 1 | 1 |
| Dusp3 | 0.000135727 | 0.121736 | 0.132 | 0.068 | 1 | 1 |
| Cul1 | 0.000135786 | 0.098173 | 0.246 | 0.15 | 1 | 1 |
| Nrbf2 | 0.000136894 | 0.098552 | 0.126 | 0.064 | 1 | 1 |
| Spg7 | 0.000138637 | 0.10376 | 0.135 | 0.07 | 1 | 1 |
| 2010007H06Rik | 0.00014151 | 0.114176 | 0.129 | 0.066 | 1 | 1 |
| Sf3b4 | 0.000141641 | 0.086058 | 0.155 | 0.083 | 1 | 1 |
| Mfsd4a | 0.000142397 | 0.123998 | 0.189 | 0.108 | 1 | 1 |
| Ulk2 | 0.000142798 | 0.136582 | 0.117 | 0.058 | 1 | 1 |
| Rmi1 | 0.000143279 | 0.1074 | 0.12 | 0.06 | 1 | 1 |
| Rad23b | 0.000144472 | 0.135296 | 0.255 | 0.161 | 1 | 1 |
| 2900097C17Rik | 0.000145299 | 0.09065 | 0.183 | 0.105 | 1 | 1 |
| Hp1bp3 | 0.000145773 | 0.091905 | 0.355 | 0.231 | 1 | 1 |
| Katna1 | 0.000147256 | 0.111759 | 0.135 | 0.07 | 1 | 1 |
| Nfe2l1 | 0.000147696 | 0.091653 | 0.175 | 0.098 | 1 | 1 |
| Snw1 | 0.000148556 | 0.13015 | 0.401 | 0.27 | 1 | 1 |
| Herc4 | 0.000148892 | 0.10067 | 0.347 | 0.224 | 1 | 1 |
| Spop | 0.000149088 | 0.097427 | 0.272 | 0.168 | 1 | 1 |
| Klc1 | 0.000149908 | 0.089981 | 0.103 | 0.049 | 1 | 1 |
| Dnajc1 | 0.00015146 | 0.126575 | 0.158 | 0.088 | 1 | 1 |
| Hspa9 | 0.000151688 | 0.107729 | 0.272 | 0.169 | 1 | 1 |
| Snx27 | 0.000151718 | 0.094781 | 0.126 | 0.064 | 1 | 1 |
| Hif1an | 0.000154682 | 0.076178 | 0.175 | 0.096 | 1 | 1 |
| Gfpt1 | 0.000156319 | 0.103 | 0.117 | 0.058 | 1 | 1 |
| Prorsd1 | 0.000156563 | 0.121438 | 0.181 | 0.103 | 1 | 1 |
| Naa30 | 0.000156813 | 0.109573 | 0.106 | 0.051 | 1 | 1 |
| Gnl3l | 0.000157447 | 0.093935 | 0.112 | 0.055 | 1 | 1 |
| Zmym4 | 0.000159173 | 0.09411 | 0.129 | 0.066 | 1 | 1 |
| Kansl2 | 0.000159245 | 0.080884 | 0.143 | 0.076 | 1 | 1 |
| Vcp | 0.000159308 | 0.140352 | 0.372 | 0.25 | 1 | 1 |
| Fip1l1 | 0.000159323 | 0.101855 | 0.335 | 0.216 | 1 | 1 |
| Man2a2 | 0.000159385 | 0.084222 | 0.143 | 0.076 | 1 | 1 |
| Ube2r2 | 0.000159545 | 0.102113 | 0.258 | 0.16 | 1 | 1 |
| Pip4k2c | 0.000161225 | 0.118144 | 0.117 | 0.058 | 1 | 1 |
| Rab11fip2 | 0.000161393 | 0.091047 | 0.146 | 0.078 | 1 | 1 |
| Nsd2 | 0.000161968 | 0.105469 | 0.117 | 0.059 | 1 | 1 |
| Vps26a | 0.000162372 | 0.052784 | 0.129 | 0.065 | 1 | 1 |
| Ddx46 | 0.000162389 | 0.121224 | 0.361 | 0.241 | 1 | 1 |
| Sik2 | 0.000164146 | 0.081409 | 0.146 | 0.077 | 1 | 1 |
| Usp12 | 0.00016451 | 0.047934 | 0.129 | 0.065 | 1 | 1 |
| Amfr | 0.000165231 | 0.097172 | 0.169 | 0.094 | 1 | 1 |
| Sell | 0.000165901 | 0.128312 | 0.656 | 0.492 | 1 | 1 |
| Laptm5 | 0.000166151 | 0.105955 | 0.86 | 0.703 | 1 | 1 |
| Smc6 | 0.000166258 | 0.13091 | 0.433 | 0.306 | 1 | 1 |
| Vps13c | 0.000167192 | 0.098955 | 0.103 | 0.049 | 1 | 1 |
| Vps4b | 0.000167356 | 0.068904 | 0.241 | 0.146 | 1 | 1 |
| Cds2 | 0.000169712 | 0.084244 | 0.146 | 0.078 | 1 | 1 |
| Zcchc7 | 0.000170983 | 0.178959 | 0.521 | 0.38 | 1 | 1 |
| Lats2 | 0.000173868 | 0.062791 | 0.223 | 0.131 | 1 | 1 |
| Fbrsl1 | 0.000174246 | 0.080208 | 0.146 | 0.078 | 1 | 1 |
| Ube2d2a | 0.000176077 | 0.087008 | 0.39 | 0.257 | 1 | 1 |
| Ppp1cc | 0.000178214 | 0.061156 | 0.393 | 0.257 | 1 | 1 |
| N4bp2l2 | 0.000178794 | 0.110532 | 0.501 | 0.341 | 1 | 1 |
| Srsf4 | 0.000181091 | 0.10719 | 0.14 | 0.075 | 1 | 1 |
| Bsdc1 | 0.000181694 | 0.061176 | 0.166 | 0.091 | 1 | 1 |
| Pik3c2b | 0.000182483 | 0.072625 | 0.126 | 0.064 | 1 | 1 |
| Mrpl9 | 0.000182683 | 0.093681 | 0.149 | 0.08 | 1 | 1 |
| Ikbkb | 0.000186343 | 0.101779 | 0.496 | 0.333 | 1 | 1 |
| Cand1 | 0.000186462 | 0.09613 | 0.149 | 0.081 | 1 | 1 |
| Tes | 0.000187143 | 0.063922 | 0.181 | 0.102 | 1 | 1 |
| Zfp131 | 0.000189801 | 0.095504 | 0.201 | 0.119 | 1 | 1 |
| Tuba4a | 0.000190637 | 0.084205 | 0.33 | 0.212 | 1 | 1 |
| 4921524J17Rik | 0.000191393 | 0.058306 | 0.203 | 0.117 | 1 | 1 |
| Pdpk1 | 0.000192412 | 0.067168 | 0.181 | 0.101 | 1 | 1 |
| H3f3a | 0.000192501 | -0.10678 | 0.991 | 0.994 | 1 | 1 |
| Txlna | 0.000192576 | 0.075951 | 0.172 | 0.096 | 1 | 1 |
| Tlk2 | 0.000193656 | 0.104567 | 0.16 | 0.089 | 1 | 1 |
| Rab5b | 0.000194187 | 0.071368 | 0.129 | 0.066 | 1 | 1 |
| Tap1 | 0.000195557 | 0.075037 | 0.318 | 0.203 | 1 | 1 |
| Trim30a | 0.000197976 | 0.131355 | 0.212 | 0.127 | 1 | 1 |
| Tmed8 | 0.000199178 | 0.085663 | 0.109 | 0.053 | 1 | 1 |
| Ell2 | 0.000199343 | 0.091454 | 0.215 | 0.129 | 1 | 1 |
| Ypel5 | 0.000199603 | 0.064605 | 0.221 | 0.13 | 1 | 1 |
| Col4a3bp | 0.000200933 | 0.093726 | 0.152 | 0.083 | 1 | 1 |
| Cenpb | 0.000202533 | 0.109495 | 0.175 | 0.099 | 1 | 1 |
| Taf3 | 0.000202941 | 0.090654 | 0.175 | 0.099 | 1 | 1 |
| Map3k1 | 0.000203298 | 0.098302 | 0.593 | 0.413 | 1 | 1 |
| Lrp6 | 0.000203763 | 0.103276 | 0.126 | 0.065 | 1 | 1 |
| Rhobtb2 | 0.000204624 | 0.102253 | 0.103 | 0.05 | 1 | 1 |
| Elk4 | 0.000204775 | 0.106322 | 0.327 | 0.214 | 1 | 1 |
| Zeb2 | 0.000206773 | 0.154718 | 0.266 | 0.171 | 1 | 1 |
| Tor1aip1 | 0.000209129 | 0.078672 | 0.361 | 0.236 | 1 | 1 |
| Tubgcp4 | 0.000209461 | 0.058218 | 0.146 | 0.077 | 1 | 1 |
| Fopnl | 0.000209504 | 0.102915 | 0.123 | 0.064 | 1 | 1 |
| Trim24 | 0.000210273 | 0.127465 | 0.206 | 0.122 | 1 | 1 |
| A630001G21Rik | 0.000210377 | 0.091551 | 0.341 | 0.222 | 1 | 1 |
| Ube2q1 | 0.000210938 | 0.030599 | 0.244 | 0.144 | 1 | 1 |
| Insig1 | 0.000211511 | 0.108309 | 0.135 | 0.071 | 1 | 1 |
| Plekhb2 | 0.000217152 | 0.089684 | 0.123 | 0.063 | 1 | 1 |
| Myo9b | 0.00021871 | 0.102146 | 0.138 | 0.073 | 1 | 1 |
| Pck2 | 0.000218947 | 0.102503 | 0.158 | 0.087 | 1 | 1 |
[truncated: 318,053 more chars]
